# Introduction of Multiple Novel High Pathogenicity Avian Influenza (H5N1) Virus of Clade 2.3.4.4b into South Korea in 2022

**DOI:** 10.1155/2023/8339427

**Published:** 2023-04-13

**Authors:** Yong-Myung Kang, Gyeong-Beom Heo, Se-Hee An, Yu-Na Lee, Ra Mi Cha, Hyun-Kyu Cho, Mingeun Sagong, Dong-Hyun Kim, Eun-Kyoung Lee, Hyun-Mi Kang, Kwang-Nyeong Lee, Youn-Jeong Lee

**Affiliations:** Animal and Plant Quarantine Agency, 177 Hyeoksin 8-ro, Gimcheon-si, Gyeongsangbuk-do 39660, Republic of Korea

## Abstract

Since October 2020, H5N1 clade 2.3.4.4b high pathogenicity avian influenza (HPAI) viruses have spread to many countries. Although these viruses evolved from Eurasian ancestors, reassortant with other LPAI viruses has generated various genotypes. Here, we identified three H5N1 HPAI viruses belonging to clade 2.3.4.4b; these viruses were isolated from mandarin duck, common teal, and domestic breeder ducks in October 2022 during an avian influenza (AI) active surveillance program. Two of the H5N1 viruses (MD/WA496 and BD/H493) have been found sporadically in China, Russia, and Korea. It is presumed that two viruses with a similar gene constellation isolated in Russia, China, and Korea were introduced into the breeding area during the spring migration, and were introduced newly to Korea during the autumn migration. Due to international bird migration, the other virus (CT/WA537) is most similar (99.3–99.8%) to a virus detected in North Dakota, USA in April 2022. These results suggest that H5N1 viruses with at least two genotypes were introduced at the same time into Korea during the autumn of 2022, and that they originated from Eurasian breeding grounds and North America. Thus, multiple 2.3.4.4b H5N1 viruses were introduced into Korea during the autumn season of 2022.

## 1. Introduction

The H5Nx high pathogenicity avian influenza (HPAI) virus that evolved from A/goose/Guangdong/1/1996 (H5N1) and spread across Asia, Europe, Africa, and North America poses a continuous threat to both animal and human health [1, 2]. Circulating H5 viruses have undergone extensive evolution into different clades (0–9) through accumulation of point mutations and reassortment with other influenza viruses [3–5]. First reported in China in 2013, clade 2.3.4.4 viruses have become the dominant strain worldwide [6, 7].

The H5N8 clade 2.3.4.4b HPAI virus was first isolated from domestic ducks in Eastern China in 2013, and then in Korea in 2014 [8, 9]. Since 2016, H5N8 clade 2.3.4.4b HPAI have been reported in Europe, Asia, and Africa, causing considerable economic damage [10, 11]. H5N1 clade 2.3.4.4b HPAI viruses have spread to many countries since being reported in the Netherlands in October 2020 [3, 12]. Genetic spatial analysis indicated that these Eurasian-origin viruses were spread from Europe to Africa and North America by wild birds [12–14]. H5N1 clade 2.3.4.4b viruses evolved from Eurasian ancestors into various genotypes through reassortment with other LPAI viruses [15]. Since 2003, wild birds have introduced H5Nx HPAI viruses of various clades and gene constellations into Korea [9, 16–21]. In particular, clade 2.3.4.4b viruses have been introduced by wild birds since 2017; these have become the main cause of HPAI outbreaks.

During AI active surveillance in October 2022, we isolated novel H5N1 viruses, which we presumed to be newly introduced, from a captured mandarin duck (*Aix galericulata*), a common teal (*Anas crecca*), and birds on a breeder duck farm. Here, we performed genetic analysis of these viruses to identify their origin and relationship with clade 2.3.4.4b H5N1 viruses that are currently prevalent in Asia, Europe, Africa, and the USA.

## 2. Materials and Methods

For AI active surveillance, 1,000 wild birds were captured annually by the Livestock Health control association (LHCA). Oropharyngeal (OP) and cloacal (CL) swabs obtained from captured birds were sent to the Animal and Plant Quarantine Agency (APQA). On October 10, during the 2022/2023 season, the H5 gene was detected for the first time in 1/18 captured mandarin ducks in Cheonan (Chungnam province). Subsequently, the H5 gene was detected in one out of the captured common teal in Gimhae (Gyeongnam province).

AI active surveillance was increased during the winter season, particularly among domestic ducks. Every 2 weeks, the provincial veterinary service laboratory collected swab samples from 20 birds per house for screening; these were sent to APQA. On October 17, 2022, the H5 gene of AIV was detected on a domestic breeder duck farm in Yecheon (Gyeongbuk Province). Samples from wild birds and the poultry farm were analyzed by real time reverse transcription polymerase chain reaction (rRT-PCR) and RT-PCR, as described previously [22]. Briefly, extracted RNA was screened by rRT-PCR to detect the H5 gene. Positive samples were subjected to H5 subtype-specific RT-PCR, followed by sequence analysis using an Applied Biosystems Genetic Analyzer (ABI 3500xL, USA) to confirm the cleavage site within the H5 HA gene.

To conduct a full genome analysis, samples positive for the M and H5 gene were inoculated into 10-day-old embryonated chicken eggs and incubated at 37°C. The complete genomes of the isolated viruses were subjected to next-generation sequencing on the Illumina Miseq platform using the Nextera DNA Flex Library Prep Kit (Illumina, San Diego, CA, USA) as described previously [21]. The nucleotide sequences of the viruses isolated in this study were deposited in the GISAID database under accession numbers EPI_ISL_15647834–EPI_ISL_15647837. For phylogenetic analysis, the reference datasets for all gene segments used in this study were downloaded from the GISAID EpiFlu database (Table S1). All sequences for each segment were aligned using MAFFT (https://phylo.org) and edited using Bioedit 7.2.5. Phylogenetic trees were built using RAxML version 8.2.12, with default parameters and a general time-reversible model. Bayesian Markov Chain Monte Carlo (MCMC)-based methods were conducted in BEAST version 1.8.1 to estimate the time of the most recent common ancestor (tMRCA) of the reassortant viruses (Table S2), as described previously [23].

## 3. Results and Discussion

We screened the original samples for AIV genes within 24 h after receiving them because rapid detection and accurate identification of HPAI are critical if we are to control infections in poultry. We amplified the H5 genes from mandarin duck, common teal, and domestic breeder ducks. The H5 genes were identified as H5 HPAI due to the presence of multiple basic amino acids at the cleavage site of the HA gene (PLREKRRKR/G). The animal health authorities in Korea implement rapid control measures for HPAI, including stamping out of infected poultry, movement restrictions, and disinfection.

The H5N1 viruses isolated from mandarin duck, common teal, and the domestic breeder duck farm were named A/mandarin duck/Korea/WA496/2022 (H5N1), referred to hereafter as MD/WA496; A/common teal/Korea/WA537/2022 (H5N1), referred to hereafter as CT/WA537; and A/duck/Korea/H493/2022 (H5N1), referred to hereafter as BD/H493, respectively (Table S3).

**Figure 1:**
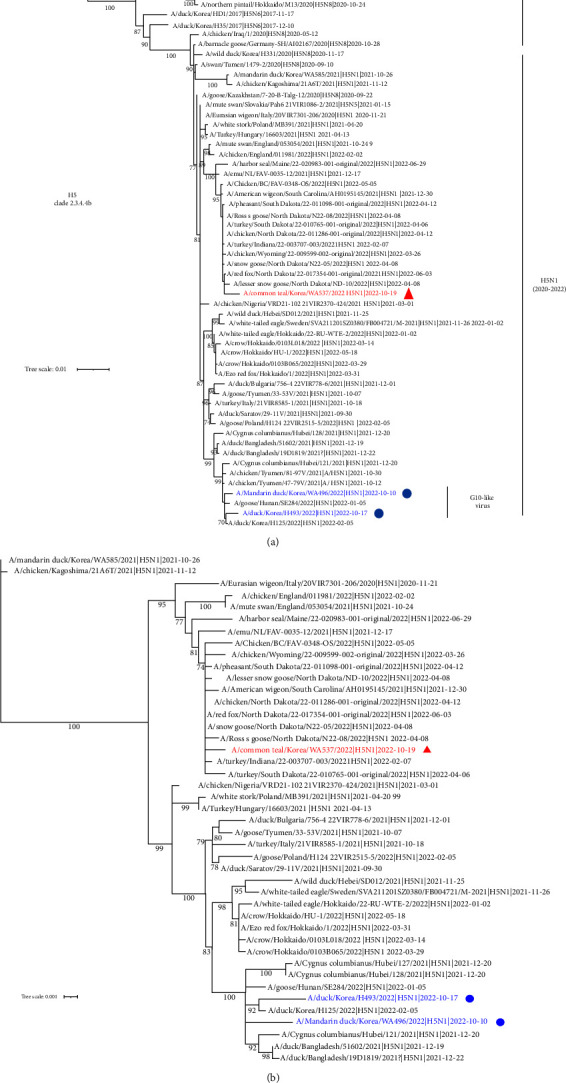
Maximum-likelihood phylogenetic tree for the hemagglutinin (HA) and neuraminidase (NA) genes. The phylogenetic tree is based on H5N1 viruses isolated recently, as well as other H5Nx viruses. Bootstrap values (1,000 replicates) >70% are displayed at the branch nodes. A/mandarin duck/Korea/WA496/2022 (H5N1) and A/breeder duck/Korea/H493/2022 (H5N1) are indicated by a blue solid circle. A/common teal/Korea/WA537/2022 (H5N1) is indicated by a red solid triangle. The scale bar indicates the number of nucleotide substitutions per site. (a) Phylogenetic tree for HA. (b) Phylogenetic tree for NA.

To identify the source of the three H5N1 viruses isolated this autumn, we performed phylogenetic analysis of the HA and NA genes, and compared them with that of other clade 2.3.4.4b H5Nx viruses (Figure 1). The phylogenetic tree shows that MD/WA496 and BD/H493 are relatively close to the Eurasian H5N1 2.3.4.4b subgroup, and that CT/WA537 is closely related to the North American H5N1 2.3.4.4b subgroup. With respect to the HA and NA genes, MD/WA496 and BD/H493 show greatest similarity to a strain identified in China in January 2022 (A/goose/Hunan/SE284/2022) and in Korea in February 2022 (A/duck/Korea/H125/2022), whereas CT/WA537 is similar to North American lineages represented by A/lesser snow goose/North Dakota/ND-10/2022 and A/skunk/Washington/22-019274-001-original/2022 (Table 1).

**Figure 2:**
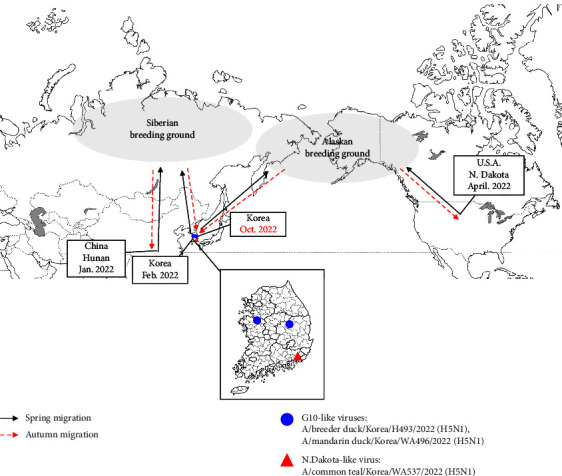
Transmission of H5N1 high pathogenicity avian influenza virus genotypes in Korea (autumn 2022) through migratory movements of wild birds. The areas presumed to be breeding grounds are indicated by gray ovals. G10-like viruses are indicated by solid blue circles. ND-like viruses are indicated by a solid red triangle. The spring migration is indicated by black arrows, and the autumn migration is indicated by dotted red arrows.

A recent study suggests that H5N1 2.3.4.4b viruses detected worldwide after 2020 could be classified viruses into at least 14 groups (G1 to G14) according to differences in gene constellation [3]. G1 viruses were first detected in Europe, and later circulated in Africa, North America, and China. G10 viruses harbor seven genes that are similar to those of the G1 virus, with only the PB2 gene differing markedly from that of the G1 virus. In present study, we found that seven of the eight genes from two H5N1 viruses (MD/WA496 and BD/H493) were similar to those of the current major genotype H5N1 clade 2.3.4.4b (98.9–99.7%); only the PB2 showed a marked difference (90.1–91.2% homology) (Figure S1). As a result, both viruses (MD/WA496 and BD/H493) were classified into the G10-like group, which has been found sporadically in China, Russia, and Korea. In Korea, H5N1 viruses belonging to the G10-like group were detected in February 2022, although G10 was not a major genotype that circulated in the 2021/2022 winter season [21]. To estimate the origin of these G10-like viruses, including MD/WA496 and BD/H493, we performed tMRCA analysis of time-scaled phylogenetic maximum clade credibility trees; the results suggest that the putative common ancestor of these viruses emerged between February 2021 and November 2021 (95% highest posterior density (HPD), May 2019 to January 2022; Table 2) via reassortment between epidemic H5N1 and LPAI viruses harboring a PB2 gene similar to that of G10-like viruses (Table 2). According to tMRCA analysis, common ancestors of G10-like viruses first appeared during the summer season of 2021 (probably in breeding areas), and G10-like viruses seem to have diversified by accumulating point mutations. Indeed, the G10-like viruses isolated last season differ from those isolated this season by approximately 0.3–1.1%; in addition, there is a 0.5–1.2% difference between the MD/WA496 and BD/H493 viruses isolated this autumn. Furthermore, it is presumed that the G10-like viruses from Russia, China, and Korea were introduced into breeding areas during the spring migration, and the newly identified viruses were introduced into Korea during the autumn migration of 2022 (Figure 2). This notion is supported by the finding that no HPAI virus was detected for 6 months (including the summer) since the last outbreak in April 2022, despite intensive AIV active surveillance. Nevertheless, it may be possible that the G10-like viruses were not detected due to a very low prevalence in wild birds.

**Figure 3:**
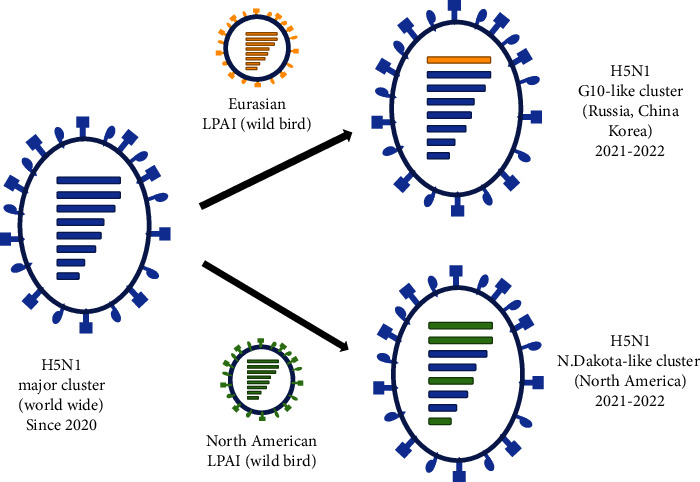
Schematic diagram showing the probable genesis of H5N1 high pathogenicity avian influenza virus genotypes in Korea during the autumn of 2022. Virus particles are represented by ovals containing horizontal bars, which themselves represent the eight gene segments from PB2 to NS, colored according to their origin. The blue horizontal bars denote the eight segments of the H5N1 major cluster isolated (worldwide) since 2020. The yellow horizontal bars denote the eight segments of Eurasian LPAI. The green horizontal bars denote the eight segments of North American LPAI.

By contrast, CT/WA537 has four genes (PA, HA, NA, and M) that are similar to those of the current major H5N1 genotype circulating in Eurasia, despite having four genes (PB2, PB1, NP, and NS) that differ from LPAI isolated in North America (Figure 3). CT/WA537 is homologous (99.3–99.8%) to a virus first detected in April 2022 in North Dakota (Figure 2). It is worth noting that this HPAI virus likely arrived in Korea through international bird migration, presumably via Alaska. This suggests that there is a need to share information and increase surveillance of migratory flyways related to international bird migration between Eurasia and North America.

In summary, we identified H5N1 viruses of at least two genotypes that were introduced into Korea this autumn, seemingly from the Eurasian breeding grounds and North America. Thus, multiple genotypes of 2.3.4.4b H5N1 viruses were introduced into Korea during the autumn season of 2022. These novel H5N1 clade 2.3.4.4b HPAI viruses were detected in wild birds and domestic poultry, suggesting the importance of rapid sharing of surveillance data and increased monitoring of wild birds to provide early warning of possible outbreaks in poultry.

## Figures and Tables

**Table 1:** Blast search of the GISAID database for the nucleotide sequences of three initial strains.

Virus	Gene	Most homologous strains	Nucleotide identity (%)	Accession number
A/mandarin duck/Korea/WA496/2022 and (H5N1) A/duck/Korea/H493/2022 (H5N1)	PB2	A/goose/Hunan/SE284/2022 (H5N1)	99.2–99.5	EPI2029895
		A/duck/Korea/H125/2022 (H5N1)		EPI2205112
	PB1	A/goose/Hunan/SE284/2022 (H5N1)	99.4–99.6	EPI2029896
		A/duck/Korea/H125/2022 (H5N1)		EPI2205111
	PA	A/cygnus columbianus/Hubei/121/2021 (H5N1)	99.2–99.7	EPI2008864
		A/duck/Korea/H125/2022 (H5N1)		EPI2205110
	HA	A/duck/Korea/H125/2022 (H5N1)	99.5–99.7	EPI2231398
		A/goose/Hunan/SE284/2022 (H5N1)		EPI2029898
	NP	A/duck/Korea/H125/2022 (H5N1)	99.4–99.6	EPI2081120
		A/goose/Hunan/SE284/2022 (H5N1)		EPI2081120
	NA	A/goose/Hunan/SE284/2022 (H5N1)	99.2–99.4	EPI2029897
		A/duck/Korea/H125/2022 (H5N1)		EPI2205107
	MP	A/duck/Korea/H125/2022 (H5N1)	99.4–99.7	EPI2205106
		A/swan/Romania/10986/22VIR2749-8/2022 (H5N1)		EPI2015036
	NS	A/goose/Hunan/SE284/2022 (H5N1)	99.3–99.5	EPI2029892
		A/duck/Korea/H125/2022 (H5N1)		EPI2205105

A/common teal/Korea/WA537/2022 (H5N1)	PB2	A/skunk/Washington/22-019274-001-original/2022 (H5N1)	99.7	EPI2182132
	PB1	A/Turkey/Minnesota/22-010928-002-original/2022 (H5N1)	99.7	EPI2179586
	PA	A/Turkey/South Dakota/22-010139-002-original/2022 (H5N1)	99.6	EPI2074664
	HA	A/skunk/Washington/22-019274-001-original/2022 (H5N1)	99.6	EPI2182135
	NP	A/skunk/Washington/22-019274-001-original/2022 (H5N1)	99.7	EPI2182128
	NA	A/skunk/Washington/22-019274-001-original/2022 (H5N1)	99.8	EPI2182134
	MP	A/lesser snow goose/North Dakota/ND-10/2022 (H5N1)	99.8	EPI2188285
	NS	A/skunk/Washington/22-019274-001-original/2022 (H5N1)	99.6	EPI2182129

**Table 2:** Estimated tMRCA of G10-like viruses.

Gene segment and node	rMRCA^a^	95% HPD^b^		Posterior
		Beginning	End	
PB2	Mar. 2021	May 2019	Aug. 2021	1
PB1	Aug. 2021	May 2021	Nov. 2021	0.99
PA	Sep. 2021	Jul. 2021	Nov. 2021	0.92
HA	Sep. 2021	Jul. 2021	Oct. 2021	1
NP	Sep. 2021	Jun. 2021	Nov. 2021	0.97
NA	Feb. 2021	Feb. 2020	Sep. 2021	1
MP	Aug. 2021	May 2021	Nov. 2021	0.97
NS	May 2021	Sep. 2021	Jan 2022	0.99

Note: ^a^Time to most recent common ancestor. ^b^Highest posterior density.

## Data Availability

The data that support the findings of this study are available in GISAID: https://platform.epicov.org/epi3/cfrontend.

## Supplementary Materials

Supplementary Materials Supplementary Table S1: List of viruses from the GISAID EPIFLU on which this research is based (Table 2). All available AIV sequences (approximately 3,200) were obtained from the GISAID EpiFlu database (https://www.gisaid.org) on 17 October 2022. Initially, RAxML was used to construct the phylogenetic trees using all eight genes of the H5NX strains collected after 2020, as well as sequences that were genetically close to MD/WA496, CT/WA537, and BD/H493 (Supplementary Table S1): Among these, representative sequences (50–60) from one cluster of the phylogenetic tree were selected after considering genetic homology, collection date, and geographical location. Phylogenetic trees were generated using the final datasets, and included reference strains detected in the past in Korea. Supplementary Table S2. List of reference H5NX isolates used for tMRCA. To identify the tMRCA of G10-like H5NX HPAI, representative H5NX AI viruses and the internal genes of other subtypes were selected based on genetic homology, geographical location, and collection date (Supplement Table S2): Complete coding sequences of each gene segment were used for comparative phylogenetic analyses. Multiple sequence alignment (PB2: 2280 bp, PB1: 2274 bp, PA: 2151 bp, HA: 1704 bp, NP: 1497 bp, NA: 1413 bp, M: 982 bp, and NS: 838 bp) was performed using MAFFT (https://phylo.org). Supplementary Table S3: Initial three cases and AI surveillance system of HPAI in 2022/2023. Maximum-likelihood phylogenetic trees for the PB2 gene (Figure S1a), PB1 gene (Figure S1b), PA gene (Figure S1c), NP gene (Figure S1d), MP gene (Figure S1e), and NS gene (Figure S1f). The phylogenetic trees are based on H5N1 viruses isolated recently, as well as other H5Nx viruses. Bootstrap values (1,000 replicates) >70% are displayed at the branch nodes. A/mandarin duck/Korea/WA496/2022 (H5N1) and A/breeder duck/Korea/H493/2022 (H5N1) are indicated by a blue solid circle. A/common teal/Korea/WA537/2022 (H5N1) is indicated by a red solid triangle. The scale bar indicates the number of nucleotide substitutions per site.
