# Supplementary material for: Introduction of Multiple Novel High Pathogenicity Avian Influenza (H5N1) Virus of Clade 2.3.4.4b into South Korea in 2022
Source: Transbound Emerg Dis. 2023 Apr 13;2023:8339427. doi: 10.1155/2023/8339427 (PMC12017251; doi:10.1155/2023/8339427)

Supplementary Figure 1(a) Maximum-likelihood tree of PB2

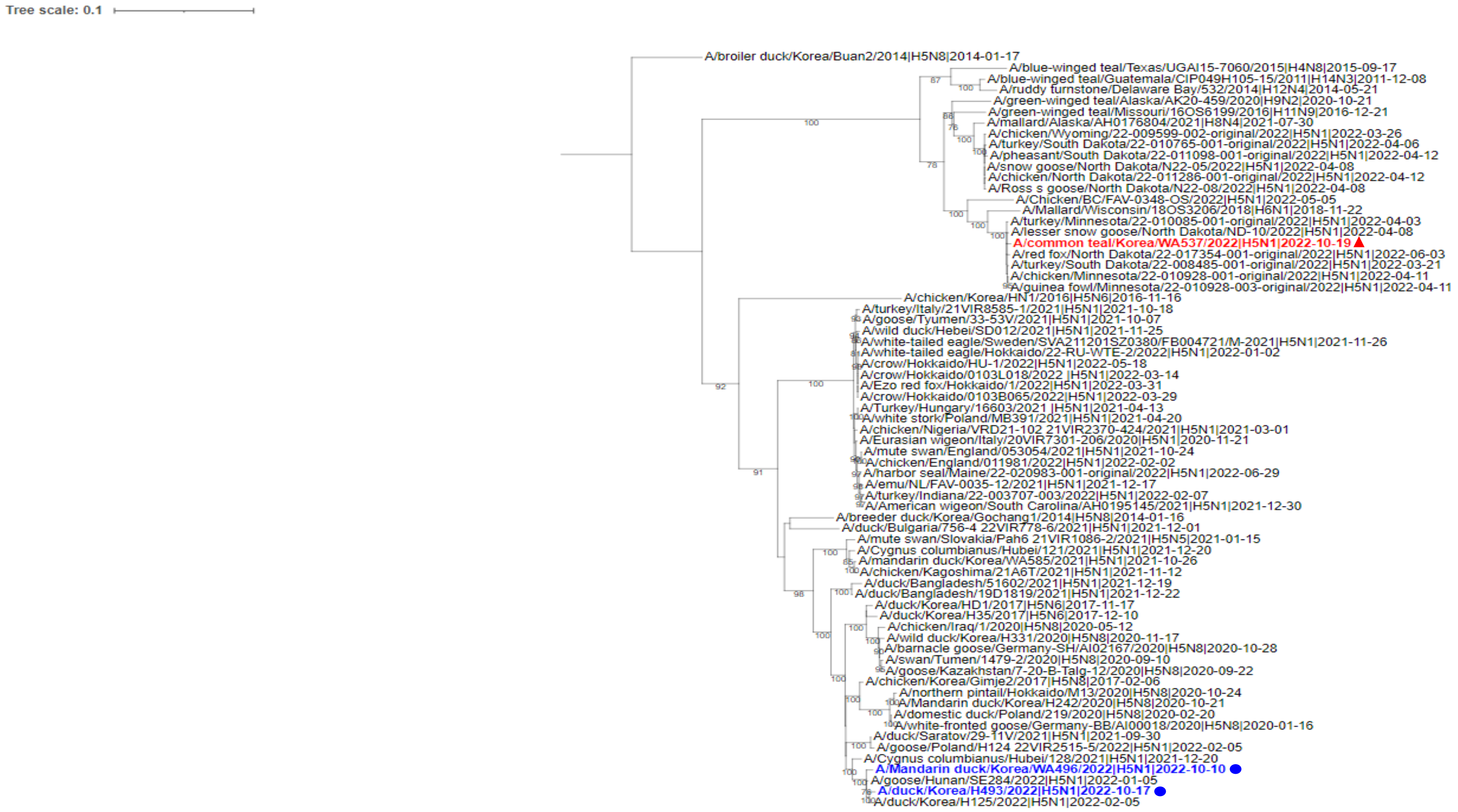

Supplementary Figure 1(b) Maximum-likelihood tree of PB1

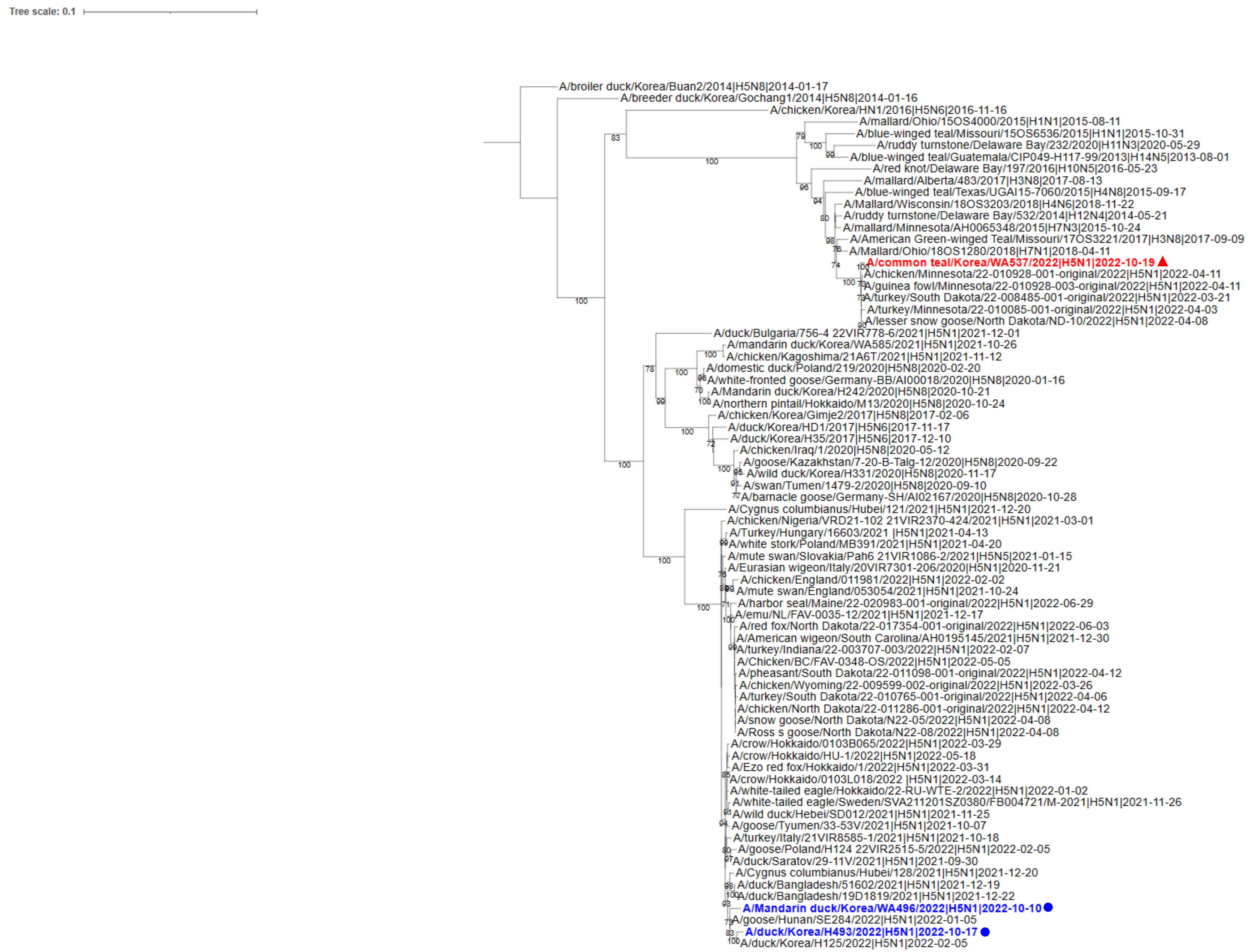

Supplementary Figure 1(c) Maximum-likelihood tree of PA

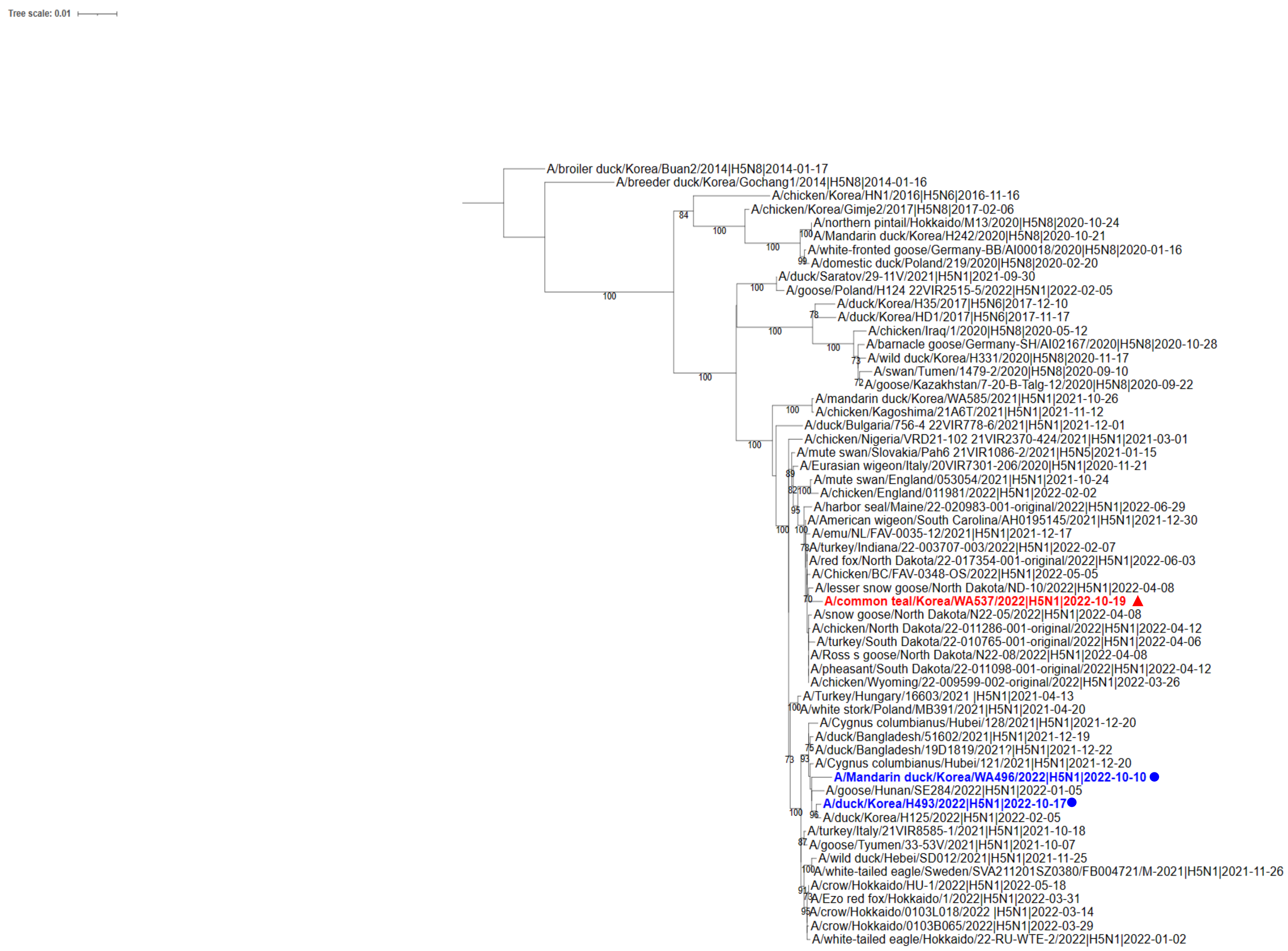

Supplementary Figure 1(d) Maximum-likelihood tree of NP

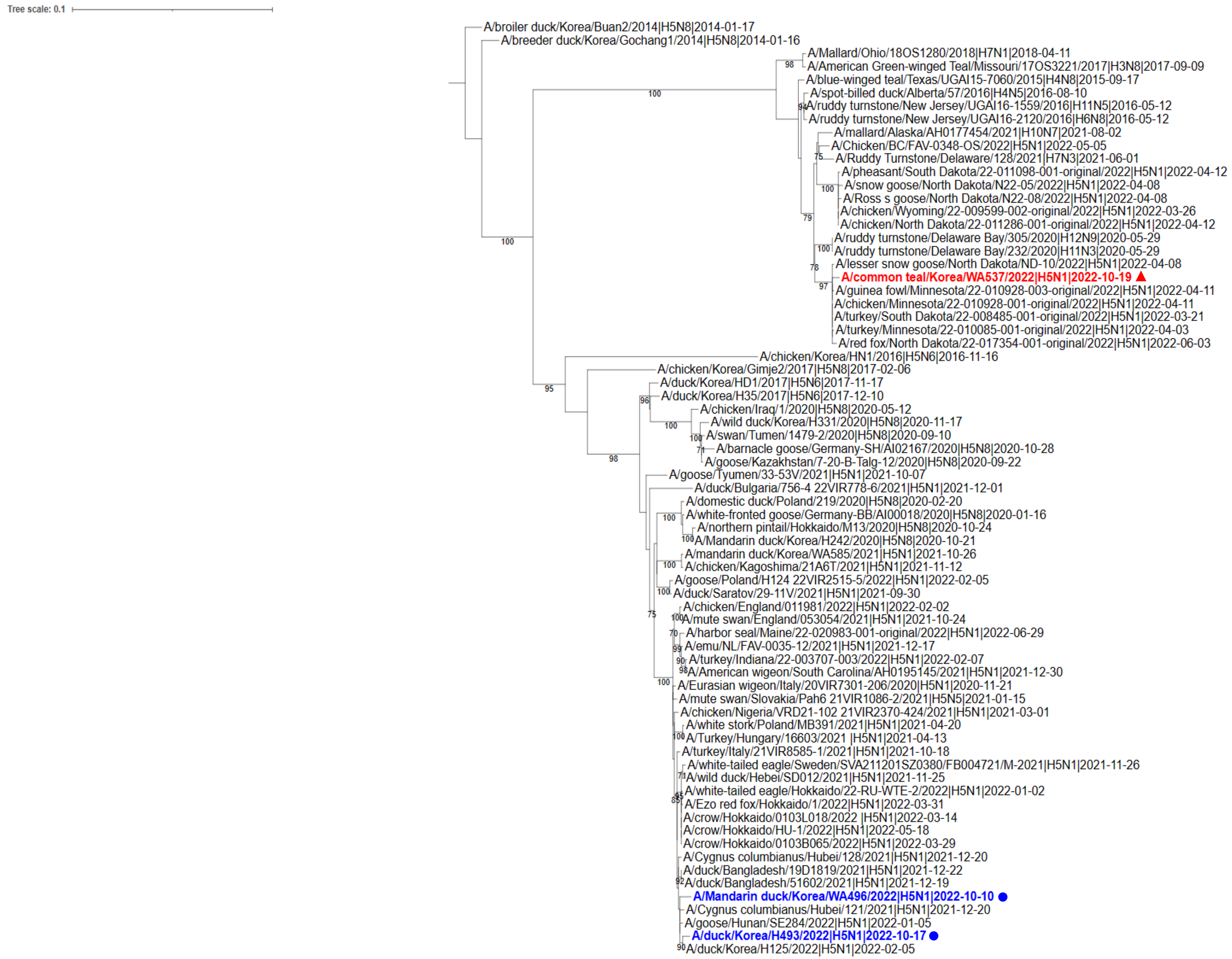

Supplementary Figure 1(e) Maximum-likelihood tree of MP

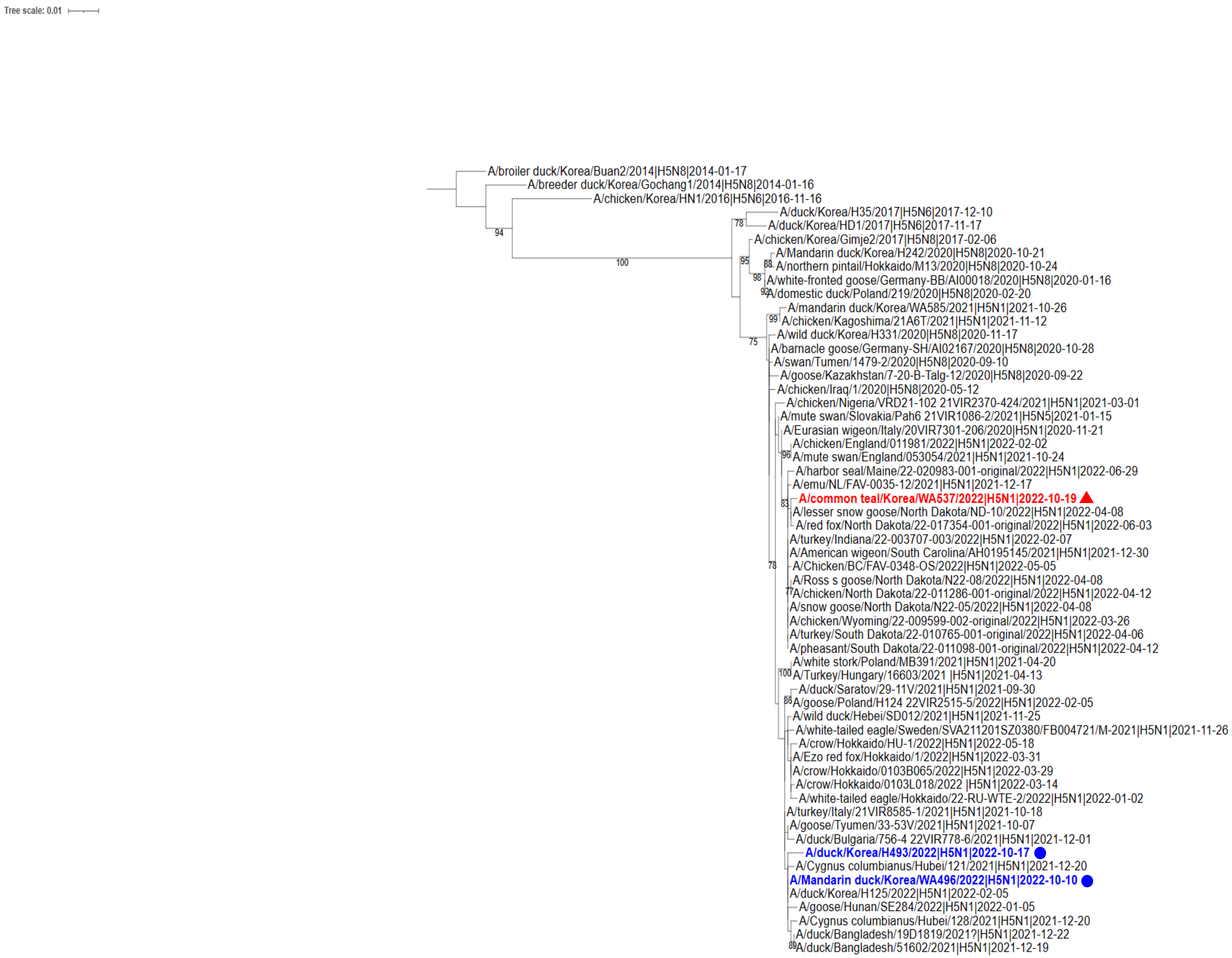

Supplementary Figure 1(f) Maximum-likelihood tree of NS

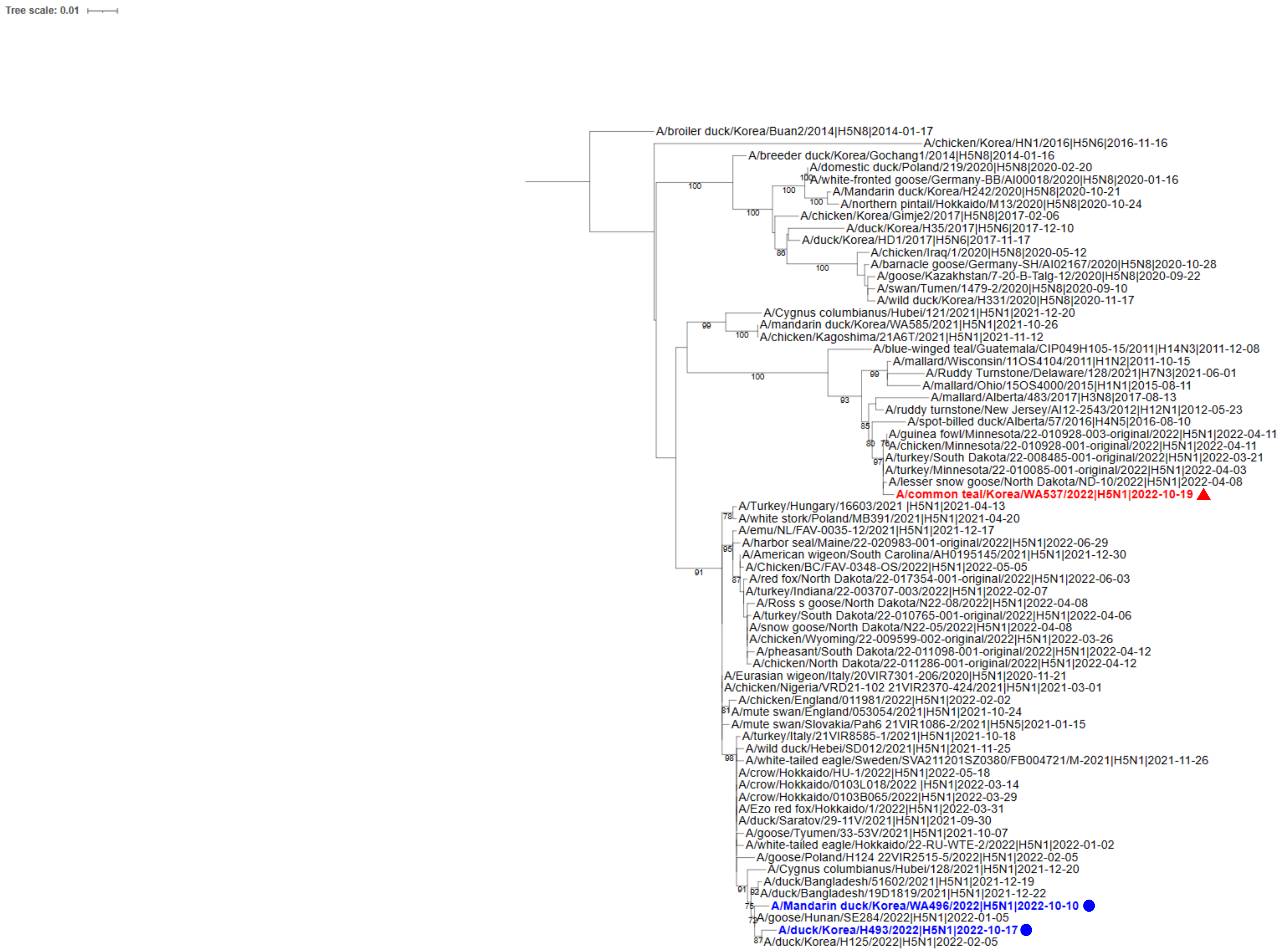

Supplement: Supplementary Materials — Supplementary Table S1: List of viruses from the GISAID EPIFLU on which this research is based (Table 2). All available AIV sequences (approximately 3,200) were obtained from the GISAID EpiFlu database (https://www.gisaid.org) on 17 October 2022. Initially, RAxML was used to construct the phylogenetic trees using all eight genes of the H5NX strains collected after 2020, as well as sequences that were genetically close to MD/WA496, CT/WA537, and BD/H493 (Supplementary Table S1): Among these, representative sequences (50–60) from one cluster of the phylogenetic tree were selected after considering genetic homology, collection date, and geographical location. Phylogenetic trees were generated using the final datasets, and included reference strains detected in the past in Korea. Supplementary Table S2. List of reference H5NX isolates used for tMRCA. To identify the tMRCA of G10-like H5NX HPAI, representative H5NX AI viruses and the internal genes of other subtypes were selected based on genetic homology, geographical location, and collection date (Supplement Table S2): Complete coding sequences of each gene segment were used for comparative phylogenetic analyses. Multiple sequence alignment (PB2: 2280 bp, PB1: 2274 bp, PA: 2151 bp, HA: 1704 bp, NP: 1497 bp, NA: 1413 bp, M: 982 bp, and NS: 838 bp) was performed using MAFFT (https://phylo.org). Supplementary Table S3: Initial three cases and AI surveillance system of HPAI in 2022/2023. Maximum-likelihood phylogenetic trees for the PB2 gene (Figure S1a), PB1 gene (Figure S1b), PA gene (Figure S1c), NP gene (Figure S1d), MP gene (Figure S1e), and NS gene (Figure S1f). The phylogenetic trees are based on H5N1 viruses isolated recently, as well as other H5Nx viruses. Bootstrap values (1,000 replicates) >70% are displayed at the branch nodes. A/mandarin duck/Korea/WA496/2022 (H5N1) and A/breeder duck/Korea/H493/2022 (H5N1) are indicated by a blue solid circle. A/common teal/Korea/WA537/2022 (H5N1) is indicated by a red solid [file 8339427.f1.zip › Supplementary figure1 (1).pdf]
