# Supplementary material for: Introduction of Multiple Novel High Pathogenicity Avian Influenza (H5N1) Virus of Clade 2.3.4.4b into South Korea in 2022
Source: Transbound Emerg Dis. 2023 Apr 13;2023:8339427. doi: 10.1155/2023/8339427 (PMC12017251; doi:10.1155/2023/8339427)
Supplement: Supplementary Materials — Supplementary Table S1: List of viruses from the GISAID EPIFLU on which this research is based (Table 2). All available AIV sequences (approximately 3,200) were obtained from the GISAID EpiFlu database (https://www.gisaid.org) on 17 October 2022. Initially, RAxML was used to construct the phylogenetic trees using all eight genes of the H5NX strains collected after 2020, as well as sequences that were genetically close to MD/WA496, CT/WA537, and BD/H493 (Supplementary Table S1): Among these, representative sequences (50–60) from one cluster of the phylogenetic tree were selected after considering genetic homology, collection date, and geographical location. Phylogenetic trees were generated using the final datasets, and included reference strains detected in the past in Korea. Supplementary Table S2. List of reference H5NX isolates used for tMRCA. To identify the tMRCA of G10-like H5NX HPAI, representative H5NX AI viruses and the internal genes of other subtypes were selected based on genetic homology, geographical location, and collection date (Supplement Table S2): Complete coding sequences of each gene segment were used for comparative phylogenetic analyses. Multiple sequence alignment (PB2: 2280 bp, PB1: 2274 bp, PA: 2151 bp, HA: 1704 bp, NP: 1497 bp, NA: 1413 bp, M: 982 bp, and NS: 838 bp) was performed using MAFFT (https://phylo.org). Supplementary Table S3: Initial three cases and AI surveillance system of HPAI in 2022/2023. Maximum-likelihood phylogenetic trees for the PB2 gene (Figure S1a), PB1 gene (Figure S1b), PA gene (Figure S1c), NP gene (Figure S1d), MP gene (Figure S1e), and NS gene (Figure S1f). The phylogenetic trees are based on H5N1 viruses isolated recently, as well as other H5Nx viruses. Bootstrap values (1,000 replicates) >70% are displayed at the branch nodes. A/mandarin duck/Korea/WA496/2022 (H5N1) and A/breeder duck/Korea/H493/2022 (H5N1) are indicated by a blue solid circle. A/common teal/Korea/WA537/2022 (H5N1) is indicated by a red solid [file 8339427.f1.zip › Supplementary table 3.pdf]

Supplementary Table S3. Initial three cases and AI surveillance system of HPAI in 2022/2023[1)]

Date	Location	Animals (Species)	Isolated virus	Clinical sign	Antigen	Antibody
					No. positive/ No. sample	No. positive/ No. sample
10th, Oct.	Choenan	Captured wild birds[2)] (Mandarin duck)	A/mandarin duck/Korea/WA496/2022	No	1/18	2/18
17th, Oct	Yechoen	Poultry[3)] (Breeder duck)	A/duck/Korea/H493/2022	No[4)]	11 house/ 11 house	4/217
19th, Oct	Gimhae	Captured wild birds (Common teal)	A/common teal/Korea/WA537/2022	No	1/1	0/1

1) HPAI H5N1 is still being detected in wild birds (160 cases) and poultry (63 cases) as of January 31, 2023.

2) AI surveillance in wild birds have been conducted by Ministry of Agriculture, Food and Rural Affairs (MAFRA) and Ministry of Environment (ME)

- In winter season 2022/2023, MAFRA detected the first case of HPAI H5N1 in a captured mandarin duck through AI active surveillance. AI Active Surveillance in wild birds is conducted annually by MAFRA including 1,200 captured birds and over 40,000 fecal samples. OP and CL swabs from captured birds are tested individually to diagnose AI, while fecal samples are pooled in five feces. When the pooled fecal samples are positive for the M gene, each sample is tested individually.
- ME also have conducted AI surveillance from fecal samples and captured birds, as well as from dead birds, to detect AIV. Dead birds have being tested only by ME, and HPAI H5N1 has been continuously detected in dead wild birds since October 2022.

3) AI active surveillance in poultry has been conducted regularly once or twice a month depending on the type of poultry and AI passive surveillance in them has been done in response to report of farmer observing clinical signs. For AI active and passive surveillance in poultry, 20 swab samples and 20 feces samples should be collected by house, as well as any dead birds (if applicable). As a result of AI active surveillance, first HPAI H5N1 virus in poultry was detected in breeder duck farm in Yecheon on 17th Oct.

4) No clinical signs were observed in the first HPAI outbreak breeder duck farm in Yecheon. However, clinical signs, such as egg drop, anorexia and mortality, were observed in subsequently some cases of HPAI cases in breeder duck farms (4 farms out of 8 HPAI outbreak breeder duck farms, 50%).

- Mortality was observed in HPAI infected broiler duck (young age) farms (16 farms out of 24 HPAI outbreak broiler duck farms, 66.7%).
