# Supplementary material for: Introduction of Multiple Novel High Pathogenicity Avian Influenza (H5N1) Virus of Clade 2.3.4.4b into South Korea in 2022
Source: Transbound Emerg Dis. 2023 Apr 13;2023:8339427. doi: 10.1155/2023/8339427 (PMC12017251; doi:10.1155/2023/8339427)
Supplement: Supplementary Materials — Supplementary Table S1: List of viruses from the GISAID EPIFLU on which this research is based (Table 2). All available AIV sequences (approximately 3,200) were obtained from the GISAID EpiFlu database (https://www.gisaid.org) on 17 October 2022. Initially, RAxML was used to construct the phylogenetic trees using all eight genes of the H5NX strains collected after 2020, as well as sequences that were genetically close to MD/WA496, CT/WA537, and BD/H493 (Supplementary Table S1): Among these, representative sequences (50–60) from one cluster of the phylogenetic tree were selected after considering genetic homology, collection date, and geographical location. Phylogenetic trees were generated using the final datasets, and included reference strains detected in the past in Korea. Supplementary Table S2. List of reference H5NX isolates used for tMRCA. To identify the tMRCA of G10-like H5NX HPAI, representative H5NX AI viruses and the internal genes of other subtypes were selected based on genetic homology, geographical location, and collection date (Supplement Table S2): Complete coding sequences of each gene segment were used for comparative phylogenetic analyses. Multiple sequence alignment (PB2: 2280 bp, PB1: 2274 bp, PA: 2151 bp, HA: 1704 bp, NP: 1497 bp, NA: 1413 bp, M: 982 bp, and NS: 838 bp) was performed using MAFFT (https://phylo.org). Supplementary Table S3: Initial three cases and AI surveillance system of HPAI in 2022/2023. Maximum-likelihood phylogenetic trees for the PB2 gene (Figure S1a), PB1 gene (Figure S1b), PA gene (Figure S1c), NP gene (Figure S1d), MP gene (Figure S1e), and NS gene (Figure S1f). The phylogenetic trees are based on H5N1 viruses isolated recently, as well as other H5Nx viruses. Bootstrap values (1,000 replicates) >70% are displayed at the branch nodes. A/mandarin duck/Korea/WA496/2022 (H5N1) and A/breeder duck/Korea/H493/2022 (H5N1) are indicated by a blue solid circle. A/common teal/Korea/WA537/2022 (H5N1) is indicated by a red solid [file 8339427.f1.zip › Supplementary Table1 (2).pdf]

Isolate_Id	Isolate_Name	Subtype	Location	Host	Isolate_Submitt	Submitting_Lab	Authors	Public	Originating_I	Collection_Date
EPI_ISL_10411	A/peregrine falcon/Tochigi/090205T/2021	A / H5N8	Asia / Japan / T	Falcon	Takehiko Saito	National Institut			National Inst	2021-02-15
EPI_ISL_10411	A/peregrine falcon/Tochigi/090205C/2021	A / H5N8	Asia / Japan / T	Falcon	Takehiko Saito	National Institut			National Inst	2021-02-15
EPI_ISL_10411	A/eastern buzzard/Toyama/160213T/2021	A / H5N8	Asia / Japan / T	Falcon	Takehiko Saito	National Institut			National Inst	2021-02-10
EPI_ISL_10411	A/eastern buzzard/Toyama/160213C/2021	A / H5N8	Asia / Japan / T	Falcon	Takehiko Saito	National Institut			National Inst	2021-02-10
EPI_ISL_11391	A/peregrine falcon/Netherlands/20018821-002/	A / H5N8	Europe / Nethe	Falco pereg	Rene Heutink ('	Wageningen Bi	Beerens, Nar		Wageningen	2020-11-25
EPI_ISL_11391	A/peregrine falcon/Netherlands/20017773-002/	A / H5N8	Europe / Nethe	Falco pereg	Rene Heutink ('	Wageningen Bi	Beerens, Nar		Wageningen	2020-11-01
EPI_ISL_98469	A/peregrine falcon/Denmark/14596-1/2020	A / H5N8	Europe / Denm	Falco pereg	Charlotte Kristia	Statens Serum	Yuan Liang, (	Liang	Statens Seru	2020-11-06
EPI_ISL_21725	A/common kestrel/Netherlands/21021301-039/	A / H5N8	Europe / Nethe	Falco tinnu	Rene Heutink ('	Wageningen Bi	Beerens, Nar		Wageningen	2021-01-08
EPI_ISL_11390	A/common kestrel/Netherlands/20017381-001/	A / H5N8	Europe / Nethe	Falco tinnu	Rene Heutink ('	Wageningen Bi	Beerens, Nar		Wageningen	2020-11-05
EPI_ISL_12150	A/goose/Czech Republic/25322-205/2021	A / H5N1	Europe / Czech	Goose	Alexander Nagy	State Veterinar	Alexander,Na		State Veterir	2021-12-18
EPI_ISL_12150	A/goose/Czech Republic/25322-179/2021	A / H5N1	Europe / Czech	Goose	Alexander Nagy	State Veterinar	Alexander,Na		State Veterir	2021-12-18
EPI_ISL_12150	A/goose/Czech Republic/913/2022	A / H5N1	Europe / Czech	Goose	Alexander Nagy	State Veterinar	Alexander,Na		State Veterir	2022-01-12
EPI_ISL_11208	A/goose/Guangdong/S4751/2021(H5N6)	A / H5N6	Asia / China / G	Goose	Pengfei Cui (Ha	Harbin Veterina	Pengfei Cui, (		Harbin Veter	2021-12-08
EPI_ISL_77336	A/goose/Italy/IZSLT-21VIR10273/2021	A / H5N1	Europe / Italy /	Goose	Bianca Zecchin	Istituto Zooprof	Zecchin, B.; F		Istituto Zoopi	2021-11-22
EPI_ISL_73816	A/Goose/Guangdong/211030-1/2021(H5N8)	A / H5N8	Asia / China / G	Goose	Jiahao Zhang (	South China Ag	Zhang, Jiahao		South China	2021-08
EPI_ISL_73811	A/Goose/Liaoning/21640/2021(H5N8)	A / H5N8	Asia / China / L	Goose	Jiahao Zhang (	South China Ag	Zhang, Jiahao		South China	2021-06
EPI_ISL_73811	A/Goose/Shandong/21369-5/2021(H5N8)	A / H5N8	Asia / China / S	Goose	Jiahao Zhang (	South China Ag	Zhang, Jiahao		South China	2021-03
EPI_ISL_73810	A/Goose/Shandong/21369-4/2021(H5N8)	A / H5N8	Asia / China / S	Goose	Jiahao Zhang (	South China Ag	Zhang, Jiahao		South China	2021-03
EPI_ISL_67591	A/Goose/Sichuan/21765-8/2021(H5N6)	A / H5N6	Asia / China / S	Goose	Jiahao Zhang (	South China Ag	Zhang, Jiahao		South China	2021-06
EPI_ISL_52604	A/goose/Liaoning/S1266/2021	A / H5N8	Asia / China / L	Goose	Pengfei Cui (Ha	Harbin Veterina	Pengfei Cui, (		Harbin Veter	2021-04-13
EPI_ISL_52604	A/goose/Zhejiang/S1266/2021	A / H5N8	Asia / China / Z	Goose	Pengfei Cui (Ha	Harbin Veterina	Pengfei Cui, (		Harbin Veter	2021-03-09
EPI_ISL_52604	A/goose/Jiangsu/S1385/2021	A / H5N8	Asia / China / J	Goose	Pengfei Cui (Ha	Harbin Veterina	Pengfei Cui, (		Harbin Veter	2021-03-24
EPI_ISL_52604	A/goose/Hunan/S11288/2021	A / H5N8	Asia / China / H	Goose	Pengfei Cui (Ha	Harbin Veterina	Pengfei Cui, (		Harbin Veter	2021-03-19
EPI_ISL_52604	A/goose/Henan/S1315/2021	A / H5N8	Asia / China / H	Goose	Pengfei Cui (Ha	Harbin Veterina	Pengfei Cui, (		Harbin Veter	2021-04-22
EPI_ISL_52604	A/goose/Guangxi/S20601/2021	A / H5N8	Asia / China / G	Goose	Pengfei Cui (Ha	Harbin Veterina	Pengfei Cui, (		Harbin Veter	2021-05-11
EPI_ISL_51447	A/domestic goose/Germany-NI/AI02980/2021	A / H5N8	Europe / Germa	Goose	Jacqueline King	Friedrich-Loeffl			Lebensmittel	2021-03-16
EPI_ISL_50966	A/wild goose/Germany-NI/AI00625/2021	A / H5N8	Europe / Germa	Goose	Jacqueline King	Friedrich-Loeffl			Lebensmittel	2021-01-12
EPI_ISL_50961	A/wild goose/Germany-NI/AI00855/2021	A / H5N8	Europe / Germa	Goose	Jacqueline King	Friedrich-Loeffl			Lebensmittel	2021-01-20
EPI_ISL_21725	A/wild goose/Netherlands/21024076-001/2021	A / H5N8	Europe / Nethe	Goose	Rene Heutink ('	Wageningen Bi	Beerens, Nar		Wageningen	2021-01-30
EPI_ISL_11391	A/wild goose/Netherlands/20018822-002/2020	A / H5N8	Europe / Nethe	Goose	Rene Heutink ('	Wageningen Bi	Beerens, Nar		Wageningen	2020-11-25
EPI_ISL_11391	A/wild goose/Netherlands/20018735-002/2020	A / H5N8	Europe / Nethe	Goose	Rene Heutink ('	Wageningen Bi	Beerens, Nar		Wageningen	2020-11-24
EPI_ISL_11391	A/wild goose/Netherlands/20017819-001/2020	A / H5N8	Europe / Nethe	Goose	Rene Heutink ('	Wageningen Bi	Beerens, Nar		Wageningen	2020-11-10
EPI_ISL_11391	A/wild goose/Netherlands/20017816-001/2020	A / H5N8	Europe / Nethe	Goose	Rene Heutink ('	Wageningen Bi	Beerens, Nar		Wageningen	2020-11-11
EPI_ISL_11390	A/wild goose/Netherlands/20017761-002/2020	A / H5N8	Europe / Nethe	Goose	Rene Heutink ('	Wageningen Bi	Beerens, Nar		Wageningen	2020-11-04
EPI_ISL_11390	A/wild goose/Netherlands/20017755-002/2020	A / H5N8	Europe / Nethe	Goose	Rene Heutink ('	Wageningen Bi	Beerens, Nar		Wageningen	2020-11-01
EPI_ISL_11390	A/wild goose/Netherlands/20017495-002/2020	A / H5N8	Europe / Nethe	Goose	Rene Heutink ('	Wageningen Bi	Beerens, Nar		Wageningen	2020-11-09
EPI_ISL_11390	A/wild goose/Netherlands/20016959-001/2020	A / H5N8	Europe / Nethe	Goose	Rene Heutink ('	Wageningen Bi	Beerens, Nar		Wageningen	2020-11-02
EPI_ISL_81114	A/Goose/Hungary/21737/2020	A / H5N8	Europe / Hunga	Goose	Katalin Szentpá	National Food (	Katalin,Szent		National Foo	2020-04-30
EPI_ISL_79601	A/Goose/Hungary/15267/2020	A / H5N8	Europe / Hunga	Goose	Katalin Szentpá	National Food (	Katalin,Szent		National Foo	2020-03-26
EPI_ISL_61507	A/domestic_goose/Kazakhstan/1-242_2-20-B/2	A / H5N8	Asia / Kazakhst	Goose	Alex Byrne (Ani	Animal and Pla			National Vet	2020-09-19
EPI_ISL_61506	A/domestic_goose/Kazakhstan/1-248_2-20-B/2	A / H5N8	Asia / Kazakhst	Goose	Alex Byrne (Ani	Animal and Pla			National Vet	2020-09-20

EPI_ISL_6150(	A/domestic_goose/Kazakhstan/1-261_2-20-B/2	A / H5N8	Asia / Kazakhst	Goose	Alex Byrne (Ani	Animal and Pla			National Vet	2020-09-22
EPI_ISL_5254(	A/domestic_goose/Poland/274/2020	A / H5N8	Europe / Polan	Goose	Edyta Świętoń (	National Veteri	Swieton, E.; S		National Vet	2020-03-01
EPI_ISL_1221	A/greylag goose/Netherlands/22005844-002/2(	A / H5N1	Europe / Nethe	Anser anse	Rene Heutink ('	Wageningen Bi	Beerens, Nar		Wageningen	2022-03-23
EPI_ISL_1941	A/goose/Czech Republic/5363-10/2021	A / H5N8	Europe / Czech	Anser anse	Alexander Nag	State Veterinar	Nagy,A;Cerni		State Veterir	2021-03-13
EPI_ISL_1139	A/greylag goose/Netherlands/20018070-002/2(	A / H5N8	Europe / Nethe	Anser anse	Rene Heutink ('	Wageningen Bi	Beerens, Nar		Wageningen	2020-11-15
EPI_ISL_1139	A/greylag goose/Netherlands/20017058-002/2(	A / H5N8	Europe / Nethe	Anser anse	Rene Heutink ('	Wageningen Bi	Beerens, Nar		Wageningen	2020-10-25
EPI_ISL_1139	A/greylag goose/Netherlands/20017386-001/2(	A / H5N8	Europe / Nethe	Anser anse	Rene Heutink ('	Wageningen Bi	Beerens, Nar		Wageningen	2020-11-05
EPI_ISL_1139	A/greylag goose/Netherlands/20016523-001/2(	A / H5N8	Europe / Nethe	Anser anse	Rene Heutink ('	Wageningen Bi	Beerens, Nar		Wageningen	2020-10-26
EPI_ISL_1139(	A/greylag goose/Netherlands/20016896-001/2(	A / H5N8	Europe / Nethe	Anser anse	Rene Heutink ('	Wageningen Bi	Beerens, Nar		Wageningen	2020-11-02
EPI_ISL_1139(	A/greylag goose/Netherlands/20017256-002/2(	A / H5N8	Europe / Nethe	Anser anse	Rene Heutink ('	Wageningen Bi	Beerens, Nar		Wageningen	2020-11-03
EPI_ISL_1139(	A/greylag goose/Netherlands/20017476-001/2(	A / H5N8	Europe / Nethe	Anser anse	Rene Heutink ('	Wageningen Bi	Beerens, Nar		Wageningen	2020-11-06
EPI_ISL_1139(	A/greylag goose/Netherlands/20017064-002/2(	A / H5N8	Europe / Nethe	Anser anse	Rene Heutink ('	Wageningen Bi	Beerens, Nar		Wageningen	2020-11-03
EPI_ISL_1139(	A/greylag goose/Netherlands/20016877-001/2(	A / H5N8	Europe / Nethe	Anser anse	Rene Heutink ('	Wageningen Bi	Beerens, Nar		Wageningen	2020-10-30
EPI_ISL_1139(	A/greylag goose/Netherlands/20017399-006/2(	A / H5N8	Europe / Nethe	Anser anse	Rene Heutink ('	Wageningen Bi	Beerens, Nar		Wageningen	2020-11-06
EPI_ISL_1139(	A/greylag goose/Netherlands/20016975-004/2(	A / H5N8	Europe / Nethe	Anser anse	Rene Heutink ('	Wageningen Bi	Beerens, Nar		Wageningen	2020-11-02
EPI_ISL_1139(	A/greylag goose/Netherlands/20016756-001/2(	A / H5N8	Europe / Nethe	Anser anse	Rene Heutink ('	Wageningen Bi	Beerens, Nar		Wageningen	2020-10-29
EPI_ISL_1139	A/pink-footed goose/Netherlands/20018068-00	A / H5N8	Europe / Nethe	Anser bracl	Rene Heutink ('	Wageningen Bi	Beerens, Nar		Wageningen	2020-11-14
EPI_ISL_1139(	A/pink-footed goose/Netherlands/20017382-00	A / H5N8	Europe / Nethe	Anser bracl	Rene Heutink ('	Wageningen Bi	Beerens, Nar		Wageningen	2020-11-06
EPI_ISL_9012(	A/guineafowl/Scotland/054471/2021	A / H5N1	Europe / Unitec	Numida me	Alex Byrne (Ani	Animal and Pla			Animal and F	2021-11-01
EPI_ISL_5260	A/brown-headed gull/Tibet/1-1/2021	A / H5N8	Asia / China / T	Gull	Pengfei Cui (Ha	Harbin Veterina	Pengfei Cui, (		Harbin Veter	2021-05-16
EPI_ISL_5260	A/black-backed gull/Shandong/SC189/2021	A / H5N8	Asia / China / S	Gull	Pengfei Cui (Ha	Harbin Veterina	Pengfei Cui, (		Harbin Veter	2021-02-22
EPI_ISL_2713	A/whiskered tern/Hubei/BQ10/2020	A / H5N8	Asia / China / H	Chlidonias	Hongliang Chai	Northeast Fore:			College of W	2020-11-16
EPI_ISL_5062	A/herring gull/Germany-MV/AI02300/2020	A / H5N8	Europe / Germa	Larus arger	Jacqueline King	Friedrich-Loeffl			Landesamt f	2020-11-02
EPI_ISL_1296	A/emu/NL/FAV-0035-12/2021	A / H5N1	North America /	Ostrich	Tamiko Hisana	Canadian Food	Hisanaga,Tar		Canadian Fo	2021-12-17
EPI_ISL_9012	A/pheasant/Wales/385129/2021	A / H5N1	Europe / Unitec	Pheasant	Alex Byrne (Ani	Animal and Pla			Animal and F	2021-10-27
EPI_ISL_1123	A/pheasant/Scotland/000348/2021	A / H5N1	Europe / Unitec	Pheasant	Alex Byrne (Ani	Animal and Pla			Animal and F	2021-02-10
EPI_ISL_1123	A/pheasant/Wales/000252/2021	A / H5N8	Europe / Unitec	Pheasant	Alex Byrne (Ani	Animal and Pla			Animal and F	2021-01-27
EPI_ISL_1697	A/peacock/Czech Republic/6529-2/2021	A / H5N8	Europe / Czech	Pavo crista	Alexander Nag	State Veterinar	Nagy,A;Cerni		State Veterir	2021-04-02
EPI_ISL_4653	A/common pheasant /Sweden/SVA210923SZ0	A / H5N1	Europe / Swed	Phasanius	Siamak Zohari	National Veteri			National Vet	2021-09-22
EPI_ISL_1238	A/common pheasant/Sweden/SVA210224SZ0	A / H5N8	Europe / Swed	Phasanius	Siamak Zohari	National Veteri			National Vet	2021-02-23
EPI_ISL_5260	A/mute swan/Shandong/1/2021	A / H5N8	Asia / China / S	Swan	Pengfei Cui (Ha	Harbin Veterina	Pengfei Cui, (		Harbin Veter	2021-01-14
EPI_ISL_5260	A/black swan/Beijing/1/2021	A / H5N8	Asia / China / B	Swan	Pengfei Cui (Ha	Harbin Veterina	Pengfei Cui, (		Harbin Veter	2021-01-25
EPI_ISL_5145	A/swan/Germany-NI/AI05045/2021	A / H5N8	Europe / Germa	Swan	Jacqueline King	Friedrich-Loeffl			Lebensmittel	2021-07-20
EPI_ISL_1139	A/swan/Netherlands/20018830-004/2020	A / H5N8	Europe / Nethe	Swan	Rene Heutink ('	Wageningen Bi	Beerens, Nar		Wageningen	2020-11-25
EPI_ISL_1139	A/swan/Netherlands/20017772-002/2020	A / H5N1	Europe / Nethe	Swan	Rene Heutink ('	Wageningen Bi	Beerens, Nar		Wageningen	2020-11-10
EPI_ISL_1139	A/swan/Netherlands/20017605-002/2020	A / H5N1	Europe / Nethe	Swan	Rene Heutink ('	Wageningen Bi	Beerens, Nar		Wageningen	2020-11-09
EPI_ISL_1081	A/whooper swan/Tochigi/090203C/2021	A / H5N8	Asia / Japan / T	Swan	Takehiko Saito	National Institut			National Inst	2021-02-14
EPI_ISL_1041	A/whooper swan/Niigata/150212T/2021	A / H5N8	Asia / Japan / N	Swan	Takehiko Saito	National Institut			National Inst	2021-02-13
EPI_ISL_6812	A/swan/Niigata/151118/2020	A / H5N8	Asia / Japan / N	Swan	Takehiko Saito	National Institut			National Inst	2020-11-16
EPI_ISL_6144(	A/mute_swan/Kazakhstan/1-267-20-B/2020	A / H5N8	Asia / Kazakhst	Swan	Alex Byrne (Ani	Animal and Pla	Whittard, Ellic		National Vet	2020-09-23
EPI_ISL_4181	A/Whooper swan/Xinjiang/13/2020	A / H5N6	Asia / China / X	Swan	Yanbing Li (Ha	Harbin Veterina	Yanbing,Li;Mi		Harbin Veter	2020-01-17
EPI_ISL_4181	A/Whooper swan/Xinjiang/12/2020	A / H5N6	Asia / China / X	Swan	Yanbing Li (Ha	Harbin Veterina	Yanbing,Li;Mi		Harbin Veter	2020-01-17

EPI_ISL_41817	A/Whooper swan/Xinjiang/11/2020	A / H5N6	Asia / China / X	Swan	Yanbing Li (Ha	Harbin Veterina	Yanbing,Li;Mi	Harbin Veter	2020-01-17
EPI_ISL_41817	A/Whooper swan/Xinjiang/10/2020	A / H5N6	Asia / China / X	Swan	Yanbing Li (Ha	Harbin Veterina	Yanbing,Li;Mi	Harbin Veter	2020-01-16
EPI_ISL_41817	A/Whooper swan/Xinjiang/9/2020	A / H5N6	Asia / China / X	Swan	Yanbing Li (Ha	Harbin Veterina	Yanbing,Li;Mi	Harbin Veter	2020-01-16
EPI_ISL_41817	A/Whooper swan/Xinjiang/8/2020	A / H5N6	Asia / China / X	Swan	Yanbing Li (Ha	Harbin Veterina	Yanbing,Li;Mi	Harbin Veter	2020-01-16
EPI_ISL_41817	A/Whooper swan/Xinjiang/7/2020	A / H5N6	Asia / China / X	Swan	Yanbing Li (Ha	Harbin Veterina	Yanbing,Li;Mi	Harbin Veter	2020-01-12
EPI_ISL_41817	A/Whooper swan/Xinjiang/6/2020	A / H5N6	Asia / China / X	Swan	Yanbing Li (Ha	Harbin Veterina	Yanbing,Li;Mi	Harbin Veter	2020-01-08
EPI_ISL_41817	A/Mute swan/Xinjiang/5/2020	A / H5N6	Asia / China / X	Swan	Yanbing Li (Ha	Harbin Veterina	Yanbing,Li;Mi	Harbin Veter	2020-01-08
EPI_ISL_41817	A/Mute swan/Xinjiang/4/2020	A / H5N6	Asia / China / X	Swan	Yanbing Li (Ha	Harbin Veterina	Yanbing,Li;Mi	Harbin Veter	2020-01-08
EPI_ISL_41817	A/Whooper swan/Xinjiang/3/2020	A / H5N6	Asia / China / X	Swan	Yanbing Li (Ha	Harbin Veterina	Yanbing,Li;Mi	Harbin Veter	2020-01-04
EPI_ISL_41817	A/Whooper swan/Xinjiang/2/2020	A / H5N6	Asia / China / X	Swan	Yanbing Li (Ha	Harbin Veterina	Yanbing,Li;Mi	Harbin Veter	2020-01-03
EPI_ISL_41816	A/Whooper swan/Xinjiang/1/2020	A / H5N6	Asia / China / X	Swan	Yanbing Li (Ha	Harbin Veterina	Yanbing,Li;Mi	Harbin Veter	2020-01-03
EPI_ISL_11391	A/black swan/Netherlands/20018185-001/2020	A / H5N8	Europe / Nethe	Cygnus atra	Rene Heutink ('	Wageningen Bi	Beerens, Nar	Wageningen	2020-11-16
EPI_ISL_11633	A/Cygnus columbianus/Hubei/128/2021	A / H5N1	Asia / China / H	Cygnus col	Ma Liping (Wul	Wuhan Institute		Wuhan Instit	2021-12-20
EPI_ISL_11633	A/Cygnus columbianus/Hubei/126/2021	A / H5N1	Asia / China / H	Cygnus col	Ma Liping (Wul	Wuhan Institute		Wuhan Instit	2021-12-20
EPI_ISL_11633	A/Cygnus columbianus/Hubei/127/2021	A / H5N1	Asia / China / H	Cygnus col	Ma Liping (Wul	Wuhan Institute		Wuhan Instit	2021-12-20
EPI_ISL_11633	A/Cygnus columbianus/Hubei/123/2021	A / H5N1	Asia / China / H	Cygnus col	Ma Liping (Wul	Wuhan Institute		Wuhan Instit	2021-12-20
EPI_ISL_11633	A/Cygnus columbianus/Hubei/121/2021	A / H5N1	Asia / China / H	Cygnus col	Ma Liping (Wul	Wuhan Institute		Wuhan Instit	2021-12-20
EPI_ISL_11633	A/Cygnus columbianus/Hubei/117/2021	A / H5N1	Asia / China / H	Cygnus col	Ma Liping (Wul	Wuhan Institute		Wuhan Instit	2021-12-20
EPI_ISL_27131	A/tundra swan/Hubei/BQ9/2020	A / H5N8	Asia / China / H	Cygnus col	Hongliang Chai	Northeast Fore		College of W	2020-11-16
EPI_ISL_25561	A/Cygnus columbianus/Hubei/49/2020	A / H5N8	Asia / China / J	Cygnus col	Ma Liping (Wul	Wuhan Institute	Jiason Xiong,	State Key La	2020-11-12
EPI_ISL_12150	A/mute swan/Czech Republic/2755/2022	A / H5N1	Europe / Czech	Cygnus olo	Alexander Nag	State Veterinar		State Veterir	2022-01-29
EPI_ISL_10364	A/mute swan/Czech Republic/4099/2021	A / H5N5	Europe / Czech	Cygnus olo	Alexander Nag	State Veterinar	Alexander,Na	State Veterir	2021-02-24
EPI_ISL_73575	A/mute swan/Croatia/101/2021	A / H5N1	Europe / Croati	Cygnus olo	Vladimir Savić	Croatian Veteri	Savić, Vladim	Croatian Vet	2021-11-12
EPI_ISL_32945	A/Mute Swan/Sweden/SVA210330SZ0417/FB	A / H5N5	Europe / Swed	Cygnus olo	Siamak Zohari	National Veteri		National Vet	2021-03-30
EPI_ISL_21140	A/mute_swan/Poland/MB272/2021	A / H5N8	Europe / Polan	Cygnus olo	Kamila Dziadek	National Veteri	Dziadek, K.;	National Vet	2021-03-06
EPI_ISL_12956	A/mute_swan/Norway/FU48/2021	A / H5N8	Europe / Norwa	Cygnus olo	Alex Byrne (An	Animal and Pla	Britt Gjerset,	Norwegian V	2021-01-26
EPI_ISL_12792	A/mute swan/Croatia/14/2021	A / H5N8	Europe / Croati	Cygnus olo	Vladimir Savić	Croatian Veteri	Savić, Vladim	Croatian Vet	2021-03-01
EPI_ISL_11802	A/mute swan/Czech Republic/1656-1/2021	A / H5N8	Europe / Czech	Cygnus olo	Alexander Nag	State Veterinar	Nagy,A; Cern	State Veterir	2021-01-24
EPI_ISL_11391	A/mute swan/Netherlands/20019137-005/2020	A / H5N8	Europe / Nethe	Cygnus olo	Rene Heutink ('	Wageningen Bi	Beerens, Nar	Wageningen	2020-11-30
EPI_ISL_11391	A/mute swan/Netherlands/20018923-001/2020	A / H5N8	Europe / Nethe	Cygnus olo	Rene Heutink ('	Wageningen Bi	Beerens, Nar	Wageningen	2020-11-26
EPI_ISL_11391	A/mute swan/Netherlands/20018824-002/2020	A / H5N8	Europe / Nethe	Cygnus olo	Rene Heutink ('	Wageningen Bi	Beerens, Nar	Wageningen	2020-11-25
EPI_ISL_11391	A/mute swan/Netherlands/20018738-001/2020	A / H5N8	Europe / Nethe	Cygnus olo	Rene Heutink ('	Wageningen Bi	Beerens, Nar	Wageningen	2020-11-24
EPI_ISL_11391	A/mute swan/Netherlands/20018754-006/2020	A / H5N8	Europe / Nethe	Cygnus olo	Rene Heutink ('	Wageningen Bi	Beerens, Nar	Wageningen	2020-11-24
EPI_ISL_11391	A/mute swan/Netherlands/20018754-004/2020	A / H5N8	Europe / Nethe	Cygnus olo	Rene Heutink ('	Wageningen Bi	Beerens, Nar	Wageningen	2020-11-24
EPI_ISL_1139	A/mute swan/Netherlands/20017717-002/2020	A / H5N8	Europe / Nethe	Cygnus olo	Rene Heutink ('	Wageningen Bi	Beerens, Nar	Wageningen	2020-11-10
EPI_ISL_1139	A/mute swan/Netherlands/20017547-002/2020	A / H5N8	Europe / Nethe	Cygnus olo	Rene Heutink ('	Wageningen Bi	Beerens, Nar	Wageningen	2020-11-10
EPI_ISL_1139	A/mute swan/Netherlands/20017153-002/2020	A / H5N8	Europe / Nethe	Cygnus olo	Rene Heutink ('	Wageningen Bi	Beerens, Nar	Wageningen	2020-11-03
EPI_ISL_1139	A/mute swan/Netherlands/20017061-001/2020	A / H5N8	Europe / Nethe	Cygnus olo	Rene Heutink ('	Wageningen Bi	Beerens, Nar	Wageningen	2020-11-02
EPI_ISL_1139	A/mute swan/Netherlands/20016960-001/2020	A / H5N8	Europe / Nethe	Cygnus olo	Rene Heutink ('	Wageningen Bi	Beerens, Nar	Wageningen	2020-11-02
EPI_ISL_1139	A/mute swan/Netherlands/20017131-006/2020	A / H5N8	Europe / Nethe	Cygnus olo	Rene Heutink ('	Wageningen Bi	Beerens, Nar	Wageningen	2020-11-04
EPI_ISL_1139	A/mute swan/Netherlands/20016973-001/2020	A / H5N8	Europe / Nethe	Cygnus olo	Rene Heutink ('	Wageningen Bi	Beerens, Nar	Wageningen	2020-11-02
EPI_ISL_1139	A/mute swan/Netherlands/20016618-001/2020	A / H5N8	Europe / Nethe	Cygnus olo	Rene Heutink ('	Wageningen Bi	Beerens, Nar	Wageningen	2020-10-28

EPI_ISL_1139(	A/mute swan/Netherlands/20016634-001/2020	A / H5N8	Europe / Nethe	Cygnus olo	Rene Heutink ('	Wageningen Bi	Beerens, Nar	Wageningen	2020-10-28
EPI_ISL_1139(	A/mute swan/Netherlands/20016516-001/2020	A / H5N8	Europe / Nethe	Cygnus olo	Rene Heutink ('	Wageningen Bi	Beerens, Nar	Wageningen	2020-10-27
EPI_ISL_1139(	A/wild bird/Netherlands/20016515-002/2020	A / H5N8	Europe / Nethe	Cygnus olo	Rene Heutink ('	Wageningen Bi	Beerens, Nar	Wageningen	2020-10-26
EPI_ISL_11233	A/mute_swan/England/234135/2020	A / H5N8	Europe / Unitec	Cygnus olo	Alex Byrne (Ani	Animal and Pla		Animal and F	2020-12-01
EPI_ISL_11233	A/mute swan/England/263814/2020	A / H5N8	Europe / Unitec	Cygnus olo	Alex Byrne (Ani	Animal and Pla		Animal and F	2020-11-10
EPI_ISL_11147	A/mute swan/North Ossetia-Alania/325-03/202	A / H5N8	Europe / Russia	Cygnus olo	Natalia Goncha	State Research	Natalia,Goncl	State Resea	2020-12-31
EPI_ISL_11147	A/mute swan/North Ossetia-Alania/325-02/202	A / H5N8	Europe / Russia	Cygnus olo	Natalia Goncha	State Research	Natalia,Goncl	State Resea	2020-12-31
EPI_ISL_11147	A/mute swan/North Ossetia-Alania/325-01/202	A / H5N8	Europe / Russia	Cygnus olo	Natalia Goncha	State Research	Natalia,Goncl	State Resea	2020-12-31
EPI_ISL_1058(	A/mute swan/Czech Republic/2669-2/2021	A / H5N8	Europe / Czech	Cygnus olo	Alexander Nagy	State Veterinar	Nagy,A; Cern	State Veterir	2021-02-04
EPI_ISL_10571	A/mute swan/Czech Republic/2669-1/2021	A / H5N8	Europe / Czech	Cygnus olo	Alexander Nagy	State Veterinar	Nagy,A; Cern	State Veterir	2021-02-04
EPI_ISL_10331	A/mute swan/Czech Republic/2600/2021	A / H5N8	Europe / Czech	Cygnus olo	Alexander Nagy	State Veterinar	Nagy,A; Cern	State Veterir	2021-02-04
EPI_ISL_59107	A/mute swan/Netherlands/20015931-001/2020	A / H5N8	Europe / Nethe	Cygnus olo	Rene Heutink ('	Wageningen Bi	Beerens, Nar	Wageningen	2020-10-17
EPI_ISL_79873	A/turkey/Italy/21VIR9117-2/2021	A / H5N1	Europe / Italy	Turkey	Adelaide Milani	Istituto Zooprof	Milani, A.; Fus	Istituto Zoopr	2021-11-04
EPI_ISL_79873	A/turkey/Italy/21VIR8540-82/2021	A / H5N1	Europe / Italy	Turkey	Adelaide Milani	Istituto Zooprof	Milani, A.; Fus	Istituto Zoopr	2021-10-16
EPI_ISL_79873	A/turkey/Italy/21VIR8480-2/2021	A / H5N1	Europe / Italy	Turkey	Adelaide Milani	Istituto Zooprof	Milani, A.; Fus	Istituto Zoopr	2021-10-11
EPI_ISL_79873	A/turkey/Italy/21VIR8480-1/2021	A / H5N1	Europe / Italy	Turkey	Adelaide Milani	Istituto Zooprof	Milani, A.; Fus	Istituto Zoopr	2021-10-11
EPI_ISL_7733(	A/turkey/Italy/21VIR8817-1/2021	A / H5N1	Europe / Italy /	Turkey	Bianca Zecchin	Istituto Zooprof	Zecchin, B.; F	Istituto Zoopr	2021-10-25
EPI_ISL_7733(	A/turkey/Italy/21VIR9210-1/2021	A / H5N1	Europe / Italy /	Turkey	Bianca Zecchin	Istituto Zooprof	Zecchin, B.; F	Istituto Zoopr	2021-11-05
EPI_ISL_7733(	A/turkey/Italy/21VIR9649-2/2021	A / H5N1	Europe / Italy /	Turkey	Bianca Zecchin	Istituto Zooprof	Zecchin, B.; F	Istituto Zoopr	2021-11-15
EPI_ISL_7733(	A/turkey/Italy/21VIR9768-8/2021	A / H5N1	Europe / Italy /	Turkey	Bianca Zecchin	Istituto Zooprof	Zecchin, B.; F	Istituto Zoopr	2021-11-17
EPI_ISL_7733(	A/turkey/Italy/21VIR9510-1/2021	A / H5N1	Europe / Italy /	Turkey	Bianca Zecchin	Istituto Zooprof	Zecchin, B.; F	Istituto Zoopr	2021-11-11
EPI_ISL_7733(	A/turkey/Italy/21VIR9143-2/2021	A / H5N1	Europe / Italy /	Turkey	Bianca Zecchin	Istituto Zooprof	Zecchin, B.; F	Istituto Zoopr	2021-11-03
EPI_ISL_7733(	A/turkey/Italy/21VIR9512-1/2021	A / H5N1	Europe / Italy /	Turkey	Bianca Zecchin	Istituto Zooprof	Zecchin, B.; F	Istituto Zoopr	2021-11-11
EPI_ISL_7733(	A/turkey/Italy/21VIR9652-2/2021	A / H5N1	Europe / Italy /	Turkey	Bianca Zecchin	Istituto Zooprof	Zecchin, B.; F	Istituto Zoopr	2021-11-15
EPI_ISL_7733(	A/turkey/Italy/21VIR10251/2021	A / H5N1	Europe / Italy /	Turkey	Bianca Zecchin	Istituto Zooprof	Zecchin, B.; F	Istituto Zoopr	2021-11-25
EPI_ISL_7733(	A/turkey/Italy/21VIR9767-3/2021	A / H5N1	Europe / Italy /	Turkey	Bianca Zecchin	Istituto Zooprof	Zecchin, B.; F	Istituto Zoopr	2021-11-17
EPI_ISL_7733(	A/turkey/Italy/21VIR10456/2021	A / H5N1	Europe / Italy /	Turkey	Bianca Zecchin	Istituto Zooprof	Zecchin, B.; F	Istituto Zoopr	2021-12-01
EPI_ISL_77335	A/turkey/Italy/21VIR9816-1/2021	A / H5N1	Europe / Italy /	Turkey	Bianca Zecchin	Istituto Zooprof	Zecchin, B.; F	Istituto Zoopr	2021-11-19
EPI_ISL_77335	A/turkey/Italy/21VIR10340/2021	A / H5N1	Europe / Italy /	Turkey	Bianca Zecchin	Istituto Zooprof	Zecchin, B.; F	Istituto Zoopr	2021-11-29
EPI_ISL_77335	A/turkey/Italy/21VIR9618-7/2021	A / H5N1	Europe / Italy /	Turkey	Bianca Zecchin	Istituto Zooprof	Zecchin, B.; F	Istituto Zoopr	2021-11-18
EPI_ISL_5146	A/turkey/Germany-NI/AI04425/2021	A / H5N1	Europe / Germa	Turkey	Jacqueline King	Friedrich-Loeffl		Lebensmittel	2021-05-03
EPI_ISL_51462	A/turkey/Germany-NI/AI04373/2021	A / H5N1	Europe / Germa	Turkey	Jacqueline King	Friedrich-Loeffl		Lebensmittel	2021-04-27
EPI_ISL_51452	A/turkey/Germany-NI/AI04455/2021	A / H5N1	Europe / Germa	Turkey	Jacqueline King	Friedrich-Loeffl		Lebensmittel	2021-05-05
EPI_ISL_5144	A/turkey/Germany-NI/AI01483/2021	A / H5N8	Europe / Germa	Turkey	Jacqueline King	Friedrich-Loeffl		Lebensmittel	2021-02-20
EPI_ISL_51447	A/turkey/Germany-NI/AI01488/2021	A / H5N8	Europe / Germa	Turkey	Jacqueline King	Friedrich-Loeffl		Lebensmittel	2021-02-21
EPI_ISL_5144(	A/turkey/Germany-NI/AI02393/2021	A / H5N8	Europe / Germa	Turkey	Jacqueline King	Friedrich-Loeffl		Lebensmittel	2021-03-08
EPI_ISL_5137	A/turkey/Germany-NI/AI02674/2021	A / H5N8	Europe / Germa	Turkey	Jacqueline King	Friedrich-Loeffl		Lebensmittel	2021-03-14
EPI_ISL_51373	A/turkey/Germany-NI/AI02678/2021	A / H5N8	Europe / Germa	Turkey	Jacqueline King	Friedrich-Loeffl		Lebensmittel	2021-03-13
EPI_ISL_51368	A/turkey/Germany-NI/AI02671/2021	A / H5N8	Europe / Germa	Turkey	Jacqueline King	Friedrich-Loeffl		Lebensmittel	2021-03-14
EPI_ISL_50972	A/turkey/Germany-NI/AI00621/2021	A / H5N8	Europe / Germa	Turkey	Jacqueline King	Friedrich-Loeffl		Lebensmittel	2021-01-17
EPI_ISL_50961	A/turkey/Germany-NI/AI00647/2021	A / H5N8	Europe / Germa	Turkey	Jacqueline King	Friedrich-Loeffl		Lebensmittel	2021-01-20
EPI_ISL_5095(	A/turkey/Germany-BB/AI01023/2021	A / H5N8	Europe / Germa	Turkey	Jacqueline King	Friedrich-Loeffl		Landeslabor	2021-01-30

EPI_ISL_50956	A/turkey/Germany-MV/AI01015/2021	A / H5N8	Europe / Germa	Turkey	Jacqueline King	Friedrich-Loeffl		Landesamt f	2021-01-28
EPI_ISL_50956	A/turkey/Germany-MV/AI00909/2021	A / H5N8	Europe / Germa	Turkey	Jacqueline King	Friedrich-Loeffl		Landesamt f	2021-01-26
EPI_ISL_50956	A/turkey/Germany-BB/AI00868/2021	A / H5N8	Europe / Germa	Turkey	Jacqueline King	Friedrich-Loeffl		Landeslabor	2021-01-22
EPI_ISL_50955	A/turkey/Germany-NI/AI00589/2021	A / H5N8	Europe / Germa	Turkey	Jacqueline King	Friedrich-Loeffl		Lebensmittel	2021-01-12
EPI_ISL_50645	A/turkey/Germany-NI/AI03584/2020	A / H5N8	Europe / Germa	Turkey	Jacqueline King	Friedrich-Loeffl		Lebensmittel	2020-12-23
EPI_ISL_50642	A/turkey/Germany-NI/AI03453/2020	A / H5N8	Europe / Germa	Turkey	Jacqueline King	Friedrich-Loeffl		Lebensmittel	2020-12-21
EPI_ISL_12956	A/turkey/Norway/FU496/2020	A / H5N8	Europe / Norwa	Turkey	Alex Byrne (Ani	Animal and Pla	Britt Gjerset,		Norwegian V	2020-11-30
EPI_ISL_81114	A/Turkey/Hungary/21753/2020	A / H5N8	Europe / Hunga	Turkey	Katalin Szentpá	National Food (	Katalin,Szent	National Foo	2020-04-30
EPI_ISL_52546	A/turkey/Poland/366/2020	A / H5N8	Europe / Polan	Turkey	Edyta Świętoń (	National Veterin	Swieton, E.; S	National Vet	2020-03-23
EPI_ISL_52544	A/turkey/Poland/182/2020	A / H5N8	Europe / Polan	Turkey	Edyta Świętoń (	National Veterin	Swieton, E.; S	National Vet	2020-02-07
EPI_ISL_52544	A/turkey/Poland/096/2020	A / H5N8	Europe / Polan	Turkey	Edyta Świętoń (	National Veterin	Swieton, E.; S	National Vet	2020-01-28
EPI_ISL_52544	A/turkey/Poland/079/2020	A / H5N8	Europe / Polan	Turkey	Edyta Świętoń (	National Veterin	Swieton, E.; S	National Vet	2020-01-25
EPI_ISL_52544	A/turkey/Poland/027/2020	A / H5N8	Europe / Polan	Turkey	Edyta Świętoń (	National Veterin	Swieton, E.; S	National Vet	2020-01-09
EPI_ISL_41931	A/turkey/Germany-ST/AI00352/2020	A / H5N8	Europe / Germa	Turkey	Jacqueline King	Friedrich-Loeffl		Landesamt f	2020-03-27
EPI_ISL_41922	A/turkey/Hungary/1020_20VIR749-1/2020	A / H5N8	Europe / Hunga	Turkey	Ambra Pastori (	Istituto Zooprof	Bálint,Ádám;N	National Foo	2020-01-09
EPI_ISL_14811	A/northern gannet/Sweden/SVA220623SZ0232	A / H5N1	Europe / Swede	Other avian	Siamak Zohari	National Veterin		National Vet	2022-06-15
EPI_ISL_14811	A/northern gannet/Sweden/SVA220623SZ0232	A / H5N1	Europe / Swede	Other avian	Siamak Zohari	National Veterin		National Vet	2022-06-20
EPI_ISL_12174	A/crow/Hokkaido/0103B065/2022	A / H5N1	Asia / Japan / H	Other avian	Norikazu Isoda	Graduate Scho	Yoshihiro, SA	Graduate Sc	2022-03-29
EPI_ISL_10724	A/seagull/Netherlands/22002274-002/2022	A / H5N1	Europe / Nethe	Other avian	Rene Heutink ('	Wageningen Bi	Beerens, Nar	Wageningen	2022-02-01
EPI_ISL_10347	A/long-eared owl/Germany-NI/AI09037/2021	A / H5N1	Europe / Germa	Other avian	Jacqueline King	Friedrich-Loeffl		Lebensmittel	2021-12-28
EPI_ISL_52604	A/wild goose/Shandong/SC196/2021	A / H5N8	Asia / China / S	Other avian	Pengfei Cui (Ha	Harbin Veterina	Pengfei Cui, (	Harbin Veter	2021-01-23
EPI_ISL_52604	A/wild duck/Jiangsu/SD019/2021	A / H5N8	Asia / China / J	Other avian	Pengfei Cui (Ha	Harbin Veterina	Pengfei Cui, (	Harbin Veter	2021-01-26
EPI_ISL_52604	A/grebe/Ningxia/SD001/2021	A / H5N8	Asia / China / N	Other avian	Pengfei Cui (Ha	Harbin Veterina	Pengfei Cui, (	Harbin Veter	2021-06-15
EPI_ISL_52604	A/grebe/Shaanxi/SD001/2021	A / H5N8	Asia / China / S	Other avian	Pengfei Cui (Ha	Harbin Veterina	Pengfei Cui, (	Harbin Veter	2021-06-05
EPI_ISL_52604	A/grebe/Shandong/SC184/2021	A / H5N8	Asia / China / S	Other avian	Pengfei Cui (Ha	Harbin Veterina	Pengfei Cui, (	Harbin Veter	2021-02-22
EPI_ISL_52604	A/egret/Jiangsu/SD021/2021	A / H5N8	Asia / China / J	Other avian	Pengfei Cui (Ha	Harbin Veterina	Pengfei Cui, (	Harbin Veter	2021-01-26
EPI_ISL_52604	A/common coot/Shandong/SC197/2021	A / H5N8	Asia / China / S	Other avian	Pengfei Cui (Ha	Harbin Veterina	Pengfei Cui, (	Harbin Veter	2021-01-23
EPI_ISL_51457	A/oystercatcher/Germany-NI/AI05047/2021	A / H5N1	Europe / Germa	Other avian	Jacqueline King	Friedrich-Loeffl		Lebensmittel	2021-07-17
EPI_ISL_31385	A/common buzzard/Sweden/SVA210415SZ033	A / H5N8	Europe / Swede	Other avian	Siamak Zohari	National Veterin		National Vet	2021-03-25
EPI_ISL_25740	A/Towny owel/Sweden/SVAU210406SZ0042/K	A / H5N8	Europe / Swede	Other avian	Siamak Zohari	National Veterin		National Vet	2021-04-06
EPI_ISL_22544	A/western jackdaw/SVA210317SZ0321/KN001	A / H5N8	Europe / Swede	Other avian	Siamak Zohari	National Veterin		National Vet	2021-03-16
EPI_ISL_16971	A/australian brushturkey/Czech Republic/5904/	A / H5N8	Europe / Czech	Other avian	Alexander Nagy	State Veterinar	Nagy,A;Cerni	State Veterir	2021-03-25
EPI_ISL_13862	A/western marsh harrier/Sweden/SVA210316S	A / H5N8	Europe / Swede	Other avian	Siamak Zohari	National Veterin		National Vet	2021-03-16
EPI_ISL_12054	A/red knot/Germany-SH/AI03424/2020	A / H5N3	Europe / Germa	Other avian	Jacqueline King	Friedrich-Loeffl		Landeslabor	2020-12-16
EPI_ISL_12054	A/red knot/Germany-SH/AI03421/2020	A / H5N3	Europe / Germa	Other avian	Jacqueline King	Friedrich-Loeffl		Landeslabor	2020-12-14
EPI_ISL_12054	A/red knot/Germany-SH/AI03419/2020	A / H5N3	Europe / Germa	Other avian	Jacqueline King	Friedrich-Loeffl		Landeslabor	2020-12-14
EPI_ISL_11390	A/great egret/Netherlands/20017754-002/2020	A / H5N8	Europe / Nethe	Other avian	Rene Heutink ('	Wageningen Bi	Beerens, Nar	Wageningen	2020-11-01
EPI_ISL_11390	A/northern lapwing/Netherlands/20017255-001	A / H5N8	Europe / Nethe	Other avian	Rene Heutink ('	Wageningen Bi	Beerens, Nar	Wageningen	2020-11-01
EPI_ISL_11390	A/northern lapwing/Netherlands/20017480-001	A / H5N8	Europe / Nethe	Other avian	Rene Heutink ('	Wageningen Bi	Beerens, Nar	Wageningen	2020-11-06
EPI_ISL_11390	A/eurasian oystercatcher/Netherlands/200175	A / H5N8	Europe / Nethe	Other avian	Rene Heutink ('	Wageningen Bi	Beerens, Nar	Wageningen	2020-11-10
EPI_ISL_11390	A/short-eared owl/Netherlands/20016896-017/2	A / H5N8	Europe / Nethe	Other avian	Rene Heutink ('	Wageningen Bi	Beerens, Nar	Wageningen	2020-11-02
EPI_ISL_20815	A/red_fox/England/AVP-M1-21-01/2020	A / H5N8	Europe / Unitec	Canine	Alex Byrne (Ani	Animal and Pla		Animal and F	2020-12-08

EPI_ISL_14533	A/fox/New_York/074441/2022	A / H5N1	North America /	Other mam	Brittany D Cron	Cornell Univers	Diel,Diego G;	Cornell Unive	2022-04-04
EPI_ISL_12174	A/Ezo red fox/Hokkaido/1/2022	A / H5N1	Asia / Japan / H	Other mam	Norikazu Isoda	Graduate Scho	Yoshihiro, SA	Graduate Sc	2022-03-31
EPI_ISL_14615	A/crow/Hokkaido/0101Q056/2022	A / H5N1	Asia / Japan / H	Avian	Norikazu Isoda	Graduate Scho	Yoshihiro, SA	Graduate Sc	2022-02-08
EPI_ISL_14615	A/crow/Hokkaido/0103L018/2022	A / H5N1	Asia / Japan / H	Avian	Norikazu Isoda	Graduate Scho		Graduate Sc	2022-03-14
EPI_ISL_14615	A/crow/Hokkaido/0102L015/2022	A / H5N1	Asia / Japan / H	Avian	Norikazu Isoda	Graduate Scho	Yoshihiro, SA	Graduate Sc	2022-03-02
EPI_ISL_13902	A/Eurasian Curlew/Netherlands/2/2022	A / H5N1	Europe / Nethe	Avian	Sanne Thewes	Erasmus Medic		Erasmus Me	2022-07-03
EPI_ISL_13338	A/Northern gannet/Sweden/SVA220525SZ040	A / H5N1	Europe / Swede	Avian	Siamak Zohari	National Veteri		National Vet	2022-05-25
EPI_ISL_13157	A/duck/Bangladesh/18D1837/2022	A / H5N1	Asia / Banglade	Avian	Mohammad Er	International Ce	Hossain, M.E	International	2022-02-25
EPI_ISL_13026	A/Barnacle goose/Luxembourg/22033922/2022	A / H5N1	Europe / Luxem	Avian	Chantal J. Sno	Luxembourg In	Aurélie Sausy	Laboratoire d	2022-02-04
EPI_ISL_13026	A/Canada goose/Luxembourg/22012198/2022	A / H5N1	Europe / Luxem	Avian	Chantal J. Sno	Luxembourg In	Aurélie Sausy	Laboratoire d	2022-01-17
EPI_ISL_13026	A/Great cormorant/Luxembourg/21243185/202	A / H5N1	Europe / Luxem	Avian	Chantal J. Sno	Luxembourg In	Aurélie Sausy	Laboratoire d	2021-12-14
EPI_ISL_13026	A/Grey heron/Luxembourg/21243177/2021	A / H5N1	Europe / Luxem	Avian	Chantal J. Sno	Luxembourg In	Aurélie Sausy	Laboratoire d	2021-12-14
EPI_ISL_13026	A/Canada goose/Luxembourg/21239614/2021	A / H5N1	Europe / Luxem	Avian	Chantal J. Sno	Luxembourg In	Aurélie Sausy	Laboratoire d	2021-12-10
EPI_ISL_13026	A/Canada goose/Luxembourg/21237199/2021	A / H5N1	Europe / Luxem	Avian	Chantal J. Sno	Luxembourg In	Aurélie Sausy	Laboratoire d	2021-12-09
EPI_ISL_13026	A/Canada goose/Luxembourg/21217776/2021	A / H5N1	Europe / Luxem	Avian	Chantal J. Sno	Luxembourg In	Aurélie Sausy	Laboratoire d	2021-11-24
EPI_ISL_13026	A/Greylag goose/Luxembourg/21217773/2021	A / H5N1	Europe / Luxem	Avian	Chantal J. Sno	Luxembourg In	Aurélie Sausy	Laboratoire d	2021-11-24
EPI_ISL_13009	A/chicken/Wyoming/22-009599-001-original/20	A / H5N1	North America /	Avian	Mary Lea Killia	National Veteri	Chinh,Thanh;	National Vet	2022-03-26
EPI_ISL_13009	A/turkey/North Carolina/22-009583-002-origina	A / H5N1	North America /	Avian	Mary Lea Killia	National Veteri	Chinh,Thanh;	National Vet	2022-03-27
EPI_ISL_13009	A/turkey/North Carolina/22-009583-001-origina	A / H5N1	North America /	Avian	Mary Lea Killia	National Veteri	Chinh,Thanh;	National Vet	2022-03-27
EPI_ISL_13009	A/turkey/Iowa/22-009550-001-original/2022	A / H5N1	North America /	Avian	Mary Lea Killia	National Veteri	Chinh,Thanh;	National Vet	2022-03-29
EPI_ISL_13009	A/turkey/South Dakota/22-009534-001-original	A / H5N1	North America /	Avian	Mary Lea Killia	National Veteri	Chinh,Thanh;	National Vet	2022-03-29
EPI_ISL_13009	A/chicken/Ohio/22-009419-001-original/2022	A / H5N1	North America /	Avian	Mary Lea Killia	National Veteri	Chinh,Thanh;	National Vet	2022-03-26
EPI_ISL_13009	A/guinea fowl/Maine/22-009412-002-original/20	A / H5N1	North America /	Avian	Mary Lea Killia	National Veteri	Chinh,Thanh;	National Vet	2022-03-25
EPI_ISL_13009	A/chicken/Maine/22-009412-001-original/2022	A / H5N1	North America /	Avian	Mary Lea Killia	National Veteri	Chinh,Thanh;	National Vet	2022-03-25
EPI_ISL_13009	A/Sebastopol goose/Massachusetts/22-00937	A / H5N1	North America /	Avian	Mary Lea Killia	National Veteri	Chinh,Thanh;	National Vet	2022-03-25
EPI_ISL_12691	A/chicken/North Dakota/22-009337-002-origina	A / H5N1	North America /	Avian	Mary Lea Killia	National Veteri	Chinh,Thanh;	National Vet	2022-03-28
EPI_ISL_12691	A/domestic goose/North Dakota/22-009337-00	A / H5N1	North America /	Avian	Mary Lea Killia	National Veteri	Chinh,Thanh;	National Vet	2022-03-28
EPI_ISL_12691	A/turkey/North Carolina/22-009332-002-origina	A / H5N1	North America /	Avian	Mary Lea Killia	National Veteri	Chinh,Thanh;	National Vet	2022-03-27
EPI_ISL_12691	A/turkey/North Carolina/22-009332-001-origina	A / H5N1	North America /	Avian	Mary Lea Killia	National Veteri	Chinh,Thanh;	National Vet	2022-03-27
EPI_ISL_12691	A/turkey/South Dakota/22-009330-002-original	A / H5N1	North America /	Avian	Mary Lea Killia	National Veteri	Chinh,Thanh;	National Vet	2022-03-28
EPI_ISL_12691	A/turkey/South Dakota/22-009330-001-original	A / H5N1	North America /	Avian	Mary Lea Killia	National Veteri	Chinh,Thanh;	National Vet	2022-03-28
EPI_ISL_12691	A/turkey/South Dakota/22-009329-002-original	A / H5N1	North America /	Avian	Mary Lea Killia	National Veteri	Chinh,Thanh;	National Vet	2022-03-27
EPI_ISL_12691	A/turkey/South Dakota/22-009329-001-original	A / H5N1	North America /	Avian	Mary Lea Killia	National Veteri	Chinh,Thanh;	National Vet	2022-03-27
EPI_ISL_12690	A/turkey/Minnesota/22-009313-002-original/20	A / H5N1	North America /	Avian	Mary Lea Killia	National Veteri	Chinh,Thanh;	National Vet	2022-03-28
EPI_ISL_12690	A/turkey/South Dakota/22-009327-002-original	A / H5N1	North America /	Avian	Mary Lea Killia	National Veteri	Chinh,Thanh;	National Vet	2022-03-27
EPI_ISL_12690	A/turkey/South Dakota/22-009327-001-original	A / H5N1	North America /	Avian	Mary Lea Killia	National Veteri	Chinh,Thanh;	National Vet	2022-03-27
EPI_ISL_12690	A/chicken/Wyoming/22-009326-002-original/20	A / H5N1	North America /	Avian	Mary Lea Killia	National Veteri	Chinh,Thanh;	National Vet	2022-03-29
EPI_ISL_12690	A/quail/New York/22-009324-009-original/2022	A / H5N1	North America /	Avian	Mary Lea Killia	National Veteri	Chinh,Thanh;	National Vet	2022-03-28
EPI_ISL_12690	A/American Buff goose/New York/22-009324-0	A / H5N1	North America /	Avian	Mary Lea Killia	National Veteri	Chinh,Thanh;	National Vet	2022-03-28
EPI_ISL_12690	A/domestic duck/New York/22-009324-004-orig	A / H5N1	North America /	Avian	Mary Lea Killia	National Veteri	Chinh,Thanh;	National Vet	2022-03-28
EPI_ISL_12690	A/chicken/New York/22-009324-002-original/20	A / H5N1	North America /	Avian	Mary Lea Killia	National Veteri	Chinh,Thanh;	National Vet	2022-03-28
EPI_ISL_12690	A/turkey/Minnesota/22-009314-002-original/20	A / H5N1	North America /	Avian	Mary Lea Killia	National Veteri	Chinh,Thanh;	National Vet	2022-03-28

EPI_ISL_1269(	A/turkey/Minnesota/22-009314-001-original/20:	A / H5N1	North America /	Avian	Mary Lea Killiar	National Veteri	Chinh,Thanh;	National Vet(	2022-03-28
EPI_ISL_1269(	A/turkey/Minnesota/22-009313-001-original/20:	A / H5N1	North America /	Avian	Mary Lea Killiar	National Veteri	Chinh,Thanh;	National Vet(	2022-03-28
EPI_ISL_1269(	A/chicken/Iowa/22-009287-002-original/2022	A / H5N1	North America /	Avian	Mary Lea Killiar	National Veteri	Chinh,Thanh;	National Vet(	2022-03-28
EPI_ISL_1269(	A/chicken/Iowa/22-009287-001-original/2022	A / H5N1	North America /	Avian	Mary Lea Killiar	National Veteri	Chinh,Thanh;	National Vet(	2022-03-28
EPI_ISL_1269(	A/turkey/Iowa/22-009286-002-original/2022	A / H5N1	North America /	Avian	Mary Lea Killiar	National Veteri	Chinh,Thanh;	National Vet(	2022-03-28
EPI_ISL_1269(	A/turkey/Iowa/22-009286-001-original/2022	A / H5N1	North America /	Avian	Mary Lea Killiar	National Veteri	Chinh,Thanh;	National Vet(	2022-03-28
EPI_ISL_1269(	A/turkey/Iowa/22-009227-001-original/2022	A / H5N1	North America /	Avian	Mary Lea Killiar	National Veteri	Chinh,Thanh;	National Vet(	2022-03-27
EPI_ISL_1269(	A/turkey/South Dakota/22-009198-001-original.	A / H5N1	North America /	Avian	Mary Lea Killiar	National Veteri	Chinh,Thanh;	National Vet(	2022-03-26
EPI_ISL_1269(	A/turkey/South Dakota/22-009196-002-original.	A / H5N1	North America /	Avian	Mary Lea Killiar	National Veteri	Chinh,Thanh;	National Vet(	2022-03-26
EPI_ISL_1269(	A/turkey/South Dakota/22-009196-001-original.	A / H5N1	North America /	Avian	Mary Lea Killiar	National Veteri	Chinh,Thanh;	National Vet(	2022-03-26
EPI_ISL_1269(	A/turkey/Minnesota/22-009195-003-original/20:	A / H5N1	North America /	Avian	Mary Lea Killiar	National Veteri	Chinh,Thanh;	National Vet(	2022-03-26
EPI_ISL_1269(	A/turkey/Minnesota/22-009195-002-original/20:	A / H5N1	North America /	Avian	Mary Lea Killiar	National Veteri	Chinh,Thanh;	National Vet(	2022-03-26
EPI_ISL_1269(	A/guinea fowl/South Dakota/22-009194-003-or	A / H5N1	North America /	Avian	Mary Lea Killiar	National Veteri	Chinh,Thanh;	National Vet(	2022-03-25
EPI_ISL_1269(	A/chicken/South Dakota/22-009194-002-origin	A / H5N1	North America /	Avian	Mary Lea Killiar	National Veteri	Chinh,Thanh;	National Vet(	2022-03-25
EPI_ISL_1269(	A/duck/Missouri/22-009192-003-original/2022	A / H5N1	North America /	Avian	Mary Lea Killiar	National Veteri	Chinh,Thanh;	National Vet(	2022-03-25
EPI_ISL_1269(	A/chicken/Missouri/22-009192-002-original/202	A / H5N1	North America /	Avian	Mary Lea Killiar	National Veteri	Chinh,Thanh;	National Vet(	2022-03-25
EPI_ISL_12567	A/chicken/Nebraska/22-009190-002-original/2(	A / H5N1	North America /	Avian	Mary Lea Killiar	National Veteri	Chinh,Thanh;	National Vet(	2022-03-24
EPI_ISL_12567	A/domestic duck/Nebraska/22-009190-001-orig	A / H5N1	North America /	Avian	Mary Lea Killiar	National Veteri	Chinh,Thanh;	National Vet(	2022-03-24
EPI_ISL_12567	A/turkey/Minnesota/22-009182-003-original/20:	A / H5N1	North America /	Avian	Mary Lea Killiar	National Veteri	Chinh,Thanh;	National Vet(	2022-03-25
EPI_ISL_12567	A/turkey/Minnesota/22-009182-001-original/20:	A / H5N1	North America /	Avian	Mary Lea Killiar	National Veteri	Chinh,Thanh;	National Vet(	2022-03-25
EPI_ISL_12567	A/Embden goose/Minnesota/22-009181-003-or	A / H5N1	North America /	Avian	Mary Lea Killiar	National Veteri	Chinh,Thanh;	National Vet(	2022-03-25
EPI_ISL_12567	A/chicken/Minnesota/22-009181-001-original/2	A / H5N1	North America /	Avian	Mary Lea Killiar	National Veteri	Chinh,Thanh;	National Vet(	2022-03-25
EPI_ISL_12567	A/chicken/Iowa/22-009180-002-original/2022	A / H5N1	North America /	Avian	Mary Lea Killiar	National Veteri	Chinh,Thanh;	National Vet(	2022-03-25
EPI_ISL_12567	A/chicken/Iowa/22-009180-001-original/2022	A / H5N1	North America /	Avian	Mary Lea Killiar	National Veteri	Chinh,Thanh;	National Vet(	2022-03-25
EPI_ISL_12567	A/chicken/Maryland/22-008243-001-original/20	A / H5N1	North America /	Avian	Mary Lea Killiar	National Veteri	Chinh,Thanh;	National Vet(	2022-03-17
EPI_ISL_1222:	A/grey heron/Czech Republic/25338-2/2021	A / H5N1	Europe / Czech	Avian	Alexander Nagy	State Veterinar	Alexander,Na	State Veterir	2021-12-18
EPI_ISL_1222:	A/grey heron/Czech Republic/25338-1/2021	A / H5N1	Europe / Czech	Avian	Alexander Nagy	State Veterinar	Alexander,Na	State Veterir	2021-12-18
EPI_ISL_1156(	A/Great black-backed Gull/Netherlands/3/2022	A / H5N1	Europe / Nethe	Avian	Mark Pronk (Er	Erasmus Medic		Erasmus Me	2022-03-15
EPI_ISL_1156(	A/Graylag goose/Netherlands/2/20222	A / H5N1	Europe / Nethe	Avian	Mark Pronk (Er	Erasmus Medic		Erasmus Me	2022-03-06
EPI_ISL_1156(	A/Barnacle Goose/Netherlands/18/2022	A / H5N1	Europe / Nethe	Avian	Mark Pronk (Er	Erasmus Medic		Erasmus Me	2022-03-04
EPI_ISL_1156(	A/Barnacle Goose/Netherlands/17/2022	A / H5N1	Europe / Nethe	Avian	Mark Pronk (Er	Erasmus Medic		Erasmus Me	2022-03-03
EPI_ISL_1156(	A/Barnacle Goose/Netherlands/16/2022	A / H5N1	Europe / Nethe	Avian	Mark Pronk (Er	Erasmus Medic		Erasmus Me	2022-03-03
EPI_ISL_1156(	A/Brent goose/Netherlands/1/2022	A / H5N1	Europe / Nethe	Avian	Mark Pronk (Er	Erasmus Medic		Erasmus Me	2022-03-03
EPI_ISL_10347	A/Barnacle Goose/Netherlands/7/2021	A / H5N1	Europe / Nethe	Avian	Pascal Lexmon	Erasmus Medic		Erasmus Me	2021-12-29
EPI_ISL_10347	A/Barnacle Goose/Netherlands/8/2022	A / H5N1	Europe / Nethe	Avian	Pascal Lexmon	Erasmus Medic		Erasmus Me	2022-01-28
EPI_ISL_10347	A/Barnacle Goose/Netherlands/6/2021	A / H5N1	Europe / Nethe	Avian	Pascal Lexmon	Erasmus Medic		Erasmus Me	2021-12-29
EPI_ISL_98801	A/American wigeon/North Carolina/AH0182954	A / H5N1	North America /	Avian	Mary Lea Killiar	National Veteri	Chinh,Thanh;	USDA-NWR(	2022-01-08
EPI_ISL_98801	A/northern shoveler/North Carolina/AH018291:	A / H5N1	North America /	Avian	Mary Lea Killiar	National Veteri	Chinh,Thanh;	USDA-NWR(	2022-01-08
EPI_ISL_98801	A/gadwall/North Carolina/AH0182894/2022	A / H5N1	North America /	Avian	Mary Lea Killiar	National Veteri	Chinh,Thanh;	USDA-NWR(	2022-01-08
EPI_ISL_9880(	A/northern pintail/North Carolina/AH0182892/2	A / H5N1	North America /	Avian	Mary Lea Killiar	National Veteri	Chinh,Thanh;	USDA-NWR(	2022-01-08
EPI_ISL_9880(	A/mallard/North Carolina/AH0182886/2022	A / H5N1	North America /	Avian	Mary Lea Killiar	National Veteri	Chinh,Thanh;	USDA-NWR(	2022-01-08
EPI_ISL_9880(	A/American wigeon/North Carolina/AH0182517	A / H5N1	North America /	Avian	Mary Lea Killiar	National Veteri	Chinh,Thanh;	USDA-NWR(	2022-01-08

EPI_ISL_90125	A/Mallard/Netherlands/15/2021	A / H5N1	Europe / Nethe	Avian	Pascal Lexmon	Erasmus Medic		Erasmus Me	2021-12-27
EPI_ISL_90125	A/Mallard/Netherlands/14/2021	A / H5N1	Europe / Nethe	Avian	Pascal Lexmon	Erasmus Medic		Erasmus Me	2021-12-27
EPI_ISL_90125	A/Mallard/Netherlands/13/2021	A / H5N1	Europe / Nethe	Avian	Pascal Lexmon	Erasmus Medic		Erasmus Me	2021-12-27
EPI_ISL_90125	A/Mallard/Netherlands/12/2021	A / H5N2	Europe / Nethe	Avian	Pascal Lexmon	Erasmus Medic		Erasmus Me	2021-12-23
EPI_ISL_90125	A/Mallard/Netherlands/11/2021	A / H5N2	Europe / Nethe	Avian	Pascal Lexmon	Erasmus Medic		Erasmus Me	2021-12-19
EPI_ISL_90125	A/Herring Gull/Netherlands/1/2021	A / H5N1	Europe / Nethe	Avian	Pascal Lexmon	Erasmus Medic		Erasmus Me	2021-12-17
EPI_ISL_60293	A/great_skua/Scotland/042505/2021	A / H5N1	Europe / Unitec	Avian	Alex Byrne (Ani	Animal and Pla		Animal and F	2021-07-22
EPI_ISL_10411	A/owl/Tochigi/090204C/2021	A / H5N8	Asia / Japan / T	Avian	Takehiko Saito	National Institut		National Inst	2021-02-16
EPI_ISL_13967	A/chicken/Czech Republic/8028-2/2022	A / H5N1	Europe / Czech	Chicken	Alexander Nagy	State Veterinar	Alexander,Na	State Veterir	2022-04-13
EPI_ISL_13157	A/chicken/Bangladesh/10C463/2022	A / H5N1	Asia / Banglade	Chicken	Mohammad Er	International Ce	Hossain, M.E	International	2022-02-26
EPI_ISL_13157	A/chicken/Bangladesh/18B557/2022	A / H5N1	Asia / Banglade	Chicken	Mohammad Er	International Ce	Hossain, M.E	International	2022-02-25
EPI_ISL_13009	A/chicken/Wyoming/22-009599-002-original/20	A / H5N1	North America /	Chicken	Mary Lea Killiar	National Veterir	Chinh,Thanh;	National Vete	2022-03-26
EPI_ISL_77336	A/chicken/Italy/IZSLT-122448_21VIR9218-1/20	A / H5N1	Europe / Italy /	Chicken	Bianca Zecchin	Istituto Zooprof	Zecchin, B.; F	Istituto Zoop	2021-10-28
EPI_ISL_77336	A/chicken/Italy/21VIR10352/2021	A / H5N1	Europe / Italy /	Chicken	Bianca Zecchin	Istituto Zooprof	Zecchin, B.; F	Istituto Zoop	2021-11-26
EPI_ISL_77336	A/chicken/Italy/21VIR9765-12/2021	A / H5N1	Europe / Italy /	Chicken	Bianca Zecchin	Istituto Zooprof	Zecchin, B.; F	Istituto Zoop	2021-11-17
EPI_ISL_77336	A/chicken/Italy/21VIR9691-2/2021	A / H5N1	Europe / Italy /	Chicken	Bianca Zecchin	Istituto Zooprof	Zecchin, B.; F	Istituto Zoop	2021-11-15
EPI_ISL_77336	A/chicken/Italy/21VIR9133-20/2021	A / H5N1	Europe / Italy /	Chicken	Bianca Zecchin	Istituto Zooprof	Zecchin, B.; F	Istituto Zoop	2021-11-04
EPI_ISL_77336	A/chicken/Italy/21VIR9580-22/2021	A / H5N1	Europe / Italy /	Chicken	Bianca Zecchin	Istituto Zooprof	Zecchin, B.; F	Istituto Zoop	2021-11-15
EPI_ISL_77336	A/chicken/Italy/21VIR10384/2021	A / H5N1	Europe / Italy /	Chicken	Bianca Zecchin	Istituto Zooprof	Zecchin, B.; F	Istituto Zoop	2021-11-30
EPI_ISL_77336	A/chicken/Italy/21VIR9951-25/2021	A / H5N1	Europe / Italy /	Chicken	Bianca Zecchin	Istituto Zooprof	Zecchin, B.; F	Istituto Zoop	2021-11-22
EPI_ISL_77335	A/chicken/Italy/21VIR10389/2021	A / H5N1	Europe / Italy /	Chicken	Bianca Zecchin	Istituto Zooprof	Zecchin, B.; F	Istituto Zoop	2021-11-30
EPI_ISL_77335	A/chicken/Italy/21VIR10239/2021	A / H5N1	Europe / Italy /	Chicken	Bianca Zecchin	Istituto Zooprof	Zecchin, B.; F	Istituto Zoop	2021-11-25
EPI_ISL_77335	A/chicken/Italy/21VIR10388/2021	A / H5N1	Europe / Italy /	Chicken	Bianca Zecchin	Istituto Zooprof	Zecchin, B.; F	Istituto Zoop	2021-11-30
EPI_ISL_73814	A/Chicken/Guangxi/21989-3/2021(H5N8)	A / H5N8	Asia / China / G	Chicken	Jiahao Zhang (	South China Ag	Zhang, Jiahao	South China	2021-07
EPI_ISL_73810	A/Chicken/Liaoning/21346-2/2021(H5N8)	A / H5N8	Asia / China / L	Chicken	Jiahao Zhang (	South China Ag	Zhang, Jiahao	South China	2021-03
EPI_ISL_51462	A/chicken/Germany-NW/AI03705/2021	A / H5N8	Europe / Germa	Chicken	Jacqueline King	Friedrich-Loeffle		Chemisches	2021-03-31
EPI_ISL_51461	A/chicken/Germany-BW/AI03634/2021	A / H5N8	Europe / Germa	Chicken	Jacqueline King	Friedrich-Loeffle		CVUA Karlsr	2021-03-29
EPI_ISL_51459	A/chicken/Germany-BW/AI03554/2021	A / H5N8	Europe / Germa	Chicken	Jacqueline King	Friedrich-Loeffle		CVUA Karlsr	2021-03-24
EPI_ISL_51458	A/chicken/Germany-TH/AI03512/2021	A / H5N8	Europe / Germa	Chicken	Jacqueline King	Friedrich-Loeffle		Thüringer La	2021-03-23
EPI_ISL_51456	A/chicken/Germany-BY/AI02060/2021	A / H5N8	Europe / Germa	Chicken	Jacqueline King	Friedrich-Loeffle		Bayrisches L	2021-03-01
EPI_ISL_50955	A/chicken/Germany-NI/AI00547/2021	A / H5N8	Europe / Germa	Chicken	Jacqueline King	Friedrich-Loeffle		Lebensmittel	2021-01-12
EPI_ISL_50955	A/chicken/Germany-MV/AI00539/2021	A / H5N8	Europe / Germa	Chicken	Jacqueline King	Friedrich-Loeffle		Landesamt fi	2021-01-12
EPI_ISL_11233	A/chicken/England/046491/2020	A / H5N8	Europe / Unitec	Chicken	Alex Byrne (Ani	Animal and Pla		Animal and F	2020-12-28
EPI_ISL_11233	A/chicken/England/045984/2020	A / H5N8	Europe / Unitec	Chicken	Alex Byrne (Ani	Animal and Pla		Animal and F	2020-12-25
EPI_ISL_11233	A/duck/England/043628/2020	A / H5N8	Europe / Unitec	Chicken	Alex Byrne (Ani	Animal and Pla		Animal and F	2020-12-18
EPI_ISL_11233	A/chicken/England/043683/2020	A / H5N8	Europe / Unitec	Chicken	Alex Byrne (Ani	Animal and Pla		Animal and F	2020-12-17
EPI_ISL_11232	A/chicken/Scotland/043405/2020	A / H5N8	Europe / Unitec	Chicken	Alex Byrne (Ani	Animal and Pla		Animal and F	2020-12-16
EPI_ISL_10411	A/chicken/Chiba/K5T/2021	A / H5N8	Asia / Japan / C	Chicken	Takehiko Saito	National Institut		National Inst	2021-02-14
EPI_ISL_10411	A/chicken/Chiba/K5C/2021	A / H5N8	Asia / Japan / C	Chicken	Takehiko Saito	National Institut		National Inst	2021-02-14
EPI_ISL_10411	A/chicken/Chiba/K11T/2021	A / H5N8	Asia / Japan / C	Chicken	Takehiko Saito	National Institut		National Inst	2021-02-14
EPI_ISL_10411	A/chicken/Chiba/K11C/2021	A / H5N8	Asia / Japan / C	Chicken	Takehiko Saito	National Institut		National Inst	2021-02-14
EPI_ISL_10411	A/chicken/Tokushima/B4T/2020	A / H5N8	Asia / Japan / T	Chicken	Takehiko Saito	National Institut		National Inst	2021-02-08

EPI_ISL_10411	A/chicken/Tokushima/B3T/2020	A / H5N8	Asia / Japan / T	Chicken	Takehiko Saito	National Institut			National Inst	2021-02-08
EPI_ISL_10411	A/chicken/Tokushima/B2T/2020	A / H5N8	Asia / Japan / T	Chicken	Takehiko Saito	National Institut			National Inst	2021-02-08
EPI_ISL_10411	A/chicken/Tokushima/B1T/2020	A / H5N8	Asia / Japan / T	Chicken	Takehiko Saito	National Institut			National Inst	2021-02-08
EPI_ISL_10411	A/chicken/Chiba/J6T/2021	A / H5N8	Asia / Japan / C	Chicken	Takehiko Saito	National Institut			National Inst	2021-02-10
EPI_ISL_10411	A/chicken/Chiba/J6C/2021	A / H5N8	Asia / Japan / C	Chicken	Takehiko Saito	National Institut			National Inst	2021-02-10
EPI_ISL_10411	A/chicken/Chiba/J1T/2021	A / H5N8	Asia / Japan / C	Chicken	Takehiko Saito	National Institut			National Inst	2021-02-10
EPI_ISL_10411	A/chicken/Chiba/J1C/2021	A / H5N8	Asia / Japan / C	Chicken	Takehiko Saito	National Institut			National Inst	2021-02-10
EPI_ISL_10411	A/chicken/Chiba/I3T/2021	A / H5N8	Asia / Japan / C	Chicken	Takehiko Saito	National Institut			National Inst	2021-02-10
EPI_ISL_10411	A/chicken/Chiba/I2T/2021	A / H5N8	Asia / Japan / C	Chicken	Takehiko Saito	National Institut			National Inst	2021-02-10
EPI_ISL_10411	A/chicken/Chiba/I1T/2021	A / H5N8	Asia / Japan / C	Chicken	Takehiko Saito	National Institut			National Inst	2021-02-10
EPI_ISL_10411	A/chicken/Chiba/I1C/2021	A / H5N8	Asia / Japan / C	Chicken	Takehiko Saito	National Institut			National Inst	2021-02-10
EPI_ISL_98469	A/chicken/Denmark/14819-6/2020	A / H5N8	Europe / Denm	Chicken	Charlotte Kristia	Statens Serum	Yuan Liang, (	Liang	Statens Seru	2020-11-15
EPI_ISL_81113	A/Chicken/Hungary/20227/2020	A / H5N8	Europe / Hunga	Chicken	Katalin Szentpá	National Food (	Katalin,Szent		National Foo	2020-04-23
EPI_ISL_81113	A/Chicken/Hungary/19776/2020	A / H5N8	Europe / Hunga	Chicken	Katalin Szentpá	National Food (	Katalin,Szent		National Foo	2020-04-21
EPI_ISL_81112	A/Chicken/Hungary/18467/2020	A / H5N8	Europe / Hunga	Chicken	Katalin Szentpá	National Food (	Katalin,Szent		National Foo	2020-04-14
EPI_ISL_73806	A/chicken/Tokushima/4T/2020	A / H5N8	Asia / Japan / T	Chicken	Takehiko Saito	National Institut			National Inst	2020-12-18
EPI_ISL_73805	A/chicken/Tokushima/3T/2020	A / H5N8	Asia / Japan / T	Chicken	Takehiko Saito	National Institut			National Inst	2020-12-18
EPI_ISL_73805	A/chicken/Tokushima/2T/2020	A / H5N8	Asia / Japan / T	Chicken	Takehiko Saito	National Institut			National Inst	2020-12-18
EPI_ISL_73805	A/chicken/Tokushima/1T/20200	A / H5N8	Asia / Japan / T	Chicken	Takehiko Saito	National Institut			National Inst	2020-12-18
EPI_ISL_73805	A/chicken/Miyazaki/H9T/2020	A / H5N8	Asia / Japan / M	Chicken	Takehiko Saito	National Institut			National Inst	2020-12-18
EPI_ISL_73805	A/chicken/Miyazaki/H6T/2020	A / H5N8	Asia / Japan / M	Chicken	Takehiko Saito	National Institut			National Inst	2020-12-18
EPI_ISL_73805	A/chicken/Miyazaki/H3T/2020	A / H5N8	Asia / Japan / M	Chicken	Takehiko Saito	National Institut			National Inst	2020-12-18
EPI_ISL_73805	A/chicken/Miyazaki/H1T/2020	A / H5N8	Asia / Japan / M	Chicken	Takehiko Saito	National Institut			National Inst	2020-12-18
EPI_ISL_73805	A/chicken/Kagawa/L9T/2020	A / H5N8	Asia / Japan / K	Chicken	Takehiko Saito	National Institut			National Inst	2020-12-15
EPI_ISL_73805	A/chicken/Kagawa/L7T/2020	A / H5N8	Asia / Japan / K	Chicken	Takehiko Saito	National Institut			National Inst	2020-12-15
EPI_ISL_73805	A/chicken/Kagawa/L6T/2020	A / H5N8	Asia / Japan / K	Chicken	Takehiko Saito	National Institut			National Inst	2020-12-15
EPI_ISL_73804	A/chicken/Kagawa/L4T/2020	A / H5N8	Asia / Japan / K	Chicken	Takehiko Saito	National Institut			National Inst	2020-12-15
EPI_ISL_68129	A/chicken/Kagawa/E7T/2020	A / H5N8	Asia / Japan / K	Chicken	Takehiko Saito	National Institut			National Inst	2020-11-14
EPI_ISL_68129	A/chicken/Kagawa/E13T/2020	A / H5N8	Asia / Japan / K	Chicken	Takehiko Saito	National Institut			National Inst	2020-11-14
EPI_ISL_68129	A/chicken/Kagawa/D5T/2020	A / H5N8	Asia / Japan / K	Chicken	Takehiko Saito	National Institut			National Inst	2020-11-12
EPI_ISL_68129	A/chicken/Kagawa/D2C/2020	A / H5N8	Asia / Japan / K	Chicken	Takehiko Saito	National Institut			National Inst	2020-11-12
EPI_ISL_68129	A/chicken/Kagawa/C5T/2020	A / H5N8	Asia / Japan / K	Chicken	Takehiko Saito	National Institut			National Inst	2020-11-10
EPI_ISL_68129	A/chicken/Kagawa/C3T/2020	A / H5N8	Asia / Japan / K	Chicken	Takehiko Saito	National Institut			National Inst	2020-11-10
EPI_ISL_68129	A/chicken/Kagawa/C3C/2020	A / H5N8	Asia / Japan / K	Chicken	Takehiko Saito	National Institut			National Inst	2020-11-10
EPI_ISL_68129	A/chicken/Kagawa/B8T/2020	A / H5N8	Asia / Japan / K	Chicken	Takehiko Saito	National Institut			National Inst	2020-11-07
EPI_ISL_68128	A/chicken/Kagawa/B13C/2020	A / H5N8	Asia / Japan / K	Chicken	Takehiko Saito	National Institut			National Inst	2020-11-07
EPI_ISL_68128	A/chicken/Kagawa/B10T/2020	A / H5N8	Asia / Japan / K	Chicken	Takehiko Saito	National Institut			National Inst	2020-11-07
EPI_ISL_68128	A/chicken/Kagawa/7C/2020	A / H5N8	Asia / Japan / K	Chicken	Takehiko Saito	National Institut			National Inst	2020-11-04
EPI_ISL_68128	A/chicken/Kagawa/11C/2020	A / H5N8	Asia / Japan / K	Chicken	Takehiko Saito	National Institut			National Inst	2020-11-04
EPI_ISL_68128	A/chicken/Hyogo/2T/2020	A / H5N8	Asia / Japan / H	Chicken	Takehiko Saito	National Institut			National Inst	2020-11-25
EPI_ISL_68128	A/chicken/Hyogo/1T/2020	A / H5N8	Asia / Japan / H	Chicken	Takehiko Saito	National Institut			National Inst	2020-11-25
EPI_ISL_68128	A/chicken/Fukuoka/T2/2020	A / H5N8	Asia / Japan / F	Chicken	Takehiko Saito	National Institut			National Inst	2020-11-24

EPI_ISL_68128	A/chicken/Fukuoka/T1/2020	A / H5N8	Asia / Japan / F	Chicken	Takehiko Saito	National Institut		National Inst	2020-11-24
EPI_ISL_68127	A/chicken/Kagawa/H8C/2020	A / H5N8	Asia / Japan / K	Chicken	Takehiko Saito	National Institut		National Inst	2020-11-20
EPI_ISL_68127	A/chicken/Kagawa/H2T/2020	A / H5N8	Asia / Japan / K	Chicken	Takehiko Saito	National Institut		National Inst	2020-11-20
EPI_ISL_68127	A/chicken/Kagawa/G4T/2020	A / H5N8	Asia / Japan / K	Chicken	Takehiko Saito	National Institut		National Inst	2020-11-19
EPI_ISL_68127	A/chicken/Kagawa/G12C/2020	A / H5N8	Asia / Japan / K	Chicken	Takehiko Saito	National Institut		National Inst	2020-11-19
EPI_ISL_68127	A/chicken/Kagawa/F3C/2020	A / H5N8	Asia / Japan / K	Chicken	Takehiko Saito	National Institut		National Inst	2020-11-19
EPI_ISL_68127	A/chicken/Kagawa/F2T/2020	A / H5N8	Asia / Japan / K	Chicken	Takehiko Saito	National Institut		National Inst	2020-11-19
EPI_ISL_62307	A/chicken/Iraq/1/2020	A / H5N8	Asia / Iraq / Mu	Chicken	Alex Byrne (Ani	Animal and Pla		Central Vete	2020-05-12
EPI_ISL_52544	A/laying_hen/Poland/095/2020	A / H5N8	Europe / Poland	Chicken	Edyta Świętoń (	National Veterin	Swieton, E.; S	National Vet	2020-01-27
EPI_ISL_52544	A/chicken/Poland/054/2020	A / H5N8	Europe / Poland	Chicken	Edyta Świętoń (	National Veterin	Swieton, E.; S	National Vet	2020-01-17
EPI_ISL_41029	A/chicken/Germany-BW/AI00049/2020	A / H5N8	Europe / Germa	Chicken	Jacqueline King	Friedrich-Loeffl		CVUA Karlsr	2020-02-06
EPI_ISL_14885	A/chicken/Bangladesh/49967/2021	A / H5N1	Asia / Banglade	Gallus gallu			Barman,S.; T		2021-09-18
EPI_ISL_14885	A/chicken/Bangladesh/50597/2021	A / H5N1	Asia / Banglade	Gallus gallu			Barman,S.; T		2021-12-14
EPI_ISL_14885	A/chicken/Bangladesh/49971/2021	A / H5N1	Asia / Banglade	Gallus gallu			Barman,S.; T		2021-09-18
EPI_ISL_12150	A/chicken/Czech Republic/913/2022	A / H5N1	Europe / Czech	Gallus gallu	Alexander Nagy	State Veterinar	Alexander,Na	State Veterir	2022-01-12
EPI_ISL_95943	A/chicken/Egypt/S18182C/2020	A / H5N8	Africa / Egypt	Gallus gallu			El-Shesheny,		2020-04-01
EPI_ISL_95942	A/chicken/Egypt/A19670/2021	A / H5N8	Africa / Egypt	Gallus gallu			El-Shesheny,		2021-02-10
EPI_ISL_91111	A/chicken/Netherlands/22001401-001005/2022	A / H5N1	Europe / Nethe	Gallus gallu	Rene Heutink (W	Wageningen Bi	Beerens, Nar	Wageningen	2022-01-22
EPI_ISL_86509	A/chicken/Netherlands/20016597-026030/2020	A / H5N8	Europe / Nethe	Gallus gallu	Rene Heutink (W	Wageningen Bi	Beerens, Nar	Wageningen	2020-10-28
EPI_ISL_55881	A/chicken/Netherlands/21037233-001/2021	A / H5N1	Europe / Nethe	Gallus gallu	Rene Heutink (W	Wageningen Bi	Beerens, Nar	Wageningen	2021-10-22
EPI_ISL_55881	A/chicken/Netherlands/21037287-006010/2021	A / H5N1	Europe / Nethe	Gallus gallu	Rene Heutink (W	Wageningen Bi	Beerens, Nar	Wageningen	2021-10-25
EPI_ISL_19415	A/chicken/Czech Republic/7100/2021	A / H5N8	Europe / Czech	Gallus gallu	Alexander Nagy	State Veterinar	Nagy,A;Cerni	State Veterir	2021-04-08
EPI_ISL_16972	A/chicken/Czech Republic/6542-2/2021	A / H5N8	Europe / Czech	Gallus gallu	Alexander Nagy	State Veterinar	Nagy,A;Cerni	State Veterir	2021-04-03
EPI_ISL_16971	A/chicken/Czech Republic/4980/2021	A / H5N8	Europe / Czech	Gallus gallu	Alexander Nagy	State Veterinar	Nagy,A;Cerni	State Veterir	2021-03-12
EPI_ISL_16971	A/chicken/Czech Republic/6527/2021	A / H5N8	Europe / Czech	Gallus gallu	Alexander Nagy	State Veterinar	Nagy,A;Cerni	State Veterir	2021-04-01
EPI_ISL_16971	A/chicken/Czech Republic/6532-2/2021	A / H5N8	Europe / Czech	Gallus gallu	Alexander Nagy	State Veterinar	Nagy,A;Cerni	State Veterir	2021-04-01
EPI_ISL_16971	A/chicken/Czech Republic/6532-1/2021	A / H5N8	Europe / Czech	Gallus gallu	Alexander Nagy	State Veterinar	Nagy,A;Cerni	State Veterir	2021-04-01
EPI_ISL_16971	A/chicken/Czech Republic/6542-1/2021	A / H5N8	Europe / Czech	Gallus gallu	Alexander Nagy	State Veterinar	Nagy,A;Cerni	State Veterir	2021-04-03
EPI_ISL_16971	A/chicken/Czech Republic/6654/2021	A / H5N8	Europe / Czech	Gallus gallu	Alexander Nagy	State Veterinar	Nagy,A;Cerni	State Veterir	2021-04-06
EPI_ISL_16971	A/chicken/Czech Republic/6684/2021	A / H5N8	Europe / Czech	Gallus gallu	Alexander Nagy	State Veterinar	Nagy,A;Cerni	State Veterir	2021-04-06
EPI_ISL_12381	A/Chicken/Sweden/SVA210302SZ0564/KN052	A / H5N5	Europe / Swede	Gallus gallu	Siamak Zohari	National Veterin		National Vet	2021-03-02
EPI_ISL_10580	A/chicken/Czech Republic/3099-1/2021	A / H5N8	Europe / Czech	Gallus gallu	Alexander Nagy	State Veterinar	Nagy,A; Cern	State Veterir	2021-02-12
EPI_ISL_63231	A/chicken/Netherlands/20017460-011015/2020	A / H5N8	Europe / Nethe	Gallus gallu	Rene Heutink (W	Wageningen Bi	Beerens, Nar	Wageningen	2020-11-09
EPI_ISL_62307	A/chicken/Netherlands/20017138-016020/2020	A / H5N8	Europe / Nethe	Gallus gallu	Rene Heutink (W	Wageningen Bi	Beerens, Nar	Wageningen	2020-11-04
EPI_ISL_14819	A/Wild Duck/Ningxia/Y99/2021	A / H5N8	Asia / China / N	Duck	Zeyu Yang (Ch	Chinese Acade		Xi'an Tianlon	2021-03-09
EPI_ISL_14819	A/Wild Duck/Ningxia/Y95/2021	A / H5N8	Asia / China / N	Duck	Zeyu Yang (Ch	Chinese Acade		Xi'an Tianlon	2021-03-09
EPI_ISL_13157	A/duck/Bangladesh/17D1868/2022	A / H5N1	Asia / Banglade	Duck	Mohammad Er	International Ce	Hossain, M.E	International	2022-03-06
EPI_ISL_13157	A/duck/Bangladesh/17D1865/2022	A / H5N1	Asia / Banglade	Duck	Mohammad Er	International Ce	Hossain, M.E	International	2022-03-06
EPI_ISL_13157	A/duck/Bangladesh/18D1840/2022	A / H5N1	Asia / Banglade	Duck	Mohammad Er	International Ce	Hossain, M.E	International	2022-02-25
EPI_ISL_13157	A/duck/Bangladesh/19D1859/2022	A / H5N1	Asia / Banglade	Duck	Mohammad Er	International Ce	Hossain, M.E	International	2022-02-23
EPI_ISL_13156	A/duck/Bangladesh/19D1850/2022	A / H5N1	Asia / Banglade	Duck	Mohammad Er	International Ce	Hossain, M.E	International	2022-02-23
EPI_ISL_13156	A/duck/Bangladesh/17D1854/2022	A / H5N1	Asia / Banglade	Duck	Mohammad Er	International Ce	Hossain, M.E	International	2022-02-13

EPI_ISL_12436	A/duck/Czech Republic/5361/2021	A / H5N8	Europe / Czech	Duck	Alexander Nagy	State Veterinar	Alexander,Na	State Veterir	2021-03-18
EPI_ISL_11208	A/duck/Zhejiang/S4854/2021(H5N6)	A / H5N6	Asia / China / Z	Duck	Pengfei Cui (Ha	Harbin Veterina	Pengfei Cui, (	Harbin Veter	2021-12-03
EPI_ISL_11208	A/duck/Yunnan/S4318/2021(H5N6)	A / H5N6	Asia / China / Y	Duck	Pengfei Cui (Ha	Harbin Veterina	Pengfei Cui, (	Harbin Veter	2021-11-02
EPI_ISL_11208	A/duck/Hunan/S40268/2021(H5N6)	A / H5N6	Asia / China / H	Duck	Pengfei Cui (Ha	Harbin Veterina	Pengfei Cui, (	Harbin Veter	2021-12-01
EPI_ISL_11208	A/duck/Hunan/S40199/2021(H5N6)	A / H5N6	Asia / China / H	Duck	Pengfei Cui (Ha	Harbin Veterina	Pengfei Cui, (	Harbin Veter	2021-12-01
EPI_ISL_11208	A/duck/Guizhou/S4702/2021(H5N6)	A / H5N6	Asia / China / G	Duck	Pengfei Cui (Ha	Harbin Veterina	Pengfei Cui, (	Harbin Veter	2021-12-02
EPI_ISL_11208	A/duck/Guangxi/S31116/2021(H5N6)	A / H5N6	Asia / China / G	Duck	Pengfei Cui (Ha	Harbin Veterina	Pengfei Cui, (	Harbin Veter	2021-10-18
EPI_ISL_11208	A/duck/Guangxi/S30428/2021(H5N6)	A / H5N6	Asia / China / G	Duck	Pengfei Cui (Ha	Harbin Veterina	Pengfei Cui, (	Harbin Veter	2021-10-19
EPI_ISL_11207	A/chicken/Bangladesh/19D1836/2022	A / H5N1	Asia / Banglade	Duck	Mohammad Er	International Ce	Hossain, M.E	International	2022-01-19
EPI_ISL_11207	A/chicken/Bangladesh/19D1833/2022	A / H5N1	Asia / Banglade	Duck	Mohammad Er	International Ce	Hossain, M.E	International	2022-01-19
EPI_ISL_11207	A/duck/Bangladesh/17D1821/2022	A / H5N1	Asia / Banglade	Duck	Mohammad Er	International Ce	Hossain, M.E	International	2022-01-30
EPI_ISL_11207	A/duck/Bangladesh/18D1795/2021	A / H5N1	Asia / Banglade	Duck	Mohammad Er	International Ce	Hossain, M.E	International	2021-12-10
EPI_ISL_11207	A/duck/Bangladesh/17D1815/2021	A / H5N3	Asia / Banglade	Duck	Mohammad Er	International Ce	Hossain, M.E	International	2021-12-05
EPI_ISL_79873	A/duck/Italy/21VIR8024-4/2021	A / H5N3	Europe / Italy	Duck	Adelaide Milani	Istituto Zooprof	Milani, A.; Fus	Istituto Zoopr	2021-09-23
EPI_ISL_79873	A/duck/Italy/21VIR8024-3/2021	A / H5N3	Europe / Italy	Duck	Adelaide Milani	Istituto Zooprof	Milani, A.; Fus	Istituto Zoopr	2021-09-23
EPI_ISL_79873	A/duck/Italy/21VIR8024-1/2021	A / H5N3	Europe / Italy	Duck	Adelaide Milani	Istituto Zooprof	Milani, A.; Fus	Istituto Zoopr	2021-09-23
EPI_ISL_77335	A/duck/Italy/21VIR10447/2021	A / H5N1	Europe / Italy /	Duck	Bianca Zecchin	Istituto Zooprof	Zecchin, B.; F	Istituto Zoopr	2021-12-01
EPI_ISL_73814	A/Duck/Guangdong/21964/2021(H5N8)	A / H5N8	Asia / China / G	Duck	Jiahao Zhang (	South China Ag	Zhang, Jiahao	South China	2021-08
EPI_ISL_73814	A/Duck/Shandong/21931-8/2021(H5N8)	A / H5N8	Asia / China / S	Duck	Jiahao Zhang (	South China Ag	Zhang, Jiahao	South China	2021-07
EPI_ISL_73813	A/Duck/Shandong/21644-7/2021(H5N8)	A / H5N8	Asia / China / S	Duck	Jiahao Zhang (	South China Ag	Zhang, Jiahao	South China	2021-05
EPI_ISL_73811	A/Duck/Shandong/21644-4/2021(H5N8)	A / H5N8	Asia / China / S	Duck	Jiahao Zhang (	South China Ag	Zhang, Jiahao	South China	2021-04
EPI_ISL_73495	A/Duck/Sichuan/21022-1/2021(H5N8)	A / H5N8	Asia / China / S	Duck	Jiahao Zhang (	South China Ag	Zhang, Jiahao	South China	2021-01
EPI_ISL_71927	A/Duck/Sichuan/21022-4/2021(H5N8)	A / H5N8	Asia / China / S	Duck	Jiahao Zhang (	South China Ag	Zhang, Jiahao	South China	2021-01
EPI_ISL_58656	A/garganey/Georgia/DT-22770/2020	A / H5N8	Asia / Georgia	Duck	Nicola Lewis (R	Royal Veterinar	Fouchier, R.A	Erasmus Me	2020-10-16
EPI_ISL_52604	A/duck/Hebei/S1070/2021	A / H5N8	Asia / China / H	Duck	Pengfei Cui (Ha	Harbin Veterina	Pengfei Cui, (	Harbin Veter	2021-04-27
EPI_ISL_52604	A/duck/Jiangxi/S10252/2021	A / H5N8	Asia / China / J	Duck	Pengfei Cui (Ha	Harbin Veterina	Pengfei Cui, (	Harbin Veter	2021-03-09
EPI_ISL_52604	A/duck/Henan/S1381/2021	A / H5N8	Asia / China / H	Duck	Pengfei Cui (Ha	Harbin Veterina	Pengfei Cui, (	Harbin Veter	2021-04-22
EPI_ISL_52604	A/duck/Guangxi/S21194/2021	A / H5N8	Asia / China / G	Duck	Pengfei Cui (Ha	Harbin Veterina	Pengfei Cui, (	Harbin Veter	2021-05-10
EPI_ISL_52604	A/duck/Guangxi/S11043/2021	A / H5N8	Asia / China / G	Duck	Pengfei Cui (Ha	Harbin Veterina	Pengfei Cui, (	Harbin Veter	2021-02-01
EPI_ISL_52604	A/duck/Guangxi/S10263/2021	A / H5N8	Asia / China / G	Duck	Pengfei Cui (Ha	Harbin Veterina	Pengfei Cui, (	Harbin Veter	2021-02-01
EPI_ISL_52604	A/duck/Guangxi/S10099/2021	A / H5N8	Asia / China / G	Duck	Pengfei Cui (Ha	Harbin Veterina	Pengfei Cui, (	Harbin Veter	2021-02-01
EPI_ISL_52604	A/duck/Guangdong/S1269/2021	A / H5N8	Asia / China / G	Duck	Pengfei Cui (Ha	Harbin Veterina	Pengfei Cui, (	Harbin Veter	2021-03-02
EPI_ISL_50961	A/domestic duck/Germany-NI/AI00646/2021	A / H5N8	Europe / Germa	Duck	Jacqueline King	Friedrich-Loeffl		Lebensmittel	2021-01-19
EPI_ISL_16971	A/duck/Czech Republic/6653-4/2021	A / H5N8	Europe / Czech	Duck	Alexander Nagy	State Veterinar	Nagy,A;Cerni	State Veterir	2021-04-06
EPI_ISL_11233	A/duck/England/046311/2020	A / H5N8	Europe / Unitec	Duck	Alex Byrne (Ani	Animal and Pla		Animal and F	2020-12-27
EPI_ISL_10411	A/mallard/Omsk region/45/2020	A / H5N2	Europe / Russia	Duck	Takehiko Saito	National Institut	Mine, J.; Uchi	Research Ins	2020-08-29
EPI_ISL_10411	A/duck/Chiba/D2A-9T/2021	A / H5N8	Asia / Japan / C	Duck	Takehiko Saito	National Institut		National Inst	2021-01-23
EPI_ISL_10411	A/duck/Chiba/D2A-7T/2021	A / H5N8	Asia / Japan / C	Duck	Takehiko Saito	National Institut		National Inst	2021-01-23
EPI_ISL_10411	A/duck/Chiba/D2A-5T/2021	A / H5N8	Asia / Japan / C	Duck	Takehiko Saito	National Institut		National Inst	2021-01-23
EPI_ISL_10411	A/duck/Chiba/D2A-3T/2021	A / H5N8	Asia / Japan / C	Duck	Takehiko Saito	National Institut		National Inst	2021-01-23
EPI_ISL_10411	A/duck/Chiba/D2A-2T/2021	A / H5N8	Asia / Japan / C	Duck	Takehiko Saito	National Institut		National Inst	2021-01-23
EPI_ISL_10411	A/duck/Chiba/D2A-1T/2021	A / H5N8	Asia / Japan / C	Duck	Takehiko Saito	National Institut		National Inst	2021-01-23

EPI_ISL_10411	A/duck/Chiba/D2A-10T/2021	A / H5N8	Asia / Japan / C	Duck	Takehiko Saito	National Institut		National Inst	2021-01-23
EPI_ISL_10411	A/duck/Chiba/D1B-9T/2021	A / H5N8	Asia / Japan / C	Duck	Takehiko Saito	National Institut		National Inst	2021-01-23
EPI_ISL_10411	A/duck/Chiba/D1B-8T/2021	A / H5N8	Asia / Japan / C	Duck	Takehiko Saito	National Institut		National Inst	2021-01-23
EPI_ISL_10411	A/duck/Chiba/D1B-7T/2021	A / H5N8	Asia / Japan / C	Duck	Takehiko Saito	National Institut		National Inst	2021-01-23
EPI_ISL_10411	A/duck/Chiba/D1B-6T/2021	A / H5N8	Asia / Japan / C	Duck	Takehiko Saito	National Institut		National Inst	2021-01-23
EPI_ISL_10411	A/duck/Chiba/D1B-5T/2021	A / H5N8	Asia / Japan / C	Duck	Takehiko Saito	National Institut		National Inst	2021-01-23
EPI_ISL_10411	A/duck/Chiba/D1B-4T/2021	A / H5N8	Asia / Japan / C	Duck	Takehiko Saito	National Institut		National Inst	2021-01-23
EPI_ISL_10411	A/duck/Chiba/D1B-3T/2021	A / H5N8	Asia / Japan / C	Duck	Takehiko Saito	National Institut		National Inst	2021-01-23
EPI_ISL_10411	A/duck/Chiba/D1B-2T/2021	A / H5N8	Asia / Japan / C	Duck	Takehiko Saito	National Institut		National Inst	2021-01-23
EPI_ISL_10411	A/duck/Chiba/D1B-1T/2021	A / H5N8	Asia / Japan / C	Duck	Takehiko Saito	National Institut		National Inst	2021-01-23
EPI_ISL_10411	A/duck/Chiba/D1B-10T/2021	A / H5N8	Asia / Japan / C	Duck	Takehiko Saito	National Institut		National Inst	2021-01-23
EPI_ISL_10411	A/duck/Chiba/D1A-9T/2021	A / H5N8	Asia / Japan / C	Duck	Takehiko Saito	National Institut		National Inst	2021-01-23
EPI_ISL_10411	A/duck/Chiba/D1A-8T/2021	A / H5N8	Asia / Japan / C	Duck	Takehiko Saito	National Institut		National Inst	2021-01-23
EPI_ISL_10411	A/duck/Chiba/D1A-7T/2021	A / H5N8	Asia / Japan / C	Duck	Takehiko Saito	National Institut		National Inst	2021-01-23
EPI_ISL_10411	A/duck/Chiba/D1A-6T/2021	A / H5N8	Asia / Japan / C	Duck	Takehiko Saito	National Institut		National Inst	2021-01-23
EPI_ISL_10411	A/duck/Chiba/D1A-5T/2021	A / H5N8	Asia / Japan / C	Duck	Takehiko Saito	National Institut		National Inst	2021-01-23
EPI_ISL_10411	A/duck/Chiba/D1A-4T/2021	A / H5N8	Asia / Japan / C	Duck	Takehiko Saito	National Institut		National Inst	2021-01-23
EPI_ISL_10411	A/duck/Chiba/D1A-3T/2021	A / H5N8	Asia / Japan / C	Duck	Takehiko Saito	National Institut		National Inst	2021-01-23
EPI_ISL_10411	A/duck/Chiba/D1A-2T/2021	A / H5N8	Asia / Japan / C	Duck	Takehiko Saito	National Institut		National Inst	2021-01-23
EPI_ISL_10411	A/duck/Chiba/D1A-1T/2021	A / H5N8	Asia / Japan / C	Duck	Takehiko Saito	National Institut		National Inst	2021-01-23
EPI_ISL_10411	A/duck/Chiba/D1A-10T/2021	A / H5N8	Asia / Japan / C	Duck	Takehiko Saito	National Institut		National Inst	2021-01-23
EPI_ISL_10411	A/duck/Chiba/D2B-9C/2021	A / H5N8	Asia / Japan / C	Duck	Takehiko Saito	National Institut		National Inst	2021-01-23
EPI_ISL_10411	A/duck/Chiba/D2B-7C/2021	A / H5N8	Asia / Japan / C	Duck	Takehiko Saito	National Institut		National Inst	2021-01-23
EPI_ISL_10411	A/duck/Chiba/D2A-8C/2021	A / H5N8	Asia / Japan / C	Duck	Takehiko Saito	National Institut		National Inst	2021-01-23
EPI_ISL_80404	A/Duck/Hungary/17957/2020	A / H5N8	Europe / Hunga	Duck	Katalin Szentpá	National Food (	Katalin,Szent	National Foo	2020-04-09
EPI_ISL_80404	A/Duck/Hungary/17806/2020	A / H5N8	Europe / Hunga	Duck	Katalin Szentpá	National Food (	Katalin,Szent	National Foo	2020-04-08
EPI_ISL_80404	A/Mallard_duck/Hungary/17319/2020	A / H5N8	Europe / Hunga	Duck	Katalin Szentpá	National Food (	Katalin,Szent	National Foo	2020-04-07
EPI_ISL_78009	A/Duck/Hungary/14788/2020	A / H5N8	Europe / Hunga	Duck	Katalin Szentpá	National Food (	Katalin,Szent	National Foo	2020-03-25
EPI_ISL_68128	A/duck/Niigata/151106/2020	A / H5N8	Asia / Japan / N	Duck	Takehiko Saito	National Institut		National Inst	2020-11-16
EPI_ISL_68127	A/duck/Niigata/151103/2/2020	A / H5N8	Asia / Japan / N	Duck	Takehiko Saito	National Institut		National Inst	2020-11-16
EPI_ISL_68127	A/duck/Niigata/151103/1/2020	A / H5N8	Asia / Japan / N	Duck	Takehiko Saito	National Institut		National Inst	2020-11-16
EPI_ISL_61507	A/domestic_duck/Kazakhstan/1-274-20-B/2020	A / H5N8	Asia / Kazakhst	Duck	Alex Byrne (Ani	Animal and Pla		National Vet	2020-09-25
EPI_ISL_52546	A/domestic_duck/Poland/271/2020	A / H5N8	Europe / Polan	Duck	Edyta Świętoń (	National Veterin	Swieton, E.; S	National Vet	2020-02-29
EPI_ISL_52546	A/domestic_duck/Poland/263/2020	A / H5N8	Europe / Polan	Duck	Edyta Świętoń (	National Veterin	Swieton, E.; S	National Vet	2020-02-26
EPI_ISL_52546	A/domestic_duck/Poland/285/2020	A / H5N8	Europe / Polan	Duck	Edyta Świętoń (	National Veterin	Swieton, E.; S	National Vet	2020-03-03
EPI_ISL_52545	A/domestic_duck/Poland/237/2020	A / H5N8	Europe / Polan	Duck	Edyta Świętoń (	National Veterin	Swieton, E.; S	National Vet	2020-02-24
EPI_ISL_52545	A/domestic_duck/Poland/230/2020	A / H5N8	Europe / Polan	Duck	Edyta Świętoń (	National Veterin	Swieton, E.; S	National Vet	2020-02-22
EPI_ISL_52545	A/domestic_duck/Poland/229/2020	A / H5N8	Europe / Polan	Duck	Edyta Świętoń (	National Veterin	Swieton, E.; S	National Vet	2020-02-22
EPI_ISL_52545	A/domestic_duck/Poland/223/2020	A / H5N8	Europe / Polan	Duck	Edyta Świętoń (	National Veterin	Swieton, E.; S	National Vet	2020-02-21
EPI_ISL_52545	A/domestic_duck/Poland/222/2020	A / H5N8	Europe / Polan	Duck	Edyta Świętoń (	National Veterin	Swieton, E.; S	National Vet	2020-02-21
EPI_ISL_52545	A/domestic_duck/Poland/221/2020	A / H5N8	Europe / Polan	Duck	Edyta Świętoń (	National Veterin	Swieton, E.; S	National Vet	2020-02-20
EPI_ISL_52544	A/domestic_duck/Poland/219/2020	A / H5N8	Europe / Polan	Duck	Edyta Świętoń (	National Veterin	Swieton, E.; S	National Vet	2020-02-20

EPI_ISL_5254	A/domestic_goose/Poland/028/2020	A / H5N8	Europe / Polan	Duck	Edyta Świętoń	National Veteri	Swieton, E.; S		National Vet	2020-01-12
EPI_ISL_4193	A/steamer duck/Germany-SN/AI00346/2020	A / H5N8	Europe / Germa	Duck	Jacqueline Kin	Friedrich-Loeffl			Landesunter	2020-03-26
EPI_ISL_4192	A/duck/Hungary/1565_20VIR749-2/2020	A / H5N8	Europe / Hunga	Duck	Ambra Pastori	Istituto Zooprof	Bálint,Ádám;M		National Foo	2020-01-10
EPI_ISL_9012	A/domestic_duck/Scotland/054469/2021	A / H5N1	Europe / Unite	Anas platyr	Alex Byrne (An	Animal and Pla			Animal and F	2021-11-01
EPI_ISL_1941	A/duck/Czech Republic/6653-15/2021	A / H5N8	Europe / Czech	Anas platyr	Alexander Nag	State Veterinar	Nagy,A;Cerni		State Veteri	2021-04-06
EPI_ISL_1941	A/duck/Czech Republic/6653-5/2021	A / H5N8	Europe / Czech	Anas platyr	Alexander Nag	State Veterinar	Nagy,A;Cerni		State Veteri	2021-04-06
EPI_ISL_1941	A/duck/Czech Republic/7681-10/2021	A / H5N8	Europe / Czech	Anas platyr	Alexander Nag	State Veterinar	Nagy,A;Cerni		State Veteri	2021-04-18
EPI_ISL_1941	A/duck/Czech Republic/7681-3/2021	A / H5N8	Europe / Czech	Anas platyr	Alexander Nag	State Veterinar	Nagy,A;Cerni		State Veteri	2021-04-18
EPI_ISL_1697	A/duck/Czech Republic/5360-2/2021	A / H5N8	Europe / Czech	Anas platyr	Alexander Nag	State Veterinar	Nagy,A;Cerni		State Veteri	2021-03-18
EPI_ISL_1697	A/duck/Czech Republic/5360-1/2021	A / H5N8	Europe / Czech	Anas platyr	Alexander Nag	State Veterinar	Nagy,A;Cerni		State Veteri	2021-03-18
EPI_ISL_1697	A/duck/Czech Republic/5448/2021	A / H5N8	Europe / Czech	Anas platyr	Alexander Nag	State Veterinar	Nagy,A;Cerni		State Veteri	2021-03-19
EPI_ISL_1697	A/duck/Czech Republic/5466/2021	A / H5N8	Europe / Czech	Anas platyr	Alexander Nag	State Veterinar	Nagy,A;Cerni		State Veteri	2021-03-19
EPI_ISL_1697	A/duck/Czech Republic/5792-19/2021	A / H5N8	Europe / Czech	Anas platyr	Alexander Nag	State Veterinar	Nagy,A;Cerni		State Veteri	2021-03-24
EPI_ISL_1697	A/duck/Czech Republic/5792-13/2021	A / H5N8	Europe / Czech	Anas platyr	Alexander Nag	State Veterinar	Nagy,A;Cerni		State Veteri	2021-03-24
EPI_ISL_1697	A/duck/Czech Republic/6653-6/2021	A / H5N8	Europe / Czech	Anas platyr	Alexander Nag	State Veterinar	Nagy,A;Cerni		State Veteri	2021-04-06
EPI_ISL_1481	A/Spot-billed Duck/Ningxia/Y16/2021	A / H5N8	Asia / China / N	Anas poeci	Zeyu Yang (Ch	Chinese Acade			Xi'an Tianlon	2021-03-11
EPI_ISL_1488	A/duck/Bangladesh/51600/2021	A / H5N1	Asia / Banglade	Anas sp.			Barman,S.; T			2021-12-19
EPI_ISL_1488	A/duck/Bangladesh/51602/2021	A / H5N1	Asia / Banglade	Anas sp.			Barman,S.; T			2021-12-19
EPI_ISL_1488	A/duck/Bangladesh/50053/2021	A / H5N1	Asia / Banglade	Anas sp.			Barman,S.; T			2021-09-18
EPI_ISL_1488	A/duck/Bangladesh/49673/2021	A / H5N1	Asia / Banglade	Anas sp.			Barman,S.; T			2021-08-28
EPI_ISL_1488	A/duck/Bangladesh/49933/2021	A / H5N1	Asia / Banglade	Anas sp.			Barman,S.; T			2021-09-18
EPI_ISL_1488	A/duck/Bangladesh/49672/2021	A / H5N1	Asia / Banglade	Anas sp.			Barman,S.; T			2021-08-28
EPI_ISL_1488	A/duck/Bangladesh/49996/2021	A / H5N1	Asia / Banglade	Anas sp.			Barman,S.; T			2021-09-18
EPI_ISL_1488	A/duck/Bangladesh/50189/2021	A / H5N1	Asia / Banglade	Anas sp.			Barman,S.; T			2021-10-16
EPI_ISL_1488	A/duck/Bangladesh/49995/2021	A / H5N1	Asia / Banglade	Anas sp.			Barman,S.; T			2021-09-18
EPI_ISL_1488	A/duck/Bangladesh/49670/2021	A / H5N1	Asia / Banglade	Anas sp.			Barman,S.; T			2021-08-28
EPI_ISL_1488	A/duck/Bangladesh/50044/2021	A / H5N1	Asia / Banglade	Anas sp.			Barman,S.; T			2021-12-20
EPI_ISL_1488	A/duck/Bangladesh/49671/2021	A / H5N1	Asia / Banglade	Anas sp.			Barman,S.; T			2021-08-28
EPI_ISL_1488	A/duck/Bangladesh/50550/2021	A / H5N1	Asia / Banglade	Anas sp.			Barman,S.; T			2021-12-14
EPI_ISL_1488	A/duck/Bangladesh/50661/2021	A / H5N1	Asia / Banglade	Anas sp.			Barman,S.; T			2021-12-14
EPI_ISL_1488	A/duck/Bangladesh/49737/2021	A / H5N1	Asia / Banglade	Anas sp.			Barman,S.; T			2021-08-28
EPI_ISL_1488	A/duck/Bangladesh/50622/2021	A / H5N1	Asia / Banglade	Anas sp.			Barman,S.; T			2021-12-14
EPI_ISL_1488	A/duck/Bangladesh/49735/2021	A / H5N1	Asia / Banglade	Anas sp.			Barman,S.; T			2021-08-28
EPI_ISL_1488	A/duck/Bangladesh/50664/2021	A / H5N1	Asia / Banglade	Anas sp.			Barman,S.; T			2021-12-14
EPI_ISL_4073	A/duck/Bangladesh/45724/2020	A / H5N1	Asia / Banglade	Anas sp.			Barman,S.; T			2020-08-22
EPI_ISL_4073	A/duck/Bangladesh/45726/2020	A / H5N1	Asia / Banglade	Anas sp.			Barman,S.; T			2020-08-22
EPI_ISL_4073	A/duck/Bangladesh/44508/2020	A / H5N6	Asia / Banglade	Anas sp.			Barman,S.; T			2020-02-22
EPI_ISL_4073	A/duck/Bangladesh/44523/2020	A / H5N6	Asia / Banglade	Anas sp.			Barman,S.; T			2020-02-22
EPI_ISL_4073	A/duck/Bangladesh/44477/2020	A / H5N6	Asia / Banglade	Anas sp.			Barman,S.; T			2020-02-22
EPI_ISL_4073	A/duck/Bangladesh/44471/2020	A / H5N6	Asia / Banglade	Anas sp.			Barman,S.; T			2020-02-22
EPI_ISL_4073	A/duck/Bangladesh/44456/2020	A / H5N6	Asia / Banglade	Anas sp.			Barman,S.; T			2020-02-22
EPI_ISL_4073	A/duck/Bangladesh/44430/2020	A / H5N6	Asia / Banglade	Anas sp.			Barman,S.; T			2020-02-22

EPI_ISL_40731	A/duck/Bangladesh/44447/2020	A / H5N6	Asia / Banglade	Anas sp.			Barman,S.; T		2020-02-22
EPI_ISL_40731	A/duck/Bangladesh/44418/2020	A / H5N6	Asia / Banglade	Anas sp.			Barman,S.; T		2020-02-22
EPI_ISL_40731	A/duck/Bangladesh/44484/2020	A / H5N6	Asia / Banglade	Anas sp.			Barman,S.; T		2020-02-22
EPI_ISL_40731	A/duck/Bangladesh/44502/2020	A / H5N6	Asia / Banglade	Anas sp.			Barman,S.; T		2020-02-22
EPI_ISL_40731	A/duck/Bangladesh/44417/2020	A / H5N6	Asia / Banglade	Anas sp.			Barman,S.; T		2020-02-22
EPI_ISL_40731	A/duck/Bangladesh/44469/2020	A / H5N6	Asia / Banglade	Anas sp.			Barman,S.; T		2020-02-22
EPI_ISL_40731	A/duck/Bangladesh/44453/2020	A / H5N6	Asia / Banglade	Anas sp.			Barman,S.; T		2020-02-22
EPI_ISL_40731	A/duck/Bangladesh/44524/2020	A / H5N6	Asia / Banglade	Anas sp.			Barman,S.; T		2020-02-22
EPI_ISL_40731	A/duck/Bangladesh/44440/2020	A / H5N6	Asia / Banglade	Anas sp.			Barman,S.; T		2020-02-22
EPI_ISL_40731	A/duck/Bangladesh/44423/2020	A / H5N6	Asia / Banglade	Anas sp.			Barman,S.; T		2020-02-22
EPI_ISL_40731	A/duck/Bangladesh/44448/2020	A / H5N6	Asia / Banglade	Anas sp.			Barman,S.; T		2020-02-22
EPI_ISL_40731	A/duck/Bangladesh/44433/2020	A / H5N6	Asia / Banglade	Anas sp.			Barman,S.; T		2020-02-22
EPI_ISL_40731	A/duck/Bangladesh/45729/2020	A / H5N1	Asia / Banglade	Anas sp.			Barman,S.; T		2020-08-22
EPI_ISL_40717	A/duck/Bangladesh/43119/2020	A / H5N6	Asia / Banglade	Anas sp.			Barman,S.; T		2020-01-27
EPI_ISL_40717	A/duck/Bangladesh/43411/2020	A / H5N1	Asia / Banglade	Anas sp.			Barman,S.; T		2020-02-19
EPI_ISL_40717	A/duck/Bangladesh/43122/2020	A / H5N6	Asia / Banglade	Anas sp.			Barman,S.; T		2020-01-27
EPI_ISL_40716	A/duck/Bangladesh/43129/2020	A / H5N6	Asia / Banglade	Anas sp.			Barman,S.; T		2020-01-27
EPI_ISL_40716	A/duck/Bangladesh/43099/2020	A / H5N6	Asia / Banglade	Anas sp.			Barman,S.; T		2020-01-27
EPI_ISL_40716	A/duck/Bangladesh/43120/2020	A / H5N6	Asia / Banglade	Anas sp.			Barman,S.; T		2020-01-27
EPI_ISL_40716	A/duck/Bangladesh/43082/2020	A / H5N6	Asia / Banglade	Anas sp.			Barman,S.; T		2020-01-27
EPI_ISL_40716	A/duck/Bangladesh/43128/2020	A / H5N6	Asia / Banglade	Anas sp.			Barman,S.; T		2020-01-27
EPI_ISL_40716	A/duck/Bangladesh/43521/2020	A / H5N1	Asia / Banglade	Anas sp.			Barman,S.; T		2020-02-18
EPI_ISL_40716	A/duck/Bangladesh/43123/2020	A / H5N6	Asia / Banglade	Anas sp.			Barman,S.; T		2020-01-27
EPI_ISL_40715	A/duck/Bangladesh/43050/2020	A / H5N6	Asia / Banglade	Anas sp.			Barman,S.; T		2020-01-27
EPI_ISL_40715	A/duck/Bangladesh/43127/2020	A / H5N6	Asia / Banglade	Anas sp.			Barman,S.; T		2020-01-27
EPI_ISL_11390	A/wild duck/Netherlands/20017794-001/2020	A / H5N8	Europe / Nethe	Anas sp.	Rene Heutink ('	Wageningen Bi	Beerens, Nar	Wageningen	2020-11-12
EPI_ISL_21725	A/gadwall/Netherlands/21024401-002/2021	A / H5N8	Europe / Nethe	Anas strepe	Rene Heutink ('	Wageningen Bi	Beerens, Nar	Wageningen	2021-01-25
EPI_ISL_11390	A/gadwall/Netherlands/20017716-001/2020	A / H5N8	Europe / Nethe	Anas strepe	Rene Heutink ('	Wageningen Bi	Beerens, Nar	Wageningen	2020-11-10
EPI_ISL_11390	A/gadwall/Netherlands/20017254-001/2020	A / H5N8	Europe / Nethe	Anas strepe	Rene Heutink ('	Wageningen Bi	Beerens, Nar	Wageningen	2020-11-01
EPI_ISL_11330	A/white-tailed eagle/Hokkaido/22-RU-WTE-2/2	A / H5N1	Asia / Japan / H	Eagle	Norikazu Isoda	Graduate Scho	Sakoda, Yosh	Graduate Sc	2022-01-02
EPI_ISL_11391	A/northern goshawk/Netherlands/20018560-00	A / H5N8	Europe / Nethe	Accipiter ge	Rene Heutink ('	Wageningen Bi	Beerens, Nar	Wageningen	2020-11-22
EPI_ISL_72672	A/common buzzard/Netherlands/21038793-00	A / H5N1	Europe / Nethe	Buteo butee	Rene Heutink ('	Wageningen Bi	Beerens, Nar	Wageningen	2021-11-12
EPI_ISL_21725	A/common buzzard/Netherlands/21024712-00	A / H5N8	Europe / Nethe	Buteo butee	Rene Heutink ('	Wageningen Bi	Beerens, Nar	Wageningen	2021-03-04
EPI_ISL_21725	A/common buzzard/Netherlands/21024357-00	A / H5N3	Europe / Nethe	Buteo butee	Rene Heutink ('	Wageningen Bi	Beerens, Nar	Wageningen	2021-02-22
EPI_ISL_21725	A/common buzzard/Netherlands/21023939-00	A / H5N8	Europe / Nethe	Buteo butee	Rene Heutink ('	Wageningen Bi	Beerens, Nar	Wageningen	2021-02-21
EPI_ISL_21725	A/common buzzard/Netherlands/21021497-00	A / H5N8	Europe / Nethe	Buteo butee	Rene Heutink ('	Wageningen Bi	Beerens, Nar	Wageningen	2021-01-10
EPI_ISL_21725	A/common buzzard/Netherlands/21021278-00	A / H5N8	Europe / Nethe	Buteo butee	Rene Heutink ('	Wageningen Bi	Beerens, Nar	Wageningen	2021-01-05
EPI_ISL_11391	A/common buzzard/Netherlands/20018339-00	A / H5N8	Europe / Nethe	Buteo butee	Rene Heutink ('	Wageningen Bi	Beerens, Nar	Wageningen	2020-11-16
EPI_ISL_11390	A/common buzzard/Netherlands/20017824-00	A / H5N8	Europe / Nethe	Buteo butee	Rene Heutink ('	Wageningen Bi	Beerens, Nar	Wageningen	2020-11-12
EPI_ISL_98469	A/common buzzard/Denmark/14600-1/2020	A / H5N8	Europe / Denm	Buteo butee	Charlotte Kristia	Statens Serum	Yuan Liang, (Liang	Statens Seru	2020-11-06
EPI_ISL_14393	A/Branta_canadensis/Belgium/5177_0003/202	A / H5N1	Europe / Belgiu	Branta cana	Steven Van Bo	Sciensano, Dep	Van Borm, St	Sciensano -	2022-04-13
EPI_ISL_14393	A/Branta_canadensis/Belgium/4821_0001/202	A / H5N1	Europe / Belgiu	Branta cana	Steven Van Bo	Sciensano, Dep	Van Borm, St	Sciensano -	2022-04-07

EPI_ISL_1139(	A/greater canada goose/Netherlands/2001740:	A / H5N8	Europe / Nethe	Branta cana	Rene Heutink ('	Wageningen Bi	Beerens, Nar	Wageningen	2020-11-07
EPI_ISL_1139(	A/greater canada goose/Netherlands/2001747!	A / H5N8	Europe / Nethe	Branta cana	Rene Heutink ('	Wageningen Bi	Beerens, Nar	Wageningen	2020-11-06
EPI_ISL_14098	A/harbor seal/Maine/22-020983-007-original/2(	A / H5N1	North America /	Seal	Mary Lea Killiar	National Veterii	Chinh,Thanh;	National Vet	2022-06-29
EPI_ISL_14098	A/harbor seal/Maine/22-020983-006-original/2(	A / H5N1	North America /	Seal	Mary Lea Killiar	National Veterii	Chinh,Thanh;	National Vet	2022-06-29
EPI_ISL_14098	A/grey seal/Maine/22-020983-003-original/202	A / H5N1	North America /	Seal	Mary Lea Killiar	National Veterii	Chinh,Thanh;	National Vet	2022-06-29
EPI_ISL_14098	A/harbor seal/Maine/22-020983-002-original/2(	A / H5N1	North America /	Seal	Mary Lea Killiar	National Veterii	Chinh,Thanh;	National Vet	2022-06-29
EPI_ISL_14098	A/harbor seal/Maine/22-020983-001-original/2(	A / H5N1	North America /	Seal	Mary Lea Killiar	National Veterii	Chinh,Thanh;	National Vet	2022-06-29
EPI_ISL_14098	A/harbor seal/Maine/22-020455-005-original/2(	A / H5N1	North America /	Seal	Mary Lea Killiar	National Veterii	Chinh,Thanh;	National Vet	2022-06-27
EPI_ISL_14098	A/harbor seal/Maine/22-020455-004-original/2(	A / H5N1	North America /	Seal	Mary Lea Killiar	National Veterii	Chinh,Thanh;	National Vet	2022-06-26
EPI_ISL_14098	A/harbor seal/Maine/22-020455-003-original/2(	A / H5N1	North America /	Seal	Mary Lea Killiar	National Veterii	Chinh,Thanh;	National Vet	2022-06-22
EPI_ISL_14098	A/harbor seal/Maine/22-020455-002-original/2(	A / H5N1	North America /	Seal	Mary Lea Killiar	National Veterii	Chinh,Thanh;	National Vet	2022-06-24
EPI_ISL_14098	A/harbor seal/Maine/22-020455-001-original/2(	A / H5N1	North America /	Seal	Mary Lea Killiar	National Veterii	Chinh,Thanh;	National Vet	2022-06-24
EPI_ISL_20815	A/seal/England/AVP-031141/2020	A / H5N8	Europe / Unitec	Seal	Alex Byrne (Ani	Animal and Pla		Animal and F	2020-12-08
EPI_ISL_1255(	A/tufted_duck/Poland/MB061/2021(H5N5)	A / H5N5	Europe / Polanc	Aythya fulig	Edyta Świętoń	National Veterii	Swieton E., S	National Vet	2021-02-01
EPI_ISL_40716	A/Ferruginous duck/Bangladesh/42380/2020	A / H5N6	Asia / Banglade	Aythya nyrc			Barman,S.; T		2020-01-19
EPI_ISL_14917	A/common_murre/Poland/MB151/2022	A / H5N1	Europe / Polanc	Wild bird	Edyta Świętoń	National Veterii	Swieton E., S	National Vet	2022-07-13
EPI_ISL_77336	A/kestrel/Italy/21VIR10468/2021	A / H5N1	Europe / Italy /	Wild bird	Bianca Zecchin	Istituto Zooprof	Zecchin, B.; F	Istituto Zoopr	2021-11-30
EPI_ISL_77336	A/seagull/Italy/21VIR9432-2/2021	A / H5N1	Europe / Italy /	Wild bird	Bianca Zecchin	Istituto Zooprof	Zecchin, B.; F	Istituto Zoopr	2021-11-11
EPI_ISL_77336	A/magpie/Italy/21VIR9487-2/2021	A / H5N1	Europe / Italy /	Wild bird	Bianca Zecchin	Istituto Zooprof	Zecchin, B.; F	Istituto Zoopr	2021-11-05
EPI_ISL_77336	A/Wild_goose/Italy/21VIR10193/2021	A / H5N1	Europe / Italy /	Wild bird	Bianca Zecchin	Istituto Zooprof	Zecchin, B.; F	Istituto Zoopr	2021-11-23
EPI_ISL_77335	A/mallard/Italy/21VIR8919-2/2021	A / H5N1	Europe / Italy /	Wild bird	Bianca Zecchin	Istituto Zooprof	Zecchin, B.; F	Istituto Zoopr	2021-10-27
EPI_ISL_77335	A/Eurasian_wigeon/Italy/21VIR8919-3/2021	A / H5N1	Europe / Italy /	Wild bird	Bianca Zecchin	Istituto Zooprof	Zecchin, B.; F	Istituto Zoopr	2021-10-28
EPI_ISL_55306	A/great_skua/Scotland/041672/2021	A / H5N1	Europe / Unitec	Wild bird	Alex Byrne (Ani	Animal and Pla		Animal and F	2021-07-20
EPI_ISL_10988	A/crane/Kagoshima/KU-93/2021	A / H5N8	Asia / Japan / K	Wild bird	Ahmed Magdy	Kagoshima Uni		Kagoshima U	2021-01-19
EPI_ISL_14819	A/Mallard/Ningxia/BY246/2020	A / H5N8	Asia / China / N	Mallard	Zeyu Yang (Ch	Chinese Acade		Xi'an Tianlon	2020-10-16
EPI_ISL_79873	A/mallard/Italy/21VIR8919-1/2021	A / H5N2	Europe / Italy	Mallard	Adelaide Milani	Istituto Zooprof	Milani, A.; Fus	Istituto Zoopr	2021-10-27
EPI_ISL_79873	A/mallard/Italy/21VIR6957-6/2021	A / H5N1	Europe / Italy	Mallard	Adelaide Milani	Istituto Zooprof	Milani, A.; Fus	Istituto Zoopr	2021-08-20
EPI_ISL_58656	A/mallard/Georgia/DT-22356/2020	A / H5N8	Asia / Georgia	Mallard	Nicola Lewis (F	Royal Veterinar	Fouchier, R.A	Erasmus Me	2020-10-02
EPI_ISL_58655	A/mallard/Georgia/DT-22362/2020	A / H5N8	Asia / Georgia	Mallard	Nicola Lewis (F	Royal Veterinar	Fouchier, R.A	Erasmus Me	2020-10-02
EPI_ISL_5096(	A/mallard/Germany-MV/AI00639/2021	A / H5N8	Europe / Germa	Mallard	Jacqueline King	Friedrich-Loeffl		Friedrich-Loe	2021-01-20
EPI_ISL_3950(	A/Mallard/Sweden/SVA210827SZ0252/KN002!	A / H5N8	Europe / Swede	Mallard	Siamak Zohari	National Veterii		National Vet	2021-08-26
EPI_ISL_3950(	A/Mallard/Sweden/SVA210827SZ0252/KN002!	A / H5N8	Europe / Swede	Mallard	Siamak Zohari	National Veterii		National Vet	2021-08-26
EPI_ISL_11147	A/mallard/Kagoshima/KU-d89/2021	A / H5N8	Asia / Japan / K	Mallard	Ahmed Magdy	National Institut		National Inst	2021-01-16
EPI_ISL_69777	A/northern pintail/Hokkaido/M13/2020	A / H5N8	Asia / Japan / H	Northern pi			Sakoda,Y.; Is		2020-10-24
EPI_ISL_11391	A/eurasian curlew/Netherlands/20016896-019/	A / H5N8	Europe / Nethe	Eurasian cu	Rene Heutink ('	Wageningen Bi	Beerens, Nar	Wageningen	2020-11-02
EPI_ISL_6641(	A/Numenius_arquata/Belgium/11956_003/202(	A / H5N8	Europe / Belgiu	Eurasian cu	Steven Van Bor	Sciensano, Dep	Van Borm,Ste	Sciensano -	2020-11-07
EPI_ISL_63231	A/eurasian curlew/Netherlands/20016890-001/	A / H5N1	Europe / Nethe	Eurasian cu	Rene Heutink ('	Wageningen Bi	Beerens, Nar	Wageningen	2020-11-01
EPI_ISL_1139(	A/eurasian wigeon/Netherlands/20017908-002	A / H5N8	Europe / Nethe	Mareca per	Rene Heutink ('	Wageningen Bi	Beerens, Nar	Wageningen	2020-11-10
EPI_ISL_1139(	A/eurasian wigeon/Netherlands/20016896-025	A / H5N8	Europe / Nethe	Mareca per	Rene Heutink ('	Wageningen Bi	Beerens, Nar	Wageningen	2020-11-02
EPI_ISL_1139(	A/eurasian wigeon/Netherlands/20016758-001	A / H5N8	Europe / Nethe	Mareca per	Rene Heutink ('	Wageningen Bi	Beerens, Nar	Wageningen	2020-10-29
EPI_ISL_10576	A/Anser_anser/Belgium/1809_0002/2022	A / H5N1	Europe / Belgiu	Greylag go	Steven Van Bor	Sciensano, Dep	Van Borm, St	Sciensano -	2022-01-23
EPI_ISL_70545	A/greylag goose /Sweden/SVA211111SZ0376/	A / H5N1	Europe / Swede	Greylag go	Siamak Zohari	National Veterii		National Vet	2021-11-08

EPI_ISL_98469	A/graylag goose/Denmark/14535-1/2020	A / H5N8	Europe / Denm	Greylag goo	Charlotte Kristia	Statens Serum	Yuan Liang, (	Liang	Statens Seru	2020-11-09
EPI_ISL_63231	A/greylag goose/Netherlands/20016879-001/2(	A / H5N8	Europe / Nethe	Greylag goo	Rene Heutink ('	Wageningen Bi	Beerens, Nar		Wageningen	2020-11-01
EPI_ISL_63231	A/greylag goose/Netherlands/20016582-004/2(	A / H5N1	Europe / Nethe	Greylag goo	Rene Heutink ('	Wageningen Bi	Beerens, Nar		Wageningen	2020-10-28
EPI_ISL_11390	A/cormorant/Netherlands/20016582-005/2020	A / H5N8	Europe / Nethe	Cormorant	Rene Heutink ('	Wageningen Bi	Beerens, Nar		Wageningen	2020-10-28
EPI_ISL_79873	A/teal/Italy/21VIR8923-73/2021	A / H5N3	Europe / Italy	Teal	Adelaide Milani	Istituto Zooprof	Milani, A.; Fus		Istituto Zoopr	2021-10-29
EPI_ISL_11391	A/muscovy duck/Netherlands/20018067-001/2(	A / H5N8	Europe / Nethe	Cairina mos	Rene Heutink ('	Wageningen Bi	Beerens, Nar		Wageningen	2020-11-12
EPI_ISL_11390	A/muscovy duck/Netherlands/20017611-002/2(	A / H5N8	Europe / Nethe	Cairina mos	Rene Heutink ('	Wageningen Bi	Beerens, Nar		Wageningen	2020-11-10
EPI_ISL_76359	A/white-tailed eagle/Sweden/SVA211201SZ03	A / H5N1	Europe / Swede	Haliaeetus	Siamak Zohari	National Veterii			National Vete	2021-11-26
EPI_ISL_21725	A/sanderling/Netherlands/21021794-002/2021	A / H5N8	Europe / Nethe	Calidris alb	Rene Heutink ('	Wageningen Bi	Beerens, Nar		Wageningen	2021-01-18
EPI_ISL_11390	A/brant goose/Netherlands/20016948-002/202	A / H5N8	Europe / Nethe	Branta berr	Rene Heutink ('	Wageningen Bi	Beerens, Nar		Wageningen	2020-10-31
EPI_ISL_11233	A/brent_goose/England/095684/2020	A / H5N5	Europe / Unitec	Branta berr	Alex Byrne (Ani	Animal and Pla			Animal and F	2020-11-12
EPI_ISL_11233	A/brent_goose/England/233339/2020	A / H5N8	Europe / Unitec	Branta berr	Alex Byrne (Ani	Animal and Pla			Animal and F	2020-11-08
EPI_ISL_98468	A/black-headed gull/Denmark/14139-4/2020	A / H5N8	Europe / Denm	Chroicocep	Charlotte Kristia	Statens Serum	Yuan Liang, (	Liang	Statens Seru	2020-11-04
EPI_ISL_70538	A/barnacle goose/Sweden/SVA211111SZ0376	A / H5N1	Europe / Swede	Branta leuc	Siamak Zohari	National Veterii			National Vete	2021-11-01
EPI_ISL_65962	A/barnacle goose/Sweden/SVA211102SZ0402	A / H5N1	Europe / Swede	Branta leuc	Siamak Zohari	National Veterii			National Vete	2021-11-02
EPI_ISL_54493	A/barnacle_goose/Denmark/17572-1.01/2021-(	A / H5N1	Europe / Denm	Branta leuc	Charlotte Kristia	Statens Serum	Hjulsager, C		Statens Seru	2021-03-01
EPI_ISL_51462	A/barnacle goose/Germany-NI/AI03980/2021	A / H5N1	Europe / Germa	Branta leuc	Jacqueline King	Friedrich-Loeffl			Lebensmittel	2021-04-14
EPI_ISL_51454	A/barnacle goose/Germany-NI/AI03914/2021	A / H5N1	Europe / Germa	Branta leuc	Jacqueline King	Friedrich-Loeffl			Lebensmittel	2021-04-08
EPI_ISL_50637	A/barnacle goose/Germany-SH/AI02379/2020	A / H5N8	Europe / Germa	Branta leuc	Jacqueline King	Friedrich-Loeffl			Landeslabor	2020-11-06
EPI_ISL_21726	A/barnacle goose/Netherlands/21024897-001/2	A / H5N8	Europe / Nethe	Branta leuc	Rene Heutink ('	Wageningen Bi	Beerens, Nar		Wageningen	2021-03-09
EPI_ISL_21725	A/barnacle goose/Netherlands/21024358-001/2	A / H5N8	Europe / Nethe	Branta leuc	Rene Heutink ('	Wageningen Bi	Beerens, Nar		Wageningen	2021-02-21
EPI_ISL_21725	A/barnacle goose/Netherlands/21024066-001/2	A / H5N1	Europe / Nethe	Branta leuc	Rene Heutink ('	Wageningen Bi	Beerens, Nar		Wageningen	2021-02-22
EPI_ISL_21725	A/barnacle goose/Netherlands/21022039-002/2	A / H5N8	Europe / Nethe	Branta leuc	Rene Heutink ('	Wageningen Bi	Beerens, Nar		Wageningen	2021-01-19
EPI_ISL_11391	A/barnacle goose/Netherlands/20018737-002/2	A / H5N8	Europe / Nethe	Branta leuc	Rene Heutink ('	Wageningen Bi	Beerens, Nar		Wageningen	2020-11-24
EPI_ISL_11391	A/barnacle goose/Netherlands/20017604-001/2	A / H5N8	Europe / Nethe	Branta leuc	Rene Heutink ('	Wageningen Bi	Beerens, Nar		Wageningen	2020-11-08
EPI_ISL_11390	A/barnacle goose/Netherlands/20017984-004/2	A / H5N8	Europe / Nethe	Branta leuc	Rene Heutink ('	Wageningen Bi	Beerens, Nar		Wageningen	2020-11-13
EPI_ISL_11390	A/barnacle goose/Netherlands/20017713-002/2	A / H5N8	Europe / Nethe	Branta leuc	Rene Heutink ('	Wageningen Bi	Beerens, Nar		Wageningen	2020-11-08
EPI_ISL_11390	A/barnacle goose/Netherlands/20017557-002/2	A / H5N8	Europe / Nethe	Branta leuc	Rene Heutink ('	Wageningen Bi	Beerens, Nar		Wageningen	2020-11-10
EPI_ISL_11390	A/barnacle goose/Netherlands/20017557-001/2	A / H5N8	Europe / Nethe	Branta leuc	Rene Heutink ('	Wageningen Bi	Beerens, Nar		Wageningen	2020-11-10
EPI_ISL_11390	A/barnacle goose/Netherlands/20016935-003/2	A / H5N8	Europe / Nethe	Branta leuc	Rene Heutink ('	Wageningen Bi	Beerens, Nar		Wageningen	2020-11-01
EPI_ISL_11390	A/barnacle goose/Netherlands/20016896-012/2	A / H5N8	Europe / Nethe	Branta leuc	Rene Heutink ('	Wageningen Bi	Beerens, Nar		Wageningen	2020-11-02
EPI_ISL_11390	A/barnacle goose/Netherlands/20016896-011/2	A / H5N8	Europe / Nethe	Branta leuc	Rene Heutink ('	Wageningen Bi	Beerens, Nar		Wageningen	2020-11-02
EPI_ISL_11390	A/barnacle goose/Netherlands/20016888-003/2	A / H5N8	Europe / Nethe	Branta leuc	Rene Heutink ('	Wageningen Bi	Beerens, Nar		Wageningen	2020-10-29
EPI_ISL_11390	A/barnacle goose/Netherlands/20016888-002/2	A / H5N8	Europe / Nethe	Branta leuc	Rene Heutink ('	Wageningen Bi	Beerens, Nar		Wageningen	2020-10-29
EPI_ISL_11390	A/barnacle goose/Netherlands/20016951-001/2	A / H5N8	Europe / Nethe	Branta leuc	Rene Heutink ('	Wageningen Bi	Beerens, Nar		Wageningen	2020-10-31
EPI_ISL_11390	A/barnacle goose/Netherlands/20017405-002/2	A / H5N8	Europe / Nethe	Branta leuc	Rene Heutink ('	Wageningen Bi	Beerens, Nar		Wageningen	2020-11-07
EPI_ISL_11390	A/barnacle goose/Netherlands/20017159-006/2	A / H5N8	Europe / Nethe	Branta leuc	Rene Heutink ('	Wageningen Bi	Beerens, Nar		Wageningen	2020-11-04
EPI_ISL_11390	A/barnacle goose/Netherlands/20017052-001/2	A / H5N8	Europe / Nethe	Branta leuc	Rene Heutink ('	Wageningen Bi	Beerens, Nar		Wageningen	2020-11-03
EPI_ISL_11390	A/barnacle goose/Netherlands/20017051-006/2	A / H5N8	Europe / Nethe	Branta leuc	Rene Heutink ('	Wageningen Bi	Beerens, Nar		Wageningen	2020-11-03
EPI_ISL_11390	A/barnacle goose/Netherlands/20017051-001/2	A / H5N8	Europe / Nethe	Branta leuc	Rene Heutink ('	Wageningen Bi	Beerens, Nar		Wageningen	2020-11-03
EPI_ISL_11390	A/barnacle goose/Netherlands/20016974-002/2	A / H5N8	Europe / Nethe	Branta leuc	Rene Heutink ('	Wageningen Bi	Beerens, Nar		Wageningen	2020-11-02
EPI_ISL_11390	A/barnacle goose/Netherlands/20016888-001/2	A / H5N8	Europe / Nethe	Branta leuc	Rene Heutink ('	Wageningen Bi	Beerens, Nar		Wageningen	2020-10-29

EPI_ISL_1139	A/barnacle goose/Netherlands/20016511-002/	A / H5N8	Europe / Nethe	Branta leuc	Rene Heutink (	Wageningen Bi	Beerens, Nar		Wageningen	2020-10-27
EPI_ISL_9846	A/barnacle goose/Denmark/14599-1/2020	A / H5N8	Europe / Denm	Branta leuc	Charlotte Kristi	Statens Serum	Yuan Liang,	Liang	Statens Seru	2020-11-07
EPI_ISL_9846	A/barnacle goose/Denmark/14600-2/2020	A / H5N8	Europe / Denm	Branta leuc	Charlotte Kristi	Statens Serum	Yuan Liang,	Liang	Statens Seru	2020-11-07
EPI_ISL_9846	A/barnacle goose/Denmark/14538-1/2020	A / H5N8	Europe / Denm	Branta leuc	Charlotte Kristi	Statens Serum	Yuan Liang,	Liang	Statens Seru	2020-11-15
EPI_ISL_9846	A/barnacle goose/Denmark/14536-1/2020	A / H5N8	Europe / Denm	Branta leuc	Charlotte Kristi	Statens Serum	Yuan Liang,	Liang	Statens Seru	2020-11-05
EPI_ISL_9846	A/barnacle goose/Denmark/14537-1/2020	A / H5N8	Europe / Denm	Branta leuc	Charlotte Kristi	Statens Serum	Yuan Liang,	Liang	Statens Seru	2020-11-04
EPI_ISL_9846	A/barnacle goose/Denmark/14534-1/2020	A / H5N8	Europe / Denm	Branta leuc	Charlotte Kristi	Statens Serum	Yuan Liang,	Liang	Statens Seru	2020-11-04
EPI_ISL_9846	A/barnacle goose/Denmark/14139-3/2020	A / H5N8	Europe / Denm	Branta leuc	Charlotte Kristi	Statens Serum	Yuan Liang,	Liang	Statens Seru	2020-11-04
EPI_ISL_9846	A/barnacle goose/Denmark/14139-2/2020	A / H5N8	Europe / Denm	Branta leuc	Charlotte Kristi	Statens Serum	Yuan Liang,	Liang	Statens Seru	2020-11-04
EPI_ISL_9846	A/barnacle goose/Denmark/14139-1/2020	A / H5N8	Europe / Denm	Branta leuc	Charlotte Kristi	Statens Serum	Yuan Liang,	Liang	Statens Seru	2020-11-04
EPI_ISL_7221	A/barnacle goose/Sweden/SVA201201SZ0353	A / H5N5	Europe / Swed	Branta leuc	Siamak Zohari	National Veteri			National Vet	2020-11-25
EPI_ISL_6323	A/barnacle goose/Netherlands/20016935-002/	A / H5N8	Europe / Nethe	Branta leuc	Rene Heutink (	Wageningen Bi	Beerens, Nar		Wageningen	2020-11-01
EPI_ISL_6144	A/barnacle goose/Germany-SH/AI02167/2020	A / H5N8	Europe / Germ	Branta leuc	Jacqueline King	Friedrich-Loeffl			Landeslabor	2020-10-28
EPI_ISL_1369	A/chicken/East_Java/Av1955/2022	A / H5N1	Asia / Indonesi	Gallus gallu	Kazufumi Shim	Institute of Trop	S,Rehman;F/		Institute of T	2022-03-05
EPI_ISL_1363	A/chicken/Kazakhstan/23/2020	A / H5N8	Asia / Kazakhst	Gallus gallu	Kairat Tabynov	Kazakh Nationa	Kaissar Taby		Aikimbayev	2020-08-01
EPI_ISL_1311	A/Chicken/BC/FAV-0348-OS/2022	A / H5N1	North America	Gallus gallu	Yohannes Berh	Canadian Food	Hisanaga, Ta		Animal Healt	2022-05-05
EPI_ISL_1311	A/Chicken/BC/FAV-0346-OS/2022	A / H5N1	North America	Gallus gallu	Yohannes Berh	Canadian Food	Hisanaga, Ta		Animal Healt	2022-04-29
EPI_ISL_1296	A/chicken/NL/FAV-0033/2021	A / H5N1	North America	Gallus gallu	Tamiko Hisana	Canadian Food	Hisanaga,Tar		Canadian Fo	2021-12-21
EPI_ISL_1296	A/Fancy chicken/NL/FAV-0035/2021	A / H5N1	North America	Gallus gallu	Tamiko Hisana	Canadian Food	Hisanaga,Ta		Canadian Fo	2021-12-17
EPI_ISL_1188	A/Gallus_gallus/Belgium/4190_0002/2022	A / H5N1	Europe / Belgiu	Gallus gallu	Steven Van Bo	Sciensano, De	Van Borm, St		Sciensano -	2022-03-28
EPI_ISL_9250	A/Gallus_gallus/Belgium/11372_0001/2021	A / H5N8	Europe / Belgiu	Gallus gallu	Steven Van Bo	Sciensano, De	Van Borm, St		Sciensano -	2021-08-31
EPI_ISL_9012	A/chicken/Scotland/054477/2021	A / H5N1	Europe / Unite	Gallus gallu	Alex Byrne (An	Animal and Pla			Animal and F	2021-11-01
EPI_ISL_9012	A/chicken/Wales/053969/2021	A / H5N1	Europe / Unite	Gallus gallu	Alex Byrne (An	Animal and Pla			Animal and F	2021-10-30
EPI_ISL_9012	A/chicken/England/053052/2021	A / H5N1	Europe / Unite	Gallus gallu	Alex Byrne (An	Animal and Pla			Animal and F	2021-10-24
EPI_ISL_6931	A/chicken/Poland/H1940-N/2021	A / H5N1	Europe / Polan	Gallus gallu	Edyta Świętoń	National Veteri	E. Swieton, K		National Vet	2021-11-05
EPI_ISL_6914	A/chicken/chongqing/H1/2021	A / H5N6	Asia / China / C	Gallus gallu	Wenming Jiang	China Animal H			China Anima	2021-06-10
EPI_ISL_2131	A/Chicken/Sweden/SVA210420SZ0002/KN09	A / H5N8	Europe / Swed	Gallus gallu	Siamak Zohari	National Veteri			National Vet	2021-04-20
EPI_ISL_1697	A/chicken/Czech Republic/5903/2021	A / H5N8	Europe / Czech	Gallus gallu	Alexander Nag	State Veterinar	Nagy,A;Cerni		State Veterir	2021-03-25
EPI_ISL_1697	A/chicken/Czech Republic/6151-1/2021	A / H5N8	Europe / Czech	Gallus gallu	Alexander Nag	State Veterinar	Nagy,A;Cerni		State Veterir	2021-03-29
EPI_ISL_1386	A/Chiken/Sweden/SVA210321SZ0001/KN073	A / H5N8	Europe / Swed	Gallus gallu	Siamak Zohari	National Veteri			National Vet	2021-03-21
EPI_ISL_1385	A/Chicken/Sweden/SVA210323SZ001/KN001	A / H5N5	Europe / Swed	Gallus gallu	Siamak Zohari	National Veteri			National Vet	2021-03-22
EPI_ISL_1303	A/Chicken/Sweden/SVA210313SZ0003/KN06	A / H5N8	Europe / Swed	Gallus gallu	Siamak Zohari	National Veteri			National Vet	2021-03-13
EPI_ISL_1303	A/Chichen/Sweden/SVA210313SZ0001/KN06	A / H5N8	Europe / Swed	Gallus gallu	Siamak Zohari	National Veteri			National Vet	2021-03-13
EPI_ISL_1114	A/chicken/Krasnodar/334-03/2021	A / H5N8	Europe / Russi	Gallus gallu	Natalia Goncha	State Research	Natalia,Gonc		State Resea	2021-01-05
EPI_ISL_1114	A/chicken/Krasnodar/334-02/2021	A / H5N8	Europe / Russi	Gallus gallu	Natalia Goncha	State Research	Natalia,Gonc		State Resea	2021-01-05
EPI_ISL_1114	A/chicken/Krasnodar/334-02/2021	A / H5N8	Europe / Russi	Gallus gallu	Natalia Goncha	State Research	Natalia,Gonc		State Resea	2021-01-05
EPI_ISL_1114	A/chicken/Krasnodar/334-01/2021	A / H5N8	Europe / Russi	Gallus gallu	Natalia Goncha	State Research	Natalia,Gonc		State Resea	2021-01-05
EPI_ISL_1114	A/chicken/Rostov-on-Don/308-04/2020	A / H5N8	Europe / Russi	Gallus gallu	Natalia Goncha	State Research	Natalia,Gonc		State Resea	2020-10-25
EPI_ISL_1114	A/chicken/Rostov-on-Don/308-03/2020	A / H5N8	Europe / Russi	Gallus gallu	Natalia Goncha	State Research	Natalia,Gonc		State Resea	2020-10-25
EPI_ISL_1114	A/chicken/Rostov-on-Don/308-02/2020	A / H5N8	Europe / Russi	Gallus gallu	Natalia Goncha	State Research	Natalia,Gonc		State Resea	2020-10-25
EPI_ISL_1114	A/chicken/Kostroma/304-10/2020	A / H5N8	Europe / Russi	Gallus gallu	Natalia Goncha	State Research	Natalia,Gonc		State Resea	2020-10-17
EPI_ISL_1114	A/chicken/Kostroma/304-08/2020	A / H5N8	Europe / Russi	Gallus gallu	Natalia Goncha	State Research	Natalia,Gonc		State Resea	2020-10-17

EPI_ISL_1114	A/chicken/Kostroma/304-06/2020	A / H5N8	Europe / Russi	Gallus gall	Natalia Goncha	State Research	Natalia,Goncl	State Resea	2020-10-17
EPI_ISL_1114	A/chicken/Kostroma/304-04/2020	A / H5N8	Europe / Russi	Gallus gall	Natalia Goncha	State Research	Natalia,Goncl	State Resea	2020-10-17
EPI_ISL_1114	A/chicken/Kostroma/304-03/2020	A / H5N8	Europe / Russi	Gallus gall	Natalia Goncha	State Research	Natalia,Goncl	State Resea	2020-10-17
EPI_ISL_1114	A/chicken/Kostroma/304-01/2020	A / H5N8	Europe / Russi	Gallus gall	Natalia Goncha	State Research	Natalia,Goncl	State Resea	2020-10-17
EPI_ISL_1114	A/chicken/Tyumen/302-02/2020	A / H5N8	Europe / Russi	Gallus gall	Natalia Goncha	State Research	Natalia,Goncl	State Resea	2020-09-26
EPI_ISL_1114	A/chicken/Tyumen/302-01/2020	A / H5N8	Europe / Russi	Gallus gall	Natalia Goncha	State Research	Natalia,Goncl	State Resea	2020-09-26
EPI_ISL_1058	A/chicken/Czech Republic/3099-2/2021	A / H5N8	Europe / Czech	Gallus gall	Alexander Nag	State Veterinar	Nagy,A; Cern	State Veterir	2021-02-12
EPI_ISL_1058	A/chicken/Czech Republic/29392021	A / H5N8	Europe / Czech	Gallus gall	Alexander Nag	State Veterinar		State Veterir	2021-02-10
EPI_ISL_1033	A/chicken/Czech Republic/2502-2/2021	A / H5N8	Europe / Czech	Gallus gall	Alexander Nag	State Veterinar	Nagy,A; Cern	State Veterir	2021-02-03
EPI_ISL_1033	A/chicken/Czech Republic/2502-1/2021	A / H5N8	Europe / Czech	Gallus gall	Alexander Nag	State Veterinar	Nagy,A; Cern	State Veterir	2021-02-03
EPI_ISL_1033	A/chicken/Czech Republic/2395/2021	A / H5N8	Europe / Czech	Gallus gall	Alexander Nag	State Veterinar	Nagy,A; Cern	State Veterir	2021-02-02
EPI_ISL_9435	A/Chicken/Sweden/SVA210117SZ0004/KN01	A / H5N5	Europe / Swed	Gallus gall	Siamak Zohari	National Veteri		National Vet	2021-01-16
EPI_ISL_9435	A/Chicken/Sweden/SVA210121SZ0033/KN00	A / H5N5	Europe / Swed	Gallus gall	Siamak Zohari	National Veteri		National Vet	2021-01-20
EPI_ISL_5254	A/chicken/Poland/004/2020	A / H5N8	Europe / Polan	Gallus gall	Edyta Świętoń	National Veteri	E. Swieton, K	National Vet	2020-01-02
EPI_ISL_5254	A/chicken/Poland/003/2020	A / H5N8	Europe / Polan	Gallus gall	Edyta Świętoń	National Veteri	E. Swieton, K	National Vet	2020-01-02
EPI_ISL_5254	A/laying_hen/Poland/002/2020	A / H5N8	Europe / Polan	Gallus gall	Edyta Świętoń	National Veteri	E. Swieton, K	National Vet	2020-01-01
EPI_ISL_1296	A/domestic goose/NL/FAV-0035-17/2021	A / H5N1	North America	Domestic g	Tamiko Hisana	Canadian Food	Hisanaga,Tar	Canadian Fo	2021-12-17
EPI_ISL_6931	A/domestic_goose/Poland/H1931-T1/2021	A / H5N1	Europe / Polan	Domestic g	Edyta Świętoń	National Veteri	E. Swieton, K	National Vet	2021-11-03
EPI_ISL_5865	A/green-winged-teal/Georgia/DT-22246/2020	A / H5N8	Asia / Georgia	Green-wing	Nicola Lewis (F	Royal Veterinar	Fouchier, R.A	Erasmus Me	2020-09-29
EPI_ISL_5260	A/green-winged teal/Guangdong/SD004/2021	A / H5N8	Asia / China / G	Green-wing	Pengfei Cui (Ha	Harbin Veterina	Pengfei Cui,	Harbin Veter	2021-01-20
EPI_ISL_1483	A/Whooper swan/Sanmenxia/B312/2020	A / H5N8	Asia / China / H	Whooper s	Zeyu Yang (Ch	Chinese Acade		Xi'an Tianlon	2020-12-06
EPI_ISL_1483	A/Whooper swan/Sanmenxia/B560/2020	A / H5N8	Asia / China / H	Whooper s	Zeyu Yang (Ch	Chinese Acade		Xi'an Tianlon	2020-12-01
EPI_ISL_1483	A/Whooper swan/Sanmenxia/Y25/2020	A / H5N8	Asia / China / H	Whooper s	Zeyu Yang (Ch	Chinese Acade		Xi'an Tianlon	2020-11-18
EPI_ISL_1483	A/Whooper swan/Sanmenxia/G25/2020	A / H5N8	Asia / China / H	Whooper s	Zeyu Yang (Ch	Chinese Acade		Xi'an Tianlon	2020-11-18
EPI_ISL_1483	A/Whooper swan/Sanmenxia/Y49/2020	A / H5N8	Asia / China / H	Whooper s	Zeyu Yang (Ch	Chinese Acade		Xi'an Tianlon	2020-11-17
EPI_ISL_1483	A/Whooper swan/Sanmenxia/Y48B/2020	A / H5N8	Asia / China / H	Whooper s	Zeyu Yang (Ch	Chinese Acade		Xi'an Tianlon	2020-11-17
EPI_ISL_1483	A/Whooper swan/Sanmenxia/G48B/2020	A / H5N8	Asia / China / H	Whooper s	Zeyu Yang (Ch	Chinese Acade		Xi'an Tianlon	2020-11-17
EPI_ISL_1483	A/Whooper swan/Sanmenxia/Y57/2020	A / H5N8	Asia / China / H	Whooper s	Zeyu Yang (Ch	Chinese Acade		Xi'an Tianlon	2020-11-16
EPI_ISL_1483	A/Whooper swan/Sanmenxia/Y54/2020	A / H5N8	Asia / China / H	Whooper s	Zeyu Yang (Ch	Chinese Acade		Xi'an Tianlon	2020-11-16
EPI_ISL_1483	A/Whooper swan/Sanmenxia/Y48A/2020	A / H5N8	Asia / China / H	Whooper s	Zeyu Yang (Ch	Chinese Acade		Xi'an Tianlon	2020-11-16
EPI_ISL_1483	A/Whooper swan/Sanmenxia/Y36/2020	A / H5N8	Asia / China / H	Whooper s	Zeyu Yang (Ch	Chinese Acade		Xi'an Tianlon	2020-11-16
EPI_ISL_1483	A/Whooper swan/Sanmenxia/Y31/2020	A / H5N8	Asia / China / H	Whooper s	Zeyu Yang (Ch	Chinese Acade		Xi'an Tianlon	2020-11-16
EPI_ISL_1483	A/Whooper swan/Sanmenxia/Y27/2020	A / H5N8	Asia / China / H	Whooper s	Zeyu Yang (Ch	Chinese Acade		Xi'an Tianlon	2020-11-16
EPI_ISL_1483	A/Whooper swan/Sanmenxia/Y26/2020	A / H5N8	Asia / China / H	Whooper s	Zeyu Yang (Ch	Chinese Acade		Xi'an Tianlon	2020-11-16
EPI_ISL_1483	A/Whooper swan/Sanmenxia/G29/2020	A / H5N8	Asia / China / H	Whooper s	Zeyu Yang (Ch	Chinese Acade		Xi'an Tianlon	2020-11-16
EPI_ISL_1483	A/Whooper swan/Sanmenxia/G28/2020	A / H5N8	Asia / China / H	Whooper s	Zeyu Yang (Ch	Chinese Acade		Xi'an Tianlon	2020-11-16
EPI_ISL_1483	A/Whooper swan/Sanmenxia/G27/2020	A / H5N8	Asia / China / H	Whooper s	Zeyu Yang (Ch	Chinese Acade		Xi'an Tianlon	2020-11-16
EPI_ISL_1483	A/Whooper swan/Sanmenxia/Y23-2/2020	A / H5N8	Asia / China / H	Whooper s	Zeyu Yang (Ch	Chinese Acade		Xi'an Tianlon	2020-11-14
EPI_ISL_1483	A/Whooper swan/Sanmenxia/Y23-1/2020	A / H5N8	Asia / China / H	Whooper s	Zeyu Yang (Ch	Chinese Acade		Xi'an Tianlon	2020-11-14
EPI_ISL_1483	A/Whooper swan/Sanmenxia/Y21/2020	A / H5N8	Asia / China / H	Whooper s	Zeyu Yang (Ch	Chinese Acade		Xi'an Tianlon	2020-11-14
EPI_ISL_1483	A/Whooper swan/Sanmenxia/Y20/2020	A / H5N8	Asia / China / H	Whooper s	Zeyu Yang (Ch	Chinese Acade		Xi'an Tianlon	2020-11-14
EPI_ISL_1483	A/Whooper swan/Sanmenxia/Y19/2020	A / H5N8	Asia / China / H	Whooper s	Zeyu Yang (Ch	Chinese Acade		Xi'an Tianlon	2020-11-14

EPI_ISL_14835	A/Whooper swan/Sanmenxia/Y56/2020	A / H5N8	Asia / China / H	Whooper sw	Zeyu Yang (Ch	Chinese Acade		Xi'an Tianlon	2020-11-13
EPI_ISL_14835	A/Whooper swan/Sanmenxia/Y18/2020	A / H5N8	Asia / China / H	Whooper sw	Zeyu Yang (Ch	Chinese Acade		Xi'an Tianlon	2020-11-13
EPI_ISL_14835	A/Whooper swan/Sanmenxia/Y24/2020	A / H5N8	Asia / China / H	Whooper sw	Zeyu Yang (Ch	Chinese Acade		Xi'an Tianlon	2020-11-12
EPI_ISL_14835	A/Whooper swan/Sanmenxia/H810/2020	A / H5N8	Asia / China / H	Whooper sw	Zeyu Yang (Ch	Chinese Acade		Xi'an Tianlon	2020-11-11
EPI_ISL_14835	A/Whooper swan/Sanmenxia/H735/2020	A / H5N8	Asia / China / H	Whooper sw	Zeyu Yang (Ch	Chinese Acade		Xi'an Tianlon	2020-11-11
EPI_ISL_14835	A/Whooper swan/Sanmenxia/H615/2020	A / H5N8	Asia / China / H	Whooper sw	Zeyu Yang (Ch	Chinese Acade		Xi'an Tianlon	2020-11-11
EPI_ISL_14835	A/Whooper swan/Sanmenxia/H1/2020	A / H5N8	Asia / China / H	Whooper sw	Zeyu Yang (Ch	Chinese Acade		Xi'an Tianlon	2020-11-11
EPI_ISL_52604	A/whooper swan/Shanxi/4-2/2020	A / H5N8	Asia / China / S	Whooper sw	Pengfei Cui (Ha	Harbin Veterina	Pengfei Cui, C	Harbin Veter	2020-11-10
EPI_ISL_52604	A/whooper swan/Shanxi/4-1/2020	A / H5N8	Asia / China / S	Whooper sw	Pengfei Cui (Ha	Harbin Veterina	Pengfei Cui, C	Harbin Veter	2020-11-10
EPI_ISL_52604	A/whooper swan/Shandong/SC188/2021	A / H5N8	Asia / China / S	Whooper sw	Pengfei Cui (Ha	Harbin Veterina	Pengfei Cui, C	Harbin Veter	2021-02-22
EPI_ISL_52604	A/whooper swan/Shandong/SC200/2021	A / H5N8	Asia / China / S	Whooper sw	Pengfei Cui (Ha	Harbin Veterina	Pengfei Cui, C	Harbin Veter	2021-01-23
EPI_ISL_52604	A/whooper swan/Shandong/SC199/2021	A / H5N8	Asia / China / S	Whooper sw	Pengfei Cui (Ha	Harbin Veterina	Pengfei Cui, C	Harbin Veter	2021-01-23
EPI_ISL_52604	A/whooper swan/Shandong/SC198/2021	A / H5N8	Asia / China / S	Whooper sw	Pengfei Cui (Ha	Harbin Veterina	Pengfei Cui, C	Harbin Veter	2021-01-23
EPI_ISL_52604	A/whooper swan/Shandong/SC195/2021	A / H5N8	Asia / China / S	Whooper sw	Pengfei Cui (Ha	Harbin Veterina	Pengfei Cui, C	Harbin Veter	2021-01-23
EPI_ISL_52604	A/whooper swan/Shandong/SC185/2021	A / H5N8	Asia / China / S	Whooper sw	Pengfei Cui (Ha	Harbin Veterina	Pengfei Cui, C	Harbin Veter	2021-02-22
EPI_ISL_52604	A/whooper swan/Shandong/SC176/2021	A / H5N8	Asia / China / S	Whooper sw	Pengfei Cui (Ha	Harbin Veterina	Pengfei Cui, C	Harbin Veter	2021-02-22
EPI_ISL_31386	A/whooper swan/Sweden/SVA210420SZ0485/	A / H5N8	Europe / Swede	Whooper sw	Siamak Zohari	National Veterin		National Vete	2021-04-15
EPI_ISL_14917	A/black-headed_gull/Poland/MB139/2022	A / H5N1	Europe / Poland	Black-head	Edyta Świętoń (	National Veterin	Swieton E., S	National Vete	2022-05-30
EPI_ISL_91602	A/Anser_albifrons/Belgium/15465_0010/2021	A / H5N1	Europe / Belgiu	White-front	Steven Van Bor	Sciensano, Dep	Van Borm, St	Sciensano -	2021-11-21
EPI_ISL_14917	A/herring_gull/Poland/MB138/2022	A / H5N1	Europe / Poland	Herring gull	Edyta Świętoń (	National Veterin	Swieton E., S	National Vete	2022-05-30
EPI_ISL_70547	A/European herring gull/Sweden/SVA211116S	A / H5N1	Europe / Swede	Herring gull	Siamak Zohari	National Veterin		National Vete	2021-11-08
EPI_ISL_11391	A/common snipe/Netherlands/20018931-003/2	A / H5N8	Europe / Nethe	Gallinago g	Rene Heutink ('	Wageningen Bi	Beerens, Nar	Wageningen	2020-11-26
EPI_ISL_33193	A/common eider/Sweden/SVA210729SZ0320/	A / H5N8	Europe / Swede	Somateria r	Siamak Zohari	National Veterin		National Vete	2021-07-19
EPI_ISL_40717	A/Common pochard/Bangladesh/42386/2020	A / H5N6	Asia / Banglade	Aythya ferir			Barman,S.; T		2020-01-20
EPI_ISL_63231	A/eurasian teal/Netherlands/20016896-013/202	A / H5N1	Europe / Nethe	Common te	Rene Heutink ('	Wageningen Bi	Beerens, Nar	Wageningen	2020-11-02
EPI_ISL_75954	A/Anas_platyrhynchos/Belgium/10413_0003/20	A / H5N2	Europe / Belgiu	Mallard duc	Steven Van Bor	Sciensano, Dep	Van Borm, St	Sciensano -	2020-10-04
EPI_ISL_11390	A/egyptian goose/Netherlands/20017027-002/2	A / H5N8	Europe / Nethe	Egyptian go	Rene Heutink ('	Wageningen Bi	Beerens, Nar	Wageningen	2020-11-03
EPI_ISL_14835	A/Bean Goose(Anser fabalis)/South Korea/H5N	A / H5N8	Asia / Korea, R	Bean goose			Tran,K.N.T.; F		2021-03-10
EPI_ISL_50955	A/bean goose/Germany-BB/AI00444/2021	A / H5N8	Europe / Germa	Bean goose	Jacqueline King	Friedrich-Loeffle		Landeslabor	2020-12-21
EPI_ISL_27133	A/bean goose/Hubei/BQ11/2020	A / H5N8	Asia / China / H	Bean goose	Hongliang Chai	Northeast Fores		College of W	2020-11-16
EPI_ISL_69305	A/turkey/Poland/H1924-T1/2021	A / H5N1	Europe / Poland	Meleagris g	Edyta Świętoń (	National Veterin	E. Swieton, K	National Vete	2021-11-03
EPI_ISL_11147	A/turkey/Rostov-on-Don/332-12/2021	A / H5N8	Europe / Russia	Meleagris g	Natalia Goncha	State Research	Natalia,Gonch	State Resear	2021-01-29
EPI_ISL_11147	A/turkey/Rostov-on-Don/332-10/2021	A / H5N8	Europe / Russia	Meleagris g	Natalia Goncha	State Research	Natalia,Gonch	State Resear	2021-01-29
EPI_ISL_11147	A/turkey/Rostov-on-Don/332-09/2021	A / H5N8	Europe / Russia	Meleagris g	Natalia Goncha	State Research	Natalia,Gonch	State Resear	2021-01-29
EPI_ISL_11147	A/turkey/Rostov-on-Don/332-08/2021	A / H5N8	Europe / Russia	Meleagris g	Natalia Goncha	State Research	Natalia,Gonch	State Resear	2021-01-29
EPI_ISL_11147	A/turkey/Rostov-on-Don/332-08/2021	A / H5N8	Europe / Russia	Meleagris g	Natalia Goncha	State Research	Natalia,Gonch	State Resear	2021-01-29
EPI_ISL_11147	A/turkey/Stavropol/320-03/2020	A / H5N8	Europe / Russia	Meleagris g	Natalia Goncha	State Research	Natalia,Gonch	State Resear	2020-12-11
EPI_ISL_11147	A/turkey/Stavropol/320-02/2020	A / H5N8	Europe / Russia	Meleagris g	Natalia Goncha	State Research	Natalia,Gonch	State Resear	2020-12-11
EPI_ISL_11147	A/turkey/Stavropol/320-01/2020	A / H5N8	Europe / Russia	Meleagris g	Natalia Goncha	State Research	Natalia,Gonch	State Resear	2020-12-11
EPI_ISL_77912	A/turkey/Poland/464/2020(H5N8)	A / H5N8	Europe / Poland	Meleagris g	Edyta Świętoń (	National Veterin	Swieton E., S	National Vete	2020-12-01
EPI_ISL_64796	A/Turkey/Sweden/SVA201114SZ0001/20KN30	A / H5N8	Europe / Swede	Meleagris g	Siamak Zohari	National Veterin		National Vete	2020-11-13
EPI_ISL_41826	A/turkey/Czech Republic/3071/2020	A / H5N8	Europe / Czech	Meleagris g	Alexander Nagy	State Veterinar	Nagy,A	State Veterin	2020-02-17

EPI_ISL_63751	A/waterfowl/Netherlands/21037914-006/2021	A / H5N1	Europe / Nethe	Wild waterf	Rene Heutink (\	Wageningen Bi	Beerens, Nar	Wageningen	2021-11-02
EPI_ISL_52604	A/wild duck/Shandong/SC177/2021	A / H5N8	Asia / China / S	Wild waterf	Pengfei Cui (Ha	Harbin Veterina	Cui, Pengfei	Harbin Veter	2021-02-22
EPI_ISL_63182	A/Mandarin duck/Korea/H242/2020	A / H5N8	Asia / Korea, R	Wild waterf	Yu-Na Lee (Ani	Animal and Pla		Animal and F	2020-10-21
EPI_ISL_12734	A/eastern buzzard/Tochigi/090311T/2021	A / H5N8	Asia / Japan / T	Falcon	Takehiko Saito	National Institut		National Inst	2021-03-03
EPI_ISL_50578	A/peregrine falcon/Germany-SH/AI02162/2020	A / H5N8	Europe / Germa	Falco pereg	Jacqueline King	Friedrich-Loeffl		Landeslabor	2020-10-26
EPI_ISL_30987	A/peregrine falcon/Sweden/SVA210309SZ040	A / H5N4	Europe / Swede	Falco pereg	Siamak Zohari	National Veterin		National Vet	2021-03-02
EPI_ISL_21733	A/peregrine falcon/Netherlands/21025108-001/	A / H5N4	Europe / Nethe	Falco pereg	Rene Heutink (\	Wageningen Bi	Beerens, Nar	Wageningen	2021-03-10
EPI_ISL_50981	A/kestrel/Germany-NI/AI03672/2020	A / H5N8	Europe / Germa	Falco tinnu	Jacqueline King	Friedrich-Loeffl		Lebensmittel	2020-12-21
EPI_ISL_14823	A/Wild geese/Hubei/H68/2021	A / H5N8	Asia / China / H	Goose	Zeyu Yang (Ch	Chinese Acade		Xi'an Tianlon	2021-01-27
EPI_ISL_14823	A/Wild geese/Hubei/H549/2021	A / H5N8	Asia / China / H	Goose	Zeyu Yang (Ch	Chinese Acade		Xi'an Tianlon	2021-01-27
EPI_ISL_14823	A/Wild geese/Hubei/H418/2021	A / H5N8	Asia / China / H	Goose	Zeyu Yang (Ch	Chinese Acade		Xi'an Tianlon	2021-01-27
EPI_ISL_14823	A/Wild geese/Hubei/H358/2021	A / H5N8	Asia / China / H	Goose	Zeyu Yang (Ch	Chinese Acade		Xi'an Tianlon	2021-01-27
EPI_ISL_11971	A/domestic goose/Michigan/22-008890-004-ori	A / H5N1	North America /	Goose	Mary Lea Killian	National Veterin	Chinh,Thanh;	National Vet	2022-03-22
EPI_ISL_11922	A/goose/Poland/H124_22VIR2515-5/2022	A / H5N1	Europe / Poland	Goose	Giacomo Barbi	Istituto Zooprof	Swieton, E.; S	National Vet	2022-02-05
EPI_ISL_11259	A/goose/Spain/294-2_22VIR2142-9/2022	A / H5N1	Europe / Spain	Goose	Giacomo Barbi	Istituto Zooprof	Ruano, M.J.;	Laboratorio C	2022-01-26
EPI_ISL_11259	A/goose/Spain/239-1_22VIR2142-8/2022	A / H5N1	Europe / Spain	Goose	Giacomo Barbi	Istituto Zooprof	Ruano, M.J.;	Laboratorio C	2022-01-22
EPI_ISL_11259	A/goose/Spain/141-9_22VIR2142-6/2022	A / H5N1	Europe / Spain	Goose	Giacomo Barbi	Istituto Zooprof	Ruano, M.J.;	Laboratorio C	2022-01-14
EPI_ISL_11259	A/goose/Spain/88-3_22VIR2142-4/2022	A / H5N1	Europe / Spain	Goose	Giacomo Barbi	Istituto Zooprof	Ruano, M.J.;	Laboratorio C	2022-01-10
EPI_ISL_11259	A/goose/Spain/65-3_22VIR2142-2/2022	A / H5N1	Europe / Spain	Goose	Giacomo Barbi	Istituto Zooprof	Ruano, M.J.;	Laboratorio C	2022-01-07
EPI_ISL_11259	A/goose/Spain/512-2_22VIR2142-13/2022	A / H5N1	Europe / Spain	Goose	Giacomo Barbi	Istituto Zooprof	Ruano, M.J.;	Laboratorio C	2022-02-04
EPI_ISL_11259	A/goose/Ireland/3869_22VIR2064-2/2022	A / H5N1	Europe / Ireland	Goose	Giacomo Barbi	Istituto Zooprof	Byrne, C.; Ga	Central Vete	2022-02-14
EPI_ISL_96039	A/goose/Czech Republic/22750/2021	A / H5N1	Europe / Czech	Goose	Alexander Nagy	State Veterinar	Alexander,Na	State Veterir	2021-11-19
EPI_ISL_96039	A/goose/Czech Republic/22608-3T/2021	A / H5N1	Europe / Czech	Goose	Alexander Nagy	State Veterinar	Alexander,Na	State Veterir	2021-11-18
EPI_ISL_96038	A/goose/Czech Republic/22608-2T/2021	A / H5N1	Europe / Czech	Goose	Alexander Nagy	State Veterinar	Alexander,Na	State Veterir	2021-11-18
EPI_ISL_96037	A/goose/Czech Republic/22608-1T/2021	A / H5N1	Europe / Czech	Goose	Alexander Nagy	State Veterinar	Alexander,Na	State Veterir	2021-11-18
EPI_ISL_96037	A/goose/Czech Republic/23458-1K/2021	A / H5N1	Europe / Czech	Goose	Alexander Nagy	State Veterinar	Alexander,Na	State Veterir	2021-11-26
EPI_ISL_96037	A/goose/Czech Republic/23458-4T/2021	A / H5N1	Europe / Czech	Goose	Alexander Nagy	State Veterinar	Alexander,Na	State Veterir	2021-11-26
EPI_ISL_96037	A/goose/Czech Republic/23458-5T/2021	A / H5N1	Europe / Czech	Goose	Alexander Nagy	State Veterinar	Alexander,Na	State Veterir	2021-11-26
EPI_ISL_96037	A/goose/Czech Republic/23458-2T/2021	A / H5N1	Europe / Czech	Goose	Alexander Nagy	State Veterinar	Alexander,Na	State Veterir	2021-11-26
EPI_ISL_77534	A/greylag goose/Germany-SH/AI06205/2021	A / H5N1	Europe / Germa	Goose	Jacqueline King	Friedrich-Loeffl		Landeslabor	2021-10-25
EPI_ISL_77534	A/greylag goose/Germany-SH/AI06144/2021	A / H5N1	Europe / Germa	Goose	Jacqueline King	Friedrich-Loeffl		Landeslabor	2021-10-25
EPI_ISL_77534	A/domestic goose/Germany-SH/AI06024/2021	A / H5N1	Europe / Germa	Goose	Jacqueline King	Friedrich-Loeffl		Landeslabor	2021-10-22
EPI_ISL_77533	A/domestic goose/Germany-SH/AI06150/2021	A / H5N1	Europe / Germa	Goose	Jacqueline King	Friedrich-Loeffl		Landeslabor	2021-10-22
EPI_ISL_77533	A/domestic goose/Germany-SH/AI06147/2021	A / H5N1	Europe / Germa	Goose	Jacqueline King	Friedrich-Loeffl		Landeslabor	2021-10-22
EPI_ISL_77533	A/lesser white-fronted goose/Germany-MV/AI0	A / H5N1	Europe / Germa	Goose	Jacqueline King	Friedrich-Loeffl		Landesamt f	2021-10-20
EPI_ISL_77532	A/barnacle goose/Germany-SH/AI06005/2021	A / H5N1	Europe / Germa	Goose	Jacqueline King	Friedrich-Loeffl		Landeslabor	2021-10-19
EPI_ISL_67606	A/Goose/Guangdong/211057/2021(H5N6)	A / H5N6	Asia / China / G	Goose	Jiahao Zhang (	South China Ag	Zhang, Jiahao	South China	2021-08
EPI_ISL_51235	A/domestic goose/Germany-NI/AI03093/2021	A / H5N8	Europe / Germa	Goose	Jacqueline King	Friedrich-Loeffl		Lebensmittel	2021-03-18
EPI_ISL_51156	A/domestic goose/Germany-SH/AI02102/2021	A / H5N8	Europe / Germa	Goose	Jacqueline King	Friedrich-Loeffl		Landeslabor	2021-03-04
EPI_ISL_51156	A/domestic goose/Germany-SH/AI02100/2021	A / H5N8	Europe / Germa	Goose	Jacqueline King	Friedrich-Loeffl		Landeslabor	2021-03-04
EPI_ISL_50981	A/wild goose/Germany-NI/AI03101/2020	A / H5N8	Europe / Germa	Goose	Jacqueline King	Friedrich-Loeffl		Lebensmittel	2020-11-19
EPI_ISL_50981	A/wild goose/Germany-NI/AI03471/2020	A / H5N8	Europe / Germa	Goose	Jacqueline King	Friedrich-Loeffl		Lebensmittel	2020-12-14

EPI_ISL_50981	A/pomeranian goose/Germany-MV/AI01100/20	A / H5N8	Europe / Germa	Goose	Jacqueline King	Friedrich-Loeffle		Landesamt f	2021-02-01
EPI_ISL_50981	A/Hawaiian goose/Germany-RP/AI00856/2021	A / H5N8	Europe / Germa	Goose	Jacqueline King	Friedrich-Loeffle		CVUA Karlsr	2021-01-18
EPI_ISL_50981	A/wild goose/Germany-NI/AI00854/2021	A / H5N8	Europe / Germa	Goose	Jacqueline King	Friedrich-Loeffle		Lebensmittel	2021-01-18
EPI_ISL_50981	A/wild goose/Germany-NI/AI00626/2021	A / H5N8	Europe / Germa	Goose	Jacqueline King	Friedrich-Loeffle		Lebensmittel	2021-01-11
EPI_ISL_16652	A/geese/Romania/10206_21VIR849-3/2021	A / H5N8	Europe / Roma	Goose	Bianca Zecchin	Istituto Zooprof	Onita, I.; Neic	Istituto Zoopr	2021-01-13
EPI_ISL_84662	A/wild_goose/Poland/MB142/2020(H5N8)	A / H5N8	Europe / Polan	Goose	Edyta Świętoń	National Veterin	Swieton, E.; S	National Vet	2020-12-15
EPI_ISL_84658	A/Goose/Hungary/19128/2020	A / H5N8	Europe / Hunga	Goose	Katalin Szentpá	National Food	Katalin,Szent	National Foo	2020-04-17
EPI_ISL_81397	A/Goose/Hungary/24021/2020	A / H5N8	Europe / Hunga	Goose	Katalin Szentpá	National Food	Katalin,Szent	National Foo	2020-05-11
EPI_ISL_81359	A/Goose/Hungary/22493/2020	A / H5N8	Europe / Hunga	Goose	Katalin Szentpá	National Food	Katalin,Szent	National Foo	2020-05-04
EPI_ISL_73968	A/goose/Russia_Omsk region/55-1/2020	A / H5N8	Europe / Russia	Goose	Ivan Sobolev (F	WHO National	Sobolev, I.; S	Research Ins	2020-08-29
EPI_ISL_73968	A/goose/Russia_Novosibirsk region/1-12/2020	A / H5N8	Europe / Russia	Goose	Ivan Sobolev (F	WHO National	Sobolev, I.; S	Research Ins	2020-09-15
EPI_ISL_12069	A/greylag goose/Netherlands/22006190-002/20	A / H5N1	Europe / Nethe	Anser anse	Rene Heutink ('	Wageningen Bi	Beerens, Nan	Wageningen	2022-03-28
EPI_ISL_90299	A/Greylag_goose/England/054503/2021	A / H5N1	Europe / United	Anser anse	Alex Byrne (Ani	Animal and Pla		Animal and F	2021-10-30
EPI_ISL_67610	A/greylag goose/Netherlands/21037809-001/20	A / H5N1	Europe / Nethe	Anser anse	Rene Heutink ('	Wageningen Bi	Beerens, Nan	Wageningen	2021-10-31
EPI_ISL_63280	A/greylag goose/Netherlands/21037497-001/20	A / H5N1	Europe / Nethe	Anser anse	Rene Heutink ('	Wageningen Bi	Beerens, Nan	Wageningen	2021-10-19
EPI_ISL_14937	A/snow goose/Kansas/W22-199C/2022	A / H5N1	North America	Anser caeru			Poulson,R.; S		2022-03-12
EPI_ISL_14937	A/snow goose/Kansas/W22-199E/2022	A / H5N1	North America	Anser caeru			Poulson,R.; S		2022-03-12
EPI_ISL_14937	A/snow goose/Kansas/W22-199B/2022	A / H5N1	North America	Anser caeru			Poulson,R.; S		2022-03-12
EPI_ISL_14937	A/snow goose/Kansas/W22-199A/2022	A / H5N1	North America	Anser caeru			Poulson,R.; S		2022-03-14
EPI_ISL_14937	A/snow goose/Kansas/W22-199F/2022	A / H5N1	North America	Anser caeru			Poulson,R.; S		2022-03-12
EPI_ISL_14937	A/snow goose/Kansas/W22-199D/2022	A / H5N1	North America	Anser caeru			Poulson,R.; S		2022-03-12
EPI_ISL_14822	A/bar_headed_goose/Tibet/T1707/2021	A / H5N8	Asia / China / T	Anser indic	Zeyu Yang (Ch	Chinese Acade		Xi'an Tianlon	2021-05-19
EPI_ISL_14822	A/bar_headed_goose/Tibet/T1640/2021	A / H5N8	Asia / China / T	Anser indic	Zeyu Yang (Ch	Chinese Acade		Xi'an Tianlon	2021-05-18
EPI_ISL_14822	A/Bar-headed Goose/Tibet/P1987/2021	A / H5N8	Asia / China / T	Anser indic	Zeyu Yang (Ch	Chinese Acade		Xi'an Tianlon	2021-03-12
EPI_ISL_14822	A/Bar-headed Goose/Tibet/P1910/2021	A / H5N8	Asia / China / T	Anser indic	Zeyu Yang (Ch	Chinese Acade		Xi'an Tianlon	2021-03-12
EPI_ISL_14822	A/Bar-headed Goose/Tibet/P1908/2021	A / H5N8	Asia / China / T	Anser indic	Zeyu Yang (Ch	Chinese Acade		Xi'an Tianlon	2021-03-12
EPI_ISL_14822	A/Bar-headed Goose/Tibet/P2320/2021	A / H5N8	Asia / China / T	Anser indic	Zeyu Yang (Ch	Chinese Acade		Xi'an Tianlon	2021-03-11
EPI_ISL_13519	A/gull/France/22P015977/2022	A / H5N1	Europe / France	Gull	Francois-Xavier	ANSES Agence		Anses (Plouf	2022-05-11
EPI_ISL_12512	A/Black-headed gull/Netherlands/2/2022	A / H5N1	Europe / Nethe	Gull	Sanne Thewess	Erasmus Medic		Erasmus Me	2022-04-06
EPI_ISL_77533	A/herring gull/Germany-SH/AI06141/2021	A / H5N1	Europe / Germa	Gull	Jacqueline King	Friedrich-Loeffle		Landeslabor	2021-10-21
EPI_ISL_50993	A/Laridae/Germany-SH/AI01498/2021	A / H5N4	Europe / Germa	Gull	Jacqueline King	Friedrich-Loeffle		Landeslabor	2021-02-17
EPI_ISL_14494	A/Larus_argentatus/Belgium/9013_0001/2022	A / H5N1	Europe / Belgiu	Larus argen	Steven Van Bo	Sciensano, Dep	Van Borm, St	Sciensano -	2022-07-08
EPI_ISL_14389	A/Larus_argentatus/Belgium/595_0008/2022	A / H5N1	Europe / Belgiu	Larus argen	Steven Van Bo	Sciensano, Dep	Van Borm, St	Sciensano -	2022-01-06
EPI_ISL_11798	A/European_herring_gull/Denmark/19968-1.02	A / H5N1	Europe / Denm	Larus argen	Charlotte Kristia	Statens Serum	Charlotte Hjul	Statens Seru	2021-05-14
EPI_ISL_50580	A/herring gull/Germany-HH/AI02182/2020	A / H5N8	Europe / Germa	Larus argen	Jacqueline King	Friedrich-Loeffle		Institut für Hy	2020-11-02
EPI_ISL_30260	A/European herring gull/Sweden/SVA210617S	A / H5N8	Europe / Swede	Larus argen	Siamak Zohari	National Veterin		National Vet	2021-06-17
EPI_ISL_12069	A/black-backed gull/Netherlands/22006192-00	A / H5N1	Europe / Nethe	Larus fuscu	Rene Heutink ('	Wageningen Bi	Beerens, Nan	Wageningen	2022-03-27
EPI_ISL_10934	A/black-headed gull/Sweden/SVA210216SZ03	A / H5N8	Europe / Swede	Larus ridibu	Siamak Zohari	National Veterin		National Vet	2021-02-07
EPI_ISL_11971	A/pheasant/New York/22-009066-001-original/	A / H5N1	North America	Pheasant	Mary Lea Killian	National Veterin	Chinh,Thanh;	National Vet	2022-03-25
EPI_ISL_11971	A/pheasant/New York/22-008760-008-original/	A / H5N1	North America	Pheasant	Mary Lea Killian	National Veterin	Chinh,Thanh;	National Vet	2022-03-22
EPI_ISL_96039	A/pheasant/Czech Republic/25827-3/2021	A / H5N1	Europe / Czech	Phasianus	Alexander Nagy	State Veterinar	Alexander,Na	State Veterir	2021-12-25
EPI_ISL_96039	A/pheasant/Czech Republic/25827-2/2021	A / H5N1	Europe / Czech	Phasianus	Alexander Nagy	State Veterinar	Alexander,Na	State Veterir	2021-12-25

EPI_ISL_96039	A/pheasant/Czech Republic/25827-1/2021	A / H5N1	Europe / Czech	Phasianus	Alexander Nagy	State Veterinar	Alexander,Na	State Veterir	2021-12-25
EPI_ISL_11922	A/swan/Romania/10986_22VIR2749-8/2022	A / H5N1	Europe / Roma	Swan	Giacomo Barbi	Istituto Zooprof	Barbuceanu,	Institute for D	2022-03-07
EPI_ISL_11922	A/swan/Romania/10678_22VIR2749-7/2022	A / H5N1	Europe / Roma	Swan	Giacomo Barbi	Istituto Zooprof	Barbuceanu,	Institute for D	2022-02-23
EPI_ISL_11922	A/swan/Romania/10656_22VIR2749-6/2022	A / H5N1	Europe / Roma	Swan	Giacomo Barbi	Istituto Zooprof	Barbuceanu,	Institute for D	2022-02-22
EPI_ISL_11922	A/swan/Romania/10455_22VIR2749-4/2022	A / H5N1	Europe / Roma	Swan	Giacomo Barbi	Istituto Zooprof	Barbuceanu,	Institute for D	2022-02-09
EPI_ISL_11922	A/swan/Romania/10394_22VIR2749-3/2022	A / H5N1	Europe / Roma	Swan	Giacomo Barbi	Istituto Zooprof	Barbuceanu,	Institute for D	2022-02-03
EPI_ISL_11922	A/swan/Romania/10324_22VIR2749-2/2022	A / H5N1	Europe / Roma	Swan	Giacomo Barbi	Istituto Zooprof	Barbuceanu,	Institute for D	2022-02-01
EPI_ISL_11922	A/swan/Romania/16905_22VIR2749-1/2021	A / H5N1	Europe / Roma	Swan	Giacomo Barbi	Istituto Zooprof	Barbuceanu,	Institute for D	2021-12-08
EPI_ISL_11922	A/swan/Poland/MB083_22VIR2515-8/2022	A / H5N1	Europe / Polan	Swan	Giacomo Barbi	Istituto Zooprof	Swieton, E.; S	National Vete	2022-02-17
EPI_ISL_11922	A/swan/Poland/MB078_22VIR2515-7/2022	A / H5N1	Europe / Polan	Swan	Giacomo Barbi	Istituto Zooprof	Swieton, E.; S	National Vete	2022-02-10
EPI_ISL_11259	A/swan/Spain/4087-1_22VIR2142-1/2021	A / H5N1	Europe / Spain	Swan	Giacomo Barbi	Istituto Zooprof	Ruano, M.J.;	Laboratorio C	2021-12-22
EPI_ISL_96039	A/mute swan/Czech Republic/25702-2/2021	A / H5N1	Europe / Czech	Swan	Alexander Nagy	State Veterinar	Alexander,Na	State Veterir	2021-12-20
EPI_ISL_96039	A/mute swan/Czech Republic/22684/2021	A / H5N1	Europe / Czech	Swan	Alexander Nagy	State Veterinar	Alexander,Na	State Veterir	2021-11-15
EPI_ISL_63280	A/swan/Netherlands/21037791-001/2021	A / H5N1	Europe / Nethe	Swan	Rene Heutink ('	Wageningen Bi	Beerens, Nar	Wageningen	2021-11-01
EPI_ISL_16652	A/whooper_swan/Romania/10311_21VIR849-2	A / H5N5	Europe / Roma	Swan	Bianca Zecchin	Istituto Zooprof	Onita, I.; Neic	Istituto Zoopr	2021-01-20
EPI_ISL_16652	A/whooper_swan/Romania/10123_21VIR849-1	A / H5N5	Europe / Roma	Swan	Bianca Zecchin	Istituto Zooprof	Onita, I.; Neic	Istituto Zoopr	2021-01-08
EPI_ISL_16652	A/mute_swan/Slovenia/1914-20_21VIR959-5/2	A / H5N5	Europe / Slover	Swan	Bianca Zecchin	Istituto Zooprof	Slavec, B.; Ra	Istituto Zoopr	2020-12-24
EPI_ISL_16652	A/mute_swan/Slovenia/1820-20_21VIR959-4/2	A / H5N8	Europe / Slover	Swan	Bianca Zecchin	Istituto Zooprof	Slavec, B.; Ra	Istituto Zoopr	2020-12-10
EPI_ISL_16652	A/mute_swan/Slovenia/1799-20_21VIR959-2/2	A / H5N8	Europe / Slover	Swan	Bianca Zecchin	Istituto Zooprof	Slavec, B.; Ra	Istituto Zoopr	2020-12-09
EPI_ISL_16652	A/mute_swan/Slovenia/1756-20_21VIR959-3/2	A / H5N8	Europe / Slover	Swan	Bianca Zecchin	Istituto Zooprof	Slavec, B.; Ra	Istituto Zoopr	2020-12-02
EPI_ISL_16652	A/mute_swan/Slovenia/1639-20_21VIR959-1/2	A / H5N8	Europe / Slover	Swan	Bianca Zecchin	Istituto Zooprof	Slavec, B.; Ra	Istituto Zoopr	2020-11-17
EPI_ISL_16652	A/mute_swan/Slovakia/Pah6_21VIR1086-2/20	A / H5N5	Europe / Slovak	Swan	Bianca Zecchin	Istituto Zooprof	Dirb?kov?, Z.	Istituto Zoopr	2021-01-15
EPI_ISL_16652	A/mute_swan/Slovakia/Pah15_21VIR1086-3/2	A / H5N5	Europe / Slovak	Swan	Bianca Zecchin	Istituto Zooprof	Dirb?kov?, Z.	Istituto Zoopr	2021-01-29
EPI_ISL_16652	A/mute_swan/Norway/FU5_21VIR850-3/2021	A / H5N8	Europe / Norwa	Swan	Bianca Zecchin	Istituto Zooprof	Madslien, K.;	Istituto Zoopr	2021-01-05
EPI_ISL_16652	A/mute_swan/Austria/21014124_21VIR1085-8	A / H5N8	Europe / Austria	Swan	Bianca Zecchin	Istituto Zooprof	Wodak, E.; R	Istituto Zoopr	2021-02-01
EPI_ISL_16652	A/mute_swan/Austria/21013162_21VIR1085-5	A / H5N5	Europe / Austria	Swan	Bianca Zecchin	Istituto Zooprof	Wodak, E.; R	Istituto Zoopr	2021-02-10
EPI_ISL_16652	A/mute_swan/Austria/21011165_21VIR1085-2	A / H5N8	Europe / Austria	Swan	Bianca Zecchin	Istituto Zooprof	Wodak, E.; R	Istituto Zoopr	2021-02-04
EPI_ISL_84662	A/swan/Poland/MB141/2020(H5N8)	A / H5N8	Europe / Polan	Swan	Edyta Świętoń (	National Veterir	Swieton, E.; S	National Vete	2020-12-16
EPI_ISL_90299	A/Whooper_swan/Scotland/056219/2021	A / H5N1	Europe / United	Cygnus cyg	Alex Byrne (Ani	Animal and Pla		Animal and P	2021-11-09
EPI_ISL_96039	A/mute swan/Czech Republic/785/2022	A / H5N1	Europe / Czech	Cygnus olo	Alexander Nagy	State Veterinar	Alexander,Na	State Veterir	2022-01-10
EPI_ISL_96039	A/mute swan/Czech Republic/22380/2021	A / H5N1	Europe / Czech	Cygnus olo	Alexander Nagy	State Veterinar	Alexander,Na	State Veterir	2021-11-15
EPI_ISL_96039	A/mute swan/Czech Republic/22477-2/2021	A / H5N1	Europe / Czech	Cygnus olo	Alexander Nagy	State Veterinar	Alexander,Na	State Veterir	2021-11-12
EPI_ISL_96039	A/mute swan/Czech Republic/22477-1/2021	A / H5N1	Europe / Czech	Cygnus olo	Alexander Nagy	State Veterinar	Alexander,Na	State Veterir	2021-11-12
EPI_ISL_90299	A/mute_swan/England/385466/2021	A / H5N1	Europe / United	Cygnus olo	Alex Byrne (Ani	Animal and Pla		Animal and P	2021-11-11
EPI_ISL_84409	A/mute_swan/Romania/16790_21VIR11355/20	A / H5N1	Europe / Roma	Cygnus olo	Bianca Zecchin	Istituto Zooprof	Burlacu, R.; N	Institute for D	2021-11-26
EPI_ISL_84401	A/Cygnus_olor/Romania/16381_21VIR10306/2	A / H5N1	Europe / Roma	Cygnus olo	Bianca Zecchin	Istituto Zooprof	Burlacu, R.; N	Institute for D	2021-11-11
EPI_ISL_67610	A/mute swan/Netherlands/21038479-002/2021	A / H5N1	Europe / Nethe	Cygnus olo	Rene Heutink ('	Wageningen Bi	Beerens, Nar	Wageningen	2021-11-09
EPI_ISL_65073	A/mute swan/Croatia/100/2021	A / H5N1	Europe / Croati	Cygnus olo	Vladimir Savić (	Croatian Veteri	Savić, Vladim	Croatian Vet	2021-11-12
EPI_ISL_13057	A/Mute Swan/Sweden/SVA210302Z0465/KN0	A / H5N5	Europe / Swede	Cygnus olo	Siamak Zohari	National Veterir		National Vete	2021-03-01
EPI_ISL_62567	A/mute swan/Inner Mongolia/w2-1/2020	A / H5N8	Asia / China / Ir	Cygnus olo	Hongliang Chai	Northeast Fores		College of W	2020-10-17
EPI_ISL_14937	A/Turkey/Egypt/Giza/2021	A / H5N8	Africa / Egypt	Turkey			Bedair,N.M.; S		2021-03-03
EPI_ISL_12176	A/turkey/Kosovo/13-2_22VIR3124-31/2022	A / H5N8	Europe / Kosov	Turkey	Giacomo Barbi	Istituto Zooprof	Armend, C.; X	Kosovo Food	2022-01-19

EPI_ISL_12028	A/turkey/Italy/21VIR11507/2021	A / H5N1	Europe / Italy	Turkey	Giacomo Barbi	Istituto Zooprof	Barbierato, G	Istituto Zoop	2021-12-20
EPI_ISL_11971	A/turkey/South Dakota/22-009023-002-original	A / H5N1	North America /	Turkey	Mary Lea Killiar	National Veteri	Chinh,Thanh;	National Vet	2022-03-24
EPI_ISL_11971	A/turkey/South Dakota/22-009023-001-original	A / H5N1	North America /	Turkey	Mary Lea Killiar	National Veteri	Chinh,Thanh;	National Vet	2022-03-24
EPI_ISL_11971	A/turkey/Michigan/22-008890-007-original/202	A / H5N1	North America /	Turkey	Mary Lea Killiar	National Veteri	Chinh,Thanh;	National Vet	2022-03-22
EPI_ISL_11971	A/turkey/South Dakota/22-008866-002-original	A / H5N1	North America /	Turkey	Mary Lea Killiar	National Veteri	Chinh,Thanh;	National Vet	2022-03-23
EPI_ISL_11971	A/turkey/South Dakota/22-008866-001-original	A / H5N1	North America /	Turkey	Mary Lea Killiar	National Veteri	Chinh,Thanh;	National Vet	2022-03-23
EPI_ISL_11971	A/turkey/Iowa/22-008862-002-original/2022	A / H5N1	North America /	Turkey	Mary Lea Killiar	National Veteri	Chinh,Thanh;	National Vet	2022-03-23
EPI_ISL_11971	A/turkey/South Dakota/22-008705-002-original	A / H5N1	North America /	Turkey	Mary Lea Killiar	National Veteri	Chinh,Thanh;	National Vet	2022-03-22
EPI_ISL_11971	A/turkey/South Dakota/22-008705-001-original	A / H5N1	North America /	Turkey	Mary Lea Killiar	National Veteri	Chinh,Thanh;	National Vet	2022-03-22
EPI_ISL_11971	A/turkey/South Dakota/22-008702-002-original	A / H5N1	North America /	Turkey	Mary Lea Killiar	National Veteri	Chinh,Thanh;	National Vet	2022-03-22
EPI_ISL_11971	A/turkey/South Dakota/22-008702-001-original	A / H5N1	North America /	Turkey	Mary Lea Killiar	National Veteri	Chinh,Thanh;	National Vet	2022-03-22
EPI_ISL_11971	A/turkey/South Dakota/22-008485-002-original	A / H5N1	North America /	Turkey	Mary Lea Killiar	National Veteri	Chinh,Thanh;	National Vet	2022-03-21
EPI_ISL_11971	A/turkey/South Dakota/22-008485-001-original	A / H5N1	North America /	Turkey	Mary Lea Killiar	National Veteri	Chinh,Thanh;	National Vet	2022-03-21
EPI_ISL_11971	A/turkey/South Dakota/22-008483-001-original	A / H5N1	North America /	Turkey	Mary Lea Killiar	National Veteri	Chinh,Thanh;	National Vet	2022-03-21
EPI_ISL_11971	A/turkey/South Dakota/22-008479-002-original	A / H5N1	North America /	Turkey	Mary Lea Killiar	National Veteri	Chinh,Thanh;	National Vet	2022-03-21
EPI_ISL_11971	A/turkey/South Dakota/22-008479-001-original	A / H5N1	North America /	Turkey	Mary Lea Killiar	National Veteri	Chinh,Thanh;	National Vet	2022-03-21
EPI_ISL_11971	A/turkey/Maine/22-008367-002-original/2022	A / H5N1	North America /	Turkey	Mary Lea Killiar	National Veteri	Chinh,Thanh;	National Vet	2022-03-18
EPI_ISL_11971	A/turkey/South Dakota/22-008242-002-original	A / H5N1	North America /	Turkey	Mary Lea Killiar	National Veteri	Chinh,Thanh;	National Vet	2022-03-17
EPI_ISL_11971	A/turkey/South Dakota/22-008242-001-original	A / H5N1	North America /	Turkey	Mary Lea Killiar	National Veteri	Chinh,Thanh;	National Vet	2022-03-17
EPI_ISL_11971	A/turkey/South Dakota/22-008239-002-original	A / H5N1	North America /	Turkey	Mary Lea Killiar	National Veteri	Chinh,Thanh;	National Vet	2022-03-17
EPI_ISL_11971	A/turkey/South Dakota/22-008239-001-original	A / H5N1	North America /	Turkey	Mary Lea Killiar	National Veteri	Chinh,Thanh;	National Vet	2022-03-17
EPI_ISL_11798	A/turkey/Denmark/24325-25/2021-10-30	A / H5N1	Europe / Denm	Turkey	Charlotte Kristi	Statens Serum	Charlotte Hjul	Statens Seru	2021-10-30
EPI_ISL_11259	A/turkey/Spain/646-7_22VIR2142-37/2022	A / H5N1	Europe / Spain	Turkey	Giacomo Barbi	Istituto Zooprof	Ruano, M.J.;	Laboratorio (	2022-02-15
EPI_ISL_11259	A/turkey/Spain/645-1_22VIR2142-36/2022	A / H5N1	Europe / Spain	Turkey	Giacomo Barbi	Istituto Zooprof	Ruano, M.J.;	Laboratorio (	2022-02-15
EPI_ISL_11259	A/turkey/Spain/586-4_22VIR2142-32/2022	A / H5N1	Europe / Spain	Turkey	Giacomo Barbi	Istituto Zooprof	Ruano, M.J.;	Laboratorio (	2022-02-14
EPI_ISL_11259	A/turkey/Spain/540-26_22VIR2142-28/2022	A / H5N1	Europe / Spain	Turkey	Giacomo Barbi	Istituto Zooprof	Ruano, M.J.;	Laboratorio (	2022-02-09
EPI_ISL_11259	A/turkey/Spain/490-24_22VIR2142-27/2022	A / H5N1	Europe / Spain	Turkey	Giacomo Barbi	Istituto Zooprof	Ruano, M.J.;	Laboratorio (	2022-02-08
EPI_ISL_11259	A/turkey/Spain/490-22_22VIR2142-26/2022	A / H5N1	Europe / Spain	Turkey	Giacomo Barbi	Istituto Zooprof	Ruano, M.J.;	Laboratorio (	2022-02-08
EPI_ISL_11259	A/turkey/Spain/489-21_22VIR2142-25/2022	A / H5N1	Europe / Spain	Turkey	Giacomo Barbi	Istituto Zooprof	Ruano, M.J.;	Laboratorio (	2022-02-08
EPI_ISL_11259	A/turkey/Spain/455-96_22VIR2142-24/2022	A / H5N1	Europe / Spain	Turkey	Giacomo Barbi	Istituto Zooprof	Ruano, M.J.;	Laboratorio (	2022-02-06
EPI_ISL_11259	A/turkey/Spain/455-83_22VIR2142-23/2022	A / H5N1	Europe / Spain	Turkey	Giacomo Barbi	Istituto Zooprof	Ruano, M.J.;	Laboratorio (	2022-02-06
EPI_ISL_11259	A/turkey/Spain/140-38_22VIR2142-19/2022	A / H5N1	Europe / Spain	Turkey	Giacomo Barbi	Istituto Zooprof	Ruano, M.J.;	Laboratorio (	2022-01-15
EPI_ISL_11259	A/turkey/Ireland/033674_22VIR1325-19/2021	A / H5N1	Europe / Irelan	Turkey	Giacomo Barbi	Istituto Zooprof	Byrne, C.; Ga	Central Vete	2021-11-19
EPI_ISL_11259	A/turkey/Ireland/035425_22VIR1325-17/2021	A / H5N1	Europe / Irelan	Turkey	Giacomo Barbi	Istituto Zooprof	Byrne, C.; Ga	Central Vete	2021-12-06
EPI_ISL_88822	A/turkey/Italy/21VIR11803-1/2021	A / H5N1	Europe / Italy	Turkey	Giacomo Barbi	Istituto Zooprof	Barbierato, G	Istituto Zoop	2021-12-27
EPI_ISL_88821	A/turkey/Italy/21VIR11053-1/2021	A / H5N1	Europe / Italy	Turkey	Giacomo Barbi	Istituto Zooprof	Barbierato, G	Istituto Zoop	2021-12-13
EPI_ISL_88821	A/turkey/Italy/21VIR10851/2021	A / H5N1	Europe / Italy	Turkey	Giacomo Barbi	Istituto Zooprof	Barbierato, G	Istituto Zoop	2021-12-06
EPI_ISL_88821	A/turkey/Italy/21VIR11591-8/2021	A / H5N1	Europe / Italy	Turkey	Giacomo Barbi	Istituto Zooprof	Barbierato, G	Istituto Zoop	2021-12-23
EPI_ISL_88821	A/turkey/Italy/21VIR11887-3/2021	A / H5N1	Europe / Italy	Turkey	Giacomo Barbi	Istituto Zooprof	Barbierato, G	Istituto Zoop	2021-12-29
EPI_ISL_88821	A/turkey/Italy/21VIR11804-1/2021	A / H5N1	Europe / Italy	Turkey	Giacomo Barbi	Istituto Zooprof	Barbierato, G	Istituto Zoop	2021-12-27
EPI_ISL_88821	A/turkey/Italy/21VIR11586-2/2021	A / H5N1	Europe / Italy	Turkey	Giacomo Barbi	Istituto Zooprof	Barbierato, G	Istituto Zoop	2021-12-23
EPI_ISL_84168	A/turkey/Lisbon/1/2021	A / H5N1	Europe / Portug	Turkey	Aryse Martins M	Instituto Nacior	Ana Margarid	Instituto Nac	2021-12-05

EPI_ISL_77532	A/turkey/Germany-MV/AI06035/2021	A / H5N1	Europe / Germa	Turkey	Jacqueline King	Friedrich-Loeffl		Landesamt f	2021-10-20
EPI_ISL_55243	A/turkey/Italy/21VIR8585-1/2021	A / H5N1	Europe / Italy /	Turkey	Bianca Zecchin	Istituto Zooprof	Zecchin, B.; F	Istituto Zoopr	2021-10-18
EPI_ISL_51394	A/turkey/Germany-NI/AI02663/2021	A / H5N8	Europe / Germa	Turkey	Jacqueline King	Friedrich-Loeffl		Lebensmittel	2021-03-14
EPI_ISL_51235	A/turkey/Germany-NI/AI02615/2021	A / H5N8	Europe / Germa	Turkey	Jacqueline King	Friedrich-Loeffl		Lebensmittel	2021-03-12
EPI_ISL_51235	A/turkey/Germany-NI/AI03126/2021	A / H5N8	Europe / Germa	Turkey	Jacqueline King	Friedrich-Loeffl		Lebensmittel	2021-03-19
EPI_ISL_51157	A/turkey/Germany-NW/AI02290/2021	A / H5N8	Europe / Germa	Turkey	Jacqueline King	Friedrich-Loeffl		Chemisches	2021-03-02
EPI_ISL_51156	A/turkey/Germany-NI/AI02128/2021	A / H5N8	Europe / Germa	Turkey	Jacqueline King	Friedrich-Loeffl		Lebensmittel	2021-03-04
EPI_ISL_51156	A/turkey/Germany-NI/AI02122/2021	A / H5N8	Europe / Germa	Turkey	Jacqueline King	Friedrich-Loeffl		Lebensmittel	2021-03-04
EPI_ISL_51156	A/turkey/Germany-BB/AI02117/2021	A / H5N8	Europe / Germa	Turkey	Jacqueline King	Friedrich-Loeffl		Landeslabor	2021-03-04
EPI_ISL_51156	A/turkey/Germany-BB/AI01980/2021	A / H5N8	Europe / Germa	Turkey	Jacqueline King	Friedrich-Loeffl		Landeslabor	2021-02-28
EPI_ISL_50993	A/turkey/Germany-MV/AI01477/2021	A / H5N8	Europe / Germa	Turkey	Jacqueline King	Friedrich-Loeffl		Bayrisches L	2021-02-19
EPI_ISL_50981	A/turkey/Germany-BB/AI01419/2021	A / H5N8	Europe / Germa	Turkey	Jacqueline King	Friedrich-Loeffl		Landeslabor	2021-02-15
EPI_ISL_50981	A/turkey/Germany-MV/AI01127/2021	A / H5N8	Europe / Germa	Turkey	Jacqueline King	Friedrich-Loeffl		Landesamt f	2021-02-06
EPI_ISL_50981	A/turkey/Germany-BB/AI01120/2021	A / H5N8	Europe / Germa	Turkey	Jacqueline King	Friedrich-Loeffl		Landeslabor	2021-02-04
EPI_ISL_50981	A/turkey/Germany-NI/AI00591/2021	A / H5N8	Europe / Germa	Turkey	Jacqueline King	Friedrich-Loeffl		Lebensmittel	2021-01-14
EPI_ISL_50981	A/turkey/Germany-NI/AI00616/2021	A / H5N8	Europe / Germa	Turkey	Jacqueline King	Friedrich-Loeffl		Lebensmittel	2021-01-15
EPI_ISL_50981	A/turkey/Germany-NI/AI00612/2021	A / H5N8	Europe / Germa	Turkey	Jacqueline King	Friedrich-Loeffl		Lebensmittel	2021-01-15
EPI_ISL_50652	A/turkey/Germany-NI/AI00042/2021	A / H5N8	Europe / Germa	Turkey	Jacqueline King	Friedrich-Loeffl		Lebensmittel	2021-01-02
EPI_ISL_50651	A/turkey/Germany-NI/AI00038/2021	A / H5N8	Europe / Germa	Turkey	Jacqueline King	Friedrich-Loeffl		Lebensmittel	2021-01-02
EPI_ISL_50650	A/turkey/Germany-NI/AI00016/2021	A / H5N8	Europe / Germa	Turkey	Jacqueline King	Friedrich-Loeffl		Lebensmittel	2020-12-31
EPI_ISL_50649	A/turkey/Germany-NI/AI03609/2020	A / H5N8	Europe / Germa	Turkey	Jacqueline King	Friedrich-Loeffl		Lebensmittel	2020-12-26
EPI_ISL_50649	A/turkey/Germany-NI/AI03606/2020	A / H5N8	Europe / Germa	Turkey	Jacqueline King	Friedrich-Loeffl		Lebensmittel	2020-12-26
EPI_ISL_50648	A/turkey/Germany-NI/AI03599/2020	A / H5N8	Europe / Germa	Turkey	Jacqueline King	Friedrich-Loeffl		Lebensmittel	2020-12-26
EPI_ISL_50647	A/turkey/Germany-NI/AI03594/2020	A / H5N8	Europe / Germa	Turkey	Jacqueline King	Friedrich-Loeffl		Lebensmittel	2020-12-25
EPI_ISL_84660	A/turkey/Poland/477/2020(H5N8)	A / H5N8	Europe / Polan	Turkey	Edyta Świętoń	National Veteri	Swieton, E.; S	National Vet	2020-12-07
EPI_ISL_84660	A/turkey/Poland/475/2020(H5N8)	A / H5N8	Europe / Polan	Turkey	Edyta Świętoń	National Veteri	Swieton, E.; S	National Vet	2020-12-03
EPI_ISL_81397	A/Turkey/Hungary/22494/2020	A / H5N8	Europe / Hunga	Turkey	Katalin Szentpá	National Food	Katalin,Szent	National Foo	2020-05-05
EPI_ISL_12471	A/pelican/Greece/64_TR_22VIR3126-9/2022	A / H5N1	Europe / Greec	Other avian	Giacomo Barbi	Istituto Zooprof	Georgiades,	Thessalonica	2022-03-10
EPI_ISL_12471	A/pelican/Greece/64_LI_22VIR3126-8/2022	A / H5N1	Europe / Greec	Other avian	Giacomo Barbi	Istituto Zooprof	Georgiades,	Thessalonica	2022-03-10
EPI_ISL_12471	A/pelican/Greece/64_SP_22VIR3126-7/2022	A / H5N1	Europe / Greec	Other avian	Giacomo Barbi	Istituto Zooprof	Georgiades,	Thessalonica	2022-03-10
EPI_ISL_12471	A/pelican/Greece/64_KI_22VIR3126-6/2022	A / H5N1	Europe / Greec	Other avian	Giacomo Barbi	Istituto Zooprof	Georgiades,	Thessalonica	2022-03-10
EPI_ISL_12471	A/pelican/Greece/41_AL2_22VIR3126-3/2022	A / H5N1	Europe / Greec	Other avian	Giacomo Barbi	Istituto Zooprof	Georgiades,	Thessalonica	2022-02-24
EPI_ISL_12471	A/pelican/Greece/41_AL1_22VIR3126-2/2022	A / H5N1	Europe / Greec	Other avian	Giacomo Barbi	Istituto Zooprof	Georgiades,	Thessalonica	2022-02-24
EPI_ISL_12471	A/pelican/Greece/72_CL_22VIR3126-11/2022	A / H5N1	Europe / Greec	Other avian	Giacomo Barbi	Istituto Zooprof	Georgiades,	Thessalonica	2022-03-15
EPI_ISL_12471	A/pelican/Greece/69_CL_22VIR3126-10/2022	A / H5N1	Europe / Greec	Other avian	Giacomo Barbi	Istituto Zooprof	Georgiades,	Thessalonica	2022-03-12
EPI_ISL_12471	A/pelican/Greece/41-TR-313_22VIR3126-1/20	A / H5N1	Europe / Greec	Other avian	Giacomo Barbi	Istituto Zooprof	Georgiades,	Thessalonica	2022-02-24
EPI_ISL_12176	A/common_quail/Kosovo/288_22VIR3124-6/20	A / H5N8	Europe / Kosov	Other avian	Giacomo Barbi	Istituto Zooprof	Armend, C.; X	Kosovo Food	2021-10-18
EPI_ISL_12176	A/pelecanus_crispus/Albania/D383-22_22VIR3	A / H5N1	Europe / Albani	Other avian	Giacomo Barbi	Istituto Zooprof	Lika, A.; Boci	Institute of P	2022-03-01
EPI_ISL_11922	A/laying_hen/Romania/10470_22VIR2749-5/20	A / H5N1	Europe / Roma	Other avian	Giacomo Barbi	Istituto Zooprof	Barbuceanu,	Institute for D	2022-02-10
EPI_ISL_11798	A/gray_heron/Denmark/24326-1.02/2021-10-28	A / H5N1	Europe / Denm	Other avian	Charlotte Kristi	Statens Serum	Charlotte Hjul	Statens Seru	2021-10-28
EPI_ISL_11259	A/stork/Spain/234-2_22VIR2142-7/2022	A / H5N1	Europe / Spain	Other avian	Giacomo Barbi	Istituto Zooprof	Ruano, M.J.;	Laboratorio (	2022-01-17
EPI_ISL_11259	A/gray_heron/Spain/88-2_22VIR2142-3/2022	A / H5N1	Europe / Spain	Other avian	Giacomo Barbi	Istituto Zooprof	Ruano, M.J.;	Laboratorio (	2022-01-11

EPI_ISL_11259	A/stork/Spain/729-1_22VIR2142-18/2022	A / H5N1	Europe / Spain	Other aviar	Giacomo Barbi	Istituto Zooprof	Ruano, M.J.;	Laboratorio (	2022-02-22
EPI_ISL_11259	A/gray_heron/Spain/602-1_22VIR2142-17/202	A / H5N1	Europe / Spain	Other aviar	Giacomo Barbi	Istituto Zooprof	Ruano, M.J.;	Laboratorio (	2022-02-10
EPI_ISL_11259	A/common_crane/Spain/597-2_22VIR2142-15/	A / H5N1	Europe / Spain	Other aviar	Giacomo Barbi	Istituto Zooprof	Ruano, M.J.;	Laboratorio (	2022-02-14
EPI_ISL_11259	A/stork/Spain/538-2_22VIR2142-14/2022	A / H5N1	Europe / Spain	Other aviar	Giacomo Barbi	Istituto Zooprof	Ruano, M.J.;	Laboratorio (	2022-02-03
EPI_ISL_11259	A/stork/Spain/442-8_22VIR2142-12/2022	A / H5N1	Europe / Spain	Other aviar	Giacomo Barbi	Istituto Zooprof	Ruano, M.J.;	Laboratorio (	2022-02-03
EPI_ISL_11259	A/brent_goose/Ireland/033257_22VIR1325-9/2	A / H5N1	Europe / Irelan	Other aviar	Giacomo Barbi	Istituto Zooprof	Byrne, C.; Ga	Central Vete	2021-11-16
EPI_ISL_11259	A/white-fronted_goose/Ireland/033181_22VIR1	A / H5N1	Europe / Irelan	Other aviar	Giacomo Barbi	Istituto Zooprof	Byrne, C.; Ga	Central Vete	2021-11-15
EPI_ISL_11259	A/greylag_goose/Ireland/033062_22VIR1325-7	A / H5N1	Europe / Irelan	Other aviar	Giacomo Barbi	Istituto Zooprof	Byrne, C.; Ga	Central Vete	2021-11-15
EPI_ISL_11259	A/greylag_goose/Ireland/032969_22VIR1325-6	A / H5N1	Europe / Irelan	Other aviar	Giacomo Barbi	Istituto Zooprof	Byrne, C.; Ga	Central Vete	2021-11-15
EPI_ISL_11259	A/magpie/Ireland/032958_22VIR1325-5/2021	A / H5N1	Europe / Irelan	Other aviar	Giacomo Barbi	Istituto Zooprof	Byrne, C.; Ga	Central Vete	2021-11-12
EPI_ISL_11259	A/whooper_swan/Ireland/032960_22VIR1325-4	A / H5N1	Europe / Irelan	Other aviar	Giacomo Barbi	Istituto Zooprof	Byrne, C.; Ga	Central Vete	2021-11-12
EPI_ISL_11259	A/peregrine_falcon/Ireland/032476_22VIR1325	A / H5N1	Europe / Irelan	Other aviar	Giacomo Barbi	Istituto Zooprof	Byrne, C.; Ga	Central Vete	2021-11-10
EPI_ISL_11259	A/whooper_swan/Ireland/032444_22VIR1325-2	A / H5N1	Europe / Irelan	Other aviar	Giacomo Barbi	Istituto Zooprof	Byrne, C.; Ga	Central Vete	2021-11-09
EPI_ISL_11259	A/white-tailed_eagle/Ireland/032034_22VIR132	A / H5N1	Europe / Irelan	Other aviar	Giacomo Barbi	Istituto Zooprof	Byrne, C.; Ga	Central Vete	2021-11-03
EPI_ISL_11259	A/layer/Ireland/034424_22VIR1325-21/2021	A / H5N1	Europe / Irelan	Other aviar	Giacomo Barbi	Istituto Zooprof	Byrne, C.; Ga	Central Vete	2021-11-29
EPI_ISL_11259	A/mute_swan/Ireland/032363_22VIR1325-1/20	A / H5N1	Europe / Irelan	Other aviar	Giacomo Barbi	Istituto Zooprof	Byrne, C.; Ga	Central Vete	2021-11-08
EPI_ISL_11259	A/mute_swan/Ireland/033945_22VIR1325-16/2	A / H5N1	Europe / Irelan	Other aviar	Giacomo Barbi	Istituto Zooprof	Byrne, C.; Ga	Central Vete	2021-11-19
EPI_ISL_11259	A/peregrine_falcon/Ireland/000191_22VIR1325	A / H5N1	Europe / Irelan	Other aviar	Giacomo Barbi	Istituto Zooprof	Byrne, C.; Ga	Central Vete	2022-01-05
EPI_ISL_11259	A/mute_swan/Ireland/033169_22VIR1325-14/2	A / H5N1	Europe / Irelan	Other aviar	Giacomo Barbi	Istituto Zooprof	Byrne, C.; Ga	Central Vete	2021-11-15
EPI_ISL_11259	A/buzzard/Ireland/000656_22VIR1325-12/2022	A / H5N1	Europe / Irelan	Other aviar	Giacomo Barbi	Istituto Zooprof	Byrne, C.; Ga	Central Vete	2022-01-07
EPI_ISL_11259	A/crow/Ireland/035624_22VIR1325-11/2021	A / H5N1	Europe / Irelan	Other aviar	Giacomo Barbi	Istituto Zooprof	Byrne, C.; Ga	Central Vete	2021-12-08
EPI_ISL_11259	A/herring_gull/Ireland/033533_22VIR1325-10/2	A / H5N1	Europe / Irelan	Other aviar	Giacomo Barbi	Istituto Zooprof	Byrne, C.; Ga	Central Vete	2021-11-17
EPI_ISL_10497	A/red knot/Germany-SH/AI01010/2022	A / H5N1	Europe / Germa	Other aviar	Jacqueline King	Friedrich-Loeffl		Landeslabor	2022-01-25
EPI_ISL_88822	A/seagull/Italy/21VIR11259-12/2021	A / H5N1	Europe / Italy	Other aviar	Giacomo Barbi	Istituto Zooprof	Barbierato, G	Istituto Zoop	2021-12-15
EPI_ISL_88822	A/buzzard/Italy/21VIR11899-5/2021	A / H5N1	Europe / Italy	Other aviar	Giacomo Barbi	Istituto Zooprof	Barbierato, G	Istituto Zoop	2021-12-27
EPI_ISL_88822	A/owl/Italy/21VIR11899-1/2021	A / H5N1	Europe / Italy	Other aviar	Giacomo Barbi	Istituto Zooprof	Barbierato, G	Istituto Zoop	2021-12-27
EPI_ISL_88822	A/heron/Italy/21VIR10998-1/2021	A / H5N1	Europe / Italy	Other aviar	Giacomo Barbi	Istituto Zooprof	Barbierato, G	Istituto Zoop	2021-12-09
EPI_ISL_88822	A/seagull/Italy/21VIR11259-10/2021	A / H5N1	Europe / Italy	Other aviar	Giacomo Barbi	Istituto Zooprof	Barbierato, G	Istituto Zoop	2021-12-15
EPI_ISL_88822	A/cignus_olor/Italy/IZSLT_21VIR10529-1/2021	A / H5N1	Europe / Italy	Other aviar	Giacomo Barbi	Istituto Zooprof	Barbierato, G	Istituto Zoop	2021-12-01
EPI_ISL_88822	A/seagull/Italy/21VIR10481-2/2021	A / H5N1	Europe / Italy	Other aviar	Giacomo Barbi	Istituto Zooprof	Barbierato, G	Istituto Zoop	2021-11-29
EPI_ISL_88822	A/little_owl/Italy/21VIR11382-1/2021	A / H5N1	Europe / Italy	Other aviar	Giacomo Barbi	Istituto Zooprof	Barbierato, G	Istituto Zoop	2021-12-13
EPI_ISL_88821	A/laying_hen/Italy/21VIR11502/2021	A / H5N1	Europe / Italy	Other aviar	Giacomo Barbi	Istituto Zooprof	Barbierato, G	Istituto Zoop	2021-12-21
EPI_ISL_30012	A/common buzzard/Sweden/SVA210510SZ02(	A / H5N8	Europe / Swed	Other aviar	Siamak Zohari	National Veteri		National Vet	2021-05-21
EPI_ISL_21740	A/common murre/Netherlands/21025491-002/2	A / H5N1	Europe / Nethe	Other aviar	Rene Heutink ('	Wageningen Bi	Beerens, Nar	Wageningen	2021-03-17
EPI_ISL_12406	A/common buzzard /Sweden/SVA210224SZ04	A / H5N8	Europe / Swed	Other aviar	Siamak Zohari	National Veteri		National Vet	2021-02-20
EPI_ISL_12062	A/red knot/Germany-NI/AI00394/2020	A / H5N3	Europe / Germa	Other aviar	Jacqueline King	Friedrich-Loeffl		Lebensmittel	2020-12-30
EPI_ISL_14551	A/fox/New_York/088592/2022	A / H5N1	North America /	mammals	Brittany D Cron	Cornell Univers	Diel, Diego G	Cornell Univ	2022-04-25
EPI_ISL_13201	A/European polecat/Netherlands/1/2022	A / H5N1	Europe / Nethe	mammals	Sanne Thewes	Erasmus Medic		Erasmus Me	2022-03-05
EPI_ISL_14552	A/fox/New_York/099451/2022	A / H5N1	North America /	Other mam	Brittany D Cron	Cornell Univers	Diel, Diego G	Cornell Univ	2022-05-04
EPI_ISL_14494	A/Vulpes_vulpes/Belgium/9031_0008/2022	A / H5N1	Europe / Belgiu	Other mam	Steven Van Bo	Sciensano, Dep	Van Borm, St	Sciensano -	2022-06-16
EPI_ISL_14493	A/Vulpes_vulpes/Belgium/8660_0016/2022	A / H5N1	Europe / Belgiu	Other mam	Steven Van Bo	Sciensano, Dep	Van Borm, St	Sciensano -	2022-04-16
EPI_ISL_13052	A/fox/Minnesota/22-014661-001-original/2022	A / H5N1	North America /	Other mam	Mary Lea Killiar	National Veteri	Chinh,Thanh;	National Vet	2022-05-09

EPI_ISL_13052	A/fox/Minnesota/22-014660-002-original/2022	A / H5N1	North America /	Other mam	Mary Lea Killiar	National Veterii	Chinh,Thanh;		National Vet	2022-05-10
EPI_ISL_13052	A/fox/Minnesota/22-014660-001-original/2022	A / H5N1	North America /	Other mam	Mary Lea Killiar	National Veterii	Chinh,Thanh;		National Vet	2022-05-10
EPI_ISL_13052	A/fox/Iowa/22-014421-001-original/2022	A / H5N1	North America /	Other mam	Mary Lea Killiar	National Veterii	Chinh,Thanh;		National Vet	2022-05-06
EPI_ISL_13052	A/fox/Minnesota/22-014182-001-original/2022	A / H5N1	North America /	Other mam	Mary Lea Killiar	National Veterii	Chinh,Thanh;		National Vet	2022-04-22
EPI_ISL_11259	A/fox/Ireland/3866_22VIR2064-1/2022	A / H5N1	Europe / Irelan	Other mam	Giacomo Barbi	Istituto Zooprof	Byrne, C.; Ga		Central Vete	2022-02-14
EPI_ISL_12069	A/Fox/Netherlands/EMC3/2022	A / H5N1	Europe / Nethe	Animal	Oanh Vuong-Z	Erasmus Medic	R.A.M., Foucl		Erasmus Me	2022-01-22
EPI_ISL_12069	A/Fox/Netherlands/EMC2/2022	A / H5N1	Europe / Nethe	Animal	Oanh Vuong-Z	Erasmus Medic	R.A.M Fouchi		Erasmus Me	2022-01-10
EPI_ISL_15077	A/red-shouldered hawk/Minnesota/22-012000-(	A / H5N1	North America /	Avian	Mary Lea Killiar	National Veterii	Chinh,Thanh;		National Vet	2022-04-18
EPI_ISL_15077	A/black vulture/Florida/22-010358-001-original/	A / H5N1	North America /	Avian	Mary Lea Killiar	National Veterii	Chinh,Thanh;		National Vet	2022-03-31
EPI_ISL_15077	A/chicken/Pennsylvania/22-012092-006-origina	A / H5N1	North America /	Avian	Mary Lea Killiar	National Veterii	Chinh,Thanh;		National Vet	2022-04-19
EPI_ISL_15077	A/chicken/Idaho/22-011347-004-original/2022	A / H5N1	North America /	Avian	Mary Lea Killiar	National Veterii	Chinh,Thanh;		National Vet	2022-04-14
EPI_ISL_14469	A/Common Tern/Netherlands/25/2022	A / H5N1	Europe / Nethe	Avian	Sanne Thewes	Erasmus Medic			Erasmus Me	2022-07-30
EPI_ISL_14469	A/Common Tern/Netherlands/24/2022	A / H5N1	Europe / Nethe	Avian	Sanne Thewes	Erasmus Medic			Erasmus Me	2022-07-29
EPI_ISL_14469	A/Common Tern/Netherlands/23/2022	A / H5N1	Europe / Nethe	Avian	Sanne Thewes	Erasmus Medic			Erasmus Me	2022-07-29
EPI_ISL_14469	A/Common Tern/Netherlands/22/2022	A / H5N1	Europe / Nethe	Avian	Sanne Thewes	Erasmus Medic			Erasmus Me	2022-07-29
EPI_ISL_14469	A/Common Tern/Netherlands/21/2022	A / H5N1	Europe / Nethe	Avian	Sanne Thewes	Erasmus Medic			Erasmus Me	2022-07-29
EPI_ISL_13429	A/Sandwich Tern/Netherlands/7/2022	A / H5N1	Europe / Nethe	Avian	Sanne Thewes	Erasmus Medic			Erasmus Me	2022-06-06
EPI_ISL_13429	A/Sandwich Tern/Netherlands/6/2022	A / H5N1	Europe / Nethe	Avian	Sanne Thewes	Erasmus Medic			Erasmus Me	2022-06-06
EPI_ISL_13429	A/Common Tern/Netherlands/1/2022	A / H5N1	Europe / Nethe	Avian	Sanne Thewes	Erasmus Medic			Erasmus Me	2022-06-03
EPI_ISL_13429	A/Sandwich Tern/Netherlands/3/2022	A / H5N1	Europe / Nethe	Avian	Sanne Thewes	Erasmus Medic			Erasmus Me	2022-06-03
EPI_ISL_13429	A/Sandwich Tern/Netherlands/2/2022	A / H5N1	Europe / Nethe	Avian	Sanne Thewes	Erasmus Medic			Erasmus Me	2022-06-03
EPI_ISL_13429	A/Sandwich Tern/Netherlands/1/2022	A / H5N1	Europe / Nethe	Avian	Sanne Thewes	Erasmus Medic			Erasmus Me	2022-06-04
EPI_ISL_13429	A/Black-headed gull/Netherlands/6/2022	A / H5N1	Europe / Nethe	Avian	Sanne Thewes	Erasmus Medic			Erasmus Me	2022-06-03
EPI_ISL_13429	A/Black-headed gull/Netherlands/5/2022	A / H5N1	Europe / Nethe	Avian	Sanne Thewes	Erasmus Medic			Erasmus Me	2022-06-03
EPI_ISL_13429	A/Sandwich Tern/Netherlands/5/2022	A / H5N1	Europe / Nethe	Avian	Sanne Thewes	Erasmus Medic			Erasmus Me	2022-06-06
EPI_ISL_13429	A/Sandwich Tern/Netherlands/8/2022	A / H5N1	Europe / Nethe	Avian	Sanne Thewes	Erasmus Medic			Erasmus Me	2022-06-06
EPI_ISL_13429	A/Caspian Gull/Netherlands/4/2022	A / H5N1	Europe / Nethe	Avian	Sanne Thewes	Erasmus Medic			Erasmus Me	2022-06-03
EPI_ISL_13201	A/Lesser Black-backed Gull/Netherlands/1/202	A / H5N1	Europe / Nethe	Avian	Sanne Thewes	Erasmus Medic			Erasmus Me	2022-04-29
EPI_ISL_13052	A/turkey/South Dakota/22-009979-002-original	A / H5N1	North America /	Avian	Mary Lea Killiar	National Veterii	Chinh,Thanh;		National Vet	2022-04-01
EPI_ISL_13052	A/turkey/South Dakota/22-009979-001-original	A / H5N1	North America /	Avian	Mary Lea Killiar	National Veterii	Chinh,Thanh;		National Vet	2022-04-01
EPI_ISL_13052	A/turkey/North Dakota/22-009978-002-original/	A / H5N1	North America /	Avian	Mary Lea Killiar	National Veterii	Chinh,Thanh;		National Vet	2022-04-01
EPI_ISL_13052	A/turkey/North Dakota/22-009978-001-original/	A / H5N1	North America /	Avian	Mary Lea Killiar	National Veterii	Chinh,Thanh;		National Vet	2022-04-01
EPI_ISL_13052	A/turkey/Minnesota/22-009976-003-original/20	A / H5N1	North America /	Avian	Mary Lea Killiar	National Veterii	Chinh,Thanh;		National Vet	2022-03-31
EPI_ISL_13052	A/silkie chicken/Wisconsin/22-009934-002-orig	A / H5N1	North America /	Avian	Mary Lea Killiar	National Veterii	Chinh,Thanh;		National Vet	2022-03-31
EPI_ISL_13052	A/chicken/Wyoming/22-009849-001-original/20	A / H5N1	North America /	Avian	Mary Lea Killiar	National Veterii	Chinh,Thanh;		National Vet	2022-03-28
EPI_ISL_13052	A/turkey/Missouri/22-009845-004-original/2022	A / H5N1	North America /	Avian	Mary Lea Killiar	National Veterii	Chinh,Thanh;		National Vet	2022-03-31
EPI_ISL_13052	A/turkey/South Dakota/22-009841-001-original	A / H5N1	North America /	Avian	Mary Lea Killiar	National Veterii	Chinh,Thanh;		National Vet	2022-03-31
EPI_ISL_13052	A/turkey/North Dakota/22-009840-001-original/	A / H5N1	North America /	Avian	Mary Lea Killiar	National Veterii	Chinh,Thanh;		National Vet	2022-03-31
EPI_ISL_13052	A/turkey/South Dakota/22-009839-001-original	A / H5N1	North America /	Avian	Mary Lea Killiar	National Veterii	Chinh,Thanh;		National Vet	2022-03-30
EPI_ISL_13052	A/turkey/Iowa/22-009825-005-original/2022	A / H5N1	North America /	Avian	Mary Lea Killiar	National Veterii	Chinh,Thanh;		National Vet	2022-03-31
EPI_ISL_13052	A/turkey/Minnesota/22-009820-001-original/20	A / H5N1	North America /	Avian	Mary Lea Killiar	National Veterii	Chinh,Thanh;		National Vet	2022-03-30
EPI_ISL_13052	A/chicken/Minnesota/22-009735-001-original/2	A / H5N1	North America /	Avian	Mary Lea Killiar	National Veterii	Chinh,Thanh;		National Vet	2022-03-29

EPI_ISL_13052	A/goose/Illinois/22-009720-003-original/2022	A / H5N1	North America /	Avian	Mary Lea Killiar	National Veteri	Chinh,Thanh;	National Vet	2022-03-29
EPI_ISL_13052	A/chicken/Illinois/22-009720-001-original/2022	A / H5N1	North America /	Avian	Mary Lea Killiar	National Veteri	Chinh,Thanh;	National Vet	2022-03-29
EPI_ISL_13052	A/turkey/South Dakota/22-009688-001-original	A / H5N1	North America /	Avian	Mary Lea Killiar	National Veteri	Chinh,Thanh;	National Vet	2022-03-30
EPI_ISL_13052	A/chicken/North Dakota/22-009677-001-origina	A / H5N1	North America /	Avian	Mary Lea Killiar	National Veteri	Chinh,Thanh;	National Vet	2022-03-30
EPI_ISL_13052	A/chicken/Maine/22-009605-002-original/2022	A / H5N1	North America /	Avian	Mary Lea Killiar	National Veteri	Chinh,Thanh;	National Vet	2022-03-28
EPI_ISL_13052	A/turkey/Maine/22-009605-001-original/2022	A / H5N1	North America /	Avian	Mary Lea Killiar	National Veteri	Chinh,Thanh;	National Vet	2022-03-28
EPI_ISL_11897	A/chicken/Iowa/22-008176-003-original/2022	A / H5N1	North America /	Avian	Mary Lea Killiar	National Veteri	Chinh,Thanh;	National Vet	2022-03-17
EPI_ISL_11897	A/chicken/Kansas/22-008114-002-original/202	A / H5N1	North America /	Avian	Mary Lea Killiar	National Veteri	Chinh,Thanh;	National Vet	2022-03-16
EPI_ISL_11897	A/duck/Kansas/22-008114-001-original/2022	A / H5N1	North America /	Avian	Mary Lea Killiar	National Veteri	Chinh,Thanh;	National Vet	2022-03-16
EPI_ISL_11897	A/chicken/Maine/22-008064-002-original/2022	A / H5N1	North America /	Avian	Mary Lea Killiar	National Veteri	Chinh,Thanh;	National Vet	2022-03-15
EPI_ISL_11897	A/guinea fowl/Maine/22-008064-001-original/20	A / H5N1	North America /	Avian	Mary Lea Killiar	National Veteri	Chinh,Thanh;	National Vet	2022-03-15
EPI_ISL_11897	A/chicken/Delaware/22-008054-003-original/20	A / H5N1	North America /	Avian	Mary Lea Killiar	National Veteri	Chinh,Thanh;	National Vet	2022-03-16
EPI_ISL_11897	A/chicken/Delaware/22-008054-002-original/20	A / H5N1	North America /	Avian	Mary Lea Killiar	National Veteri	Chinh,Thanh;	National Vet	2022-03-16
EPI_ISL_11897	A/turkey/New Hampshire/22-007886-001-origir	A / H5N1	North America /	Avian	Mary Lea Killiar	National Veteri	Chinh,Thanh;	National Vet	2022-03-15
EPI_ISL_11897	A/waterfowl/Nebraska/22-007805-002-original/	A / H5N1	North America /	Avian	Mary Lea Killiar	National Veteri	Chinh,Thanh;	National Vet	2022-03-14
EPI_ISL_11897	A/chicken/Nebraska/22-007805-001-original/20	A / H5N1	North America /	Avian	Mary Lea Killiar	National Veteri	Chinh,Thanh;	National Vet	2022-03-14
EPI_ISL_11897	A/chicken/Missouri/22-007677-002-original/202	A / H5N1	North America /	Avian	Mary Lea Killiar	National Veteri	Chinh,Thanh;	National Vet	2022-03-14
EPI_ISL_11897	A/chicken/Missouri/22-007677-001-original/202	A / H5N1	North America /	Avian	Mary Lea Killiar	National Veteri	Chinh,Thanh;	National Vet	2022-03-14
EPI_ISL_11897	A/chicken/Maine/22-007582-001-original/2022	A / H5N1	North America /	Avian	Mary Lea Killiar	National Veteri	Chinh,Thanh;	National Vet	2022-03-11
EPI_ISL_11897	A/chicken/Wisconsin/22-007545-002-original/2	A / H5N1	North America /	Avian	Mary Lea Killiar	National Veteri	Chinh,Thanh;	National Vet	2022-03-13
EPI_ISL_11897	A/chicken/Wisconsin/22-007545-001-original/2	A / H5N1	North America /	Avian	Mary Lea Killiar	National Veteri	Chinh,Thanh;	National Vet	2022-03-13
EPI_ISL_11897	A/turkey/South Dakota/22-007534-002-original	A / H5N1	North America /	Avian	Mary Lea Killiar	National Veteri	Chinh,Thanh;	National Vet	2022-03-11
EPI_ISL_11897	A/turkey/South Dakota/22-007534-001-original	A / H5N1	North America /	Avian	Mary Lea Killiar	National Veteri	Chinh,Thanh;	National Vet	2022-03-11
EPI_ISL_11897	A/chicken/Maine/22-007410-002-original/2022	A / H5N1	North America /	Avian	Mary Lea Killiar	National Veteri	Chinh,Thanh;	National Vet	2022-03-10
EPI_ISL_11897	A/turkey/Maine/22-007410-001-original/2022	A / H5N1	North America /	Avian	Mary Lea Killiar	National Veteri	Chinh,Thanh;	National Vet	2022-03-10
EPI_ISL_11897	A/poultry/Kansas/22-007391-002-original/2022	A / H5N1	North America /	Avian	Mary Lea Killiar	National Veteri	Chinh,Thanh;	National Vet	2022-03-09
EPI_ISL_11897	A/poultry/Kansas/22-007391-001-original/2022	A / H5N1	North America /	Avian	Mary Lea Killiar	National Veteri	Chinh,Thanh;	National Vet	2022-03-09
EPI_ISL_11897	A/guinea fowl/Illinois/22-007382-001-original/2	A / H5N1	North America /	Avian	Mary Lea Killiar	National Veteri	Chinh,Thanh;	National Vet	2022-03-09
EPI_ISL_11897	A/chicken/Iowa/22-007376-002-original/2022	A / H5N1	North America /	Avian	Mary Lea Killiar	National Veteri	Chinh,Thanh;	National Vet	2022-03-10
EPI_ISL_11897	A/chicken/Iowa/22-007376-001-original/2022	A / H5N1	North America /	Avian	Mary Lea Killiar	National Veteri	Chinh,Thanh;	National Vet	2022-03-10
EPI_ISL_11897	A/chicken/Maryland/22-007273-002-original/20	A / H5N1	North America /	Avian	Mary Lea Killiar	National Veteri	Chinh,Thanh;	National Vet	2022-03-08
EPI_ISL_11897	A/chicken/Maryland/22-007273-001-original/20	A / H5N1	North America /	Avian	Mary Lea Killiar	National Veteri	Chinh,Thanh;	National Vet	2022-03-08
EPI_ISL_11897	A/turkey/Missouri/22-007087-002-original/2022	A / H5N1	North America /	Avian	Mary Lea Killiar	National Veteri	Chinh,Thanh;	National Vet	2022-03-08
EPI_ISL_11897	A/turkey/Missouri/22-007087-001-original/2022	A / H5N1	North America /	Avian	Mary Lea Killiar	National Veteri	Chinh,Thanh;	National Vet	2022-03-08
EPI_ISL_11897	A/chicken/Maryland/22-007086-001-original/20	A / H5N1	North America /	Avian	Mary Lea Killiar	National Veteri	Chinh,Thanh;	National Vet	2022-03-07
EPI_ISL_11897	A/chicken/Maryland/22-006948-001-original/20	A / H5N1	North America /	Avian	Mary Lea Killiar	National Veteri	Chinh,Thanh;	National Vet	2022-03-04
EPI_ISL_11897	A/chicken/Delaware/22-006945-002-original/20	A / H5N1	North America /	Avian	Mary Lea Killiar	National Veteri	Chinh,Thanh;	National Vet	2022-03-07
EPI_ISL_11897	A/chicken/Delaware/22-006945-001-original/20	A / H5N1	North America /	Avian	Mary Lea Killiar	National Veteri	Chinh,Thanh;	National Vet	2022-03-07
EPI_ISL_11897	A/turkey/Missouri/22-006944-002-original/2022	A / H5N1	North America /	Avian	Mary Lea Killiar	National Veteri	Chinh,Thanh;	National Vet	2022-03-07
EPI_ISL_11897	A/turkey/Iowa/22-006795-002-original/2022	A / H5N1	North America /	Avian	Mary Lea Killiar	National Veteri	Chinh,Thanh;	National Vet	2022-03-06
EPI_ISL_11897	A/turkey/Iowa/22-006795-001-original/2022	A / H5N1	North America /	Avian	Mary Lea Killiar	National Veteri	Chinh,Thanh;	National Vet	2022-03-06
EPI_ISL_11897	A/turkey/South Dakota/22-006792-002-original	A / H5N1	North America /	Avian	Mary Lea Killiar	National Veteri	Chinh,Thanh;	National Vet	2022-03-04

EPI_ISL_11897	A/turkey/South Dakota/22-006792-001-original	A / H5N1	North America /	Avian	Mary Lea Killiar	National Veteri	Chinh,Thanh;	National Vet	2022-03-04
EPI_ISL_11897	A/chicken/Missouri/22-006639-001-original/202	A / H5N1	North America /	Avian	Mary Lea Killiar	National Veteri	Chinh,Thanh;	National Vet	2022-03-03
EPI_ISL_11897	A/chicken/Maryland/22-006578-002-original/20	A / H5N1	North America /	Avian	Mary Lea Killiar	National Veteri	Chinh,Thanh;	National Vet	2022-03-03
EPI_ISL_11897	A/chicken/Maryland/22-006578-001-original/20	A / H5N1	North America /	Avian	Mary Lea Killiar	National Veteri	Chinh,Thanh;	National Vet	2022-03-03
EPI_ISL_11628	A/turkey/Iowa/22-006795-002/2022	A / H5N1	North America /	Avian	Mary Lea Killiar	National Veteri	Chinh,Thanh;	National Vet	2022-03-06
EPI_ISL_11628	A/turkey/Iowa/22-006795-001/2022	A / H5N1	North America /	Avian	Mary Lea Killiar	National Veteri	Chinh,Thanh;	National Vet	2022-03-06
EPI_ISL_11628	A/turkey/South Dakota/22-006792-002/2022	A / H5N1	North America /	Avian	Mary Lea Killiar	National Veteri	Chinh,Thanh;	National Vet	2022-03-04
EPI_ISL_11628	A/turkey/South Dakota/22-006792-001/2022	A / H5N1	North America /	Avian	Mary Lea Killiar	National Veteri	Chinh,Thanh;	National Vet	2022-03-04
EPI_ISL_11628	A/chicken/Missouri/22-006639-001/2022	A / H5N1	North America /	Avian	Mary Lea Killiar	National Veteri	Chinh,Thanh;	National Vet	2022-03-03
EPI_ISL_11628	A/chicken/Maryland/22-006578-002/2022	A / H5N1	North America /	Avian	Mary Lea Killiar	National Veteri	Chinh,Thanh;	National Vet	2022-03-03
EPI_ISL_11628	A/chicken/Maryland/22-006578-001/2022	A / H5N1	North America /	Avian	Mary Lea Killiar	National Veteri	Chinh,Thanh;	National Vet	2022-03-03
EPI_ISL_11628	A/chicken/Missouri/22-006569-002/2022	A / H5N1	North America /	Avian	Mary Lea Killiar	National Veteri	Chinh,Thanh;	National Vet	2022-03-02
EPI_ISL_11628	A/chicken/Missouri/22-006569-001/2022	A / H5N1	North America /	Avian	Mary Lea Killiar	National Veteri	Chinh,Thanh;	National Vet	2022-03-02
EPI_ISL_11628	A/turkey/Indiana/22-006261-002/2022	A / H5N1	North America /	Avian	Mary Lea Killiar	National Veteri	Chinh,Thanh;	National Vet	2022-03-01
EPI_ISL_11628	A/turkey/Indiana/22-006261-001/2022	A / H5N1	North America /	Avian	Mary Lea Killiar	National Veteri	Chinh,Thanh;	National Vet	2022-03-01
EPI_ISL_11628	A/turkey/Indiana/22-006259-002/2022	A / H5N1	North America /	Avian	Mary Lea Killiar	National Veteri	Chinh,Thanh;	National Vet	2022-02-28
EPI_ISL_11628	A/turkey/Indiana/22-006259-001/2022	A / H5N1	North America /	Avian	Mary Lea Killiar	National Veteri	Chinh,Thanh;	National Vet	2022-02-28
EPI_ISL_11628	A/chicken/Connecticut/22-006118-001/2022	A / H5N1	North America /	Avian	Mary Lea Killiar	National Veteri	Chinh,Thanh;	National Vet	2022-02-28
EPI_ISL_11628	A/silkie chicken/Iowa/22-006114-001/2022	A / H5N1	North America /	Avian	Mary Lea Killiar	National Veteri	Chinh,Thanh;	National Vet	2022-02-28
EPI_ISL_11628	A/turkey/Indiana/22-005696-003/2022	A / H5N1	North America /	Avian	Mary Lea Killiar	National Veteri	Chinh,Thanh;	National Vet	2022-02-21
EPI_ISL_11628	A/turkey/Indiana/22-005696-002/2022	A / H5N1	North America /	Avian	Mary Lea Killiar	National Veteri	Chinh,Thanh;	National Vet	2022-02-21
EPI_ISL_11628	A/turkey/Indiana/22-005696-001/2022	A / H5N1	North America /	Avian	Mary Lea Killiar	National Veteri	Chinh,Thanh;	National Vet	2022-02-21
EPI_ISL_11628	A/pheasant/New York/22-005647-002/2022	A / H5N1	North America /	Avian	Mary Lea Killiar	National Veteri	Chinh,Thanh;	National Vet	2022-02-22
EPI_ISL_11628	A/pheasant/New York/22-005647-001/2022	A / H5N1	North America /	Avian	Mary Lea Killiar	National Veteri	Chinh,Thanh;	National Vet	2022-02-22
EPI_ISL_11628	A/chicken/Maine/22-005443-001/2022	A / H5N1	North America /	Avian	Mary Lea Killiar	National Veteri	Chinh,Thanh;	National Vet	2022-02-20
EPI_ISL_11628	A/guinea fowl/Michigan/22-005440-003/2022	A / H5N1	North America /	Avian	Mary Lea Killiar	National Veteri	Chinh,Thanh;	National Vet	2022-02-22
EPI_ISL_11628	A/chicken/Michigan/22-005440-002/2022	A / H5N1	North America /	Avian	Mary Lea Killiar	National Veteri	Chinh,Thanh;	National Vet	2022-02-22
EPI_ISL_11628	A/turkey/Michigan/22-005440-001/2022	A / H5N1	North America /	Avian	Mary Lea Killiar	National Veteri	Chinh,Thanh;	National Vet	2022-02-22
EPI_ISL_11628	A/turkey/Indiana/22-005328-002/2022	A / H5N1	North America /	Avian	Mary Lea Killiar	National Veteri	Chinh,Thanh;	National Vet	2022-02-18
EPI_ISL_11628	A/turkey/Indiana/22-005328-001/2022	A / H5N1	North America /	Avian	Mary Lea Killiar	National Veteri	Chinh,Thanh;	National Vet	2022-02-18
EPI_ISL_11628	A/turkey/Indiana/22-005289-002/2022	A / H5N1	North America /	Avian	Mary Lea Killiar	National Veteri	Chinh,Thanh;	National Vet	2022-02-16
EPI_ISL_11628	A/turkey/Indiana/22-005289-001/2022	A / H5N1	North America /	Avian	Mary Lea Killiar	National Veteri	Chinh,Thanh;	National Vet	2022-02-16
EPI_ISL_11628	A/chicken/Delaware/22-005260-003/2022	A / H5N1	North America /	Avian	Mary Lea Killiar	National Veteri	Chinh,Thanh;	National Vet	2022-02-21
EPI_ISL_11628	A/chicken/Delaware/22-005260-002/2022	A / H5N1	North America /	Avian	Mary Lea Killiar	National Veteri	Chinh,Thanh;	National Vet	2022-02-21
EPI_ISL_11628	A/chicken/Delaware/22-005260-001/2022	A / H5N1	North America /	Avian	Mary Lea Killiar	National Veteri	Chinh,Thanh;	National Vet	2022-02-21
EPI_ISL_11628	A/chicken/Maine/22-005158-001/2022	A / H5N1	North America /	Avian	Mary Lea Killiar	National Veteri	Chinh,Thanh;	National Vet	2022-02-17
EPI_ISL_11628	A/turkey/Indiana/22-004688-006/2022	A / H5N1	North America /	Avian	Mary Lea Killiar	National Veteri	Chinh,Thanh;	National Vet	2022-02-14
EPI_ISL_11628	A/turkey/Indiana/22-004688-003/2022	A / H5N1	North America /	Avian	Mary Lea Killiar	National Veteri	Chinh,Thanh;	National Vet	2022-02-14
EPI_ISL_11628	A/turkey/Indiana/22-004688-002/2022	A / H5N1	North America /	Avian	Mary Lea Killiar	National Veteri	Chinh,Thanh;	National Vet	2022-02-14
EPI_ISL_11628	A/turkey/Kentucky/22-004546-002/2022	A / H5N1	North America /	Avian	Mary Lea Killiar	National Veteri	Chinh,Thanh;	National Vet	2022-02-12
EPI_ISL_11628	A/turkey/Kentucky/22-004546-001/2022	A / H5N1	North America /	Avian	Mary Lea Killiar	National Veteri	Chinh,Thanh;	National Vet	2022-02-12
EPI_ISL_11628	A/chicken/Kentucky/22-004416-003/2022	A / H5N1	North America /	Avian	Mary Lea Killiar	National Veteri	Chinh,Thanh;	National Vet	2022-02-11

EPI_ISL_11628	A/chicken/Kentucky/22-004416-002/2022	A / H5N1	North America /	Avian	Mary Lea Killiar	National Veterii	Chinh,Thanh;	National Vet	2022-02-11
EPI_ISL_11628	A/chicken/Kentucky/22-004416-001/2022	A / H5N1	North America /	Avian	Mary Lea Killiar	National Veterii	Chinh,Thanh;	National Vet	2022-02-11
EPI_ISL_11628	A/chicken/Virginia/22-004415-001/2022	A / H5N1	North America /	Avian	Mary Lea Killiar	National Veterii	Chinh,Thanh;	National Vet	2022-02-11
EPI_ISL_11112	A/Great black-backed Gull/Netherlands/2/2022	A / H5N1	Europe / Nethe	Avian	Mark Pronk (Er	Erasmus Medic		Erasmus Me	2022-02-23
EPI_ISL_11112	A/European Herring Gull/Netherlands/2/2022	A / H5N1	Europe / Nethe	Avian	Mark Pronk (Er	Erasmus Medic		Erasmus Me	2022-02-26
EPI_ISL_11112	A/Eurasian Curlew/Netherlands/1/2022	A / H5N1	Europe / Nethe	Avian	Mark Pronk (Er	Erasmus Medic		Erasmus Me	2022-02-09
EPI_ISL_11112	A/Common Gull/Netherlands/1/2022	A / H5N1	Europe / Nethe	Avian	Mark Pronk (Er	Erasmus Medic		Erasmus Me	2022-02-01
EPI_ISL_11112	A/Barnacle Goose/Netherlands/9/2022	A / H5N1	Europe / Nethe	Avian	Mark Pronk (Er	Erasmus Medic		Erasmus Me	2022-02-07
EPI_ISL_11112	A/Barnacle Goose/Netherlands/15/2022	A / H5N1	Europe / Nethe	Avian	Mark Pronk (Er	Erasmus Medic		Erasmus Me	2022-02-26
EPI_ISL_11112	A/Barnacle Goose/Netherlands/13/2022	A / H5N1	Europe / Nethe	Avian	Mark Pronk (Er	Erasmus Medic		Erasmus Me	2022-02-23
EPI_ISL_11112	A/Barnacle Goose/Netherlands/12/2022	A / H5N1	Europe / Nethe	Avian	Mark Pronk (Er	Erasmus Medic		Erasmus Me	2022-02-09
EPI_ISL_11112	A/Barnacle Goose/Netherlands/11/2022	A / H5N1	Europe / Nethe	Avian	Mark Pronk (Er	Erasmus Medic		Erasmus Me	2022-02-09
EPI_ISL_11112	A/Barnacle Goose/Netherlands/10/2022	A / H5N1	Europe / Nethe	Avian	Mark Pronk (Er	Erasmus Medic		Erasmus Me	2022-02-07
EPI_ISL_99887	A/duck/Bangladesh/17D1710/2021	A / H5N1	Asia / Banglade	Avian	Mohammad Er	International Ce	Hossain, M.E	International	2021-07-07
EPI_ISL_77534	A/buzzard/Germany-SH/AI06210/2021	A / H5N1	Europe / Germa	Avian	Jacqueline King	Friedrich-Loeffl		Landeslabor	2021-10-26
EPI_ISL_77534	A/barnacle goose/Germany-SH/AI06145/2021	A / H5N1	Europe / Germa	Avian	Jacqueline King	Friedrich-Loeffl		Landeslabor	2021-10-25
EPI_ISL_77534	A/Eurasian wigeon/Germany-SH/AI06142/2021	A / H5N1	Europe / Germa	Avian	Jacqueline King	Friedrich-Loeffl		Landeslabor	2021-10-22
EPI_ISL_77533	A/Eurasian wigeon/Germany-SH/AI06143/2021	A / H5N1	Europe / Germa	Avian	Jacqueline King	Friedrich-Loeffl		Landeslabor	2021-10-22
EPI_ISL_77533	A/Eurasian teal/Germany-BY/AI05977/2021	A / H5N1	Europe / Germa	Avian	Jacqueline King	Friedrich-Loeffl		Bayrisches L	2021-10-21
EPI_ISL_77533	A/white stork/Germany-MV/AI05979/2021	A / H5N1	Europe / Germa	Avian	Jacqueline King	Friedrich-Loeffl		Landesamt f	2021-10-20
EPI_ISL_77532	A/Eurasian wigeon/Germany-SH/AI05954/2021	A / H5N1	Europe / Germa	Avian	Jacqueline King	Friedrich-Loeffl		Landeslabor	2021-10-14
EPI_ISL_77531	A/Eurasian wigeon/Germany-SH/AI05951/2021	A / H5N1	Europe / Germa	Avian	Jacqueline King	Friedrich-Loeffl		Landeslabor	2021-10-14
EPI_ISL_77531	A/Eurasian wigeon/Germany-SH/AI05956/2021	A / H5N1	Europe / Germa	Avian	Jacqueline King	Friedrich-Loeffl		Landeslabor	2021-10-14
EPI_ISL_77531	A/Eurasian wigeon/Germany-SH/AI05953/2021	A / H5N1	Europe / Germa	Avian	Jacqueline King	Friedrich-Loeffl		Landeslabor	2021-10-14
EPI_ISL_77531	A/Eurasian wigeon/Germany-SH/AI05955/2021	A / H5N1	Europe / Germa	Avian	Jacqueline King	Friedrich-Loeffl		Landeslabor	2021-10-14
EPI_ISL_26810	A/white_stork/Poland/MB391/2021	A / H5N1	Europe / Polan	Avian	Edyta Świętoń	National Veterii	Edyta, Swietc	National Vet	2021-04-20
EPI_ISL_15751	A/Mute Swan/Netherlands/5/2020	A / H5N8	Europe / Nethe	Avian	Theo Bestebroe	Erasmus Medic		Erasmus Me	2020-11-06
EPI_ISL_15751	A/Greylag Goose/Netherlands/1/2020	A / H5N8	Europe / Nethe	Avian	Theo Bestebroe	Erasmus Medic		Erasmus Me	2020-11-06
EPI_ISL_15751	A/Mute Swan/Netherlands/3/2020	A / H5N8	Europe / Nethe	Avian	Theo Bestebroe	Erasmus Medic		Erasmus Me	2020-11-06
EPI_ISL_15751	A/Herring Gull/Netherlands/3/2020	A / H5N8	Europe / Nethe	Avian	Theo Bestebroe	Erasmus Medic		Erasmus Me	2020-11-06
EPI_ISL_15751	A/Herring Gull/Netherlands/2/2020	A / H5N8	Europe / Nethe	Avian	Theo Bestebroe	Erasmus Medic		Erasmus Me	2020-11-06
EPI_ISL_15751	A/Herring Gull/Netherlands/1/2020	A / H5N8	Europe / Nethe	Avian	Theo Bestebroe	Erasmus Medic		Erasmus Me	2020-11-06
EPI_ISL_15751	A/Mute Swan/Netherlands/1/2020	A / H5N8	Europe / Nethe	Avian	Theo Bestebroe	Erasmus Medic		Erasmus Me	2020-11-06
EPI_ISL_15751	A/Common Buzzard/Netherlands/4/2020	A / H5N8	Europe / Nethe	Avian	Theo Bestebroe	Erasmus Medic		Erasmus Me	2020-11-06
EPI_ISL_13057	A/Common peacock/Sweden/SVA210311SZ0	A / H5N5	Europe / Swede	Avian	Siamak Zohari	National Veterii		National Vet	2021-03-10
EPI_ISL_12407	A/northern goshawk/Sweden/SVA210225SZ03	A / H5N8	Europe / Swede	Avian	Siamak Zohari	National Veterii		National Vet	2021-02-21
EPI_ISL_12391	A/northern goshawk/Sweden/SVA210224SZ04	A / H5N8	Europe / Swede	Avian	Siamak Zohari	National Veterii		National Vet	2021-02-22
EPI_ISL_12391	A/northern goshawk/Sweden/SVA210224SZ04	A / H5N8	Europe / Swede	Avian	Siamak Zohari	National Veterii		National Vet	2021-02-24
EPI_ISL_10097	A/duck/Korea/H016/2021	A / H5N8	Asia / Korea, R	Avian	Yu-Na Lee (Ani	Animal and Pla		Animal and F	2021-01-06
EPI_ISL_10097	A/wild duck/Korea/H331/2020	A / H5N8	Asia / Korea, R	Avian	Yu-Na Lee (Ani	Animal and Pla		Animal and F	2020-11-17
EPI_ISL_10097	A/wild bird/Korea/H496-3/2020	A / H5N8	Asia / Korea, R	Avian	Yu-Na Lee (Ani	Animal and Pla		Animal and F	2020-12-16
EPI_ISL_10097	A/spot-billed duck/Korea/WA1000/2020	A / H5N8	Asia / Korea, R	Avian	Yu-Na Lee (Ani	Animal and Pla		Animal and F	2020-12-15

EPI_ISL_10097	A/wild bird/Korea/H467/2020	A / H5N8	Asia / Korea, R	Avian	Yu-Na Lee (Ani	Animal and Pla	Animal and F	2020-12-14
EPI_ISL_10097	A/wild bird/Korea/H379/2020	A / H5N8	Asia / Korea, R	Avian	Yu-Na Lee (Ani	Animal and Pla	Animal and F	2020-12-02
EPI_ISL_10097	A/mandarin duck/Korea/WA913/2020	A / H5N8	Asia / Korea, R	Avian	Yu-Na Lee (Ani	Animal and Pla	Animal and F	2020-12-01
EPI_ISL_10097	A/mandarin duck/Korea/WA899/2020	A / H5N8	Asia / Korea, R	Avian	Yu-Na Lee (Ani	Animal and Pla	Animal and F	2020-12-01
EPI_ISL_10097	A/spot-billed duck/Korea/WA889/2020	A / H5N8	Asia / Korea, R	Avian	Yu-Na Lee (Ani	Animal and Pla	Animal and F	2020-12-01
EPI_ISL_10097	A/mandarin duck/Korea/WA877/2020	A / H5N8	Asia / Korea, R	Avian	Yu-Na Lee (Ani	Animal and Pla	Animal and F	2020-12-01
EPI_ISL_10097	A/mandarin duck/Korea/WA857/2020	A / H5N8	Asia / Korea, R	Avian	Yu-Na Lee (Ani	Animal and Pla	Animal and F	2020-11-26
EPI_ISL_10097	A/spot-billed duck/Korea/WA854/2020	A / H5N8	Asia / Korea, R	Avian	Yu-Na Lee (Ani	Animal and Pla	Animal and F	2020-11-26
EPI_ISL_10096	A/mandarin duck/Korea/WA831/2020	A / H5N8	Asia / Korea, R	Avian	Yu-Na Lee (Ani	Animal and Pla	Animal and F	2020-11-25
EPI_ISL_10096	A/mallard/Korea/WA820/2020	A / H5N8	Asia / Korea, R	Avian	Yu-Na Lee (Ani	Animal and Pla	Animal and F	2020-11-25
EPI_ISL_10096	A/wild bird/Korea/H357/2020	A / H5N8	Asia / Korea, R	Avian	Yu-Na Lee (Ani	Animal and Pla	Animal and F	2020-11-23
EPI_ISL_10096	A/mandarin duck/Korea/WB80/2020	A / H5N8	Asia / Korea, R	Avian	Yu-Na Lee (Ani	Animal and Pla	Animal and F	2020-11-10
EPI_ISL_10096	A/spot-billed duck/Korea/WA612/2020	A / H5N8	Asia / Korea, R	Avian	Yu-Na Lee (Ani	Animal and Pla	Animal and F	2020-11-03
EPI_ISL_10096	A/duck/Korea/H025/2021	A / H5N8	Asia / Korea, R	Avian	Yu-Na Lee (Ani	Animal and Pla	Animal and F	2021-01-09
EPI_ISL_10096	A/chicken/Korea/H022/2021	A / H5N8	Asia / Korea, R	Avian	Yu-Na Lee (Ani	Animal and Pla	Animal and F	2021-01-08
EPI_ISL_10096	A/duck/Korea/H010/2021	A / H5N8	Asia / Korea, R	Avian	Yu-Na Lee (Ani	Animal and Pla	Animal and F	2021-01-05
EPI_ISL_10096	A/duck/Korea/H009/2021	A / H5N8	Asia / Korea, R	Avian	Yu-Na Lee (Ani	Animal and Pla	Animal and F	2021-01-04
EPI_ISL_10096	A/chicken/Korea/H008/2021	A / H5N8	Asia / Korea, R	Avian	Yu-Na Lee (Ani	Animal and Pla	Animal and F	2021-01-04
EPI_ISL_10096	A/chicken/Korea/H002/2021	A / H5N8	Asia / Korea, R	Avian	Yu-Na Lee (Ani	Animal and Pla	Animal and F	2021-01-03
EPI_ISL_10096	A/chicken/Korea/H001/2021	A / H5N8	Asia / Korea, R	Avian	Yu-Na Lee (Ani	Animal and Pla	Animal and F	2021-01-01
EPI_ISL_10096	A/quail/Korea/H551/2020	A / H5N8	Asia / Korea, R	Avian	Yu-Na Lee (Ani	Animal and Pla	Animal and F	2020-12-31
EPI_ISL_10096	A/chicken/Korea/H550/2020	A / H5N8	Asia / Korea, R	Avian	Yu-Na Lee (Ani	Animal and Pla	Animal and F	2020-12-31
EPI_ISL_10096	A/duck/Korea/H549/2020	A / H5N8	Asia / Korea, R	Avian	Yu-Na Lee (Ani	Animal and Pla	Animal and F	2020-12-30
EPI_ISL_10096	A/duck/Korea/H548/2020	A / H5N8	Asia / Korea, R	Avian	Yu-Na Lee (Ani	Animal and Pla	Animal and F	2020-12-30
EPI_ISL_10096	A/duck/Korea/H542/2020	A / H5N8	Asia / Korea, R	Avian	Yu-Na Lee (Ani	Animal and Pla	Animal and F	2020-12-29
EPI_ISL_10096	A/chicken/Korea/H544/2020	A / H5N8	Asia / Korea, R	Avian	Yu-Na Lee (Ani	Animal and Pla	Animal and F	2020-12-29
EPI_ISL_10096	A/chicken/Korea/H541/2020	A / H5N8	Asia / Korea, R	Avian	Yu-Na Lee (Ani	Animal and Pla	Animal and F	2020-12-29
EPI_ISL_10096	A/White peacock/Korea/H533/2020	A / H5N8	Asia / Korea, R	Avian	Yu-Na Lee (Ani	Animal and Pla	Animal and F	2020-12-28
EPI_ISL_10096	A/duck/Korea/H007/2020	A / H5N8	Asia / Korea, R	Avian	Yu-Na Lee (Ani	Animal and Pla	Animal and F	2020-12-28
EPI_ISL_98521	A/duck/Korea/H538/2020	A / H5N8	Asia / Korea, R	Avian	Yu-Na Lee (Ani	Animal and Pla	Animal and F	2020-12-28
EPI_ISL_98521	A/chicken/Korea/H532/2020	A / H5N8	Asia / Korea, R	Avian	Yu-Na Lee (Ani	Animal and Pla	Animal and F	2020-12-28
EPI_ISL_98520	A/chicken/Korea/H531/2020	A / H5N8	Asia / Korea, R	Avian	Yu-Na Lee (Ani	Animal and Pla	Animal and F	2020-12-28
EPI_ISL_98520	A/duck/Korea/H528/2020	A / H5N8	Asia / Korea, R	Avian	Yu-Na Lee (Ani	Animal and Pla	Animal and F	2020-12-27
EPI_ISL_98520	A/chicken/Korea/H526/2020	A / H5N8	Asia / Korea, R	Avian	Yu-Na Lee (Ani	Animal and Pla	Animal and F	2020-12-25
EPI_ISL_98520	A/chicken/Korea/H525/2020	A / H5N8	Asia / Korea, R	Avian	Yu-Na Lee (Ani	Animal and Pla	Animal and F	2020-12-25
EPI_ISL_98520	A/duck/Korea/H524/2020	A / H5N8	Asia / Korea, R	Avian	Yu-Na Lee (Ani	Animal and Pla	Animal and F	2020-12-24
EPI_ISL_98520	A/duck/Korea/H516/2020	A / H5N8	Asia / Korea, R	Avian	Yu-Na Lee (Ani	Animal and Pla	Animal and F	2020-12-23
EPI_ISL_98520	A/duck/Korea/H515/2020	A / H5N8	Asia / Korea, R	Avian	Yu-Na Lee (Ani	Animal and Pla	Animal and F	2020-12-23
EPI_ISL_98520	A/duck/Korea/H514/2020	A / H5N8	Asia / Korea, R	Avian	Yu-Na Lee (Ani	Animal and Pla	Animal and F	2020-12-23
EPI_ISL_98520	A/duck/Korea/H511/2020	A / H5N8	Asia / Korea, R	Avian	Yu-Na Lee (Ani	Animal and Pla	Animal and F	2020-12-22
EPI_ISL_98520	A/duck/Korea/H509/2020	A / H5N8	Asia / Korea, R	Avian	Yu-Na Lee (Ani	Animal and Pla	Animal and F	2020-12-22
EPI_ISL_98519	A/chicken/Korea/H510/2020	A / H5N8	Asia / Korea, R	Avian	Yu-Na Lee (Ani	Animal and Pla	Animal and F	2020-12-22

EPI_ISL_9851§	A/chicken/Korea/H491/2020	A / H5N8	Asia / Korea, R	Avian	Yu-Na Lee (Ani	Animal and Pla		Animal and F	2020-12-21
EPI_ISL_9851§	A/duck/Korea/H499/2020	A / H5N8	Asia / Korea, R	Avian	Yu-Na Lee (Ani	Animal and Pla		Animal and F	2020-12-20
EPI_ISL_9851§	A/duck/Korea/H471/2020	A / H5N8	Asia / Korea, R	Avian	Yu-Na Lee (Ani	Animal and Pla		Animal and F	2020-12-16
EPI_ISL_9851§	A/chicken/Korea/H470/2020	A / H5N8	Asia / Korea, R	Avian	Yu-Na Lee (Ani	Animal and Pla		Animal and F	2020-12-16
EPI_ISL_9851§	A/goose/Korea/H449/2020	A / H5N8	Asia / Korea, R	Avian	Yu-Na Lee (Ani	Animal and Pla		Animal and F	2020-12-14
EPI_ISL_9851§	A/chicken/Korea/H450/2020	A / H5N8	Asia / Korea, R	Avian	Yu-Na Lee (Ani	Animal and Pla		Animal and F	2020-12-14
EPI_ISL_9851§	A/chicken/Korea/H441/2020	A / H5N8	Asia / Korea, R	Avian	Yu-Na Lee (Ani	Animal and Pla		Animal and F	2020-12-14
EPI_ISL_9851§	A/chicken/Korea/H440/2020	A / H5N8	Asia / Korea, R	Avian	Yu-Na Lee (Ani	Animal and Pla		Animal and F	2020-12-12
EPI_ISL_9851§	A/duck/Korea/H439/2020	A / H5N8	Asia / Korea, R	Avian	Yu-Na Lee (Ani	Animal and Pla		Animal and F	2020-12-11
EPI_ISL_9851{	A/duck/Korea/H438/2020	A / H5N8	Asia / Korea, R	Avian	Yu-Na Lee (Ani	Animal and Pla		Animal and F	2020-12-11
EPI_ISL_9851{	A/duck/Korea/H432/2020	A / H5N8	Asia / Korea, R	Avian	Yu-Na Lee (Ani	Animal and Pla		Animal and F	2020-12-10
EPI_ISL_9851{	A/duck/Korea/H431/2020	A / H5N8	Asia / Korea, R	Avian	Yu-Na Lee (Ani	Animal and Pla		Animal and F	2020-12-10
EPI_ISL_9851{	A/duck/Korea/H419/2020	A / H5N8	Asia / Korea, R	Avian	Yu-Na Lee (Ani	Animal and Pla		Animal and F	2020-12-09
EPI_ISL_9851{	A/quail/Korea/H412/2020	A / H5N8	Asia / Korea, R	Avian	Yu-Na Lee (Ani	Animal and Pla		Animal and F	2020-12-08
EPI_ISL_9851{	A/duck/Korea/H411/2020	A / H5N8	Asia / Korea, R	Avian	Yu-Na Lee (Ani	Animal and Pla		Animal and F	2020-12-08
EPI_ISL_9851{	A/quail/Korea/H394/2020	A / H5N8	Asia / Korea, R	Avian	Yu-Na Lee (Ani	Animal and Pla		Animal and F	2020-12-07
EPI_ISL_9851{	A/chicken/Korea/H390/2020	A / H5N8	Asia / Korea, R	Avian	Yu-Na Lee (Ani	Animal and Pla		Animal and F	2020-12-06
EPI_ISL_9851{	A/duck/Korea/H385/2020	A / H5N8	Asia / Korea, R	Avian	Yu-Na Lee (Ani	Animal and Pla		Animal and F	2020-12-04
EPI_ISL_9851{	A/chicken/Korea/H365/2020	A / H5N8	Asia / Korea, R	Avian	Yu-Na Lee (Ani	Animal and Pla		Animal and F	2020-12-01
EPI_ISL_9851{	A/duck/Korea/H338/2020	A / H5N8	Asia / Korea, R	Avian	Yu-Na Lee (Ani	Animal and Pla		Animal and F	2020-11-26
EPI_ISL_1487(	A/chicken/Jalisco/CPA-03604-19/2020	A / H5N2	North America /	Chicken			Xu,W.; Navar		2020-03-05
EPI_ISL_1317§	A/chicken/Bangladesh/18B569/2022	A / H5N1	Asia / Banglade	Chicken	Mohammad Er	International Ce	Hossain, M.E	International	2022-03-11
EPI_ISL_1217(	A/chicken/Albania/D381-22_22VIR3125-1/2022	A / H5N8	Europe / Albani	Chicken	Giacomo Barbi	Istituto Zooprof	Lika, A.; Boci	Institute of P	2022-03-01
EPI_ISL_1217(	A/chicken/Kosovo/22-59_22VIR3124-20/2022	A / H5N8	Europe / Kosov	Chicken	Giacomo Barbi	Istituto Zooprof	Armend, C.; X	Kosovo Fooc	2022-03-01
EPI_ISL_1217(	A/chicken/Kosovo/22-50_22VIR3124-19/2022	A / H5N8	Europe / Kosov	Chicken	Giacomo Barbi	Istituto Zooprof	Armend, C.; X	Kosovo Fooc	2022-02-15
EPI_ISL_1217(	A/chicken/Kosovo/126_22VIR3124-18/2021	A / H5N8	Europe / Kosov	Chicken	Giacomo Barbi	Istituto Zooprof	Armend, C.; X	Kosovo Fooc	2021-06-14
EPI_ISL_1217(	A/chicken/Kosovo/22-9_22VIR3124-15/2022	A / H5N8	Europe / Kosov	Chicken	Giacomo Barbi	Istituto Zooprof	Armend, C.; X	Kosovo Fooc	2022-01-19
EPI_ISL_1217(	A/chicken/Kosovo/22-8_22VIR3124-14/2022	A / H5N8	Europe / Kosov	Chicken	Giacomo Barbi	Istituto Zooprof	Armend, C.; X	Kosovo Fooc	2022-01-14
EPI_ISL_1217(	A/chicken/Kosovo/22-2_22VIR3124-13/2022	A / H5N8	Europe / Kosov	Chicken	Giacomo Barbi	Istituto Zooprof	Armend, C.; X	Kosovo Fooc	2022-01-06
EPI_ISL_1217(	A/chicken/Kosovo/303_22VIR3124-10/2021	A / H5N8	Europe / Kosov	Chicken	Giacomo Barbi	Istituto Zooprof	Armend, C.; X	Kosovo Fooc	2021-11-05
EPI_ISL_1217(	A/chicken/Kosovo/302_22VIR3124-9/2021	A / H5N8	Europe / Kosov	Chicken	Giacomo Barbi	Istituto Zooprof	Armend, C.; X	Kosovo Fooc	2021-11-05
EPI_ISL_1217(	A/chicken/Kosovo/299_22VIR3124-8/2021	A / H5N8	Europe / Kosov	Chicken	Giacomo Barbi	Istituto Zooprof	Armend, C.; X	Kosovo Fooc	2021-11-03
EPI_ISL_1217(	A/chicken/Kosovo/290_22VIR3124-7/2021	A / H5N8	Europe / Kosov	Chicken	Giacomo Barbi	Istituto Zooprof	Armend, C.; X	Kosovo Fooc	2021-10-21
EPI_ISL_1217(	A/chicken/Kosovo/284_22VIR3124-5/2021	A / H5N8	Europe / Kosov	Chicken	Giacomo Barbi	Istituto Zooprof	Armend, C.; X	Kosovo Fooc	2021-10-11
EPI_ISL_1217(	A/chicken/Kosovo/283_22VIR3124-4/2021	A / H5N8	Europe / Kosov	Chicken	Giacomo Barbi	Istituto Zooprof	Armend, C.; X	Kosovo Fooc	2021-10-07
EPI_ISL_1217(	A/chicken/Kosovo/280_22VIR3124-3/2021	A / H5N8	Europe / Kosov	Chicken	Giacomo Barbi	Istituto Zooprof	Armend, C.; X	Kosovo Fooc	2021-10-05
EPI_ISL_1217(	A/chicken/Kosovo/279_22VIR3124-2/2021	A / H5N8	Europe / Kosov	Chicken	Giacomo Barbi	Istituto Zooprof	Armend, C.; X	Kosovo Fooc	2021-10-04
EPI_ISL_1217(	A/chicken/Kosovo/278_22VIR3124-1/2021	A / H5N8	Europe / Kosov	Chicken	Giacomo Barbi	Istituto Zooprof	Armend, C.; X	Kosovo Fooc	2021-09-29
EPI_ISL_11971	A/chicken/Nebraska/22-009006-001-original/2(	A / H5N1	North America /	Chicken	Mary Lea Killiar	National Veterii	Chinh,Thanh;	National Vet	2022-03-23
EPI_ISL_11971	A/chicken/Michigan/22-008890-006-original/20	A / H5N1	North America /	Chicken	Mary Lea Killiar	National Veterii	Chinh,Thanh;	National Vet	2022-03-22
EPI_ISL_11971	A/chicken/South Dakota/22-008704-002-origin	A / H5N1	North America /	Chicken	Mary Lea Killiar	National Veterii	Chinh,Thanh;	National Vet	2022-03-22
EPI_ISL_11971	A/chicken/South Dakota/22-008704-001-origin	A / H5N1	North America /	Chicken	Mary Lea Killiar	National Veterii	Chinh,Thanh;	National Vet	2022-03-22

EPI_ISL_11971	A/chicken/Maine/22-008540-001-original/2022	A / H5N1	North America /	Chicken	Mary Lea Killiar	National Veterii	Chinh,Thanh;	National Vet	2022-03-22
EPI_ISL_11971	A/chicken/Nebraska/22-008467-001-original/2(	A / H5N1	North America /	Chicken	Mary Lea Killiar	National Veterii	Chinh,Thanh;	National Vet	2022-03-21
EPI_ISL_11971	A/chicken/Iowa/22-008373-002-original/2022	A / H5N1	North America /	Chicken	Mary Lea Killiar	National Veterii	Chinh,Thanh;	National Vet	2022-03-19
EPI_ISL_11971	A/chicken/Iowa/22-008373-001-original/2022	A / H5N1	North America /	Chicken	Mary Lea Killiar	National Veterii	Chinh,Thanh;	National Vet	2022-03-19
EPI_ISL_11971	A/chicken/Maine/22-008367-001-original/2022	A / H5N1	North America /	Chicken	Mary Lea Killiar	National Veterii	Chinh,Thanh;	National Vet	2022-03-18
EPI_ISL_11971	A/chicken/Maine/22-008366-001-original/2022	A / H5N1	North America /	Chicken	Mary Lea Killiar	National Veterii	Chinh,Thanh;	National Vet	2022-03-18
EPI_ISL_11971	A/chicken/Kansas/22-008259-002-original/202	A / H5N1	North America /	Chicken	Mary Lea Killiar	National Veterii	Chinh,Thanh;	National Vet	2022-03-16
EPI_ISL_11971	A/chicken/Kansas/22-008259-001-original/202	A / H5N1	North America /	Chicken	Mary Lea Killiar	National Veterii	Chinh,Thanh;	National Vet	2022-03-16
EPI_ISL_11922	A/chicken/Poland/H071_22VIR2515-6/2022	A / H5N1	Europe / Polan	Chicken	Giacomo Barbi	Istituto Zooprof	Swieton, E.; S	National Vet	2022-01-23
EPI_ISL_11922	A/chicken/Poland/H157_22VIR2515-3/2022	A / H5N1	Europe / Polan	Chicken	Giacomo Barbi	Istituto Zooprof	Swieton, E.; S	National Vet	2022-02-18
EPI_ISL_11922	A/chicken/Poland/H182_22VIR2515-1/2022	A / H5N2	Europe / Polan	Chicken	Giacomo Barbi	Istituto Zooprof	Swieton, E.; S	National Vet	2022-02-28
EPI_ISL_11798	A/chicken/Denmark/24357-11/2021-11-02	A / H5N1	Europe / Denm	Chicken	Charlotte Kristi	Statens Serum	Charlotte Hjul	Statens Seru	2021-11-02
EPI_ISL_11725	A/chicken/Germany-BB/AI06242/2021	A / H5N1	Europe / Germa	Chicken	Jacqueline King	Friedrich-Loeffl		Landeslabor	2021-11-02
EPI_ISL_11725	A/chicken/Germany-BB/AI06219/2021	A / H5N1	Europe / Germa	Chicken	Jacqueline King	Friedrich-Loeffl		Landeslabor	2021-10-29
EPI_ISL_11504	A/chicken/Vietnam/HU14-LB11/2021	A / H5N8	Asia / Vietnam	Chicken	Norikazu Isoda	Graduate Scho	Yoshihiro, Sa	Graduate Sc	2021-12-01
EPI_ISL_11259	A/chicken/Spain/649-6_22VIR2142-38/2022	A / H5N1	Europe / Spain	Chicken	Giacomo Barbi	Istituto Zooprof	Ruano, M.J.;	Laboratorio (	2022-02-16
EPI_ISL_11259	A/chicken/Spain/644-8_22VIR2142-35/2022	A / H5N1	Europe / Spain	Chicken	Giacomo Barbi	Istituto Zooprof	Ruano, M.J.;	Laboratorio (	2022-02-16
EPI_ISL_11259	A/chicken/Spain/622-8_22VIR2142-34/2022	A / H5N1	Europe / Spain	Chicken	Giacomo Barbi	Istituto Zooprof	Ruano, M.J.;	Laboratorio (	2022-02-14
EPI_ISL_11259	A/chicken/Spain/587-1_22VIR2142-33/2022	A / H5N1	Europe / Spain	Chicken	Giacomo Barbi	Istituto Zooprof	Ruano, M.J.;	Laboratorio (	2022-02-14
EPI_ISL_11259	A/chicken/Spain/564-11_22VIR2142-31/2022	A / H5N1	Europe / Spain	Chicken	Giacomo Barbi	Istituto Zooprof	Ruano, M.J.;	Laboratorio (	2022-02-11
EPI_ISL_11259	A/chicken/Spain/564-4_22VIR2142-30/2022	A / H5N1	Europe / Spain	Chicken	Giacomo Barbi	Istituto Zooprof	Ruano, M.J.;	Laboratorio (	2022-02-11
EPI_ISL_11259	A/chicken/Spain/562-1_22VIR2142-29/2022	A / H5N1	Europe / Spain	Chicken	Giacomo Barbi	Istituto Zooprof	Ruano, M.J.;	Laboratorio (	2022-02-11
EPI_ISL_11259	A/chicken/Spain/452-17_22VIR2142-22/2022	A / H5N1	Europe / Spain	Chicken	Giacomo Barbi	Istituto Zooprof	Ruano, M.J.;	Laboratorio (	2022-02-07
EPI_ISL_11259	A/chicken/Spain/452-1_22VIR2142-21/2022	A / H5N1	Europe / Spain	Chicken	Giacomo Barbi	Istituto Zooprof	Ruano, M.J.;	Laboratorio (	2022-02-07
EPI_ISL_11259	A/chicken/Spain/340-37_22VIR2142-20/2022	A / H5N1	Europe / Spain	Chicken	Giacomo Barbi	Istituto Zooprof	Ruano, M.J.;	Laboratorio (	2022-01-31
EPI_ISL_11259	A/broiler/Ireland/033734_22VIR1325-20/2021	A / H5N1	Europe / Irelan	Chicken	Giacomo Barbi	Istituto Zooprof	Byrne, C.; Ga	Central Vete	2021-11-22
EPI_ISL_96039	A/chicken/Czech Republic/25690/2021	A / H5N1	Europe / Czech	Chicken	Alexander Nag	State Veterinar	Alexander,Na	State Veterir	2021-12-22
EPI_ISL_88821	A/chicken/Italy/21VIR10573-1/2021	A / H5N1	Europe / Italy	Chicken	Giacomo Barbi	Istituto Zooprof	Barbierato, G	Istituto Zoop	2021-12-03
EPI_ISL_88821	A/chicken/Italy/21VIR10850/2021	A / H5N1	Europe / Italy	Chicken	Giacomo Barbi	Istituto Zooprof	Barbierato, G	Istituto Zoop	2021-12-06
EPI_ISL_74576	A/Chicken/Guangdong/211106-3/2021(H5N6)	A / H5N6	Asia / China / G	Chicken	Jiahao Zhang (	South China Ag	Zhang, Jiaha	South China	2021-08
EPI_ISL_67606	A/Chicken/Hebei/211021/2021(Mixed)	A / H5N6	Asia / China / H	Chicken	Jiahao Zhang (	South China Ag		South China	2021-06
EPI_ISL_67606	A/Chicken/Sichuan/21859-5/2021(H5N6)	A / H5N6	Asia / China / S	Chicken	Jiahao Zhang (	South China Ag	Zhang, Jiaha	South China	2021-07
EPI_ISL_59425	A/chicken/Netherlands/21037750-001005/2021	A / H5N1	Europe / Nethe	Chicken	Rene Heutink (	Wageningen Bi	Beerens, Nar	Wageningen	2021-10-31
EPI_ISL_51238	A/chicken/Germany-SH/AI02562/2021	A / H5N8	Europe / Germa	Chicken	Jacqueline King	Friedrich-Loeffl		Landeslabor	2021-03-11
EPI_ISL_51156	A/chicken/Germany-MV/AI02141/2021	A / H5N8	Europe / Germa	Chicken	Jacqueline King	Friedrich-Loeffl		Landesamt f	2021-03-04
EPI_ISL_51156	A/chicken/Germany-BE/AI01974/2021	A / H5N8	Europe / Germa	Chicken	Jacqueline King	Friedrich-Loeffl		Landeslabor	2021-02-26
EPI_ISL_51156	A/chicken/Germany-MV/AI01965/2021	A / H5N8	Europe / Germa	Chicken	Jacqueline King	Friedrich-Loeffl		Landesamt f	2021-03-01
EPI_ISL_50993	A/chicken/Germany-MV/AI01587/2021	A / H5N8	Europe / Germa	Chicken	Jacqueline King	Friedrich-Loeffl		Landesamt f	2021-02-22
EPI_ISL_50993	A/chicken/Germany-BY/AI01355/2021	A / H5N8	Europe / Germa	Chicken	Jacqueline King	Friedrich-Loeffl		Bayrisches L	2021-02-07
EPI_ISL_50981	A/chicken/Germany-MV/AI01347/2021	A / H5N8	Europe / Germa	Chicken	Jacqueline King	Friedrich-Loeffl		Landesamt f	2021-02-08
EPI_ISL_50981	A/chicken/Germany-NI/AI00887/2021	A / H5N8	Europe / Germa	Chicken	Jacqueline King	Friedrich-Loeffl		Lebensmittel	2021-01-22
EPI_ISL_50495	A/chicken/Luxembourg/21168413/2021	A / H5N8	Europe / Luxem	Chicken	Chantal J. Sno	Luxembourg In	Aurélie Sausy	Laboratoire (	2021-09-03

EPI_ISL_2681(	A/chicken/Romania/12448_21VIR3734-3/2021	A / H5N8	Europe / Roma	Chicken	Bianca Zecchin	Istituto Zooprof	Onita, I.; Neic	Institute for [	2021-05-05
EPI_ISL_16652	A/chicken/Slovakia/Pah10_21VIR1086-5/2021	A / H5N5	Europe / Slovak	Chicken	Bianca Zecchin	Istituto Zooprof	Dirb?kov?, Z.	Istituto Zoopr	2021-01-22
EPI_ISL_16652	A/chicken/Romania/10101_21VIR2044-1/2021	A / H5N5	Europe / Roma	Chicken	Bianca Zecchin	Istituto Zooprof	Onita, I.; Neic	Istituto Zoopr	2021-02-23
EPI_ISL_16652	A/chicken/Italy/21VIR1293-9/2021	A / H5N8	Europe / Italy /	Chicken	Bianca Zecchin	Istituto Zooprof	Zecchin, B.; F	Istituto Zoopr	2021-02-23
EPI_ISL_16652	A/chicken/Italy/21VIR1151-2/2021	A / H5N8	Europe / Italy /	Chicken	Bianca Zecchin	Istituto Zooprof	Zecchin, B.; F	Istituto Zoopr	2021-02-17
EPI_ISL_1273	A/chicken/Tochigi/5T/2021	A / H5N8	Asia / Japan / T	Chicken	Takehiko Saito	National Institut		National Inst	2021-03-13
EPI_ISL_1273	A/chicken/Tochigi/4T/2021	A / H5N8	Asia / Japan / T	Chicken	Takehiko Saito	National Institut		National Inst	2021-03-13
EPI_ISL_1273	A/chicken/Tochigi/3T/2021	A / H5N8	Asia / Japan / T	Chicken	Takehiko Saito	National Institut		National Inst	2021-03-13
EPI_ISL_1273	A/chicken/Tochigi/2T/2021	A / H5N8	Asia / Japan / T	Chicken	Takehiko Saito	National Institut		National Inst	2021-03-13
EPI_ISL_1273	A/chicken/Kagawa/H8T/2020	A / H5N8	Asia / Japan / K	Chicken	Takehiko Saito	National Institut		National Inst	2020-11-20
EPI_ISL_1273	A/chicken/Kagawa/H4T/2020	A / H5N8	Asia / Japan / K	Chicken	Takehiko Saito	National Institut		National Inst	2020-11-20
EPI_ISL_1273	A/chicken/Kagawa/H1T/2020	A / H5N8	Asia / Japan / K	Chicken	Takehiko Saito	National Institut		National Inst	2020-11-20
EPI_ISL_1273	A/chicken/Kagawa/G12T/2020	A / H5N8	Asia / Japan / K	Chicken	Takehiko Saito	National Institut		National Inst	2020-11-19
EPI_ISL_1273	A/chicken/Kagawa/G10T/2020	A / H5N8	Asia / Japan / K	Chicken	Takehiko Saito	National Institut		National Inst	2020-11-19
EPI_ISL_1273	A/chicken/Kagawa/F5T/2020	A / H5N8	Asia / Japan / K	Chicken	Takehiko Saito	National Institut		National Inst	2020-11-19
EPI_ISL_1273	A/chicken/Kagawa/F4T/2020	A / H5N8	Asia / Japan / K	Chicken	Takehiko Saito	National Institut		National Inst	2020-11-19
EPI_ISL_1273	A/chicken/Kagawa/F3T/2020	A / H5N8	Asia / Japan / K	Chicken	Takehiko Saito	National Institut		National Inst	2020-11-19
EPI_ISL_1273	A/chicken/Kagawa/E5T/2020	A / H5N8	Asia / Japan / K	Chicken	Takehiko Saito	National Institut		National Inst	2020-11-14
EPI_ISL_1273	A/chicken/Kagawa/E4T/2020	A / H5N8	Asia / Japan / K	Chicken	Takehiko Saito	National Institut		National Inst	2020-11-14
EPI_ISL_1273	A/chicken/Kagawa/D3T/2020	A / H5N8	Asia / Japan / K	Chicken	Takehiko Saito	National Institut		National Inst	2020-11-12
EPI_ISL_1273	A/chicken/Kagawa/D2T/2020	A / H5N8	Asia / Japan / K	Chicken	Takehiko Saito	National Institut		National Inst	2020-11-12
EPI_ISL_1273	A/chicken/Kagawa/D1T/2020	A / H5N8	Asia / Japan / K	Chicken	Takehiko Saito	National Institut		National Inst	2020-11-12
EPI_ISL_1273	A/chicken/Kagawa/C2T/2020	A / H5N8	Asia / Japan / K	Chicken	Takehiko Saito	National Institut		National Inst	2020-11-10
EPI_ISL_1273	A/chicken/Kagawa/C1T/2020	A / H5N8	Asia / Japan / K	Chicken	Takehiko Saito	National Institut		National Inst	2020-11-10
EPI_ISL_1273	A/chicken/Kagawa/B9T/2020	A / H5N8	Asia / Japan / K	Chicken	Takehiko Saito	National Institut		National Inst	2020-11-07
EPI_ISL_1273	A/chicken/Kagawa/B7T/2020	A / H5N8	Asia / Japan / K	Chicken	Takehiko Saito	National Institut		National Inst	2020-11-07
EPI_ISL_1273	A/chicken/Kagawa/7T/2020	A / H5N8	Asia / Japan / K	Chicken	Takehiko Saito	National Institut		National Inst	2020-11-04
EPI_ISL_1273	A/chicken/Kagawa/6T/2020	A / H5N8	Asia / Japan / K	Chicken	Takehiko Saito	National Institut		National Inst	2020-11-04
EPI_ISL_1273	A/chicken/Kagawa/2T/2020	A / H5N8	Asia / Japan / K	Chicken	Takehiko Saito	National Institut		National Inst	2020-11-04
EPI_ISL_1273	A/chicken/Kagawa/10T/2020	A / H5N8	Asia / Japan / K	Chicken	Takehiko Saito	National Institut		National Inst	2020-11-04
EPI_ISL_1273	A/chicken/Ibaraki/2T/2021	A / H5N8	Asia / Japan / I	Chicken	Takehiko Saito	National Institut		National Inst	2021-02-01
EPI_ISL_1273	A/chicken/Ibaraki/1T/2021	A / H5N8	Asia / Japan / I	Chicken	Takehiko Saito	National Institut		National Inst	2021-02-01
EPI_ISL_1273	A/chicken/Hyogo/4T/2020	A / H5N8	Asia / Japan / H	Chicken	Takehiko Saito	National Institut		National Inst	2020-11-25
EPI_ISL_1273	A/chicken/Hyogo/3T/2020	A / H5N8	Asia / Japan / H	Chicken	Takehiko Saito	National Institut		National Inst	2020-11-25
EPI_ISL_1273	A/chicken/Fukuoka/C2/2020	A / H5N8	Asia / Japan / F	Chicken	Takehiko Saito	National Institut		National Inst	2020-11-24
EPI_ISL_1273	A/chicken/Fukuoka/C1/2020	A / H5N8	Asia / Japan / F	Chicken	Takehiko Saito	National Institut		National Inst	2020-11-24
EPI_ISL_1273	A/chicken/Chiba/F3T/2021	A / H5N8	Asia / Japan / C	Chicken	Takehiko Saito	National Institut		National Inst	2021-02-05
EPI_ISL_9951	A/chicken/Northern_Ireland/2020-17671_21VIF	A / H5N8	Europe / Unitec	Chicken	Bianca Zecchin	Istituto Zooprof	McMenamy, M	AFBI - Agri-F	2020-12-31
EPI_ISL_9852	A/chicken/Miyazaki/K8T/2021	A / H5N8	Asia / Japan / M	Chicken	Takehiko Saito	National Institut		National Inst	2021-02-06
EPI_ISL_9852	A/chicken/Miyazaki/K5T/2021	A / H5N8	Asia / Japan / M	Chicken	Takehiko Saito	National Institut		National Inst	2021-02-06
EPI_ISL_9852	A/chicken/Miyazaki/K3T/2021	A / H5N8	Asia / Japan / M	Chicken	Takehiko Saito	National Institut		National Inst	2021-02-06
EPI_ISL_9852	A/chicken/Miyazaki/K1T/2021	A / H5N8	Asia / Japan / M	Chicken	Takehiko Saito	National Institut		National Inst	2021-02-06

EPI_ISL_98522	A/chicken/Chiba/H2T/2021	A / H5N8	Asia / Japan / C	Chicken	Takehiko Saito	National Institut		National Inst	2021-02-07
EPI_ISL_98522	A/chicken/Chiba/H2C/2021	A / H5N8	Asia / Japan / C	Chicken	Takehiko Saito	National Institut		National Inst	2021-02-07
EPI_ISL_98522	A/chicken/Chiba/H1T/2021	A / H5N8	Asia / Japan / C	Chicken	Takehiko Saito	National Institut		National Inst	2021-02-07
EPI_ISL_98522	A/chicken/Chiba/H1C/2021	A / H5N8	Asia / Japan / C	Chicken	Takehiko Saito	National Institut		National Inst	2021-02-07
EPI_ISL_98522	A/chicken/Chiba/G2T/2021	A / H5N8	Asia / Japan / C	Chicken	Takehiko Saito	National Institut		National Inst	2021-02-06
EPI_ISL_98522	A/chicken/Chiba/G2C/2021	A / H5N8	Asia / Japan / C	Chicken	Takehiko Saito	National Institut		National Inst	2021-02-06
EPI_ISL_98522	A/chicken/Chiba/G1T/2021	A / H5N8	Asia / Japan / C	Chicken	Takehiko Saito	National Institut		National Inst	2021-02-06
EPI_ISL_98522	A/chicken/Chiba/G1C/2021	A / H5N8	Asia / Japan / C	Chicken	Takehiko Saito	National Institut		National Inst	2021-02-06
EPI_ISL_85461	A/chicken/Miyazaki/I9T/2020	A / H5N8	Asia / Japan / M	Chicken	Takehiko Saito	National Institut		National Inst	2020-12-29
EPI_ISL_85461	A/chicken/Miyazaki/I5T/2020	A / H5N8	Asia / Japan / M	Chicken	Takehiko Saito	National Institut		National Inst	2020-12-29
EPI_ISL_85461	A/chicken/Miyazaki/I12T/2020	A / H5N8	Asia / Japan / M	Chicken	Takehiko Saito	National Institut		National Inst	2020-12-29
EPI_ISL_85460	A/chicken/Miyazaki/I11T/2020	A / H5N8	Asia / Japan / M	Chicken	Takehiko Saito	National Institut		National Inst	2020-12-29
EPI_ISL_85460	A/chicken/Kagoshima/6T/2021	A / H5N8	Asia / Japan / K	Chicken	Takehiko Saito	National Institut		National Inst	2021-01-12
EPI_ISL_85460	A/chicken/Kagoshima/5T/2021	A / H5N8	Asia / Japan / K	Chicken	Takehiko Saito	National Institut		National Inst	2021-01-12
EPI_ISL_85460	A/chicken/Kagoshima/3T/2021	A / H5N8	Asia / Japan / K	Chicken	Takehiko Saito	National Institut		National Inst	2021-01-12
EPI_ISL_85460	A/chicken/Kagoshima/1T/2021	A / H5N8	Asia / Japan / K	Chicken	Takehiko Saito	National Institut		National Inst	2021-01-12
EPI_ISL_85460	A/chicken/Gifu/7T/2021	A / H5N8	Asia / Japan / G	Chicken	Takehiko Saito	National Institut		National Inst	2021-01-01
EPI_ISL_85460	A/chicken/Gifu/6T/2021	A / H5N8	Asia / Japan / G	Chicken	Takehiko Saito	National Institut		National Inst	2021-01-01
EPI_ISL_85460	A/chicken/Gifu/3T/2021	A / H5N8	Asia / Japan / G	Chicken	Takehiko Saito	National Institut		National Inst	2021-01-01
EPI_ISL_85460	A/chicken/Gifu/2T/2021	A / H5N8	Asia / Japan / G	Chicken	Takehiko Saito	National Institut		National Inst	2021-01-01
EPI_ISL_85460	A/chicken/Gifu/11T/2021	A / H5N8	Asia / Japan / G	Chicken	Takehiko Saito	National Institut		National Inst	2021-01-01
EPI_ISL_85459	A/chicken/Gifu/10T/2021	A / H5N8	Asia / Japan / G	Chicken	Takehiko Saito	National Institut		National Inst	2021-01-01
EPI_ISL_85459	A/chicken/Chiba/B5T/2021	A / H5N8	Asia / Japan / C	Chicken	Takehiko Saito	National Institut		National Inst	2021-01-10
EPI_ISL_85459	A/chicken/Chiba/B4T/2021	A / H5N8	Asia / Japan / C	Chicken	Takehiko Saito	National Institut		National Inst	2021-01-10
EPI_ISL_85459	A/chicken/Chiba/B3T/2021	A / H5N8	Asia / Japan / C	Chicken	Takehiko Saito	National Institut		National Inst	2021-01-10
EPI_ISL_85459	A/chicken/Chiba/B2T/2021	A / H5N8	Asia / Japan / C	Chicken	Takehiko Saito	National Institut		National Inst	2021-01-10
EPI_ISL_84660	A/chicken/Poland/476/2020(H5N8)	A / H5N8	Europe / Polan	Chicken	Edyta Świętoń (	National Veteri	Swieton, E.; S	National Vet	2020-12-04
EPI_ISL_84660	A/chicken/Poland/474/2020(H5N8)	A / H5N8	Europe / Polan	Chicken	Edyta Świętoń (	National Veteri	Swieton, E.; S	National Vet	2020-12-03
EPI_ISL_84658	A/Chicken/Hungary/29723/2020	A / H5N8	Europe / Hunga	Chicken	Katalin Szentpá	National Food (	Katalin,Szent	National Foo	2020-06-05
EPI_ISL_84658	A/Chicken/Hungary/24596/2020	A / H5N8	Europe / Hunga	Chicken	Katalin Szentpá	National Food (	Katalin,Szent	National Foo	2020-05-13
EPI_ISL_84658	A/Chicken/Hungary/21379/2020	A / H5N8	Europe / Hunga	Chicken	Katalin Szentpá	National Food (	Katalin,Szent	National Foo	2020-04-30
EPI_ISL_73969	A/chicken/Russia_Novosibirsk region/3-29/202	A / H5N8	Europe / Russia	Chicken	Ivan Sobolev (F	WHO National	Sobolev, I.; S	Research Ins	2020-09-20
EPI_ISL_73969	A/chicken/Russia_Novosibirsk region/3-15/202	A / H5N8	Europe / Russia	Chicken	Ivan Sobolev (F	WHO National	Sobolev, I.; S	Research Ins	2020-09-20
EPI_ISL_73969	A/chicken/Russia_Novosibirsk region/3-1/2020	A / H5N8	Europe / Russia	Chicken	Ivan Sobolev (F	WHO National	Sobolev, I.; S	Research Ins	2020-09-20
EPI_ISL_73968	A/chicken/Russia_Novosibirsk region/1910-2/2	A / H5N8	Europe / Russia	Chicken	Ivan Sobolev (F	WHO National	Sobolev, I.; S	Research Ins	2020-09-22
EPI_ISL_73968	A/chicken/Russia_Novosibirsk region/1910-1/2	A / H5N8	Europe / Russia	Chicken	Ivan Sobolev (F	WHO National	Sobolev, I.; S	Research Ins	2020-09-22
EPI_ISL_73968	A/chicken/Kazakhstan/Kn-6/2020	A / H5N8	Asia / Kazakhst	Chicken	Ivan Sobolev (F	WHO National	Sobolev, I.; S	Research Ins	2020-09-18
EPI_ISL_73968	A/chicken/Kazakhstan/Kn-3/2020	A / H5N8	Asia / Kazakhst	Chicken	Ivan Sobolev (F	WHO National	Sobolev, I.; S	Research Ins	2020-09-18
EPI_ISL_73294	A/chicken/Shiga/7T/2020	A / H5N8	Asia / Japan / S	Chicken	Takehiko Saito	National Institut		National Inst	2020-12-12
EPI_ISL_73294	A/chicken/Shiga/6T/2020	A / H5N8	Asia / Japan / S	Chicken	Takehiko Saito	National Institut		National Inst	2020-12-12
EPI_ISL_73294	A/chicken/Shiga/5T/2020	A / H5N8	Asia / Japan / S	Chicken	Takehiko Saito	National Institut		National Inst	2020-12-12
EPI_ISL_73294	A/chicken/Shiga/4T/2020	A / H5N8	Asia / Japan / S	Chicken	Takehiko Saito	National Institut		National Inst	2020-12-12

EPI_ISL_73294	A/chicken/Miyazaki/G9T/2020	A / H5N8	Asia / Japan / M	Chicken	Takehiko Saito	National Institut		National Inst	2020-12-13
EPI_ISL_73294	A/chicken/Miyazaki/G5T/2020	A / H5N8	Asia / Japan / M	Chicken	Takehiko Saito	National Institut		National Inst	2020-12-13
EPI_ISL_73294	A/chicken/Miyazaki/G3T/2020	A / H5N8	Asia / Japan / M	Chicken	Takehiko Saito	National Institut		National Inst	2020-12-13
EPI_ISL_73293	A/chicken/Miyazaki/G1T/2020	A / H5N8	Asia / Japan / M	Chicken	Takehiko Saito	National Institut		National Inst	2020-12-13
EPI_ISL_73293	A/chicken/Miyazaki/F7T/2020	A / H5N8	Asia / Japan / M	Chicken	Takehiko Saito	National Institut		National Inst	2020-12-13
EPI_ISL_73293	A/chicken/Miyazaki/F5T/2020	A / H5N8	Asia / Japan / M	Chicken	Takehiko Saito	National Institut		National Inst	2020-12-13
EPI_ISL_73293	A/chicken/Miyazaki/F4T/2020	A / H5N8	Asia / Japan / M	Chicken	Takehiko Saito	National Institut		National Inst	2020-12-13
EPI_ISL_73293	A/chicken/Miyazaki/F1T/2020	A / H5N8	Asia / Japan / M	Chicken	Takehiko Saito	National Institut		National Inst	2020-12-13
EPI_ISL_73293	A/chicken/Kochi/7T/2020	A / H5N8	Asia / Japan / K	Chicken	Takehiko Saito	National Institut		National Inst	2020-12-15
EPI_ISL_73293	A/chicken/Kochi/6T/2020	A / H5N8	Asia / Japan / K	Chicken	Takehiko Saito	National Institut		National Inst	2020-12-15
EPI_ISL_73293	A/chicken/Kochi/5C/2020	A / H5N8	Asia / Japan / K	Chicken	Takehiko Saito	National Institut		National Inst	2020-12-15
EPI_ISL_73293	A/chicken/Kochi/4C/2020	A / H5N8	Asia / Japan / K	Chicken	Takehiko Saito	National Institut		National Inst	2020-12-15
EPI_ISL_73293	A/chicken/Kagawa/K6T/2020	A / H5N8	Asia / Japan / K	Chicken	Takehiko Saito	National Institut		National Inst	2020-12-13
EPI_ISL_73292	A/chicken/Kagawa/K4T/2020	A / H5N8	Asia / Japan / K	Chicken	Takehiko Saito	National Institut		National Inst	2020-12-13
EPI_ISL_73292	A/chicken/Kagawa/K3T/2020	A / H5N8	Asia / Japan / K	Chicken	Takehiko Saito	National Institut		National Inst	2020-12-13
EPI_ISL_73292	A/chicken/Kagawa/K2T/2020	A / H5N8	Asia / Japan / K	Chicken	Takehiko Saito	National Institut		National Inst	2020-12-13
EPI_ISL_14494	A/Gallus_gallus/Belgium/9548_0001/2022	A / H5N1	Europe / Belgiu	Gallus gallu	Steven Van Bor	Sciensano, Dep	Van Borm, St	Sciensano -	2022-08-03
EPI_ISL_63280	A/chicken/Netherlands/21038675-001005/2021	A / H5N1	Europe / Nethe	Gallus gallu	Rene Heutink ('	Wageningen Bi	Beerens, Nar	Wageningen	2021-11-14
EPI_ISL_63280	A/chicken/Netherlands/21037907-006010/2021	A / H5N1	Europe / Nethe	Gallus gallu	Rene Heutink ('	Wageningen Bi	Beerens, Nar	Wageningen	2021-11-02
EPI_ISL_24029	A/chicken/Czech Republic/10405/2021	A / H5N8	Europe / Czech	Gallus gallu	Alexander Nagy	State Veterinar	Nagy,A;Cerni	State Veterir	2021-05-17
EPI_ISL_24029	A/chicken/Czech Republic/10251-2/2021	A / H5N8	Europe / Czech	Gallus gallu	Alexander Nagy	State Veterinar	Nagy,A;Cerni	State Veterir	2021-05-31
EPI_ISL_24029	A/chicken/Czech Republic/10251-1/2021	A / H5N8	Europe / Czech	Gallus gallu	Alexander Nagy	State Veterinar	Nagy,A;Cerni	State Veterir	2021-05-16
EPI_ISL_64139	A/chicken/Netherlands/20017694-004/2020	A / H5N8	Europe / Nethe	Gallus gallu	Rene Heutink ('	Wageningen Bi	Beerens, Nar	Wageningen	2020-11-11
EPI_ISL_64139	A/chicken/Netherlands/20017639-001/2020	A / H5N8	Europe / Nethe	Gallus gallu	Rene Heutink ('	Wageningen Bi	Beerens, Nar	Wageningen	2020-11-10
EPI_ISL_64137	A/chicken/Netherlands/20016978-001/2020	A / H5N8	Europe / Nethe	Gallus gallu	Rene Heutink ('	Wageningen Bi	Beerens, Nar	Wageningen	2020-11-02
EPI_ISL_14822	A/Wild Duck/Ningxia/Y54/2021	A / H5N8	Asia / China / N	Duck	Zeyu Yang (Ch	Chinese Acade		Xi'an Tianlon	2021-03-11
EPI_ISL_13175	A/duck/Bangladesh/19D1890/2022	A / H5N1	Asia / Banglade	Duck	Mohammad Er	International Ce	Hossain, M.E	International	2022-04-20
EPI_ISL_13175	A/duck/Bangladesh/18D1853/2022	A / H5N1	Asia / Banglade	Duck	Mohammad Er	International Ce	Hossain, M.E	International	2022-03-11
EPI_ISL_13175	A/duck/Bangladesh/18D1851/2022	A / H5N1	Asia / Banglade	Duck	Mohammad Er	International Ce	Hossain, M.E	International	2022-03-11
EPI_ISL_13175	A/duck/Bangladesh/17D1869/2022	A / H5N1	Asia / Banglade	Duck	Mohammad Er	International Ce	Hossain, M.E	International	2022-03-06
EPI_ISL_12437	A/duck/Czech Republic/6017/2021	A / H5N8	Europe / Czech	Duck	Alexander Nagy	State Veterinar	Alexander,Na	State Veterir	2021-03-26
EPI_ISL_12437	A/duck/Czech Republic/5467/2021	A / H5N8	Europe / Czech	Duck	Alexander Nagy	State Veterinar	Alexander,Na	State Veterir	2021-03-19
EPI_ISL_12324	A/duck/Czech Republic/3306-1/2022	A / H5N1	Europe / Czech	Duck	Alexander Nagy	State Veterinar	Nagy,Alexanc	State Veterir	2022-02-09
EPI_ISL_11971	A/domestic duck/Michigan/22-008890-001-orig	A / H5N1	North America /	Duck	Mary Lea Killiar	National Veteri	Chinh,Thanh;	National Vete	2022-03-22
EPI_ISL_11922	A/duck/Poland/H126_22VIR2515-4/2022	A / H5N1	Europe / Polan	Duck	Giacomo Barbi	Istituto Zooprof	Swieton, E.; S	National Vete	2022-02-08
EPI_ISL_11922	A/duck/Poland/H188_22VIR2515-2/2022	A / H5N1	Europe / Polan	Duck	Giacomo Barbi	Istituto Zooprof	Swieton, E.; S	National Vete	2022-03-02
EPI_ISL_11725	A/domestic duck/Germany-BB/AI06239/2021	A / H5N1	Europe / Germa	Duck	Jacqueline King	Friedrich-Loeffl		Landeslabor	2021-11-02
EPI_ISL_11259	A/duck/Ireland/036105_22VIR1325-22/2021	A / H5N1	Europe / Irelan	Duck	Giacomo Barbi	Istituto Zooprof	Byrne, C.; Ga	Central Vete	2021-12-11
EPI_ISL_11259	A/duck/Ireland/036646_22VIR1325-18/2021	A / H5N1	Europe / Irelan	Duck	Giacomo Barbi	Istituto Zooprof	Byrne, C.; Ga	Central Vete	2021-12-17
EPI_ISL_77532	A/mallard/Germany-NI/AI06010/2021	A / H5N1	Europe / Germa	Duck	Jacqueline King	Friedrich-Loeffl		Lebensmittel	2021-10-17
EPI_ISL_67606	A/Duck/Sichuan/21957-1/2021(H5N6)	A / H5N6	Asia / China / S	Duck	Jiahao Zhang (	South China Ag		South China	2021-07
EPI_ISL_67606	A/Duck/Sichuan/21921-1/2021(H5N6)	A / H5N6	Asia / China / S	Duck	Jiahao Zhang (	South China Ag		South China	2021-07

EPI_ISL_67606	A/Duck/Sichuan/21898/2021(H5N6)	A / H5N6	Asia / China / S	Duck	Jiahao Zhang (	South China Ag	Zhang, Jiahao	South China	2021-07
EPI_ISL_67606	A/Duck/Sichuan/21840-5/2021(H5N6)	A / H5N6	Asia / China / S	Duck	Jiahao Zhang (	South China Ag	Zhang, Jiahao	South China	2021-07
EPI_ISL_67606	A/Duck/Sichuan/21826-5/2021(H5N6)	A / H5N6	Asia / China / S	Duck	Jiahao Zhang (	South China Ag	Zhang, Jiahao	South China	2021-07
EPI_ISL_67604	A/Duck/Sichuan/21826-1/2021(H5N6)	A / H5N6	Asia / China / S	Duck	Jiahao Zhang (	South China Ag	Zhang, Jiahao	South China	2021-07
EPI_ISL_51235	A/domestic duck/Germany-BB/AI02599/2021	A / H5N8	Europe / Germa	Duck	Jacqueline King	Friedrich-Loeffl		Landeslabor	2021-03-11
EPI_ISL_51235	A/domestic duck/Germany-NI/AI03099/2021	A / H5N8	Europe / Germa	Duck	Jacqueline King	Friedrich-Loeffl		Lebensmittel	2021-03-19
EPI_ISL_50993	A/domestic duck/Germany-BB/AI01444/2021	A / H5N8	Europe / Germa	Duck	Jacqueline King	Friedrich-Loeffl		Landeslabor	2021-02-17
EPI_ISL_50981	A/domestic duck/Germany-BB/AI01437/2021	A / H5N8	Europe / Germa	Duck	Jacqueline King	Friedrich-Loeffl		Landeslabor	2021-02-17
EPI_ISL_50981	A/domestic duck/Germany-BB/AI01423/2021	A / H5N8	Europe / Germa	Duck	Jacqueline King	Friedrich-Loeffl		Landeslabor	2021-02-16
EPI_ISL_50981	A/wild duck/Germany-NI/AI03479/2020	A / H5N8	Europe / Germa	Duck	Jacqueline King	Friedrich-Loeffl		Lebensmittel	2020-12-16
EPI_ISL_50578	A/Eurasian wigeon/Germany-SH/AI02176/2020	A / H5N8	Europe / Germa	Duck	Jacqueline King	Friedrich-Loeffl		Landeslabor	2020-10-30
EPI_ISL_16652	A/muscovy_duck/Slovakia/Pah1_21VIR1086-1	A / H5N8	Europe / Slovak	Duck	Bianca Zecchin	Istituto Zooprof	Dirb?kov?, Z.	Istituto Zoopr	2021-01-08
EPI_ISL_16652	A/duck/Romania/10206_21VIR849-4/2021	A / H5N8	Europe / Roma	Duck	Bianca Zecchin	Istituto Zooprof	Onita, I.; Neic	Istituto Zoopr	2021-01-13
EPI_ISL_16652	A/duck/Italy/21VIR1293-15/2021	A / H5N8	Europe / Italy /	Duck	Bianca Zecchin	Istituto Zooprof	Zecchin, B.; F	Istituto Zoopr	2021-02-23
EPI_ISL_98523	A/mallard duck/Niigata/150209T/2021	A / H5N8	Asia / Japan / N	Duck	Takehiko Saito	National Institut		National Inst	2021-02-08
EPI_ISL_98523	A/mallard duck/Niigata/150209C/2021	A / H5N8	Asia / Japan / N	Duck	Takehiko Saito	National Institut		National Inst	2021-02-08
EPI_ISL_97759	A/duck/Northern China/LSP/2020(H5N8)	A / H5N8	Asia / China / S	Duck	Jiahao Zhang (	South China Ag	Zhang, Jiahao	South China	2020-12-21
EPI_ISL_97759	A/duck/Southwestern China/B1904/2020(H5N8	A / H5N8	Asia / China / S	Duck	Jiahao Zhang (	South China Ag	Zhang, Jiahao	South China	2020-12-31
EPI_ISL_97758	A/duck/Northern China/ZGL/2020(H5N8)	A / H5N8	Asia / China / S	Duck	Jiahao Zhang (	South China Ag	Zhang, Jiahao	South China	2020-12-29
EPI_ISL_64151	A/duck/Netherlands/20017868-016020/2020	A / H5N8	Europe / Nethe	Duck	Rene Heutink ('	Wageningen Bi	Beerens, Nar	Wageningen	2020-11-13
EPI_ISL_11561	A/domestic_duck/England/012973/2022	A / H5N1	Europe / Unitec	Anas platyr	Alex Byrne (Ani	Animal and Pla		Animal and F	2022-02-08
EPI_ISL_11406	A/domestic_duck/England/007588/2022	A / H5N1	Europe / Unitec	Anas platyr	Alex Byrne (Ani	Animal and Pla		Animal and F	2022-01-23
EPI_ISL_90299	A/domestic_duck/England/058612/2021	A / H5N1	Europe / Unitec	Anas platyr	Alex Byrne (Ani	Animal and Pla		Animal and F	2021-11-18
EPI_ISL_69341	A/domestic_duck/Poland/H1942-N/2021	A / H5N1	Europe / Polan	Anas platyr	Edyta Świętoń (	National Veterii	E. Swieton, K	National Vet	2021-11-07
EPI_ISL_14822	A/Spot-billed Duck/Ningxia/Y26/2021	A / H5N8	Asia / China / N	Anas poeci	Zeyu Yang (Ch	Chinese Acade		Xi'an Tianlon	2021-03-11
EPI_ISL_14886	A/duck/Bangladesh/51601/2021	A / H5N1	Asia / Banglade	Anas sp.			Barman,S.; T		2021-12-19
EPI_ISL_13057	A/Duck/Sweden/SVA210311SZ0004/KN00015	A / H5N5	Europe / Swede	Anas sp.	Siamak Zohari	National Veterii		National Vet	2021-03-10
EPI_ISL_77532	A/white-tailed eagle/Germany-MV/AI05975/202	A / H5N1	Europe / Germa	Eagle	Jacqueline King	Friedrich-Loeffl		Landesamt f	2021-10-20
EPI_ISL_14388	A/Buteo_buteo/belgium/334_0013/2021	A / H5N1	Europe / Belgiu	Buteo bute	Steven Van Bo	Sciensano, Dep	Van Borm, St	Sciensano -	2021-12-24
EPI_ISL_11798	A/common_buzzard/Denmark/24271-1.02/202	A / H5N1	Europe / Denm	Buteo bute	Charlotte Kristi	Statens Serum	Charlotte Hjul	Statens Seru	2021-10-25
EPI_ISL_11725	A/buzzard/Germany-SH/AI07099/2021	A / H5N1	Europe / Germa	Buteo bute	Jacqueline King	Friedrich-Loeffl		Landeslabor	2021-11-09
EPI_ISL_10497	A/buzzard/Germany-BB/AI01212/2022	A / H5N1	Europe / Germa	Buteo bute	Jacqueline King	Friedrich-Loeffl		Landeslabor	2022-02-11
EPI_ISL_51466	A/buzzard/Germany-NI/AI04429/2021	A / H5N1	Europe / Germa	Buteo bute	Jacqueline King	Friedrich-Loeffl		Lebensmittel	2021-04-28
EPI_ISL_90299	A/Canada_goose/England/385250/2021	A / H5N1	Europe / Unitec	Branta cana	Alex Byrne (Ani	Animal and Pla		Animal and F	2021-11-01
EPI_ISL_13057	A/Canada_goose/Sweden/SVA210302SZ0455	A / H5N8	Europe / Swede	Branta cana	Siamak Zohari	National Veterii		National Vet	2021-02-19
EPI_ISL_10053	A/harbour_seal/Denmark/521-2/2021-09-15(H5	A / H5N8	Europe / Denm	Primate	Charlotte Kristi	Statens Serum	Hjulsager, Ch	National Vet	2021-09-15
EPI_ISL_14722	A/vulture/France/22P018210/2022	A / H5N1	Europe / Franc	Wild bird	Francois-Xavie	ANSES Agence		Anses (Plouf	2022-05-09
EPI_ISL_14388	A/Tyto_alba/Belgium/334_0012/2021	A / H5N1	Europe / Belgiu	Wild bird	Steven Van Bo	Sciensano, Dep	Van Borm, St	Sciensano -	2021-12-23
EPI_ISL_85154	A/great egret/Czech_Republic/23609/2021	A / H5N1	Europe / Czech	Wild bird	Alexander Nagy	State Veterinar	Alexander,Na	State Veterir	2021-11-28
EPI_ISL_85154	A/grey heron/Czech_Republic/23608/2021	A / H5N1	Europe / Czech	Wild bird	Alexander Nagy	State Veterinar	Alexander,Na	State Veterir	2021-11-28
EPI_ISL_16652	A/house_sparrow/Romania/10327_21VIR849-5	A / H5N8	Europe / Roma	Wild bird	Bianca Zecchin	Istituto Zooprof	Onita, I.; Neic	Istituto Zoopr	2021-01-19
EPI_ISL_16652	A/common_buzzard/Italy/21VIR431-7/2020	A / H5N8	Europe / Italy /	Wild bird	Bianca Zecchin	Istituto Zooprof	Zecchin, B.; F	Istituto Zoopr	2020-11-17

EPI_ISL_81397	A/peregrine_falcon/Ireland/20VIR7872-1/2020	A / H5N8	Europe / Ireland	Wild bird	Bianca Zecchin	Istituto Zooprof	Flynn, O.; Co	Central Vete	2020-11
EPI_ISL_68375	A/Eurasian_wigeon/Italy/20VIR7301-31/2020	A / H5N8	Europe / Italy /	Wild bird	Bianca Zecchin	Istituto Zooprof		Istituto Zoopr	2020-11-21
EPI_ISL_68359	A/Eurasian_wigeon/Italy/20VIR7139-121/2020	A / H5N8	Europe / Italy /	Wild bird	Bianca Zecchin	Istituto Zooprof	Zecchin, B.; F	Istituto Zoopr	2020-11-14
EPI_ISL_68359	A/Eurasian_wigeon/Italy/20VIR7301-206/2020	A / H5N1	Europe / Italy /	Wild bird	Bianca Zecchin	Istituto Zooprof	Zecchin, B.; F	Istituto Zoopr	2020-11-21
EPI_ISL_11971	A/mallard/New York/22-008760-007-original/20	A / H5N1	North America /	Mallard	Mary Lea Killian	National Veterin	Chinh,Thanh;	National Vete	2022-03-22
EPI_ISL_28205	A/mallard/Ningxia/249/2020	A / H5N8	Asia / China / N	Mallard	Hongliang Chai	Northeast Fores		College of W	2020-10-16
EPI_ISL_28205	A/mallard/Ningxia/247/2020	A / H5N8	Asia / China / N	Mallard	Hongliang Chai	Northeast Fores		College of W	2020-10-16
EPI_ISL_28204	A/mallard/Ningxia/241/2020	A / H5N8	Asia / China / N	Mallard	Hongliang Chai	Northeast Fores		College of W	2020-10-16
EPI_ISL_28202	A/mallard/Ningxia/239/2020	A / H5N8	Asia / China / N	Mallard	Hongliang Chai	Northeast Fores		College of W	2020-10-16
EPI_ISL_28202	A/mallard/Ningxia/176/2020	A / H5N8	Asia / China / N	Mallard	Hongliang Chai	Northeast Fores		College of W	2020-10-15
EPI_ISL_12635	A/mallard/France/20P017917/2020	A / H5N3	Europe / France	Mallard	Francois-Xavier	ANSES Agence		Anses (Plouf	2020-12-07
EPI_ISL_68359	A/mallard/Italy/20VIR7139-124_feather/2020	A / H5N8	Europe / Italy /	Mallard	Bianca Zecchin	Istituto Zooprof	Zecchin, B.; F	Istituto Zoopr	2020-11-14
EPI_ISL_11798	A/Eurasian_wigeon/Denmark/24279-1/2021-10	A / H5N1	Europe / Denm	Mareca per	Charlotte Kristia	Statens Serum	Charlotte Hjul	Statens Seru	2021-10-24
EPI_ISL_14206	A/wigeon/Latvia/23903/2021	A / H5N8	Europe / Latvia	Mareca per	Juris Kibilds (In	Institute of Food	Kibilds J, Cve	Institute of F	2021-02-04
EPI_ISL_11798	A/greylag_goose/Denmark/24343-1.02/2021-1	A / H5N1	Europe / Denm	Greylag goo	Charlotte Kristia	Statens Serum	Charlotte Hjul	Statens Seru	2021-11-01
EPI_ISL_11798	A/greylag_goose/Denmark/24309-1.01/2021-10	A / H5N1	Europe / Denm	Greylag goo	Charlotte Kristia	Statens Serum	Charlotte Hjul	Statens Seru	2021-10-27
EPI_ISL_70553	A/greylag goose /Sweden/SVA211118SZ0354/	A / H5N1	Europe / Swede	Greylag goo	Siamak Zohari	National Veterin		National Vete	2021-11-17
EPI_ISL_85154	A/grey heron/Czech_Republic/23608-1K/2021	A / H5N1	Europe / Czech	Wild birds	Alexander Nagy	State Veterinary		State Veterin	2021-11-28
EPI_ISL_16652	A/guinea_fowl/Italy/21VIR1293-20/2021	A / H5N8	Europe / Italy /	Guinea fow	Bianca Zecchin	Istituto Zooprof	Zecchin, B.; F	Istituto Zoopr	2021-02-23
EPI_ISL_13175	A/Common pheasant/Sweden/SVA210224SZ0	A / H5N8	Europe / Swede	Phasianus	Siamak Zohari	National Veterin		National Vete	2021-02-24
EPI_ISL_16652	A/common_teal/Italy/20VIR7439-191/2020	A / H5N8	Europe / Italy /	Teal	Bianca Zecchin	Istituto Zooprof	Zecchin, B.; F	Istituto Zoopr	2020-11-28
EPI_ISL_88091	A/Muscovy duck/England/074477/2021	A / H5N1	Europe / United	Cairina mos	Alex Byrne (Ani	Animal and Pla		Animal and F	2021-12-21
EPI_ISL_10934	A/Common goldeneye/SVA210202SZ0464/KN	A / H5N5	Europe / Swede	Bucephala	Siamak Zohari	National Veterin		National Vete	2021-02-01
EPI_ISL_14937	A/bald eagle/Florida/W22-189/2022	A / H5N1	North America /	Haliaeetus			Poulson,R.; S		2022-03-03
EPI_ISL_14937	A/bald eagle/Georgia/W22-194A/2022	A / H5N1	North America /	Haliaeetus			Poulson,R.; S		2022-03-08
EPI_ISL_14937	A/bald eagle/South Carolina/W22-205/2022	A / H5N1	North America /	Haliaeetus			Poulson,R.; S		2022-03-01
EPI_ISL_14937	A/bald eagle/Georgia/W22-202/2022	A / H5N1	North America /	Haliaeetus			Poulson,R.; S		2022-03-01
EPI_ISL_14937	A/bald eagle/Florida/W22-195/2022	A / H5N1	North America /	Haliaeetus			Poulson,R.; S		2022-03-13
EPI_ISL_14937	A/bald eagle/Florida/W22-191/2022	A / H5N1	North America /	Haliaeetus			Poulson,R.; S		2022-03-08
EPI_ISL_14937	A/bald eagle/Georgia/W22-194B/2022	A / H5N1	North America /	Haliaeetus			Poulson,R.; S		2022-03-14
EPI_ISL_14936	A/bald eagle/Kansas/W22-197/2022	A / H5N1	North America /	Haliaeetus			Poulson,R.; S		2022-03-13
EPI_ISL_75965	A/Pica_pica/Belgium/12100_005/2020	A / H5N8	Europe / Belgiu	Pica	Steven Van Bor	Sciensano, Dep	Van Borm, St	Sciensano -	2020-11-13
EPI_ISL_11561	A/black-headed_gull/England/388256/2022	A / H5N1	Europe / United	Chroicocep	Alex Byrne (Ani	Animal and Pla		Animal and F	2022-02-03
EPI_ISL_11406	A/black-headed_gull/England/306270/2022	A / H5N1	Europe / United	Chroicocep	Alex Byrne (Ani	Animal and Pla		Animal and F	2022-01-31
EPI_ISL_11798	A/barnacle_goose/Denmark/24342-1.02/2021-	A / H5N1	Europe / Denm	Branta leuc	Charlotte Kristia	Statens Serum	Charlotte Hjul	Statens Seru	2021-10-30
EPI_ISL_11798	A/barnacle_goose/Denmark/24273-1.02/2021-	A / H5N1	Europe / Denm	Branta leuc	Charlotte Kristia	Statens Serum	Charlotte Hjul	Statens Seru	2021-10-26
EPI_ISL_11798	A/barnacle/goose_Denmark/19027-1.02/2021-	A / H5N1	Europe / Denm	Branta leuc	Charlotte Kristia	Statens Serum	Charlotte Hjul	Statens Seru	2021-04-18
EPI_ISL_11725	A/barnacle goose/Germany-MV/AI06175/2021	A / H5N1	Europe / Germa	Branta leuc	Jacqueline King	Friedrich-Loeffl		Landesamt f	2021-10-26
EPI_ISL_67611	A/Branta_leucopsis/Belgium/14735_0001/2021	A / H5N1	Europe / Belgiu	Branta leuc	Steven Van Bor	Sciensano, Dep	Van Borm, St	Sciensano -	2021-11-04
EPI_ISL_50579	A/barnacle goose/Germany-SH/AI02180/2020	A / H5N8	Europe / Germa	Branta leuc	Jacqueline King	Friedrich-Loeffl		Landeslabor	2020-11-01
EPI_ISL_50578	A/barnacle goose/Germany-SH/AI02172/2020	A / H5N8	Europe / Germa	Branta leuc	Jacqueline King	Friedrich-Loeffl		Landeslabor	2020-10-30
EPI_ISL_50578	A/barnacle goose/Germany-SH/AI02168/2020	A / H5N8	Europe / Germa	Branta leuc	Jacqueline King	Friedrich-Loeffl		Landeslabor	2020-10-28

EPI_ISL_21747	A/barnacle goose/Netherlands/21025769-002/2	A / H5N1	Europe / Nethe	Branta leuc	Rene Heutink ('	Wageningen Bi	Beerens, Nar	Wageningen	2021-03-22
EPI_ISL_12407	A/barnacle_goose/Sweden/SVA210225SZ030	A / H5N8	Europe / Swede	Branta leuc	Siamak Zohari	National Veterin		National Vet	2021-01-13
EPI_ISL_13175	A/Chicken/Sweden/SVA210223SZ0647/FB046	A / H5N8	Europe / Swede	Gallus gallu	Siamak Zohari	National Veterin		National Vet	2021-02-23
EPI_ISL_11561	A/chicken/England/012967/2022	A / H5N1	Europe / Unitec	Gallus gallu	Alex Byrne (Ani	Animal and Pla		Animal and F	2022-02-08
EPI_ISL_11561	A/chicken/England/011981/2022	A / H5N1	Europe / Unitec	Gallus gallu	Alex Byrne (Ani	Animal and Pla		Animal and F	2022-02-02
EPI_ISL_11406	A/chicken/England/000187/2022	A / H5N1	Europe / Unitec	Gallus gallu	Alex Byrne (Ani	Animal and Pla		Animal and F	2022-01-03
EPI_ISL_11406	A/chicken/England/002070/2022	A / H5N1	Europe / Unitec	Gallus gallu	Alex Byrne (Ani	Animal and Pla		Animal and F	2022-01-06
EPI_ISL_90299	A/chicken/England/057314/2021	A / H5N1	Europe / Unitec	Gallus gallu	Alex Byrne (Ani	Animal and Pla		Animal and F	2021-11-14
EPI_ISL_85154	A/chicken/Czech_Republic/23404/2021	A / H5N1	Europe / Czech	Gallus gallu	Alexander Nagy	State Veterinar	Alexander,Na	State Veterir	2021-11-25
EPI_ISL_75895	A/Gallus_gallus/Belgium/5107_002/2021	A / H5N8	Europe / Belgiu	Gallus gallu	Steven Van Bo	Sciensano, Dep	Van Born	Sciensano -	2021-04-09
EPI_ISL_13057	A/Chicken/Sweden/SVA210314SZ0002/KN066	A / H5N8	Europe / Swede	Gallus gallu	Siamak Zohari	National Veterin		National Vet	2021-03-14
EPI_ISL_13057	A/Chicken/Sweden/SVA210314SZ0001/KN066	A / H5N8	Europe / Swede	Gallus gallu	Siamak Zohari	National Veterin		National Vet	2021-03-14
EPI_ISL_12962	A/Chicken/Sweden/SVA210308SZ0214/KN057	A / H5N8	Europe / Swede	Gallus gallu	Siamak Zohari	National Veterin		National Vet	2021-03-08
EPI_ISL_11915	A/chicken/Czech Republic/1566-2/2021	A / H5N8	Europe / Czech	Gallus gallu	Alexander Nagy	State Veterinar	Nagy,A;Cerni	State Veterir	2021-01-22
EPI_ISL_10934	A/Chicken/Sweden/SVA210217SZ0001/KN001	A / H5N8	Europe / Swede	Gallus gallu	Siamak Zohari	National Veterin		National Vet	2021-02-16
EPI_ISL_97751	A/chicken/Czech Republic/1566-1/2021	A / H5N8	Europe / Czech	Gallus gallu	Alexander Nagy	State Veterinar	Nagy,A;Cerni	State Veterir	2021-01-22
EPI_ISL_40577	A/ruddy turnstone/Delaware Bay/374/2020	A / H5N9	North America /	Arenaria int			Direct Submis		2020-05-29
EPI_ISL_14838	A/Whooper swan/Sanmenxia/B497/2020	A / H5N8	Asia / China / H	Whooper sv	Zeyu Yang (Ch	Chinese Acade		Xi'an Tianlon	2020-12-12
EPI_ISL_18292	A/whooper swan/Henan/SMQ5/2020	A / H5N8	Asia / China / H	Whooper sv	Hongliang Chai	Northeast Fores		College of W	2020-11-02
EPI_ISL_70701	A/Whooper swan/Mongolia/25/2020	A / H5N6	Asia / Mongolia	Whooper sv			Jeong,S.; Otg		2020-04-30
EPI_ISL_70701	A/Whooper swan/Mongolia/24/2020	A / H5N6	Asia / Mongolia	Whooper sv			Jeong,S.; Otg		2020-04-21
EPI_ISL_62567	A/whooper swan/Inner Mongolia/w1-1/2020	A / H5N8	Asia / China / Ir	Whooper sv	Hongliang Chai	Northeast Fores		College of W	2020-10-17
EPI_ISL_85154	A/goose/Czech_Republic/22608-2/2021	A / H5N1	Europe / Czech	Anser anse	Alexander Nagy	State Veterinar	Alexander,Na	State Veterir	2021-11-18
EPI_ISL_85154	A/goose/Czech_Republic/22608-1/2021	A / H5N1	Europe / Czech	Anser anse	Alexander Nagy	State Veterinar	Alexander,Na	State Veterir	2021-11-18
EPI_ISL_13057	A/Goose/Sweden/SVA210311SZ0003/KN0001	A / H5N5	Europe / Swede	Anser anse	Siamak Zohari	National Veterin		National Vet	2021-03-10
EPI_ISL_64137	A/greater white-fronted goose/Netherlands/200	A / H5N5	Europe / Nethe	White-front	Rene Heutink ('	Wageningen Bi	Beerens, Nar	Wageningen	2020-11-07
EPI_ISL_40499	A/white-fronted goose/Germany-BB/AI00018/2	A / H5N8	Europe / Germa	White-front	Jacqueline King	Friedrich-Loeffl		Landeslabor	2020-01-16
EPI_ISL_30266	A/common eider/Sweden/SVA210617SZ0354/I	A / H5N8	Europe / Swede	Somateria s	Siamak Zohari	National Veterin		National Vet	2021-06-17
EPI_ISL_33198	A/common eider/Sweden/SVA210729SZ0323/I	A / H5N8	Europe / Swede	Somateria r	Siamak Zohari	National Veterin		National Vet	2021-07-19
EPI_ISL_30987	A/common eider/Sweden/SVA210617SZ0354/I	A / H5N8	Europe / Swede	Somateria r	Siamak Zohari	National Veterin		National Vet	2021-06-17
EPI_ISL_28204	A/common pochard/Ningxia/243/2020	A / H5N8	Asia / China / N	Aythya ferir	Hongliang Chai	Northeast Fores		College of W	2020-10-16
EPI_ISL_11798	A/Eurasian_teal/Denmark/24115-2/2021-10-16	A / H5N1	Europe / Denm	Common te	Charlotte Kristia	Statens Serum	Charlotte Hjul	Statens Seru	2021-10-16
EPI_ISL_28205	A/common teal/Ningxia/253/2020	A / H5N8	Asia / China / N	Common te	Hongliang Chai	Northeast Fores		College of W	2020-10-16
EPI_ISL_28204	A/common teal/Ningxia/245/2020	A / H5N8	Asia / China / N	Common te	Hongliang Chai	Northeast Fores		College of W	2020-10-16
EPI_ISL_28202	A/common teal/Ningxia/237/2020	A / H5N8	Asia / China / N	Common te	Hongliang Chai	Northeast Fores		College of W	2020-10-16
EPI_ISL_28202	A/common teal/Ningxia/189/2020	A / H5N8	Asia / China / N	Common te	Hongliang Chai	Northeast Fores		College of W	2020-10-15
EPI_ISL_28202	A/common teal/Ningxia/181/2020	A / H5N8	Asia / China / N	Common te	Hongliang Chai	Northeast Fores		College of W	2020-10-15
EPI_ISL_10934	A/Taiga bean goose/SVA210217SZ0303/KN00	A / H5N8	Europe / Swede	Bean goose	Siamak Zohari	National Veterin		National Vet	2021-02-16
EPI_ISL_13175	A/Turkey/Sweden/SVA210315SZ0260/FB0675	A / H5N8	Europe / Swede	Meleagris g	Siamak Zohari	National Veterin		National Vet	2021-03-15
EPI_ISL_13117	A/Chicken/BC/FAV-0488-OS/2022	A / H5N1	North America /	Meleagris g	Yohannes Berh	Canadian Food		Animal Healt	2022-05-18
EPI_ISL_12749	A/turkey/Israel/537/2021	A / H5N1	Asia / Israel / N	Meleagris g	Irina Shkoda (K	Kimron Veterina	Zuckerman,	Kimron Vete	2021-10-12
EPI_ISL_11561	A/turkey/England/016515/2022	A / H5N1	Europe / Unitec	Meleagris g	Alex Byrne (Ani	Animal and Pla		Animal and F	2022-02-20

EPI_ISL_11406	A/turkey/England/004737/2022	A / H5N1	Europe / Unitec	Meleagris g	Alex Byrne (Ani	Animal and Pla			Animal and F	2022-01-12
EPI_ISL_90299	A/turkey/England/057679/2021	A / H5N1	Europe / Unitec	Meleagris g	Alex Byrne (Ani	Animal and Pla			Animal and F	2021-11-16
EPI_ISL_10934	A/Turkey/Sweden/SVA210214SZ0002/KN0356	A / H5N8	Europe / Swede	Meleagris g	Siamak Zohari	National Veterii			National Vet	2021-02-13
EPI_ISL_66689	A/turkey/Croatia/104/2020	A / H5N8	Europe / Croati	Meleagris g	Vladimir Savić (	Croatian Veteri	Savić, Vladim		Croatian Vet	2020-11-17
EPI_ISL_14389	A/Tachybaptus_ruficollis/Belgium/1234_0008/2	A / H5N1	Europe / Belgiu	Tachybaptu	Steven Van Bo	Sciensano, Dep	Van Borm, St		Sciensano -	2022-01-24
EPI_ISL_12409	A/eastern buzzard/Tochigi/090311C/2021	A / H5N8	Asia / Japan / T	Falcon	Takehiko Saito	National Institut			National Inst	2021-03-03
EPI_ISL_11845	A/eastern buzzard/Toyama/160208T/2021	A / H5N8	Asia / Japan / T	Falcon	Takehiko Saito	National Institut			National Inst	2021-02-24
EPI_ISL_11845	A/eastern buzzard/Toyama/160208C/2021	A / H5N8	Asia / Japan / T	Falcon	Takehiko Saito	National Institut			National Inst	2021-02-24
EPI_ISL_76605	A/falcon/England/041976/2020	A / H5N8	Europe / Unitec	Falcon	Alex Byrne (Ani	Animal and Pla			Animal and F	2020-12-14
EPI_ISL_12210	A/Falco_peregrinus/Belgium/4055_0002/2022	A / H5N1	Europe / Belgiu	Falco pereç	Steven Van Bo	Sciensano, Dep	Van Borm, St		Sciensano -	2022-03-19
EPI_ISL_32982	A/peregrine falcon/Sweden/SVA210325SZ034	A / H5N4	Europe / Swede	Falco pereç	Siamak Zohari	National Veterii			National Vet	2021-03-05
EPI_ISL_77525	A/peregrine falcon/Netherlands/20020038-001/	A / H5N8	Europe / Nethe	Falco pereç	Rene Heutink ('	Wageningen Bi	Beerens, Nar		Wageningen	2020-12-13
EPI_ISL_66845	A/Peregrine falcon/Sweden/SVA201117SZ046	A / H5N8	Europe / Swede	Falco pereç	Siamak Zohari	National Veterii			National Vet	2020-11-10
EPI_ISL_64473	A/peregrine falcon/Denmark/13776-1/2020-10-	A / H5N5	Europe / Denm	Falco pereç	Charlotte Kristia	Statens Serum	Charlotte,Hjul	Liang	Statens Seru	2020-10-30
EPI_ISL_14823	A/Wild geese/Hubei/H262/2021	A / H5N8	Asia / China / H	Goose	Zeyu Yang (Ch	Chinese Acade			Xi'an Tianlon	2021-01-27
EPI_ISL_14823	A/Wild geese/Hubei/H159/2021	A / H5N8	Asia / China / H	Goose	Zeyu Yang (Ch	Chinese Acade			Xi'an Tianlon	2021-01-27
EPI_ISL_14775	A/goose/Hebei/HG12/2021	A / H5N8	Asia / China / H	Goose			Cui,H.; Zhang			2021-01
EPI_ISL_14161	A/Goose/Korea/22H277/2022	A / H5N3	Asia / Korea, R	Goose	Se-hee An (Ani	Animal and Pla			Animal and F	2022-03-15
EPI_ISL_12572	A/goose/Hunan/SE284/2022(H5N1)	A / H5N1	Asia / China / H	Goose	Pengfei Cui (Ha	Harbin Veterina	Pengfei Cui, (		Harbin Veter	2022-01-05
EPI_ISL_12572	A/goose/Guizhou/S1541/2022(H5N1)	A / H5N1	Asia / China / G	Goose	Pengfei Cui (Ha	Harbin Veterina	Pengfei Cui, (		Harbin Veter	2022-02-22
EPI_ISL_12325	A/goose/Czech Republic/25322-229/2021	A / H5N1	Europe / Czech	Goose	Alexander Nag	State Veterinar	Alexander,Na		State Veterir	2021-12-18
EPI_ISL_93770	A/goose/France/21P014207/2021	A / H5N1	Europe / Franc	Goose	Francois-Xavie	ANSES Agence			Anses (Plouf	2021-12-23
EPI_ISL_83774	A/goose/France/21P013228/2021	A / H5N1	Europe / Franc	Goose	Francois-Xavie	ANSES Agence			Anses (Plouf	2021-11-25
EPI_ISL_76226	A/Anser_Brachyrhynchus_Anser_Anser/Belgiu	A / H5N8	Europe / Belgiu	Goose	Steven Van Bo	Sciensano, Dep	Van Borm, St		Sciensano -	2020-12-03
EPI_ISL_51159	A/domestic goose/Germany-MV/AI02319/2021	A / H5N8	Europe / Germa	Goose	Jacqueline King	Friedrich-Loeffl			Landesamt f	2021-03-05
EPI_ISL_50588	A/wild goose/Germany-SH/AI02194/2020	A / H5N8	Europe / Germa	Goose	Jacqueline King	Friedrich-Loeffl			Landeslabor	2020-11-02
EPI_ISL_31020	A/goose/Poland/H1036_21RS1385-6/2021	A / H5N8	Europe / Polan	Goose	Bianca Zecchin	Istituto Zooprof	Smietanka, K		Istituto Zoopi	2021-05-03
EPI_ISL_31020	A/goose/Poland/H1044_21RS1385-4/2021	A / H5N8	Europe / Polan	Goose	Bianca Zecchin	Istituto Zooprof	Smietanka, K		Istituto Zoopi	2021-05-05
EPI_ISL_95641	A/greater_white-fronted_goose/Italy/20VIR807	A / H5N1	Europe / Italy /	Goose	Bianca Zecchin	Istituto Zooprof	Zecchin, B.; F		Istituto Zoopi	2020-11-23
EPI_ISL_83358	A/Goose/Hungary/19959/2020	A / H5N8	Europe / Hunga	Goose	Katalin Szentpá	National Food (	Katalin,Szent		National Foo	2020-04-22
EPI_ISL_83358	A/Goose/Hungary/19953/2020	A / H5N8	Europe / Hunga	Goose	Katalin Szentpá	National Food (	Katalin,Szent		National Foo	2020-04-22
EPI_ISL_83358	A/Goose/Hungary/19118/2020	A / H5N8	Europe / Hunga	Goose	Katalin Szentpá	National Food (	Katalin,Szent		National Foo	2020-04-17
EPI_ISL_83348	A/Goose/Hungary/18325/2020	A / H5N8	Europe / Hunga	Goose	Katalin Szentpá	National Food (	Katalin,Szent		National Foo	2020-04-10
EPI_ISL_68403	A/goose/Tatarstan/1730-2/2020	A / H5N8	Europe / Russia	Goose	Nikolay Zinyako	Federal Centre	N., Zinyakov;		Federal Cen	2020-10-09
EPI_ISL_62665	A/goose/Russian_Federation/Kurgan/1345-25/	A / H5N8	Europe / Russia	Goose	Alex Byrne (Ani	Animal and Pla			Federal Cen	2020-08-20
EPI_ISL_62664	A/goose/Russian_Federation/Omsk/1680-6/20	A / H5N5	Europe / Russia	Goose	Alex Byrne (Ani	Animal and Pla			Federal Cen	2020-10-02
EPI_ISL_76228	A/Anser_brachyrhynchus/Belgium/151/2020	A / H5N8	Europe / Belgiu	Anser bracl	Steven Van Bo	Sciensano, Dep	Van Borm, St		Sciensano -	2020-12-26
EPI_ISL_75987	A/Anser_brachyrhynchus/Belgium/13275_0009	A / H5N8	Europe / Belgiu	Anser bracl	Steven Van Bo	Sciensano, Dep	Van Borm, St		Sciensano -	2020-12-03
EPI_ISL_66131	A/guinea fowl/Germany-NW/AI01184/2020	A / H5N8	Europe / Germa	Guineafowl	Jacqueline King	Friedrich-Loeffl			Chemisches	2020-09-11
EPI_ISL_76230	A/Larus_argentatus/Belgium/568/2021	A / H5N8	Europe / Belgiu	Larus argei	Steven Van Bo	Sciensano, Dep	Van Borm, St		Sciensano -	2021-01-11
EPI_ISL_59454	A/European Herring Gull/Netherlands/2103741	A / H5N1	Europe / Nethe	Larus argei	Rene Heutink ('	Wageningen Bi	Beerens, Nar		Wageningen	2021-10-25
EPI_ISL_12250	A/european herring gull/Netherlands/21023937	A / H5N4	Europe / Nethe	Larus argei	Rene Heutink ('	Wageningen Bi	Beerens, Nar		Wageningen	2021-02-21

EPI_ISL_1439(	A/Larus_canus/Belgium/1668_0019/2022	A / H5N1	Europe / Belgiu	Larus canu	Steven Van Bo	Sciensano, Dep	Van Borm, St	Sciensano -	2022-01-21
EPI_ISL_1217	A/black-backed gull/Netherlands/22006711-00	A / H5N1	Europe / Nethe	Larus fuscu	Rene Heutink ('	Wageningen Bi	Beerens, Nar	Wageningen	2022-04-05
EPI_ISL_1351	A/Thalasseus sandvicensis/Belgium/7473_000	A / H5N1	Europe / Belgiu	Sterna sand	Steven Van Bo	Sciensano, Dep	Van Borm, St	Sciensano -	2022-06-04
EPI_ISL_1100	A/partridge/Bulgaria/745_22VIR778-3/2021	A / H5N1	Europe / Bulgai	Partridge	Giacomo Barbi	Istituto Zooprof	Goujgoulova,	NDRVMI (Na	2021-11-23
EPI_ISL_7778	A/pheasant/Finland/1589_21VIR7689-3/2021	A / H5N8	Europe / Finlan	Pheasant	Bianca Zecchin	Istituto Zooprof	Tammiranta,	Finnish Food	2021-02-01
EPI_ISL_7778	A/pheasant/Finland/499_21VIR7689-1/2021	A / H5N8	Europe / Finlan	Pheasant	Bianca Zecchin	Istituto Zooprof	Tammiranta,	Finnish Food	2021-01-01
EPI_ISL_6600	A/common pheasant /Sweden/SVA211104SZ0	A / H5N1	Europe / Swede	Pheasant	Siamak Zohari	National Veterin		National Vet	2021-10-29
EPI_ISL_6599	A/common pheasant /Sweden/SVA211104SZ0	A / H5N1	Europe / Swede	Pheasant	Siamak Zohari	National Veterin		National Vet	2021-10-29
EPI_ISL_8334	A/Pheasant/Hungary/18731/2020	A / H5N8	Europe / Hunga	Pheasant	Katalin Szentpá	National Food (	Katalin,Szent	National Foo	2020-04-19
EPI_ISL_1224	A/peacock/Netherlands/21022591-002/2021	A / H5N8	Europe / Nethe	Pavo crista	Rene Heutink ('	Wageningen Bi	Beerens, Nar	Wageningen	2021-02-01
EPI_ISL_1100	A/swan/Slovenia/13_22VIR777-8/2022	A / H5N1	Europe / Sloven	Swan	Giacomo Barbi	Istituto Zooprof	Slavec, B.; Ra	University of	2022-01-03
EPI_ISL_1100	A/swan/Slovenia/2073_22VIR777-6/2021	A / H5N1	Europe / Sloven	Swan	Giacomo Barbi	Istituto Zooprof	Slavec, B.; Ra	University of	2021-12-30
EPI_ISL_1100	A/swan/Slovenia/2072_22VIR777-5/2021	A / H5N1	Europe / Sloven	Swan	Giacomo Barbi	Istituto Zooprof	Slavec, B.; Ra	University of	2021-12-30
EPI_ISL_1100	A/swan/Slovenia/2060_22VIR777-4/2021	A / H5N1	Europe / Sloven	Swan	Giacomo Barbi	Istituto Zooprof	Slavec, B.; Ra	University of	2021-12-29
EPI_ISL_1100	A/swan/Slovenia/2049_22VIR777-3/2021	A / H5N1	Europe / Sloven	Swan	Giacomo Barbi	Istituto Zooprof	Slavec, B.; Ra	University of	2021-12-27
EPI_ISL_1100	A/swan/Slovenia/2041_22VIR777-2/2021	A / H5N1	Europe / Sloven	Swan	Giacomo Barbi	Istituto Zooprof	Slavec, B.; Ra	University of	2021-12-26
EPI_ISL_1043	A/swan/Germany-BW/AI00997/2022	A / H5N2	Europe / Germa	Swan	Jacqueline King	Friedrich-Loeffl		CVUA Karlsr	2022-01-30
EPI_ISL_1043	A/swan/Germany-BW/AI00996/2022	A / H5N2	Europe / Germa	Swan	Jacqueline King	Friedrich-Loeffl		CVUA Karlsr	2022-01-29
EPI_ISL_5116	A/swan/Germany-HE/AI02335/2021	A / H5N8	Europe / Germa	Swan	Jacqueline King	Friedrich-Loeffl		Landesbetrie	2021-02-26
EPI_ISL_9789	A/mute swan/Ibaraki/080203T/2021	A / H5N8	Asia / Japan / I	Swan	Takehiko Saito	National Institut		National Inst	2021-02-01
EPI_ISL_9789	A/mute swan/Ibaraki/080203C/2021	A / H5N8	Asia / Japan / I	Swan	Takehiko Saito	National Institut		National Inst	2021-02-01
EPI_ISL_7668	A/mute_swan/England/234255/2020	A / H5N1	Europe / Unitec	Swan	Alex Byrne (Ani	Animal and Pla		Animal and F	2020-12-03
EPI_ISL_6611	A/swan/Tumen/1479-2/2020	A / H5N8	Europe / Russia	Swan	Nikolay Zinyako	Federal Centre	N., Zinyakov;	Federal Cen	2020-09-10
EPI_ISL_7952	A/mute swan/Netherlands/21039824-002/2021	A / H5N1	Europe / Nethe	Cygnus olo	Rene Heutink ('	Wageningen Bi	Beerens, Nar	Wageningen	2021-11-25
EPI_ISL_6590	A/swan/France/21P012384/2021	A / H5N1	Europe / Franc	Cygnus olo	Francois-Xavie	ANSES Agence		Anses (Plouf	2021-11-08
EPI_ISL_6328	A/mute swan/Czech Republic/21312/2021	A / H5N1	Europe / Czech	Cygnus olo	Alexander Nagy	State Veterinar	Nagy,Alexand	State Veterir	2021-11-01
EPI_ISL_5804	A/Mute swan/Netherlands/21037283-002/2021	A / H5N1	Europe / Nethe	Cygnus olo	Rene Heutink ('	Wageningen Bi	Beerens, Nar	Wageningen	2021-10-24
EPI_ISL_5804	A/mute_swan/England/053054/2021	A / H5N1	Europe / Unitec	Cygnus olo	Alex Byrne (Ani	Animal and Pla		Animal and F	2021-10-24
EPI_ISL_5116	A/mute swan/Germany-HE/AI02373/2021	A / H5N8	Europe / Germa	Cygnus olo	Jacqueline King	Friedrich-Loeffl		Landesbetrie	2021-03-02
EPI_ISL_2111	A/mute_swan/Poland/MB131/2021	A / H5N8	Europe / Polan	Cygnus olo	Kamila Dziadek	National Veterin	Dziadek, K.; S	National Vet	2021-02-15
EPI_ISL_1308	A/Mute Swan/Sweden/SVA210303SZ0392/KN	A / H5N5	Europe / Swede	Cygnus olo	Siamak Zohari	National Veterin		National Vet	2021-02-26
EPI_ISL_1307	A/Mute Swan/Sweden/SVA210303SZ0380/KN	A / H5N8	Europe / Swede	Cygnus olo	Siamak Zohari	National Veterin		National Vet	2021-03-02
EPI_ISL_1307	A/Mute Swan/Sweden/SVA210304SZ0311/KN	A / H5N8	Europe / Swede	Cygnus olo	Siamak Zohari	National Veterin		National Vet	2021-03-03
EPI_ISL_1224	A/mute swan/Netherlands/21022898-002/2021	A / H5N8	Europe / Nethe	Cygnus olo	Rene Heutink ('	Wageningen Bi	Beerens, Nar	Wageningen	2021-02-04
EPI_ISL_9808	A/mute swan/Czech Republic/1410-1/2021	A / H5N8	Europe / Czech	Cygnus olo	Alexander Nagy	State Veterinar	Nagy,A;Cerni	State Veterir	2021-01-22
EPI_ISL_9563	A/mute swan/Czech Republic/1410-2/2021	A / H5N8	Europe / Czech	Cygnus olo	Alexander Nagy	State Veterinar	Nagy,A;Cerni	State Veterir	2021-01-19
EPI_ISL_7752	A/mute swan/Netherlands/20020133-001/2020	A / H5N8	Europe / Nethe	Cygnus olo	Rene Heutink ('	Wageningen Bi	Beerens, Nar	Wageningen	2020-12-15
EPI_ISL_6839	A/mute swan/Wales/048068/2020	A / H5N5	Europe / Unitec	Cygnus olo	Alex Byrne (Ani	Animal and Pla		Animal and F	2020-11-24
EPI_ISL_6839	A/mute swan/Wales/048069/2020	A / H5N5	Europe / Unitec	Cygnus olo	Alex Byrne (Ani	Animal and Pla		Animal and F	2020-11-24
EPI_ISL_6447	A/Cygnus_olor/Belgium/11956_001/2020 (H5N	A / H5N8	Europe / Belgiu	Cygnus olo	Steven Van Bo	Sciensano, Dep	Van Borm, St	Sciensano -	2020-11-04
EPI_ISL_1523	A/turkey/Spain/2755-6_22VIR8632-2/2022	A / H5N1	Europe / Spain	Turkey	Giacomo Barbi	Istituto Zooprof	Ruano, M.J.;	Laboratorio (	2022-08-04
EPI_ISL_1523	A/turkey/Spain/2996-42_22VIR8632-22/2022	A / H5N1	Europe / Spain	Turkey	Giacomo Barbi	Istituto Zooprof	Ruano, M.J.;	Laboratorio (	2022-08-29

EPI_ISL_1523	A/turkey/Spain/2996-43_22VIR8632-21/2022	A / H5N1	Europe / Spain	Turkey	Giacomo Barbi	Istituto Zooprof	Ruano, M.J.;	Laboratorio (	2022-08-29
EPI_ISL_1523	A/turkey/Spain/2755-5_22VIR8632-1/2022	A / H5N1	Europe / Spain	Turkey	Giacomo Barbi	Istituto Zooprof	Ruano, M.J.;	Laboratorio (	2022-08-04
EPI_ISL_1100	A/turkey/Italy/21VIR9520/2021	A / H5N1	Europe / Italy	Turkey	Giacomo Barbi	Istituto Zooprof	Barbierato, G	Istituto Zoop	2021-11-14
EPI_ISL_1100	A/turkey/Bulgaria/755-1_22VIR778-4/2021	A / H5N1	Europe / Bulga	Turkey	Giacomo Barbi	Istituto Zooprof	Goujgoulova,	NDRVMI (Na	2021-11-30
EPI_ISL_5142	A/turkey/Germany-NI/AI02553/2021	A / H5N8	Europe / Germa	Turkey	Jacqueline Kin	Friedrich-Loeffl		Lebensmittel	2021-03-10
EPI_ISL_5142	A/turkey/Germany-NI/AI02991/2021	A / H5N8	Europe / Germa	Turkey	Jacqueline Kin	Friedrich-Loeffl		Lebensmittel	2021-03-18
EPI_ISL_5142	A/turkey/Germany-NW/AI03104/2021	A / H5N8	Europe / Germa	Turkey	Jacqueline Kin	Friedrich-Loeffl		Chemisches	2021-03-18
EPI_ISL_5134	A/turkey/Germany-NI/AI02950/2021	A / H5N8	Europe / Germa	Turkey	Jacqueline Kin	Friedrich-Loeffl		Lebensmittel	2021-03-14
EPI_ISL_5116	A/turkey/Germany-NI/AI02349/2021	A / H5N8	Europe / Germa	Turkey	Jacqueline Kin	Friedrich-Loeffl		Lebensmittel	2021-03-07
EPI_ISL_5115	A/turkey/Germany-BB/AI02318/2021	A / H5N8	Europe / Germa	Turkey	Jacqueline Kin	Friedrich-Loeffl		Landeslabor	2021-03-04
EPI_ISL_5115	A/turkey/Germany-NI/AI02306/2021	A / H5N8	Europe / Germa	Turkey	Jacqueline Kin	Friedrich-Loeffl		Lebensmittel	2021-03-05
EPI_ISL_5115	A/turkey/Germany-NI/AI02303/2021	A / H5N8	Europe / Germa	Turkey	Jacqueline Kin	Friedrich-Loeffl		Lebensmittel	2021-03-05
EPI_ISL_5100	A/turkey/Germany-NI/AI02025/2021	A / H5N8	Europe / Germa	Turkey	Jacqueline Kin	Friedrich-Loeffl		Lebensmittel	2021-03-03
EPI_ISL_5100	A/turkey/Germany-NI/AI02013/2021	A / H5N8	Europe / Germa	Turkey	Jacqueline Kin	Friedrich-Loeffl		Lebensmittel	2021-02-28
EPI_ISL_5099	A/turkey/Germany-NI/AI01805/2021	A / H5N8	Europe / Germa	Turkey	Jacqueline Kin	Friedrich-Loeffl		Lebensmittel	2021-02-27
EPI_ISL_5099	A/turkey/Germany-NI/AI01799/2021	A / H5N8	Europe / Germa	Turkey	Jacqueline Kin	Friedrich-Loeffl		Lebensmittel	2021-02-27
EPI_ISL_5067	A/turkey/Germany-NI/AI00072/2021	A / H5N8	Europe / Germa	Turkey	Jacqueline Kin	Friedrich-Loeffl		Lebensmittel	2021-01-04
EPI_ISL_5067	A/turkey/Germany-NI/AI00063/2021	A / H5N8	Europe / Germa	Turkey	Jacqueline Kin	Friedrich-Loeffl		Lebensmittel	2021-01-04
EPI_ISL_5066	A/turkey/Germany-NI/AI03432/2020	A / H5N8	Europe / Germa	Turkey	Jacqueline Kin	Friedrich-Loeffl		Lebensmittel	2020-12-20
EPI_ISL_5066	A/turkey/Germany-NI/AI03654/2020	A / H5N8	Europe / Germa	Turkey	Jacqueline Kin	Friedrich-Loeffl		Lebensmittel	2020-12-29
EPI_ISL_5066	A/turkey/Germany-NI/AI03452/2020	A / H5N8	Europe / Germa	Turkey	Jacqueline Kin	Friedrich-Loeffl		Lebensmittel	2020-12-21
EPI_ISL_3102	A/turkey/Poland/H1289_21RS1385-20/2021	A / H5N8	Europe / Polan	Turkey	Bianca Zecchin	Istituto Zooprof	Smietanka, K	Istituto Zoop	2021-05-24
EPI_ISL_3102	A/turkey/Poland/H1168_21RS1385-15/2021	A / H5N8	Europe / Polan	Turkey	Bianca Zecchin	Istituto Zooprof	Smietanka, K	Istituto Zoop	2021-05-13
EPI_ISL_3102	A/turkey/Poland/H1184_21RS1385-14/2021	A / H5N8	Europe / Polan	Turkey	Bianca Zecchin	Istituto Zooprof	Smietanka, K	Istituto Zoop	2021-05-14
EPI_ISL_8335	A/Turkey/Hungary/19394/2020	A / H5N8	Europe / Hunga	Turkey	Katalin Szentp	National Food (	Katalin,Szent	National Foo	2020-04-18
EPI_ISL_8334	A/Turkey/Hungary/19338/2020	A / H5N8	Europe / Hunga	Turkey	Katalin Szentp	National Food (	Katalin,Szent	National Foo	2020-04-18
EPI_ISL_7660	A/turkey/England/039472/2020	A / H5N8	Europe / Unitec	Turkey	Alex Byrne (Ani	Animal and Pla		Animal and F	2020-12-04
EPI_ISL_7660	A/turkey/England/039352/2020	A / H5N8	Europe / Unitec	Turkey	Alex Byrne (Ani	Animal and Pla		Animal and F	2020-12-04
EPI_ISL_7660	A/turkey/England/038730/2020	A / H5N8	Europe / Unitec	Turkey	Alex Byrne (Ani	Animal and Pla		Animal and F	2020-12-03
EPI_ISL_7105	A/turkey/England/038115/2020	A / H5N8	Europe / Unitec	Turkey	Alex Byrne (Ani	Animal and Pla		Animal and F	2020-12-02
EPI_ISL_7105	A/turkey/England/037784/2020	A / H5N8	Europe / Unitec	Turkey	Alex Byrne (Ani	Animal and Pla		Animal and F	2020-11-28
EPI_ISL_1523	A/bearded_vulture/Spain/2116-3_22VIR8632-8	A / H5N1	Europe / Spain	Other aviar	Giacomo Barbi	Istituto Zooprof	Ruano, M.J.;	Laboratorio (	2022-06-09
EPI_ISL_1523	A/Anser_anser/Spain/810-6_22VIR8632-16/20	A / H5N1	Europe / Spain	Other aviar	Giacomo Barbi	Istituto Zooprof	Ruano, M.J.;	Laboratorio (	2022-02-23
EPI_ISL_1523	A/Anser_anser/Spain/2825-1_22VIR8632-13/2	A / H5N1	Europe / Spain	Other aviar	Giacomo Barbi	Istituto Zooprof	Ruano, M.J.;	Laboratorio (	2022-08-10
EPI_ISL_1523	A/Anser_anser/Spain/2753-3_22VIR8632-12/2	A / H5N1	Europe / Spain	Other aviar	Giacomo Barbi	Istituto Zooprof	Ruano, M.J.;	Laboratorio (	2022-08-03
EPI_ISL_1523	A/Anser_anser/Spain/2636-3_22VIR8632-11/2	A / H5N1	Europe / Spain	Other aviar	Giacomo Barbi	Istituto Zooprof	Ruano, M.J.;	Laboratorio (	2022-06-29
EPI_ISL_1523	A/red-backed-hawk/Spain/2313-1_22VIR8632-	A / H5N1	Europe / Spain	Other aviar	Giacomo Barbi	Istituto Zooprof	Ruano, M.J.;	Laboratorio (	2022-06-29
EPI_ISL_1330	A/white stork/Netherlands/22009973-002/2022	A / H5N1	Europe / Nethe	Other aviar	Rene Heutink ('	Wageningen Bi	Beerens, Nar	Wageningen	2022-05-27
EPI_ISL_1257	A/wild duck/Hebei/SD012/2021(H5N1)	A / H5N1	Asia / China / H	Other aviar	Pengfei Cui (Ha	Harbin Veterina	Pengfei Cui, (	Harbin Veter	2021-11-25
EPI_ISL_1126	A/mute_swan/Ireland/037311_22VIR1325-13/2	A / H5N1	Europe / Irelan	Other aviar	Giacomo Barbi	Istituto Zooprof	Byrne, C.; Ga	Central Vete	2021-12-22
EPI_ISL_1100	A/laying_hen/Moldova/68-2_22VIR638-2/2022	A / H5N1	Europe / Moldo	Other aviar	Giacomo Barbi	Istituto Zooprof	Arseniev, S.;	Republican (	2022-01-03
EPI_ISL_1100	A/quail/Niger/22VIR1409-30/2022	A / H5N1	Africa / Niger	Other aviar	Giacomo Barbi	Istituto Zooprof	Souley, M.M.;	Laboratoire (	2022-01-01

EPI_ISL_11007	A/quail/Niger/22VIR1409-28/2022	A / H5N1	Africa / Niger	Other aviar	Giacomo Barbi	Istituto Zooprof	Souley, M.M.;	Laboratoire (	2022-01-01
EPI_ISL_11007	A/hen/Bulgaria/757-6_22VIR778-7/2021	A / H5N1	Europe / Bulga	Other aviar	Giacomo Barbi	Istituto Zooprof	Goujgoulova,	NDRVMI (Na	2021-12-02
EPI_ISL_11007	A/hen/Bulgaria/722-1_22VIR778-1/2021	A / H5N1	Europe / Bulga	Other aviar	Giacomo Barbi	Istituto Zooprof	Goujgoulova,	NDRVMI (Na	2021-11-15
EPI_ISL_11007	A/hen/Bulgaria/854-1_22VIR778-10/2021	A / H5N1	Europe / Bulga	Other aviar	Giacomo Barbi	Istituto Zooprof	Goujgoulova,	NDRVMI (Na	2021-12-29
EPI_ISL_11007	A/seagull/Slovenia/2075_22VIR777-7/2021	A / H5N1	Europe / Slover	Other aviar	Giacomo Barbi	Istituto Zooprof	Slavec, B.; Ra	University of	2021-12-30
EPI_ISL_11007	A/rooster/Slovenia/2039_22VIR777-1/2021	A / H5N1	Europe / Slover	Other aviar	Giacomo Barbi	Istituto Zooprof	Slavec, B.; Ra	University of	2021-12-26
EPI_ISL_11007	A/laying_hen/Moldova/68-1_22VIR638-1/2022	A / H5N1	Europe / Moldo	Other aviar	Giacomo Barbi	Istituto Zooprof	Arseniev, S.;	Republican (	2022-01-03
EPI_ISL_83772	A/egret/France/21P013418/2021	A / H5N1	Europe / France	Other aviar	Francois-Xavie	ANSES Agence		Anses (Plouf	2021-12-03
EPI_ISL_79521	A/western jackdaw/Netherlands/21039297-002	A / H5N1	Europe / Nethe	Other aviar	Rene Heutink ('	Wageningen Bi	Beerens, Nar	Wageningen	2021-11-20
EPI_ISL_51262	A/eagle owl/Germany-ST/AI02542/2021	A / H5N8	Europe / Germa	Other aviar	Jacqueline King	Friedrich-Loeffl		Landesamt f	2021-03-10
EPI_ISL_51160	A/greater rhea/Germany-SN/AI02351/2021	A / H5N8	Europe / Germa	Other aviar	Jacqueline King	Friedrich-Loeffl		Landesunter	2021-03-02
EPI_ISL_33149	A/common buzzard/Sweden/SVA210323SZ042	A / H5N8	Europe / Swede	Other aviar	Siamak Zohari	National Veteri		National Vet	2021-03-14
EPI_ISL_33149	A/common buzzard/Sweden/SVA10324SZ0359	A / H5N8	Europe / Swede	Other aviar	Siamak Zohari	National Veteri		National Vet	2021-03-17
EPI_ISL_13905	A/northern goshawk/Sweden/SVA210316SZ04	A / H5N8	Europe / Swede	Other aviar	Siamak Zohari	National Veteri		National Vet	2021-03-15
EPI_ISL_13893	A/northern goshawk/Sweden/SVA210316SZ04	A / H5N8	Europe / Swede	Other aviar	Siamak Zohari	National Veteri		National Vet	2021-03-15
EPI_ISL_10954	A/Common buzzard/Sweden/SVA210212SZ02	A / H5N8	Europe / Swede	Other aviar	Siamak Zohari	National Veteri		National Vet	2021-02-11
EPI_ISL_15078	A/dolphin/Florida/22-025319-002-original/2022	A / H5N1	North America /	Other mam	Mary Lea Killiar	National Veteri	Chinh,Thanh;	National Vet	2022-03-30
EPI_ISL_15078	A/skunk/Washington/22-019274-001-original/2	A / H5N1	North America /	Other mam	Mary Lea Killiar	National Veteri	Chinh,Thanh;	National Vet	2022-06-07
EPI_ISL_15078	A/red fox/Michigan/22-018712-001-original/202	A / H5N1	North America /	Other mam	Mary Lea Killiar	National Veteri	Chinh,Thanh;	National Vet	2022-06-08
EPI_ISL_15078	A/raccoon/Washington/22-018406-002-original	A / H5N1	North America /	Other mam	Mary Lea Killiar	National Veteri	Chinh,Thanh;	National Vet	2022-06-08
EPI_ISL_15078	A/red fox/North Dakota/22-017354-001-original	A / H5N1	North America /	Other mam	Mary Lea Killiar	National Veteri	Chinh,Thanh;	National Vet	2022-06-03
EPI_ISL_15078	A/Virginia opossum/Iowa/22-016780-001-origir	A / H5N1	North America /	Other mam	Mary Lea Killiar	National Veteri	Chinh,Thanh;	National Vet	2022-05-25
EPI_ISL_15078	A/bobcat/Wisconsin/22-016051-001-original/20	A / H5N1	North America /	Other mam	Mary Lea Killiar	National Veteri	Chinh,Thanh;	National Vet	2022-05-24
EPI_ISL_15078	A/fox/Michigan/22-014536-004-original/2022	A / H5N1	North America /	Other mam	Mary Lea Killiar	National Veteri	Chinh,Thanh;	National Vet	2022-04-22
EPI_ISL_15069	A/bottlenose dolphin/UFTt2203/2022	A / H5N1	North America /	Other mam	Thomas Paul F	St. Jude Childre	Murawski, All	University of	2022-03-30
EPI_ISL_14553	A/fox/New_York/115912/2022	A / H5N1	North America /	Other mam	Brittany D Cron	Cornell Univers	Diel, Diego G	Cornell Univ	2022-05-12
EPI_ISL_14553	A/fox/New_York/103994/2022	A / H5N1	North America /	Other mam	Brittany D Cron	Cornell Univers		Cornell Univ	2022-05-10
EPI_ISL_14553	A/fox/New_York/099488/2022	A / H5N1	North America /	Other mam	Brittany D Cron	Cornell Univers	Diel, Diego G	Cornell Univ	2022-04-04
EPI_ISL_14070	A/tanuki/Hokkaido/1/2022	A / H5N1	Asia / Japan / H	Other mam	Norikazu Isoda	Graduate Scho	Yoshihiro, Sa	Graduate Sc	2022-04-01
EPI_ISL_77788	A/Red_fox/Estonia/TA2126820_21VIR10433-1	A / H5N1	Europe / Estoni	Other mam	Bianca Zecchin	Istituto Zooprof	Nurmoja, I.; V	Estonian Vet	2021-11-08
EPI_ISL_15364	A/Mute Swan/Netherlands/2/2022	A / H5N1	Europe / Nethe	Avian	Sanne Thewes	Erasmus Medic		Erasmus Me	2022-09-19
EPI_ISL_15364	A/Mallard/Netherlands/9/2022	A / H5N1	Europe / Nethe	Avian	Sanne Thewes	Erasmus Medic		Erasmus Me	2022-09-11
EPI_ISL_15364	A/Mallard/Netherlands/8/2022	A / H5N1	Europe / Nethe	Avian	Sanne Thewes	Erasmus Medic		Erasmus Me	2022-09-08
EPI_ISL_15364	A/Mallard/Netherlands/13/2022	A / H5N1	Europe / Nethe	Avian	Sanne Thewes	Erasmus Medic		Erasmus Me	2022-09-26
EPI_ISL_15364	A/Mallard/Netherlands/11/2022	A / H5N1	Europe / Nethe	Avian	Sanne Thewes	Erasmus Medic		Erasmus Me	2022-09-14
EPI_ISL_15364	A/Greylag Goose/Netherlands/17/2022	A / H5N1	Europe / Nethe	Avian	Sanne Thewes	Erasmus Medic		Erasmus Me	2022-09-16
EPI_ISL_15364	A/European Herring Gull/Netherlands/18/2022	A / H5N1	Europe / Nethe	Avian	Sanne Thewes	Erasmus Medic		Erasmus Me	2022-09-10
EPI_ISL_15267	A/Mallard/Netherlands/7/2022	A / H5N3	Europe / Nethe	Avian	Sanne Thewes	Erasmus Medic		Erasmus Me	2022-09-05
EPI_ISL_15267	A/Northern Gannet/Netherlands/6/2022	A / H5N1	Europe / Nethe	Avian	Sanne Thewes	Erasmus Medic		Erasmus Me	2022-06-21
EPI_ISL_15267	A/Northern Gannet/Netherlands/5/2022	A / H5N1	Europe / Nethe	Avian	Sanne Thewes	Erasmus Medic		Erasmus Me	2022-06-26
EPI_ISL_15267	A/European Herring Gull/Netherlands/16/2022	A / H5N1	Europe / Nethe	Avian	Sanne Thewes	Erasmus Medic		Erasmus Me	2022-09-03
EPI_ISL_15267	A/Common Tern/Netherlands/27/2022	A / H5N1	Europe / Nethe	Avian	Sanne Thewes	Erasmus Medic		Erasmus Me	2022-09-02

EPI_ISL_15267	A/Greylag Goose/Netherlands/16/2022	A / H5N1	Europe / Nethe	Avian	Sanne Thewes	Erasmus Medic		Erasmus Me	2022-09-02
EPI_ISL_15267	A/Greylag Goose/Netherlands/15/2022	A / H5N1	Europe / Nethe	Avian	Sanne Thewes	Erasmus Medic		Erasmus Me	2022-09-02
EPI_ISL_15267	A/Greylag Goose/Netherlands/14/2022	A / H5N1	Europe / Nethe	Avian	Sanne Thewes	Erasmus Medic		Erasmus Me	2022-08-31
EPI_ISL_15267	A/Eurasian Spoonbill/Netherlands/8/2022	A / H5N1	Europe / Nethe	Avian	Sanne Thewes	Erasmus Medic		Erasmus Me	2022-08-31
EPI_ISL_15267	A/Eurasian Spoonbill/Netherlands/7/2022	A / H5N1	Europe / Nethe	Avian	Sanne Thewes	Erasmus Medic		Erasmus Me	2022-06-16
EPI_ISL_15267	A/Eurasian Spoonbill/Netherlands/6/2022	A / H5N1	Europe / Nethe	Avian	Sanne Thewes	Erasmus Medic		Erasmus Me	2022-06-16
EPI_ISL_15267	A/Eurasian Spoonbill/Netherlands/5/2022	A / H5N1	Europe / Nethe	Avian	Sanne Thewes	Erasmus Medic		Erasmus Me	2022-06-16
EPI_ISL_15267	A/Lesser Black-backed Gull/Netherlands/4/202	A / H5N1	Europe / Nethe	Avian	Sanne Thewes	Erasmus Medic		Erasmus Me	2022-09-04
EPI_ISL_15078	A/chicken/Pennsylvania/22-012092-010-origina	A / H5N1	North America /	Avian	Mary Lea Killiar	National Veteri	Chinh,Thanh;	National Vet	2022-04-19
EPI_ISL_15078	A/chicken/Michigan/22-013961-001-original/20	A / H5N1	North America /	Avian	Mary Lea Killiar	National Veteri	Chinh,Thanh;	National Vet	2022-05-04
EPI_ISL_15078	A/bald eagle/Wyoming/22-013015-001-original	A / H5N1	North America /	Avian	Mary Lea Killiar	National Veteri	Chinh,Thanh;	National Vet	2022-04-22
EPI_ISL_15078	A/Cooper's hawk/Minnesota/22-012931-001-or	A / H5N1	North America /	Avian	Mary Lea Killiar	National Veteri	Chinh,Thanh;	National Vet	2022-04-26
EPI_ISL_15078	A/black vulture/Maryland/22-012407-001-origin	A / H5N1	North America /	Avian	Mary Lea Killiar	National Veteri	Chinh,Thanh;	National Vet	2022-04-21
EPI_ISL_15078	A/turkey/Iowa/22-012098-001-original/2022	A / H5N1	North America /	Avian	Mary Lea Killiar	National Veteri	Chinh,Thanh;	National Vet	2022-04-20
EPI_ISL_15078	A/Canada goose/Wyoming/22-011671-001-orig	A / H5N1	North America /	Avian	Mary Lea Killiar	National Veteri	Chinh,Thanh;	National Vet	2022-04-13
EPI_ISL_15077	A/black vulture/Florida/22-012333-001-original/	A / H5N1	North America /	Avian	Mary Lea Killiar	National Veteri	Chinh,Thanh;	National Vet	2022-04-16
EPI_ISL_15077	A/black vulture/Florida/22-012331-001-original/	A / H5N1	North America /	Avian	Mary Lea Killiar	National Veteri	Chinh,Thanh;	National Vet	2022-04-15
EPI_ISL_15069	A/Common Tern/Netherlands/26/2022	A / H5N1	Europe / Nethe	Avian	Sanne Thewes	Erasmus Medic		Erasmus Me	2022-08-12
EPI_ISL_15069	A/Mute Swan/Netherlands/1/2022	A / H5N1	Europe / Nethe	Avian	Sanne Thewes	Erasmus Medic		Erasmus Me	2022-08-10
EPI_ISL_15069	A/Black-headed gull/Netherlands/10/2022	A / H5N1	Europe / Nethe	Avian	Sanne Thewes	Erasmus Medic		Erasmus Me	2022-08-10
EPI_ISL_15069	A/European Herring Gull/Netherlands/12/2022	A / H5N1	Europe / Nethe	Avian	Sanne Thewes	Erasmus Medic		Erasmus Me	2022-08-10
EPI_ISL_15004	A/chicken/Minnesota/22-010994-001-original/2	A / H5N1	North America /	Avian	Mary Lea Killiar	National Veteri	Chinh,Thanh;	National Vet	2022-04-10
EPI_ISL_15004	A/chicken/Wisconsin/22-011334-003-original/2	A / H5N1	North America /	Avian	Mary Lea Killiar	National Veteri	Chinh,Thanh;	National Vet	2022-04-13
EPI_ISL_15004	A/chicken/Wisconsin/22-011334-002-original/2	A / H5N1	North America /	Avian	Mary Lea Killiar	National Veteri	Chinh,Thanh;	National Vet	2022-04-13
EPI_ISL_15004	A/chicken/North Dakota/22-011286-001-origina	A / H5N1	North America /	Avian	Mary Lea Killiar	National Veteri	Chinh,Thanh;	National Vet	2022-04-12
EPI_ISL_15004	A/chicken/Wisconsin/22-011213-002-original/2	A / H5N1	North America /	Avian	Mary Lea Killiar	National Veteri	Chinh,Thanh;	National Vet	2022-04-11
EPI_ISL_15004	A/chicken/Wisconsin/22-011213-001-original/2	A / H5N1	North America /	Avian	Mary Lea Killiar	National Veteri	Chinh,Thanh;	National Vet	2022-04-11
EPI_ISL_15004	A/turkey/Minnesota/22-011142-002-original/20	A / H5N1	North America /	Avian	Mary Lea Killiar	National Veteri	Chinh,Thanh;	National Vet	2022-04-11
EPI_ISL_15004	A/turkey/Minnesota/22-011142-001-original/20	A / H5N1	North America /	Avian	Mary Lea Killiar	National Veteri	Chinh,Thanh;	National Vet	2022-04-11
EPI_ISL_15004	A/turkey/Minnesota/22-011141-002-original/20	A / H5N1	North America /	Avian	Mary Lea Killiar	National Veteri	Chinh,Thanh;	National Vet	2022-04-11
EPI_ISL_15004	A/turkey/Minnesota/22-011141-001-original/20	A / H5N1	North America /	Avian	Mary Lea Killiar	National Veteri	Chinh,Thanh;	National Vet	2022-04-11
EPI_ISL_15004	A/turkey/Minnesota/22-011140-001-original/20	A / H5N1	North America /	Avian	Mary Lea Killiar	National Veteri	Chinh,Thanh;	National Vet	2022-04-11
EPI_ISL_15004	A/chicken/Montana/22-010923-004-original/20	A / H5N1	North America /	Avian	Mary Lea Killiar	National Veteri	Chinh,Thanh;	National Vet	2022-04-09
EPI_ISL_15004	A/domestic goose/Michigan/22-011121-005-ori	A / H5N1	North America /	Avian	Mary Lea Killiar	National Veteri	Chinh,Thanh;	National Vet	2022-04-11
EPI_ISL_15004	A/chicken/Michigan/22-011121-003-original/20	A / H5N1	North America /	Avian	Mary Lea Killiar	National Veteri	Chinh,Thanh;	National Vet	2022-04-11
EPI_ISL_15004	A/domestic duck/Michigan/22-011121-002-orig	A / H5N1	North America /	Avian	Mary Lea Killiar	National Veteri	Chinh,Thanh;	National Vet	2022-04-11
EPI_ISL_15004	A/chicken/Michigan/22-011121-001-original/20	A / H5N1	North America /	Avian	Mary Lea Killiar	National Veteri	Chinh,Thanh;	National Vet	2022-04-11
EPI_ISL_15004	A/turkey/Kansas/22-011119-004-original/2022	A / H5N1	North America /	Avian	Mary Lea Killiar	National Veteri	Chinh,Thanh;	National Vet	2022-04-09
EPI_ISL_15004	A/chicken/North Dakota/22-011109-001-origina	A / H5N1	North America /	Avian	Mary Lea Killiar	National Veteri	Chinh,Thanh;	National Vet	2022-04-11
EPI_ISL_15004	A/pheasant/South Dakota/22-011098-001-origi	A / H5N1	North America /	Avian	Mary Lea Killiar	National Veteri	Chinh,Thanh;	National Vet	2022-04-12
EPI_ISL_15004	A/turkey/Minnesota/22-010995-002-original/20	A / H5N1	North America /	Avian	Mary Lea Killiar	National Veteri	Chinh,Thanh;	National Vet	2022-04-09
EPI_ISL_15004	A/turkey/Minnesota/22-010995-001-original/20	A / H5N1	North America /	Avian	Mary Lea Killiar	National Veteri	Chinh,Thanh;	National Vet	2022-04-09

EPI_ISL_15004	A/chicken/Minnesota/22-010994-002-original/2	A / H5N1	North America /	Avian	Mary Lea Killiar	National Veteri	Chinh,Thanh;	National Vet	2022-04-10
EPI_ISL_15004	A/chicken/Minnesota/22-010993-004-original/2	A / H5N1	North America /	Avian	Mary Lea Killiar	National Veteri	Chinh,Thanh;	National Vet	2022-04-10
EPI_ISL_15004	A/chicken/Minnesota/22-010993-001-original/2	A / H5N1	North America /	Avian	Mary Lea Killiar	National Veteri	Chinh,Thanh;	National Vet	2022-04-10
EPI_ISL_15004	A/turkey/Minnesota/22-010992-002-original/20	A / H5N1	North America /	Avian	Mary Lea Killiar	National Veteri	Chinh,Thanh;	National Vet	2022-04-09
EPI_ISL_15004	A/turkey/Minnesota/22-010992-001-original/20	A / H5N1	North America /	Avian	Mary Lea Killiar	National Veteri	Chinh,Thanh;	National Vet	2022-04-09
EPI_ISL_15004	A/turkey/Minnesota/22-010991-002-original/20	A / H5N1	North America /	Avian	Mary Lea Killiar	National Veteri	Chinh,Thanh;	National Vet	2022-04-09
EPI_ISL_15004	A/turkey/Minnesota/22-010991-001-original/20	A / H5N1	North America /	Avian	Mary Lea Killiar	National Veteri	Chinh,Thanh;	National Vet	2022-04-09
EPI_ISL_15004	A/chicken/North Dakota/22-010977-002-origina	A / H5N1	North America /	Avian	Mary Lea Killiar	National Veteri	Chinh,Thanh;	National Vet	2022-04-10
EPI_ISL_15004	A/chicken/North Dakota/22-010977-001-origina	A / H5N1	North America /	Avian	Mary Lea Killiar	National Veteri	Chinh,Thanh;	National Vet	2022-04-10
EPI_ISL_15004	A/turkey/Wisconsin/22-010935-002-original/20	A / H5N1	North America /	Avian	Mary Lea Killiar	National Veteri	Chinh,Thanh;	National Vet	2022-04-10
EPI_ISL_15004	A/chicken/North Carolina/22-010929-002-origir	A / H5N1	North America /	Avian	Mary Lea Killiar	National Veteri	Chinh,Thanh;	National Vet	2022-04-11
EPI_ISL_15004	A/chicken/North Carolina/22-010929-001-origir	A / H5N1	North America /	Avian	Mary Lea Killiar	National Veteri	Chinh,Thanh;	National Vet	2022-04-11
EPI_ISL_15004	A/domestic duck/Minnesota/22-010928-004-ori	A / H5N1	North America /	Avian	Mary Lea Killiar	National Veteri	Chinh,Thanh;	National Vet	2022-04-11
EPI_ISL_15004	A/guinea fowl/Minnesota/22-010928-003-origin	A / H5N1	North America /	Avian	Mary Lea Killiar	National Veteri	Chinh,Thanh;	National Vet	2022-04-11
EPI_ISL_15004	A/turkey/Minnesota/22-010928-002-original/20	A / H5N1	North America /	Avian	Mary Lea Killiar	National Veteri	Chinh,Thanh;	National Vet	2022-04-11
EPI_ISL_15004	A/chicken/Minnesota/22-010928-001-original/2	A / H5N1	North America /	Avian	Mary Lea Killiar	National Veteri	Chinh,Thanh;	National Vet	2022-04-11
EPI_ISL_15004	A/chicken/Montana/22-010923-005-original/20	A / H5N1	North America /	Avian	Mary Lea Killiar	National Veteri	Chinh,Thanh;	National Vet	2022-04-09
EPI_ISL_15004	A/chicken/Nebraska/22-010905-001-original/20	A / H5N1	North America /	Avian	Mary Lea Killiar	National Veteri	Chinh,Thanh;	National Vet	2022-04-11
EPI_ISL_15004	A/Catalina macaw/Michigan/22-010848-002-or	A / H5N1	North America /	Avian	Mary Lea Killiar	National Veteri	Chinh,Thanh;	National Vet	2022-04-04
EPI_ISL_15004	A/Amazon parrot/Michigan/22-010848-001-orig	A / H5N1	North America /	Avian	Mary Lea Killiar	National Veteri	Chinh,Thanh;	National Vet	2022-04-04
EPI_ISL_15004	A/duck/Michigan/22-010845-003-original/2022	A / H5N1	North America /	Avian	Mary Lea Killiar	National Veteri	Chinh,Thanh;	National Vet	2022-04-07
EPI_ISL_15004	A/goose/Michigan/22-010845-002-original/202	A / H5N1	North America /	Avian	Mary Lea Killiar	National Veteri	Chinh,Thanh;	National Vet	2022-04-07
EPI_ISL_15004	A/goose/Michigan/22-010845-001-original/202	A / H5N1	North America /	Avian	Mary Lea Killiar	National Veteri	Chinh,Thanh;	National Vet	2022-04-07
EPI_ISL_15004	A/turkey/Minnesota/22-010773-001-original/20	A / H5N1	North America /	Avian	Mary Lea Killiar	National Veteri	Chinh,Thanh;	National Vet	2022-04-07
EPI_ISL_15004	A/turkey/Minnesota/22-010772-004-original/20	A / H5N1	North America /	Avian	Mary Lea Killiar	National Veteri	Chinh,Thanh;	National Vet	2022-04-07
EPI_ISL_15004	A/turkey/Minnesota/22-010771-003-original/20	A / H5N1	North America /	Avian	Mary Lea Killiar	National Veteri	Chinh,Thanh;	National Vet	2022-04-06
EPI_ISL_15004	A/turkey/Minnesota/22-010771-001-original/20	A / H5N1	North America /	Avian	Mary Lea Killiar	National Veteri	Chinh,Thanh;	National Vet	2022-04-06
EPI_ISL_15004	A/turkey/Minnesota/22-010770-003-original/20	A / H5N1	North America /	Avian	Mary Lea Killiar	National Veteri	Chinh,Thanh;	National Vet	2022-04-07
EPI_ISL_15004	A/turkey/Minnesota/22-010770-002-original/20	A / H5N1	North America /	Avian	Mary Lea Killiar	National Veteri	Chinh,Thanh;	National Vet	2022-04-07
EPI_ISL_15004	A/turkey/South Dakota/22-010765-001-original	A / H5N1	North America /	Avian	Mary Lea Killiar	National Veteri	Chinh,Thanh;	National Vet	2022-04-06
EPI_ISL_15004	A/chicken/Colorado/22-010668-001-original/20	A / H5N1	North America /	Avian	Mary Lea Killiar	National Veteri	Chinh,Thanh;	National Vet	2022-04-07
EPI_ISL_15004	A/goose/North Dakota/22-010657-002-original/	A / H5N1	North America /	Avian	Mary Lea Killiar	National Veteri	Chinh,Thanh;	National Vet	2022-04-06
EPI_ISL_15004	A/chicken/North Dakota/22-010657-001-origina	A / H5N1	North America /	Avian	Mary Lea Killiar	National Veteri	Chinh,Thanh;	National Vet	2022-04-06
EPI_ISL_15004	A/turkey/Minnesota/22-010654-001-original/20	A / H5N1	North America /	Avian	Mary Lea Killiar	National Veteri	Chinh,Thanh;	National Vet	2022-04-04
EPI_ISL_15004	A/turkey/Minnesota/22-010652-002-original/20	A / H5N1	North America /	Avian	Mary Lea Killiar	National Veteri	Chinh,Thanh;	National Vet	2022-04-06
EPI_ISL_15004	A/turkey/Minnesota/22-010652-001-original/20	A / H5N1	North America /	Avian	Mary Lea Killiar	National Veteri	Chinh,Thanh;	National Vet	2022-04-06
EPI_ISL_15004	A/turkey/South Dakota/22-010639-002-original	A / H5N1	North America /	Avian	Mary Lea Killiar	National Veteri	Chinh,Thanh;	National Vet	2022-04-05
EPI_ISL_15004	A/turkey/South Dakota/22-010639-001-original	A / H5N1	North America /	Avian	Mary Lea Killiar	National Veteri	Chinh,Thanh;	National Vet	2022-04-05
EPI_ISL_15004	A/Pekin duck/Indiana/22-010624-001-original/2	A / H5N1	North America /	Avian	Mary Lea Killiar	National Veteri	Chinh,Thanh;	National Vet	2022-04-07
EPI_ISL_15004	A/Pekin duck/Indiana/22-010611-001-original/2	A / H5N1	North America /	Avian	Mary Lea Killiar	National Veteri	Chinh,Thanh;	National Vet	2022-04-07
EPI_ISL_14604	A/crow/Hokkaido/0101Q061/2022	A / H5N1	Asia / Japan / H	Avian	Norikazu Isoda	Graduate Scho	Yoshihiro, SA	Graduate Sc	2022-02-14
EPI_ISL_14497	A/Eurasian Spoonbill/Netherlands/4/2022	A / H5N1	Europe / Nethe	Avian	Sanne Thewes	Erasmus Medic		Erasmus Me	2022-08-10

EPI_ISL_14233	A/Caspian Gull/Netherlands/6/2022	A / H5N1	Europe / Nethe	Avian	Sanne Thewes	Erasmus Medic		Erasmus Me	2022-07-15
EPI_ISL_14233	A/Common Tern/Netherlands/16/2022	A / H5N1	Europe / Nethe	Avian	Sanne Thewes	Erasmus Medic		Erasmus Me	2022-07-14
EPI_ISL_14233	A/Common Tern/Netherlands/15/2022	A / H5N1	Europe / Nethe	Avian	Sanne Thewes	Erasmus Medic		Erasmus Me	2022-07-14
EPI_ISL_14233	A/Common Tern/Netherlands/14/2022	A / H5N1	Europe / Nethe	Avian	Sanne Thewes	Erasmus Medic		Erasmus Me	2022-07-14
EPI_ISL_14233	A/Common Tern/Netherlands/11/2022	A / H5N1	Europe / Nethe	Avian	Sanne Thewes	Erasmus Medic		Erasmus Me	2022-07-14
EPI_ISL_14233	A/Common Tern/Netherlands/20/2022	A / H5N1	Europe / Nethe	Avian	Sanne Thewes	Erasmus Medic		Erasmus Me	2022-07-22
EPI_ISL_14233	A/Common Tern/Netherlands/19/2022	A / H5N1	Europe / Nethe	Avian	Sanne Thewes	Erasmus Medic		Erasmus Me	2022-07-22
EPI_ISL_14233	A/European Herring Gull/Netherlands/11/2022	A / H5N1	Europe / Nethe	Avian	Sanne Thewes	Erasmus Medic		Erasmus Me	2022-07-19
EPI_ISL_14233	A/Common Tern/Netherlands/18/2022	A / H5N1	Europe / Nethe	Avian	Sanne Thewes	Erasmus Medic		Erasmus Me	2022-07-15
EPI_ISL_14233	A/Common Tern/Netherlands/17/2022	A / H5N1	Europe / Nethe	Avian	Sanne Thewes	Erasmus Medic		Erasmus Me	2022-07-15
EPI_ISL_14233	A/Eurasian Spoonbill/Netherlands/3/2022	A / H5N1	Europe / Nethe	Avian	Sanne Thewes	Erasmus Medic		Erasmus Me	2022-07-24
EPI_ISL_13613	A/Common Tern/Netherlands/3/2022	A / H5N1	Europe / Nethe	Avian	Sanne Thewes	Erasmus Medic		Erasmus Me	2022-06-13
EPI_ISL_13613	A/Sandwich Tern/Netherlands/10/2022	A / H5N1	Europe / Nethe	Avian	Sanne Thewes	Erasmus Medic		Erasmus Me	2022-06-12
EPI_ISL_13613	A/Sandwich Tern/Netherlands/9/2022	A / H5N1	Europe / Nethe	Avian	Sanne Thewes	Erasmus Medic		Erasmus Me	2022-06-12
EPI_ISL_13613	A/Black-headed gull/Netherlands/9/2022	A / H5N1	Europe / Nethe	Avian	Sanne Thewes	Erasmus Medic		Erasmus Me	2022-06-10
EPI_ISL_13613	A/Black-headed gull/Netherlands/8/2022	A / H5N1	Europe / Nethe	Avian	Sanne Thewes	Erasmus Medic		Erasmus Me	2022-06-10
EPI_ISL_13613	A/Black-headed gull/Netherlands/7/2022	A / H5N1	Europe / Nethe	Avian	Sanne Thewes	Erasmus Medic		Erasmus Me	2022-06-10
EPI_ISL_13613	A/Common Tern/Netherlands/2/2022	A / H5N1	Europe / Nethe	Avian	Sanne Thewes	Erasmus Medic		Erasmus Me	2022-06-10
EPI_ISL_13613	A/Greylag Goose/Netherlands/8/2022	A / H5N1	Europe / Nethe	Avian	Sanne Thewes	Erasmus Medic		Erasmus Me	2022-06-06
EPI_ISL_13613	A/Greylag Goose/Netherlands/7/2022	A / H5N1	Europe / Nethe	Avian	Sanne Thewes	Erasmus Medic		Erasmus Me	2022-06-06
EPI_ISL_13613	A/Greylag Goose/Netherlands/6/2022	A / H5N1	Europe / Nethe	Avian	Sanne Thewes	Erasmus Medic		Erasmus Me	2022-06-06
EPI_ISL_13613	A/Greylag Goose/Netherlands/5/2022	A / H5N1	Europe / Nethe	Avian	Sanne Thewes	Erasmus Medic		Erasmus Me	2022-06-06
EPI_ISL_13613	A/Greylag Goose/Netherlands/4/2022	A / H5N1	Europe / Nethe	Avian	Sanne Thewes	Erasmus Medic		Erasmus Me	2022-06-06
EPI_ISL_13432	A/guinea fowl/New York/22-010200-003-origina	A / H5N1	North America /	Avian	Mary Lea Killian	National Veterin	Chinh,Thanh;	National Vete	2022-04-04
EPI_ISL_13432	A/chicken/New York/22-010200-002-original/20	A / H5N1	North America /	Avian	Mary Lea Killian	National Veterin	Chinh,Thanh;	National Vete	2022-04-04
EPI_ISL_13432	A/goose/Maine/22-010181-002-original/2022	A / H5N1	North America /	Avian	Mary Lea Killian	National Veterin	Chinh,Thanh;	National Vete	2022-04-04
EPI_ISL_13432	A/guinea fowl/Maine/22-010181-001-original/20	A / H5N1	North America /	Avian	Mary Lea Killian	National Veterin	Chinh,Thanh;	National Vete	2022-04-04
EPI_ISL_13432	A/chicken/Wyoming/22-010157-002-original/20	A / H5N1	North America /	Avian	Mary Lea Killian	National Veterin	Chinh,Thanh;	National Vete	2022-03-31
EPI_ISL_13432	A/chicken/Wyoming/22-010157-001-original/20	A / H5N1	North America /	Avian	Mary Lea Killian	National Veterin	Chinh,Thanh;	National Vete	2022-03-31
EPI_ISL_13432	A/chicken/Nebraska/22-010155-001-original/20	A / H5N1	North America /	Avian	Mary Lea Killian	National Veterin	Chinh,Thanh;	National Vete	2022-03-31
EPI_ISL_13432	A/turkey/North Carolina/22-010146-002-origina	A / H5N1	North America /	Avian	Mary Lea Killian	National Veterin	Chinh,Thanh;	National Vete	2022-04-04
EPI_ISL_13432	A/turkey/North Carolina/22-010146-001-origina	A / H5N1	North America /	Avian	Mary Lea Killian	National Veterin	Chinh,Thanh;	National Vete	2022-04-04
EPI_ISL_13432	A/turkey/North Carolina/22-010145-002-origina	A / H5N1	North America /	Avian	Mary Lea Killian	National Veterin	Chinh,Thanh;	National Vete	2022-03-31
EPI_ISL_13432	A/turkey/North Carolina/22-010145-001-origina	A / H5N1	North America /	Avian	Mary Lea Killian	National Veterin	Chinh,Thanh;	National Vete	2022-03-31
EPI_ISL_13432	A/turkey/North Carolina/22-010144-002-origina	A / H5N1	North America /	Avian	Mary Lea Killian	National Veterin	Chinh,Thanh;	National Vete	2022-03-31
EPI_ISL_13432	A/turkey/North Carolina/22-010144-001-origina	A / H5N1	North America /	Avian	Mary Lea Killian	National Veterin	Chinh,Thanh;	National Vete	2022-03-31
EPI_ISL_13432	A/turkey/Missouri/22-010142-001-original/2022	A / H5N1	North America /	Avian	Mary Lea Killian	National Veterin	Chinh,Thanh;	National Vete	2022-04-04
EPI_ISL_13432	A/chicken/North Dakota/22-010141-001-origina	A / H5N1	North America /	Avian	Mary Lea Killian	National Veterin	Chinh,Thanh;	National Vete	2022-04-04
EPI_ISL_13432	A/turkey/South Dakota/22-010139-002-original	A / H5N1	North America /	Avian	Mary Lea Killian	National Veterin	Chinh,Thanh;	National Vete	2022-04-04
EPI_ISL_13432	A/turkey/South Dakota/22-010139-001-original	A / H5N1	North America /	Avian	Mary Lea Killian	National Veterin	Chinh,Thanh;	National Vete	2022-04-04
EPI_ISL_13431	A/turkey/South Dakota/22-010138-002-original	A / H5N1	North America /	Avian	Mary Lea Killian	National Veterin	Chinh,Thanh;	National Vete	2022-04-04
EPI_ISL_13431	A/turkey/South Dakota/22-010138-001-original	A / H5N1	North America /	Avian	Mary Lea Killian	National Veterin	Chinh,Thanh;	National Vete	2022-04-04

EPI_ISL_13431	A/turkey/South Dakota/22-010137-002-original	A / H5N1	North America /	Avian	Mary Lea Killiar	National Veteri	Chinh,Thanh;	National Vet	2022-04-04
EPI_ISL_13431	A/turkey/South Dakota/22-010137-001-original	A / H5N1	North America /	Avian	Mary Lea Killiar	National Veteri	Chinh,Thanh;	National Vet	2022-04-04
EPI_ISL_13431	A/turkey/South Dakota/22-010135-002-original	A / H5N1	North America /	Avian	Mary Lea Killiar	National Veteri	Chinh,Thanh;	National Vet	2022-04-04
EPI_ISL_13431	A/turkey/South Dakota/22-010135-001-original	A / H5N1	North America /	Avian	Mary Lea Killiar	National Veteri	Chinh,Thanh;	National Vet	2022-04-04
EPI_ISL_13431	A/turkey/Iowa/22-010132-002-original/2022	A / H5N1	North America /	Avian	Mary Lea Killiar	National Veteri	Chinh,Thanh;	National Vet	2022-04-04
EPI_ISL_13431	A/turkey/Iowa/22-010132-001-original/2022	A / H5N1	North America /	Avian	Mary Lea Killiar	National Veteri	Chinh,Thanh;	National Vet	2022-04-04
EPI_ISL_13431	A/turkey/Minnesota/22-010127-002-original/20	A / H5N1	North America /	Avian	Mary Lea Killiar	National Veteri	Chinh,Thanh;	National Vet	2022-04-04
EPI_ISL_13431	A/turkey/Minnesota/22-010127-001-original/20	A / H5N1	North America /	Avian	Mary Lea Killiar	National Veteri	Chinh,Thanh;	National Vet	2022-04-04
EPI_ISL_13431	A/turkey/Minnesota/22-010126-002-original/20	A / H5N1	North America /	Avian	Mary Lea Killiar	National Veteri	Chinh,Thanh;	National Vet	2022-04-04
EPI_ISL_13431	A/turkey/Minnesota/22-010126-001-original/20	A / H5N1	North America /	Avian	Mary Lea Killiar	National Veteri	Chinh,Thanh;	National Vet	2022-04-04
EPI_ISL_13431	A/turkey/Minnesota/22-010125-002-original/20	A / H5N1	North America /	Avian	Mary Lea Killiar	National Veteri	Chinh,Thanh;	National Vet	2022-04-04
EPI_ISL_13431	A/turkey/Minnesota/22-010125-001-original/20	A / H5N1	North America /	Avian	Mary Lea Killiar	National Veteri	Chinh,Thanh;	National Vet	2022-04-04
EPI_ISL_13431	A/turkey/Minnesota/22-010124-002-original/20	A / H5N1	North America /	Avian	Mary Lea Killiar	National Veteri	Chinh,Thanh;	National Vet	2022-04-04
EPI_ISL_13431	A/turkey/Minnesota/22-010124-001-original/20	A / H5N1	North America /	Avian	Mary Lea Killiar	National Veteri	Chinh,Thanh;	National Vet	2022-04-04
EPI_ISL_13431	A/turkey/Minnesota/22-010123-004-original/20	A / H5N1	North America /	Avian	Mary Lea Killiar	National Veteri	Chinh,Thanh;	National Vet	2022-04-04
EPI_ISL_13431	A/turkey/Minnesota/22-010123-001-original/20	A / H5N1	North America /	Avian	Mary Lea Killiar	National Veteri	Chinh,Thanh;	National Vet	2022-04-04
EPI_ISL_13431	A/turkey/Minnesota/22-010122-002-original/20	A / H5N1	North America /	Avian	Mary Lea Killiar	National Veteri	Chinh,Thanh;	National Vet	2022-04-04
EPI_ISL_13431	A/turkey/Minnesota/22-010122-001-original/20	A / H5N1	North America /	Avian	Mary Lea Killiar	National Veteri	Chinh,Thanh;	National Vet	2022-04-04
EPI_ISL_13285	A/turkey/Iowa/22-010094-002-original/2022	A / H5N1	North America /	Avian	Mary Lea Killiar	National Veteri	Chinh,Thanh;	National Vet	2022-04-02
EPI_ISL_13285	A/turkey/Iowa/22-010094-001-original/2022	A / H5N1	North America /	Avian	Mary Lea Killiar	National Veteri	Chinh,Thanh;	National Vet	2022-04-02
EPI_ISL_13285	A/turkey/Minnesota/22-010092-003-original/20	A / H5N1	North America /	Avian	Mary Lea Killiar	National Veteri	Chinh,Thanh;	National Vet	2022-04-03
EPI_ISL_13285	A/turkey/Minnesota/22-010092-002-original/20	A / H5N1	North America /	Avian	Mary Lea Killiar	National Veteri	Chinh,Thanh;	National Vet	2022-04-03
EPI_ISL_13285	A/turkey/Minnesota/22-010085-002-original/20	A / H5N1	North America /	Avian	Mary Lea Killiar	National Veteri	Chinh,Thanh;	National Vet	2022-04-03
EPI_ISL_13285	A/turkey/Minnesota/22-010085-001-original/20	A / H5N1	North America /	Avian	Mary Lea Killiar	National Veteri	Chinh,Thanh;	National Vet	2022-04-03
EPI_ISL_13285	A/turkey/North Dakota/22-010015-002-original/	A / H5N1	North America /	Avian	Mary Lea Killiar	National Veteri	Chinh,Thanh;	National Vet	2022-04-02
EPI_ISL_13285	A/turkey/North Dakota/22-010015-001-original/	A / H5N1	North America /	Avian	Mary Lea Killiar	National Veteri	Chinh,Thanh;	National Vet	2022-04-02
EPI_ISL_13285	A/turkey/South Dakota/22-010013-002-original	A / H5N1	North America /	Avian	Mary Lea Killiar	National Veteri	Chinh,Thanh;	National Vet	2022-04-02
EPI_ISL_13285	A/turkey/South Dakota/22-010013-001-original	A / H5N1	North America /	Avian	Mary Lea Killiar	National Veteri	Chinh,Thanh;	National Vet	2022-04-02
EPI_ISL_13285	A/pheasant/Texas/22-010008-002-original/202	A / H5N1	North America /	Avian	Mary Lea Killiar	National Veteri	Chinh,Thanh;	National Vet	2022-03-29
EPI_ISL_13285	A/pheasant/Texas/22-010008-001-original/202	A / H5N1	North America /	Avian	Mary Lea Killiar	National Veteri	Chinh,Thanh;	National Vet	2022-03-29
EPI_ISL_13285	A/turkey/Minnesota/22-009999-002-original/20	A / H5N1	North America /	Avian	Mary Lea Killiar	National Veteri	Chinh,Thanh;	National Vet	2022-04-02
EPI_ISL_13285	A/turkey/Minnesota/22-010000-001-original/20	A / H5N1	North America /	Avian	Mary Lea Killiar	National Veteri	Chinh,Thanh;	National Vet	2022-04-03
EPI_ISL_13285	A/turkey/Minnesota/22-009999-001-original/20	A / H5N1	North America /	Avian	Mary Lea Killiar	National Veteri	Chinh,Thanh;	National Vet	2022-04-02
EPI_ISL_13285	A/turkey/Minnesota/22-009998-001-original/20	A / H5N1	North America /	Avian	Mary Lea Killiar	National Veteri	Chinh,Thanh;	National Vet	2022-04-02
EPI_ISL_13285	A/turkey/Minnesota/22-009997-002-original/20	A / H5N1	North America /	Avian	Mary Lea Killiar	National Veteri	Chinh,Thanh;	National Vet	2022-04-02
EPI_ISL_13285	A/turkey/Minnesota/22-009997-001-original/20	A / H5N1	North America /	Avian	Mary Lea Killiar	National Veteri	Chinh,Thanh;	National Vet	2022-04-02
EPI_ISL_13285	A/turkey/Minnesota/22-009996-001-original/20	A / H5N1	North America /	Avian	Mary Lea Killiar	National Veteri	Chinh,Thanh;	National Vet	2022-04-01
EPI_ISL_13285	A/turkey/Iowa/22-009986-002-original/2022	A / H5N1	North America /	Avian	Mary Lea Killiar	National Veteri	Chinh,Thanh;	National Vet	2022-04-02
EPI_ISL_13285	A/turkey/Iowa/22-009986-001-original/2022	A / H5N1	North America /	Avian	Mary Lea Killiar	National Veteri	Chinh,Thanh;	National Vet	2022-04-02
EPI_ISL_13285	A/chicken/Iowa/22-009985-001-original/2022	A / H5N1	North America /	Avian	Mary Lea Killiar	National Veteri	Chinh,Thanh;	National Vet	2022-04-01
EPI_ISL_13285	A/pheasant/Texas/22-009984-001-original/202	A / H5N1	North America /	Avian	Mary Lea Killiar	National Veteri	Chinh,Thanh;	National Vet	2022-03-29
EPI_ISL_13285	A/turkey/North Carolina/22-009983-008-origina	A / H5N1	North America /	Avian	Mary Lea Killiar	National Veteri	Chinh,Thanh;	National Vet	2022-03-31

EPI_ISL_13285	A/turkey/North Carolina/22-009983-007-origina	A / H5N1	North America /	Avian	Mary Lea Killiar	National Veterii	Chinh,Thanh;	National Vet	2022-03-31
EPI_ISL_13285	A/turkey/North Carolina/22-009982-002-origina	A / H5N1	North America /	Avian	Mary Lea Killiar	National Veterii	Chinh,Thanh;	National Vet	2022-03-31
EPI_ISL_13285	A/turkey/North Carolina/22-009982-001-origina	A / H5N1	North America /	Avian	Mary Lea Killiar	National Veterii	Chinh,Thanh;	National Vet	2022-03-31
EPI_ISL_13285	A/turkey/North Carolina/22-009981-004-origina	A / H5N1	North America /	Avian	Mary Lea Killiar	National Veterii	Chinh,Thanh;	National Vet	2022-03-31
EPI_ISL_13285	A/turkey/North Carolina/22-009981-001-origina	A / H5N1	North America /	Avian	Mary Lea Killiar	National Veterii	Chinh,Thanh;	National Vet	2022-03-31
EPI_ISL_13285	A/turkey/South Dakota/22-009980-002-original	A / H5N1	North America /	Avian	Mary Lea Killiar	National Veterii	Chinh,Thanh;	National Vet	2022-04-01
EPI_ISL_13285	A/turkey/South Dakota/22-009980-001-original	A / H5N1	North America /	Avian	Mary Lea Killiar	National Veterii	Chinh,Thanh;	National Vet	2022-04-01
EPI_ISL_12693	A/chicken/Iowa/22-009819-001-original/2022	A / H5N1	North America /	Avian	Mary Lea Killiar	National Veterii	Chinh,Thanh;	National Vet	2022-03-31
EPI_ISL_12514	A/Common raven/Netherlands/2/2022	A / H5N1	Europe / Nethe	Avian	Sanne Thewes	Erasmus Medic		Erasmus Me	2022-04-14
EPI_ISL_12514	A/Song Thrush/Netherlands/1/2022	A / H5N1	Europe / Nethe	Avian	Sanne Thewes	Erasmus Medic		Erasmus Me	2022-04-13
EPI_ISL_12514	A/Common raven/Netherlands/1/2022	A / H5N1	Europe / Nethe	Avian	Sanne Thewes	Erasmus Medic		Erasmus Me	2022-04-11
EPI_ISL_12514	A/Caspian Gull/Netherlands/3/2022	A / H5N1	Europe / Nethe	Avian	Sanne Thewes	Erasmus Medic		Erasmus Me	2022-04-14
EPI_ISL_12514	A/Black-headed gull/Netherlands/4/2022	A / H5N1	Europe / Nethe	Avian	Sanne Thewes	Erasmus Medic		Erasmus Me	2022-04-14
EPI_ISL_12514	A/Black-headed gull/Netherlands/3/2022	A / H5N1	Europe / Nethe	Avian	Sanne Thewes	Erasmus Medic		Erasmus Me	2022-04-14
EPI_ISL_11112	A/Caspian Gull/Netherlands/2/2022	A / H5N1	Europe / Nethe	Avian	Mark Pronk (Er	Erasmus Medic		Erasmus Me	2022-02-23
EPI_ISL_92617	A/Barnacle goose/Netherlands/6/2022	A / H5N1	Europe / Nethe	Avian	Pascal Lexmon	Erasmus Medic		Erasmus Me	2022-01-14
EPI_ISL_92617	A/Black-headed gull/Netherlands/1/2022	A / H5N1	Europe / Nethe	Avian	Pascal Lexmon	Erasmus Medic		Erasmus Me	2022-01-14
EPI_ISL_92617	A/Caspian gull/Netherlands/1/2022	A / H5N1	Europe / Nethe	Avian	Pascal Lexmon	Erasmus Medic		Erasmus Me	2022-01-14
EPI_ISL_92617	A/Grey heron/Netherlands/1/2022	A / H5N1	Europe / Nethe	Avian	Pascal Lexmon	Erasmus Medic		Erasmus Me	2022-01-14
EPI_ISL_92617	A/Barnacle goose/Netherlands/5/2022	A / H5N1	Europe / Nethe	Avian	Pascal Lexmon	Erasmus Medic		Erasmus Me	2022-01-14
EPI_ISL_92617	A/Barnacle goose/Netherlands/3/2022	A / H5N1	Europe / Nethe	Avian	Pascal Lexmon	Erasmus Medic		Erasmus Me	2022-01-11
EPI_ISL_92617	A/Barnacle goose/Netherlands/2/2022	A / H5N1	Europe / Nethe	Avian	Pascal Lexmon	Erasmus Medic		Erasmus Me	2022-01-11
EPI_ISL_92617	A/Oystercatcher/Netherlands/1/2022	A / H5N1	Europe / Nethe	Avian	Pascal Lexmon	Erasmus Medic		Erasmus Me	2022-01-11
EPI_ISL_92617	A/Great black-backed gull/1/2022	A / H5N1	Europe / Nethe	Avian	Pascal Lexmon	Erasmus Medic		Erasmus Me	2022-01-06
EPI_ISL_92617	A/Barnacle goose/Netherlands/1/2022	A / H5N1	Europe / Nethe	Avian	Pascal Lexmon	Erasmus Medic		Erasmus Me	2022-01-04
EPI_ISL_92617	A/Mallard/Netherlands/17/2021	A / H5N3	Europe / Nethe	Avian	Pascal Lexmon	Erasmus Medic		Erasmus Me	2021-12-29
EPI_ISL_92617	A/Mallard/Netherlands/16/2021	A / H5N2	Europe / Nethe	Avian	Pascal Lexmon	Erasmus Medic		Erasmus Me	2021-12-23
EPI_ISL_70505	A/western jackdaw/Sweden/SVA211111SZ037	A / H5N1	Europe / Swed	Avian	Siamak Zohari	National Veterii		National Vet	2021-11-08
EPI_ISL_66007	A/common buzzard /Sweden/SVA211104SZ03	A / H5N1	Europe / Swed	Avian	Siamak Zohari	National Veterii		National Vet	2021-10-29
EPI_ISL_13077	A/northern goshawk/Sweden/SVA210303SZ03	A / H5N8	Europe / Swed	Avian	Siamak Zohari	National Veterii		National Vet	2021-03-02
EPI_ISL_12911	A/duck/Laos/NL-2072066/2020	A / H5N1	Asia / Lao, Peo	Avian	Yunho Jang (C	Centers for Dis	Phommachar	NAHC	2020-10-16
EPI_ISL_12911	A/duck/Laos/NL-2072063/2020	A / H5N1	Asia / Lao, Peo	Avian	Yunho Jang (C	Centers for Dis	Phommachar	NAHC	2020-10-16
EPI_ISL_12911	A/duck/Laos/NL-2072060/2020	A / H5N1	Asia / Lao, Peo	Avian	Yunho Jang (C	Centers for Dis	Phommachar	NAHC	2020-10-16
EPI_ISL_12909	A/duck/Laos/NL-2072073/2020	A / H5N1	Asia / Lao, Peo	Avian	Yunho Jang (C	Centers for Dis	Phommachar	NAHC	2020-10-16
EPI_ISL_12909	A/duck/Laos/NL-2072072/2020	A / H5N1	Asia / Lao, Peo	Avian	Yunho Jang (C	Centers for Dis	Phommachar	NAHC	2020-10-16
EPI_ISL_12909	A/duck/Laos/NL-2072071/2020	A / H5N1	Asia / Lao, Peo	Avian	Yunho Jang (C	Centers for Dis	Phommachar	NAHC	2020-10-16
EPI_ISL_12909	A/duck/Laos/NL-2072070/2020	A / H5N1	Asia / Lao, Peo	Avian	Yunho Jang (C	Centers for Dis	Phommachar	NAHC	2020-10-16
EPI_ISL_12909	A/duck/Laos/NL-2072069/2020	A / H5N1	Asia / Lao, Peo	Avian	Yunho Jang (C	Centers for Dis	Phommachar	NAHC	2020-10-16
EPI_ISL_12909	A/duck/Laos/NL-2072068/2020	A / H5N1	Asia / Lao, Peo	Avian	Yunho Jang (C	Centers for Dis	Phommachar	NAHC	2020-10-16
EPI_ISL_12909	A/duck/Laos/NL-2072067/2020	A / H5N1	Asia / Lao, Peo	Avian	Yunho Jang (C	Centers for Dis	Phommachar	NAHC	2020-10-16
EPI_ISL_12909	A/duck/Laos/NL-2072065/2020	A / H5N1	Asia / Lao, Peo	Avian	Yunho Jang (C	Centers for Dis	Phommachar	NAHC	2020-10-16
EPI_ISL_12909	A/duck/Laos/NL-2072064/2020	A / H5N1	Asia / Lao, Peo	Avian	Yunho Jang (C	Centers for Dis	Phommachar	NAHC	2020-10-16

EPI_ISL_1290(	A/duck/Laos/NL-2072062/2020	A / H5N1	Asia / Lao, Peo	Avian	Yunho Jang (C	Centers for Dis	Phommachan	NAHC	2020-10-16
EPI_ISL_1290(	A/duck/Laos/NL-2072061/2020	A / H5N1	Asia / Lao, Peo	Avian	Yunho Jang (C	Centers for Dis	Phommachan	NAHC	2020-10-16
EPI_ISL_1241(	A/owl/Tochigi/090204T/2021	A / H5N8	Asia / Japan / T	Avian	Takehiko Saito	National Institut		National Inst	2021-02-16
EPI_ISL_6031(	A/Eurasian Wigeon/Netherlands/7/2020	A / H5N8	Europe / Nethe	Avian	Theo Bestebroe	Erasmus Medic		Erasmus Me	2020-10-16
EPI_ISL_6031(	A/Eurasian Wigeon/Netherlands/5/2020	A / H5N1	Europe / Nethe	Avian	Theo Bestebroe	Erasmus Medic		Erasmus Me	2020-10-16
EPI_ISL_6031(	A/Eurasian Wigeon/Netherlands/4/2020	A / H5N1	Europe / Nethe	Avian	Theo Bestebroe	Erasmus Medic		Erasmus Me	2020-10-16
EPI_ISL_6031(	A/Eurasian Wigeon/Netherlands/1/2020	A / H5N1	Europe / Nethe	Avian	Theo Bestebroe	Erasmus Medic		Erasmus Me	2020-10-16
EPI_ISL_1523(	A/chicken/Spain/2854-7_22VIR8632-4/2022	A / H5N1	Europe / Spain	Chicken	Giacomo Barbi	Istituto Zooprof	Ruano, M.J.;	Laboratorio (	2022-08-16
EPI_ISL_1523(	A/chicken/Spain/2854-5_22VIR8632-3/2022	A / H5N1	Europe / Spain	Chicken	Giacomo Barbi	Istituto Zooprof	Ruano, M.J.;	Laboratorio (	2022-08-16
EPI_ISL_1487(	A/chicken/Kazakhstan/23/2020	A / H5N8	Asia / Kazakhst	Chicken			Tabynov,K.		2020-08-01
EPI_ISL_1395(	A/chicken/Czech Republic/8028-1/2022	A / H5N1	Europe / Czech	Chicken	Alexander Nag	State Veterinar	Alexander,Na	State Veterir	2022-04-13
EPI_ISL_1395(	A/chicken/Czech Republic/2968/2022	A / H5N1	Europe / Czech	Chicken	Alexander Nag	State Veterinar	Alexander,Na	State Veterir	2022-02-04
EPI_ISL_1257(	A/chicken/Jiangxi/S40653/2021(H5N1)	A / H5N1	Asia / China / J	Chicken	Pengfei Cui (Ha	Harbin Veterina	Pengfei Cui, (	Harbin Veter	2021-12-07
EPI_ISL_1257(	A/chicken/Anhui/S1740/2022(H5N1)	A / H5N1	Asia / China / A	Chicken	Pengfei Cui (Ha	Harbin Veterina	Pengfei Cui, (	Harbin Veter	2022-03-03
EPI_ISL_1232(	A/chicken/Czech Republic/3306-2/2022	A / H5N1	Europe / Czech	Chicken	Alexander Nag	State Veterinar	Nagy,Alexand	State Veterir	2022-02-09
EPI_ISL_1100(	A/chicken/Niger/22VIR1409-9/2022	A / H5N1	Africa / Niger	Chicken	Giacomo Barbi	Istituto Zooprof	Souley, M.M.;	Laboratoire (	2022-01-01
EPI_ISL_1100(	A/chicken/Niger/22VIR1409-5/2022	A / H5N1	Africa / Niger	Chicken	Giacomo Barbi	Istituto Zooprof	Souley, M.M.;	Laboratoire (	2022-01-01
EPI_ISL_1100(	A/chicken/Niger/22VIR1409-23/2022	A / H5N1	Africa / Niger	Chicken	Giacomo Barbi	Istituto Zooprof	Souley, M.M.;	Laboratoire (	2022-01-01
EPI_ISL_1100(	A/chicken/Niger/22VIR1409-13/2022	A / H5N1	Africa / Niger	Chicken	Giacomo Barbi	Istituto Zooprof	Souley, M.M.;	Laboratoire (	2022-01-01
EPI_ISL_8377(	A/chicken/France/21P013076/2021	A / H5N1	Europe / Franc	Chicken	Francois-Xavie	ANSES Agence		Anses (Plouf	2021-11-25
EPI_ISL_6829(	A/chicken/Kagoshima/B3T/2021	A / H5N8	Asia / Japan / K	Chicken	Takehiko Saito	National Institut		National Inst	2021-11-14
EPI_ISL_6829(	A/chicken/Kagoshima/21A6T/2021	A / H5N1	Asia / Japan / K	Chicken	Takehiko Saito	National Institut		National Inst	2021-11-12
EPI_ISL_6772(	A/Chicken/Guangdong/211064-5/2021(H5N6)	A / H5N6	Asia / China / G	Chicken	Jiahao Zhang (	South China Ag	Zhang, Jiaha	South China	2021-08
EPI_ISL_6772(	A/Chicken/Guangdong/211064-4/2021(H5N6)	A / H5N6	Asia / China / G	Chicken	Jiahao Zhang (	South China Ag		South China	2021-08
EPI_ISL_6772(	A/Chicken/Guangdong/211064-3/2021(H5N6)	A / H5N6	Asia / China / G	Chicken	Jiahao Zhang (	South China Ag	Zhang, Jiaha	South China	2021-08
EPI_ISL_6772(	A/Chicken/Guangdong/211064-2/2021(H5N6)	A / H5N6	Asia / China / G	Chicken	Jiahao Zhang (	South China Ag		South China	2021-08
EPI_ISL_6772(	A/Chicken/Guangdong/211064-1/2021(H5N6)	A / H5N6	Asia / China / G	Chicken	Jiahao Zhang (	South China Ag	Zhang, Jiaha	South China	2021-08
EPI_ISL_5142(	A/chicken/Germany-ST/AI02967/2021	A / H5N8	Europe / Germa	Chicken	Jacqueline King	Friedrich-Loeffl		Landesamt f	2021-03-16
EPI_ISL_5133(	A/chicken/Germany-NI/AI02954/2021	A / H5N8	Europe / Germa	Chicken	Jacqueline King	Friedrich-Loeffl		Lebensmittel	2021-03-13
EPI_ISL_5116(	A/chicken/Germany-MV/AI02376/2021	A / H5N8	Europe / Germa	Chicken	Jacqueline King	Friedrich-Loeffl		Landesamt f	2021-03-08
EPI_ISL_5116(	A/chicken/Germany-SN/AI02353/2021	A / H5N8	Europe / Germa	Chicken	Jacqueline King	Friedrich-Loeffl		Landesunter	2021-03-05
EPI_ISL_5115(	A/chicken/Germany-NI/AI02328/2021	A / H5N8	Europe / Germa	Chicken	Jacqueline King	Friedrich-Loeffl		Lebensmittel	2021-03-06
EPI_ISL_5115(	A/chicken/Germany-SH/AI02312/2021	A / H5N8	Europe / Germa	Chicken	Jacqueline King	Friedrich-Loeffl		Landeslabor	2021-03-05
EPI_ISL_5099(	A/chicken/Germany-NI/AI01815/2021	A / H5N8	Europe / Germa	Chicken	Jacqueline King	Friedrich-Loeffl		Lebensmittel	2021-02-28
EPI_ISL_5099(	A/chicken/Germany-MV/AI01794/2021	A / H5N8	Europe / Germa	Chicken	Jacqueline King	Friedrich-Loeffl		Landesamt f	2021-02-26
EPI_ISL_5099(	A/chicken/Germany-BY/AI01617/2021	A / H5N8	Europe / Germa	Chicken	Jacqueline King	Friedrich-Loeffl		Bayrisches L	2021-02-24
EPI_ISL_5099(	A/chicken/Germany-NI/AI01599/2021	A / H5N1	Europe / Germa	Chicken	Jacqueline King	Friedrich-Loeffl		Lebensmittel	2021-02-23
EPI_ISL_3102(	A/chicken/Austria/21052483_21VIR3291-1/202	A / H5N8	Europe / Austri	Chicken	Bianca Zecchin	Istituto Zooprof	Wodak, E.; R	Istituto Zoopr	2021-04-27
EPI_ISL_3102(	A/chicken/Poland/H928_21RS1385-16/2021	A / H5N8	Europe / Polan	Chicken	Bianca Zecchin	Istituto Zooprof	Smietanka, K	Istituto Zoopr	2021-04-25
EPI_ISL_3102(	A/chicken/Poland/H1084_21RS1385-13/2021	A / H5N8	Europe / Polan	Chicken	Bianca Zecchin	Istituto Zooprof	Smietanka, K	Istituto Zoopr	2021-05-07
EPI_ISL_3102(	A/chicken/Poland/H712_21RS1385-12/2021	A / H5N8	Europe / Polan	Chicken	Bianca Zecchin	Istituto Zooprof	Smietanka, K	Istituto Zoopr	2021-04-16
EPI_ISL_3102(	A/chicken/Poland/H1161_21RS1385-10/2021	A / H5N8	Europe / Polan	Chicken	Bianca Zecchin	Istituto Zooprof	Smietanka, K	Istituto Zoopr	2021-05-13

EPI_ISL_3102(	A/chicken/Poland/H984_21RS1385-9/2021	A / H5N8	Europe / Polan	Chicken	Bianca Zecchin	Istituto Zooprof	Smietanka, K	Istituto Zoop	2021-04-28
EPI_ISL_3102(	A/chicken/Poland/H293_21RS1385-8/2021	A / H5N8	Europe / Polan	Chicken	Bianca Zecchin	Istituto Zooprof	Smietanka, K	Istituto Zoop	2021-03-23
EPI_ISL_3102(	A/chicken/Poland/H812_21RS1385-7/2021	A / H5N8	Europe / Polan	Chicken	Bianca Zecchin	Istituto Zooprof	Smietanka, K	Istituto Zoop	2021-04-20
EPI_ISL_3102(	A/chicken/Poland/H1124_21RS1385-3/2021	A / H5N8	Europe / Polan	Chicken	Bianca Zecchin	Istituto Zooprof	Smietanka, K	Istituto Zoop	2021-05-11
EPI_ISL_3102(	A/chicken/Bulgaria/298-1_21VIR4270-9/2021	A / H5N8	Europe / Bulga	Chicken	Bianca Zecchin	Istituto Zooprof	Goujgoulova,	Istituto Zoop	2021-05-05
EPI_ISL_3102(	A/chicken/Bulgaria/297-2_21VIR4270-8/2021	A / H5N8	Europe / Bulga	Chicken	Bianca Zecchin	Istituto Zooprof	Goujgoulova,	Istituto Zoop	2021-05-05
EPI_ISL_3102(	A/chicken/Bulgaria/275-4_21VIR4270-6/2021	A / H5N8	Europe / Bulga	Chicken	Bianca Zecchin	Istituto Zooprof	Goujgoulova,	Istituto Zoop	2021-04-26
EPI_ISL_3102(	A/chicken/Bulgaria/274-5_21VIR4270-4/2021	A / H5N8	Europe / Bulga	Chicken	Bianca Zecchin	Istituto Zooprof	Goujgoulova,	Istituto Zoop	2021-04-23
EPI_ISL_1184	A/chicken/Miyazaki/L9T/2021	A / H5N8	Asia / Japan / N	Chicken	Takehiko Saito	National Institut		National Inst	2021-02-24
EPI_ISL_1184	A/chicken/Miyazaki/L4T/2021	A / H5N8	Asia / Japan / N	Chicken	Takehiko Saito	National Institut		National Inst	2021-02-24
EPI_ISL_1184	A/chicken/Miyazaki/L1T/2021	A / H5N8	Asia / Japan / N	Chicken	Takehiko Saito	National Institut		National Inst	2021-02-24
EPI_ISL_1184	A/chicken/Miyazaki/L10T/2021	A / H5N8	Asia / Japan / N	Chicken	Takehiko Saito	National Institut		National Inst	2021-02-24
EPI_ISL_9960(	A/chicken/Northern_Ireland/2021-000067_21V	A / H5N8	Europe / Unitec	Chicken	Bianca Zecchin	Istituto Zooprof	McMenamy, N	AFBI - Agri-F	2021-01-05
EPI_ISL_9789(	A/chicken/Toyama/4T/2021	A / H5N8	Asia / Japan / T	Chicken	Takehiko Saito	National Institut		National Inst	2021-01-22
EPI_ISL_9789(	A/chicken/Toyama/3T/2021	A / H5N8	Asia / Japan / T	Chicken	Takehiko Saito	National Institut		National Inst	2021-01-22
EPI_ISL_9789(	A/chicken/Toyama/2T/2021	A / H5N8	Asia / Japan / T	Chicken	Takehiko Saito	National Institut		National Inst	2021-01-22
EPI_ISL_9789(	A/chicken/Toyama/1T/2021	A / H5N8	Asia / Japan / T	Chicken	Takehiko Saito	National Institut		National Inst	2021-01-22
EPI_ISL_9789(	A/chicken/Miyazaki/J6T/2021	A / H5N8	Asia / Japan / N	Chicken	Takehiko Saito	National Institut		National Inst	2021-01-30
EPI_ISL_9789(	A/chicken/Miyazaki/J4T/2021	A / H5N8	Asia / Japan / N	Chicken	Takehiko Saito	National Institut		National Inst	2021-01-30
EPI_ISL_9789(	A/chicken/Miyazaki/J3T/2021	A / H5N8	Asia / Japan / N	Chicken	Takehiko Saito	National Institut		National Inst	2021-01-30
EPI_ISL_9789(	A/chicken/Miyazaki/J12T/2021	A / H5N8	Asia / Japan / N	Chicken	Takehiko Saito	National Institut		National Inst	2021-01-30
EPI_ISL_9789(	A/chicken/Ibaraki/8T/2021	A / H5N8	Asia / Japan / I	Chicken	Takehiko Saito	National Institut		National Inst	2021-02-01
EPI_ISL_9789(	A/chicken/Ibaraki/7C/2021	A / H5N8	Asia / Japan / I	Chicken	Takehiko Saito	National Institut		National Inst	2021-02-01
EPI_ISL_9788	A/chicken/Ibaraki/3T/2021	A / H5N8	Asia / Japan / I	Chicken	Takehiko Saito	National Institut		National Inst	2021-02-01
EPI_ISL_9788	A/chicken/Ibaraki/1C/2021	A / H5N8	Asia / Japan / I	Chicken	Takehiko Saito	National Institut		National Inst	2021-02-01
EPI_ISL_9788	A/chicken/Chiba/F4T/2021	A / H5N8	Asia / Japan / C	Chicken	Takehiko Saito	National Institut		National Inst	2021-02-05
EPI_ISL_9788	A/chicken/Chiba/F3C/2021	A / H5N8	Asia / Japan / C	Chicken	Takehiko Saito	National Institut		National Inst	2021-02-05
EPI_ISL_9788	A/chicken/Chiba/F2T/2021	A / H5N8	Asia / Japan / C	Chicken	Takehiko Saito	National Institut		National Inst	2021-02-05
EPI_ISL_9788	A/chicken/Chiba/F1T/2021	A / H5N8	Asia / Japan / C	Chicken	Takehiko Saito	National Institut		National Inst	2021-02-05
EPI_ISL_9788	A/chicken/Chiba/E4T/2021	A / H5N8	Asia / Japan / C	Chicken	Takehiko Saito	National Institut		National Inst	2021-02-03
EPI_ISL_9788	A/chicken/Chiba/E3T/2021	A / H5N8	Asia / Japan / C	Chicken	Takehiko Saito	National Institut		National Inst	2021-02-03
EPI_ISL_9788	A/chicken/Chiba/E2T/2021	A / H5N8	Asia / Japan / C	Chicken	Takehiko Saito	National Institut		National Inst	2021-02-03
EPI_ISL_9788	A/chicken/Chiba/E1T/2021	A / H5N8	Asia / Japan / C	Chicken	Takehiko Saito	National Institut		National Inst	2021-02-03
EPI_ISL_7598	A/chicken/Kagawa/M2T/2020	A / H5N8	Asia / Japan / K	Chicken	Takehiko Saito	National Institut		National Inst	2020-12-22
EPI_ISL_7598	A/chicken/Kagawa/M12T/2020	A / H5N8	Asia / Japan / K	Chicken	Takehiko Saito	National Institut		National Inst	2020-12-22
EPI_ISL_7598	A/chicken/Kagawa/M11T/2020	A / H5N8	Asia / Japan / K	Chicken	Takehiko Saito	National Institut		National Inst	2020-12-22
EPI_ISL_7598	A/chicken/Kagawa/M10T/2020	A / H5N8	Asia / Japan / K	Chicken	Takehiko Saito	National Institut		National Inst	2020-12-22
EPI_ISL_7598	A/chicken/Chiba/4T/2020	A / H5N8	Asia / Japan / C	Chicken	Takehiko Saito	National Institut		National Inst	2020-12-23
EPI_ISL_7598	A/chicken/Chiba/3T/2020	A / H5N8	Asia / Japan / C	Chicken	Takehiko Saito	National Institut		National Inst	2020-12-23
EPI_ISL_7598	A/chicken/Chiba/2T/2020	A / H5N8	Asia / Japan / C	Chicken	Takehiko Saito	National Institut		National Inst	2020-12-23
EPI_ISL_7598	A/chicken/Chiba/1T/2020	A / H5N8	Asia / Japan / C	Chicken	Takehiko Saito	National Institut		National Inst	2020-12-23
EPI_ISL_7181	A/chicken/Wakayama/4T/2020	A / H5N8	Asia / Japan / V	Chicken	Takehiko Saito	National Institut		National Inst	2020-12-09

EPI_ISL_71812	A/chicken/Wakayama/3T/2020	A / H5N8	Asia / Japan / V	Chicken	Takehiko Saito	National Institut	National Inst	2020-12-09
EPI_ISL_71812	A/chicken/Wakayama/2T/2020	A / H5N8	Asia / Japan / V	Chicken	Takehiko Saito	National Institut	National Inst	2020-12-09
EPI_ISL_71812	A/chicken/Wakayama/1T/2020	A / H5N8	Asia / Japan / V	Chicken	Takehiko Saito	National Institut	National Inst	2020-12-09
EPI_ISL_71811	A/chicken/Okayama/7T/2020	A / H5N8	Asia / Japan / C	Chicken	Takehiko Saito	National Institut	National Inst	2020-12-10
EPI_ISL_71811	A/chicken/Okayama/6T/2020	A / H5N8	Asia / Japan / C	Chicken	Takehiko Saito	National Institut	National Inst	2020-12-10
EPI_ISL_71811	A/chicken/Okayama/2T/2020	A / H5N8	Asia / Japan / C	Chicken	Takehiko Saito	National Institut	National Inst	2020-12-10
EPI_ISL_71811	A/chicken/Okayama/1T/2020	A / H5N8	Asia / Japan / C	Chicken	Takehiko Saito	National Institut	National Inst	2020-12-10
EPI_ISL_71811	A/chicken/Oita/5T/2020	A / H5N8	Asia / Japan / C	Chicken	Takehiko Saito	National Institut	National Inst	2020-12-09
EPI_ISL_71811	A/chicken/Oita/3T/2020	A / H5N8	Asia / Japan / C	Chicken	Takehiko Saito	National Institut	National Inst	2020-12-09
EPI_ISL_71811	A/chicken/Oita/2T/2020	A / H5N8	Asia / Japan / C	Chicken	Takehiko Saito	National Institut	National Inst	2020-12-09
EPI_ISL_71811	A/chicken/Oita/27T/2020	A / H5N8	Asia / Japan / C	Chicken	Takehiko Saito	National Institut	National Inst	2020-12-09
EPI_ISL_71811	A/chicken/Miyazaki/E9T/2020	A / H5N8	Asia / Japan / M	Chicken	Takehiko Saito	National Institut	National Inst	2020-12-07
EPI_ISL_71811	A/chicken/Miyazaki/E6T/2020	A / H5N8	Asia / Japan / M	Chicken	Takehiko Saito	National Institut	National Inst	2020-12-07
EPI_ISL_71810	A/chicken/Miyazaki/E3T/2020	A / H5N8	Asia / Japan / M	Chicken	Takehiko Saito	National Institut	National Inst	2020-12-07
EPI_ISL_71810	A/chicken/Miyazaki/E1T/2020	A / H5N8	Asia / Japan / M	Chicken	Takehiko Saito	National Institut	National Inst	2020-12-07
EPI_ISL_71810	A/chicken/Miyazaki/D5T/2020	A / H5N8	Asia / Japan / M	Chicken	Takehiko Saito	National Institut	National Inst	2020-12-07
EPI_ISL_71810	A/chicken/Miyazaki/D5C/2020	A / H5N8	Asia / Japan / M	Chicken	Takehiko Saito	National Institut	National Inst	2020-12-07
EPI_ISL_71045	A/chicken/Nara/9T/2020	A / H5N8	Asia / Japan / N	Chicken	Takehiko Saito	National Institut	National Inst	2020-12-05
EPI_ISL_71045	A/chicken/Nara/5T/2020	A / H5N8	Asia / Japan / N	Chicken	Takehiko Saito	National Institut	National Inst	2020-12-05
EPI_ISL_71045	A/chicken/Nara/1C/2020	A / H5N8	Asia / Japan / N	Chicken	Takehiko Saito	National Institut	National Inst	2020-12-05
EPI_ISL_71045	A/chicken/Nara/12T/2020	A / H5N8	Asia / Japan / N	Chicken	Takehiko Saito	National Institut	National Inst	2020-12-05
EPI_ISL_71044	A/chicken/Miyazaki/C8T/2020	A / H5N8	Asia / Japan / M	Chicken	Takehiko Saito	National Institut	National Inst	2020-12-02
EPI_ISL_71044	A/chicken/Miyazaki/C7T/2020	A / H5N8	Asia / Japan / M	Chicken	Takehiko Saito	National Institut	National Inst	2020-12-02
EPI_ISL_71044	A/chicken/Miyazaki/C6T/2020	A / H5N8	Asia / Japan / M	Chicken	Takehiko Saito	National Institut	National Inst	2020-12-02
EPI_ISL_71044	A/chicken/Miyazaki/C4T/2020	A / H5N8	Asia / Japan / M	Chicken	Takehiko Saito	National Institut	National Inst	2020-12-02
EPI_ISL_71044	A/chicken/Miyazaki/B6T/2020	A / H5N8	Asia / Japan / M	Chicken	Takehiko Saito	National Institut	National Inst	2020-12-01
EPI_ISL_71044	A/chicken/Miyazaki/B2T/2020	A / H5N8	Asia / Japan / M	Chicken	Takehiko Saito	National Institut	National Inst	2020-12-01
EPI_ISL_71044	A/chicken/Miyazaki/B1T/2020	A / H5N8	Asia / Japan / M	Chicken	Takehiko Saito	National Institut	National Inst	2020-12-01
EPI_ISL_71044	A/chicken/Miyazaki/B10T/2020	A / H5N8	Asia / Japan / M	Chicken	Takehiko Saito	National Institut	National Inst	2020-12-01
EPI_ISL_71044	A/chicken/Miyazaki/6T/2020	A / H5N8	Asia / Japan / M	Chicken	Takehiko Saito	National Institut	National Inst	2020-11-30
EPI_ISL_71044	A/chicken/Miyazaki/4T/2020	A / H5N8	Asia / Japan / M	Chicken	Takehiko Saito	National Institut	National Inst	2020-11-30
EPI_ISL_71043	A/chicken/Miyazaki/2T/2020	A / H5N8	Asia / Japan / M	Chicken	Takehiko Saito	National Institut	National Inst	2020-11-30
EPI_ISL_71043	A/chicken/Miyazaki/1T/2020	A / H5N8	Asia / Japan / M	Chicken	Takehiko Saito	National Institut	National Inst	2020-11-30
EPI_ISL_71043	A/chicken/Hiroshima/4T/2020	A / H5N8	Asia / Japan / H	Chicken	Takehiko Saito	National Institut	National Inst	2020-12-06
EPI_ISL_71043	A/chicken/Hiroshima/3T/2020	A / H5N8	Asia / Japan / H	Chicken	Takehiko Saito	National Institut	National Inst	2020-12-06
EPI_ISL_71043	A/chicken/Hiroshima/2T/2020	A / H5N8	Asia / Japan / H	Chicken	Takehiko Saito	National Institut	National Inst	2020-12-06
EPI_ISL_71043	A/chicken/Hiroshima/1T/2020	A / H5N8	Asia / Japan / H	Chicken	Takehiko Saito	National Institut	National Inst	2020-12-06
EPI_ISL_70872	A/chicken/Kagawa/J9T/2020	A / H5N8	Asia / Japan / K	Chicken	Takehiko Saito	National Institut	National Inst	2020-12-01
EPI_ISL_70871	A/chicken/Kagawa/J9C/2020	A / H5N8	Asia / Japan / K	Chicken	Takehiko Saito	National Institut	National Inst	2020-12-01
EPI_ISL_70871	A/chicken/Kagawa/J8T/2020	A / H5N8	Asia / Japan / K	Chicken	Takehiko Saito	National Institut	National Inst	2020-12-01
EPI_ISL_70871	A/chicken/Kagawa/J8C/2020	A / H5N8	Asia / Japan / K	Chicken	Takehiko Saito	National Institut	National Inst	2020-12-01
EPI_ISL_70871	A/chicken/Kagawa/J7T/2020	A / H5N8	Asia / Japan / K	Chicken	Takehiko Saito	National Institut	National Inst	2020-12-01

EPI_ISL_70871	A/chicken/Kagawa/J7C/2020	A / H5N8	Asia / Japan / K	Chicken	Takehiko Saito	National Institut		National Inst	2020-12-01
EPI_ISL_70871	A/chicken/Kagawa/J4T/2020	A / H5N8	Asia / Japan / K	Chicken	Takehiko Saito	National Institut		National Inst	2020-12-01
EPI_ISL_70871	A/chicken/Kagawa/J4C/2020	A / H5N8	Asia / Japan / K	Chicken	Takehiko Saito	National Institut		National Inst	2020-12-01
EPI_ISL_70871	A/chicken/Kagawa/J3T/2020	A / H5N8	Asia / Japan / K	Chicken	Takehiko Saito	National Institut		National Inst	2020-12-01
EPI_ISL_70871	A/chicken/Kagawa/J3C/2020	A / H5N8	Asia / Japan / K	Chicken	Takehiko Saito	National Institut		National Inst	2020-12-01
EPI_ISL_70871	A/chicken/Kagawa/I6T/2020	A / H5N8	Asia / Japan / K	Chicken	Takehiko Saito	National Institut		National Inst	2020-12-01
EPI_ISL_70870	A/chicken/Kagawa/I6C/2020	A / H5N8	Asia / Japan / K	Chicken	Takehiko Saito	National Institut		National Inst	2020-12-01
EPI_ISL_70870	A/chicken/Kagawa/I5T/2020	A / H5N8	Asia / Japan / K	Chicken	Takehiko Saito	National Institut		National Inst	2020-12-01
EPI_ISL_70870	A/chicken/Kagawa/I5C/2020	A / H5N8	Asia / Japan / K	Chicken	Takehiko Saito	National Institut		National Inst	2020-12-01
EPI_ISL_70870	A/chicken/Kagawa/I4T/2020	A / H5N8	Asia / Japan / K	Chicken	Takehiko Saito	National Institut		National Inst	2020-12-01
EPI_ISL_70870	A/chicken/Kagawa/I4C/2020	A / H5N8	Asia / Japan / K	Chicken	Takehiko Saito	National Institut		National Inst	2020-12-01
EPI_ISL_70870	A/chicken/Kagawa/I2T/2020	A / H5N8	Asia / Japan / K	Chicken	Takehiko Saito	National Institut		National Inst	2020-12-01
EPI_ISL_70870	A/chicken/Kagawa/I2C/2020	A / H5N8	Asia / Japan / K	Chicken	Takehiko Saito	National Institut		National Inst	2020-12-01
EPI_ISL_70870	A/chicken/Kagawa/I1T/2020	A / H5N8	Asia / Japan / K	Chicken	Takehiko Saito	National Institut		National Inst	2020-12-01
EPI_ISL_70870	A/chicken/Kagawa/I1C/2020	A / H5N8	Asia / Japan / K	Chicken	Takehiko Saito	National Institut		National Inst	2020-12-01
EPI_ISL_62665	A/chicken/England/030720/2020	A / H5N8	Europe / United	Chicken	Alex Byrne (Ani	Animal and Pla		Animal and P	2020-11-02
EPI_ISL_62664	A/chicken/Russian_Federation/Omsk/1680-10/	A / H5N5	Europe / Russia	Chicken	Alex Byrne (Ani	Animal and Pla		Federal Cen	2020-10-02
EPI_ISL_60323	A/chicken/Bulgaria/380-1_20VIR3542-1/2020	A / H5N8	Europe / Bulgar	Chicken	Bianca Zecchin	Istituto Zooprof	Zecchin, B.; F	Istituto Zoop	2020-06-03
EPI_ISL_60323	A/chicken/Bulgaria/221_20VIR1725-1/2020	A / H5N2	Europe / Bulgar	Chicken	Bianca Zecchin	Istituto Zooprof	Zecchin, B.; F	Istituto Zoop	2020-03-11
EPI_ISL_60323	A/chicken/Bulgaria/201_20VIR1723-1/2020	A / H5N2	Europe / Bulgar	Chicken	Bianca Zecchin	Istituto Zooprof	Zecchin, B.; F	Istituto Zoop	2020-03-02
EPI_ISL_50396	A/chicken/Bulgaria/77_20VIR1727/2020	A / H5N2	Europe / Bulgar	Chicken			Goujgoulova,		2020-02-21
EPI_ISL_50396	A/chicken/Bulgaria/217_20VIR1724-1/2020	A / H5N8	Europe / Bulgar	Chicken			Goujgoulova,		2020-03-09
EPI_ISL_11327	A/chicken/Czech Republic/63/2022	A / H5N1	Europe / Czech	Gallus gallu	Alexander Nagy	State Veterinar	Alexander,Na	State Veterir	2022-01-03
EPI_ISL_11327	A/chicken/Czech Republic/61-2/2022	A / H5N1	Europe / Czech	Gallus gallu	Alexander Nagy	State Veterinar	Alexander,Na	State Veterir	2021-12-31
EPI_ISL_11327	A/chicken/Czech Republic/61-1/2022	A / H5N1	Europe / Czech	Gallus gallu	Alexander Nagy	State Veterinar	Alexander,Na	State Veterir	2021-12-31
EPI_ISL_58046	A/chicken/Netherlands/21037708-006010/2021	A / H5N1	Europe / Nethe	Gallus gallu	Rene Heutink ('	Wageningen Bi	Beerens, Nar	Wageningen	2021-10-30
EPI_ISL_11850	A/chicken/Astrakhan/2171-1/2020	A / H5N8	Europe / Russia	Gallus gallu	Nikolay Zinyako	Federal Centre	N., Zinyakov;	Federal Cen	2020-12-07
EPI_ISL_71053	A/chicken/Netherlands/20019422-001005/2020	A / H5N8	Europe / Nethe	Gallus gallu	Rene Heutink ('	Wageningen Bi	Beerens, Nar	Wageningen	2020-12-06
EPI_ISL_71053	A/chicken/Netherlands/20019226-001/2020	A / H5N8	Europe / Nethe	Gallus gallu	Rene Heutink ('	Wageningen Bi	Beerens, Nar	Wageningen	2020-12-02
EPI_ISL_71053	A/chicken/Netherlands/20019237-001005/2020	A / H5N8	Europe / Nethe	Gallus gallu	Rene Heutink ('	Wageningen Bi	Beerens, Nar	Wageningen	2020-12-02
EPI_ISL_69351	A/chicken/Netherlands/20019411-006010/2020	A / H5N8	Europe / Nethe	Gallus gallu	Rene Heutink ('	Wageningen Bi	Beerens, Nar	Wageningen	2020-12-04
EPI_ISL_66781	A/chicken/France/20P016448/2020	A / H5N8	Europe / France	Gallus gallu	Francois-Xavier	ANSES Agence		Anses (Plouf	2020-11-10
EPI_ISL_66117	A/chicken/Poland/448/2020	A / H5N8	Europe / Poland	Gallus gallu	Edyta Świętoń (	National Veterin	Świętoń E., Ś	National Vete	2020-11-24
EPI_ISL_15234	A/duck/Spain/2095-2_22VIR8632-5/2022	A / H5N1	Europe / Spain	Duck	Giacomo Barbie	Istituto Zooprof	Ruano, M.J.;	Laboratorio C	2022-06-10
EPI_ISL_13243	A/duck/Bangladesh/19D1819/2021	A / H5N1	Asia / Banglade	Duck	Mohammad Er	International Ce	Hossain, M.E	International	2021-12-22
EPI_ISL_13243	A/duck/Bangladesh/19D1874/2022	A / H5N1	Asia / Banglade	Duck	Mohammad Er	International Ce	Hossain, M.E	International	2022-03-09
EPI_ISL_12572	A/duck/Jiangxi/S40833/2021(H5N1)	A / H5N1	Asia / China / J	Duck	Pengfei Cui (Ha	Harbin Veterina	Pengfei Cui, (	Harbin Veter	2021-12-08
EPI_ISL_12572	A/duck/Hubei/SE220/2022(H5N1)	A / H5N1	Asia / China / H	Duck	Pengfei Cui (Ha	Harbin Veterina	Pengfei Cui, (	Harbin Veter	2022-01-10
EPI_ISL_12572	A/duck/Hubei/SE128/2022(H5N1)	A / H5N1	Asia / China / H	Duck	Pengfei Cui (Ha	Harbin Veterina	Pengfei Cui, (	Harbin Veter	2022-01-10
EPI_ISL_12572	A/duck/Hubei/S4465/2021(H5N1)	A / H5N1	Asia / China / H	Duck	Pengfei Cui (Ha	Harbin Veterina	Pengfei Cui, (	Harbin Veter	2021-11-27
EPI_ISL_12572	A/duck/Guizhou/S1321/2022(H5N1)	A / H5N1	Asia / China / G	Duck	Pengfei Cui (Ha	Harbin Veterina	Pengfei Cui, (	Harbin Veter	2022-02-22
EPI_ISL_12572	A/duck/Guangdong/S4525/2021(H5N1)	A / H5N1	Asia / China / G	Duck	Pengfei Cui (Ha	Harbin Veterina	Pengfei Cui, (	Harbin Veter	2021-12-08

EPI_ISL_12572	A/duck/Guangdong/S4518/2021(H5N1)	A / H5N1	Asia / China / G	Duck	Pengfei Cui (Ha	Harbin Veterina	Pengfei Cui, (	Harbin Veter	2021-12-08
EPI_ISL_11212	A/duck/Bangladesh/18D1811/2022	A / H5N3	Asia / Banglade	Duck	Mohammad Er	International Ce	Hossain, M.E	International	2022-01-10
EPI_ISL_11211	A/duck/Bangladesh/19D1818/2021	A / H5N1	Asia / Banglade	Duck	Mohammad Er	International Ce	Hossain, M.E	International	2021-12-22
EPI_ISL_11211	A/duck/Bangladesh/17D1839/2022	A / H5N3	Asia / Banglade	Duck	Mohammad Er	International Ce	Hossain, M.E	International	2022-01-30
EPI_ISL_11007	A/duck/Bulgaria/756-4_22VIR778-6/2021	A / H5N1	Europe / Bulgar	Duck	Giacomo Barbi	Istituto Zooprof	Goujgoulova,	NDRVMI (Na	2021-12-01
EPI_ISL_79758	A/duck/Italy/21VIR8022-1/2021	A / H5N3	Europe / Italy /	Duck	Adelaide Milani	Istituto Zooprof	Milani, A.; Fus	Istituto Zoopr	2021-09-23
EPI_ISL_74587	A/Duck/Shandong/21644-1/2021(H5N8)	A / H5N8	Asia / China / S	Duck	Jiahao Zhang (	South China Ag	Zhang, Jiahao	South China	2021-03
EPI_ISL_54035	A/Eurasian wigeon/Germany-SH/AI05948/2021	A / H5N1	Europe / Germa	Duck	Jacqueline King	Friedrich-Loeffl		Landeslabor	2021-10-14
EPI_ISL_51413	A/domestic duck/Germany-NI/AI02660/2021	A / H5N8	Europe / Germa	Duck	Jacqueline King	Friedrich-Loeffl		Lebensmittel	2021-03-14
EPI_ISL_50996	A/domestic duck/Germany-BB/AI01443/2021	A / H5N8	Europe / Germa	Duck	Jacqueline King	Friedrich-Loeffl		Landeslabor	2021-02-17
EPI_ISL_50994	A/domestic duck/Germany-BB/AI01435/2021	A / H5N8	Europe / Germa	Duck	Jacqueline King	Friedrich-Loeffl		Landeslabor	2021-02-17
EPI_ISL_50679	A/domestic duck/Germany-NI/AI00079/2021	A / H5N8	Europe / Germa	Duck	Jacqueline King	Friedrich-Loeffl		Lebensmittel	2021-01-04
EPI_ISL_31020	A/duck/Poland/H514_21RS1385-18/2021	A / H5N8	Europe / Poland	Duck	Bianca Zecchin	Istituto Zooprof	Smietanka, K	Istituto Zoopr	2021-04-07
EPI_ISL_31020	A/duck/Poland/H515_21RS1385-17/2021	A / H5N8	Europe / Poland	Duck	Bianca Zecchin	Istituto Zooprof	Smietanka, K	Istituto Zoopr	2021-04-07
EPI_ISL_31020	A/duck/Poland/H1029_21RS1385-5/2021	A / H5N8	Europe / Poland	Duck	Bianca Zecchin	Istituto Zooprof	Smietanka, K	Istituto Zoopr	2021-05-01
EPI_ISL_31020	A/duck/Poland/H542_21RS1385-2/2021	A / H5N8	Europe / Poland	Duck	Bianca Zecchin	Istituto Zooprof	Smietanka, K	Istituto Zoopr	2021-04-09
EPI_ISL_31020	A/duck/Poland/H160_21RS1385-1/2021	A / H5N8	Europe / Poland	Duck	Bianca Zecchin	Istituto Zooprof	Smietanka, K	Istituto Zoopr	2021-03-05
EPI_ISL_12409	A/mallard/Novosibirsk region/3509k/2020	A / H5N8	Europe / Russia	Duck	Takehiko Saito	National Institut	Mine, J.; Uchi	Research Ins	2020-08-29
EPI_ISL_97891	A/duck/Chiba/D1-3T/2021	A / H5N8	Asia / Japan / C	Duck	Takehiko Saito	National Institut		National Inst	2021-01-23
EPI_ISL_97891	A/duck/Chiba/C5T/2021	A / H5N8	Asia / Japan / C	Duck	Takehiko Saito	National Institut		National Inst	2021-01-20
EPI_ISL_97891	A/duck/Chiba/C3T/2021	A / H5N8	Asia / Japan / C	Duck	Takehiko Saito	National Institut		National Inst	2021-01-20
EPI_ISL_97891	A/duck/Chiba/C2T/2021	A / H5N8	Asia / Japan / C	Duck	Takehiko Saito	National Institut		National Inst	2021-01-20
EPI_ISL_97891	A/duck/Chiba/C1T/2021	A / H5N8	Asia / Japan / C	Duck	Takehiko Saito	National Institut		National Inst	2021-01-20
EPI_ISL_83357	A/Duck/Hungary/18414/2020	A / H5N8	Europe / Hunga	Duck	Katalin Szentpá	National Food (	Katalin,Szent	National Foo	2020-04-14
EPI_ISL_80825	A/Duck/Hungary/18358/2020	A / H5N8	Europe / Hunga	Duck	Katalin Szentpá	National Food (	Katalin,Szent	National Foo	2020-04-11
EPI_ISL_71051	A/whistling_duck/England/035643/2020	A / H5N8	Europe / United	Duck	Alex Byrne (Ani	Animal and Pla		Animal and P	2020-11-19
EPI_ISL_63709	A/duck/Chelyabinsk/1207-1/2020	A / H5N8	Europe / Russia	Duck	Nikolay Zinyako	Federal Centre	N., Zinyakov;	Federal Cen	2020-07-31
EPI_ISL_62665	A/duck/Russian_Federation/Omsk/1328-2/2020	A / H5N8	Europe / Russia	Duck	Alex Byrne (Ani	Animal and Pla		Federal Cen	2020-08-17
EPI_ISL_62664	A/duck/Russian_Federation/Saratov/1578-2/20	A / H5N8	Europe / Russia	Duck	Alex Byrne (Ani	Animal and Pla		Federal Cen	2020-09-18
EPI_ISL_60323	A/mule_duck/Bulgaria/147_20VIR1721-1/2020	A / H5N8	Europe / Bulgar	Duck	Bianca Zecchin	Istituto Zooprof	Zecchin, B.; F	Istituto Zoopr	2020-02-21
EPI_ISL_60323	A/mule_duck/Bulgaria/50-506_20VIR1414-1/20	A / H5N2	Europe / Bulgar	Duck	Bianca Zecchin	Istituto Zooprof	Zecchin, B.; F	Istituto Zoopr	2020-02-11
EPI_ISL_60323	A/duck/Bulgaria/78-4t_20VIR1416-3/2020	A / H5N8	Europe / Bulgar	Duck	Bianca Zecchin	Istituto Zooprof	Zecchin, B.; F	Istituto Zoopr	2020-02-21
EPI_ISL_14776	A/duck/Bangladesh/46160/2020	A / H5N1	Asia / Banglade	Anas sp.			Barman,S.; T		2020-10-22
EPI_ISL_14776	A/duck/Bangladesh/46162/2020	A / H5N1	Asia / Banglade	Anas sp.			Barman,S.; T		2020-10-22
EPI_ISL_14776	A/duck/Bangladesh/46252/2020	A / H5N1	Asia / Banglade	Anas sp.			Barman,S.; T		2020-12-21
EPI_ISL_14776	A/duck/Bangladesh/46244/2020	A / H5N1	Asia / Banglade	Anas sp.			Barman,S.; T		2020-12-21
EPI_ISL_14776	A/duck/Bangladesh/46156/2020	A / H5N1	Asia / Banglade	Anas sp.			Barman,S.; T		2020-10-22
EPI_ISL_14776	A/duck/Bangladesh/46161/2020	A / H5N1	Asia / Banglade	Anas sp.			Barman,S.; T		2020-10-22
EPI_ISL_14775	A/duck/Bangladesh/46157/2020	A / H5N1	Asia / Banglade	Anas sp.			Barman,S.; T		2020-10-22
EPI_ISL_14775	A/duck/Bangladesh/46095/2020	A / H5N1	Asia / Banglade	Anas sp.			Barman,S.; T		2020-10-22
EPI_ISL_14775	A/duck/Bangladesh/46158/2020	A / H5N1	Asia / Banglade	Anas sp.			Barman,S.; T		2020-10-22
EPI_ISL_14775	A/duck/Bangladesh/46247/2020	A / H5N1	Asia / Banglade	Anas sp.			Barman,S.; T		2020-12-21

EPI_ISL_95728	A/duck/Vietnam/QN6519/2020	A / H5N6	Asia / Vietnam	Anas sp.			Kawaoka,Y.;		2020-08-21
EPI_ISL_95727	A/duck/Vietnam/HN6611/2020	A / H5N6	Asia / Vietnam	Anas sp.			Kawaoka,Y.;		2020-09-29
EPI_ISL_64413	A/wild duck/Omsk/01111/2020	A / H5N8	Europe / Russia	Anas sp.	Natalia Goncha	State Research	Natalia,Goncl	State Resea	2020-08-17
EPI_ISL_21756	A/common buzzard/Netherlands/21021187-001	A / H5N8	Europe / Nethe	Buteo buteo	Rene Heutink (\	Wageningen Bi	Beerens, Nar	Wageningen	2021-01-04
EPI_ISL_12249	A/common buzzard/Netherlands/21022834-002	A / H5N1	Europe / Nethe	Buteo buteo	Rene Heutink (\	Wageningen Bi	Beerens, Nar	Wageningen	2021-02-01
EPI_ISL_26520	A/White-Tailed Eagle/Sweden/SVA210528SZ0	A / H5N1	Europe / Swede	Halietus alb	Siamak Zohari	National Veterii		National Vet	2021-05-25
EPI_ISL_75903	A/Branta_canadensis/Belgium/500/2021	A / H5N8	Europe / Belgiu	Branta cana	Steven Van Bo	Sciensano, Dep	Van Borm, St	Sciensano -	2021-01-11
EPI_ISL_32901	A/Canada Goose/Sweden/SVA210330SZ0426	A / H5N8	Europe / Swede	Branta cana	Siamak Zohari	National Veterii		National Vet	2021-03-24
EPI_ISL_13077	A/Canada goose/Sweden/SVA210305SZ0255/	A / H5N8	Europe / Swede	Branta cana	Siamak Zohari	National Veterii		National Vet	2021-02-28
EPI_ISL_13077	A/Canade goose/Sweden/SVA210304SZ0320/	A / H5N8	Europe / Swede	Branta cana	Siamak Zohari	National Veterii		National Vet	2021-02-21
EPI_ISL_10953	A/Canada goose/Sweden/SVA210209SZ0420/	A / H5N8	Europe / Swede	Branta cana	Siamak Zohari	National Veterii		National Vet	2021-02-07
EPI_ISL_71050	A/Canada_goose/England/032697/2020	A / H5N8	Europe / Unitec	Branta cana	Alex Byrne (Ani	Animal and Pla		Animal and F	2020-11-03
EPI_ISL_48059	A/seal/Germany-SH/AI05379/2021	A / H5N8	Europe / Germa	Seal	Jacqueline King	Friedrich-Loeffl		Tierärztliche	2021-08-19
EPI_ISL_48058	A/seal/Germany-SH/AI05377/2021	A / H5N8	Europe / Germa	Seal	Jacqueline King	Friedrich-Loeffl		Tierärztliche	2021-08-18
EPI_ISL_48048	A/seal/Germany-SH/AI05373/2021	A / H5N8	Europe / Germa	Seal	Jacqueline King	Friedrich-Loeffl		Landeslabor	2021-08-15
EPI_ISL_14390	A/Phalacrocorax_carbo/Belgium/1734_0002/20	A / H5N1	Europe / Belgiu	Wild bird	Steven Van Bo	Sciensano, Dep	Van Borm, St	Sciensano -	2022-02-02
EPI_ISL_83770	A/pelican/France/21P013720/2021	A / H5N1	Europe / France	Wild bird	Francois-Xavie	ANSES Agence		Anses (Plouf	2021-12-15
EPI_ISL_77787	A/common_eider/Norway/FU458_21VIR7634-4	A / H5N8	Europe / Norwa	Wild bird	Bianca Zecchin	Istituto Zooprof	Gjerset, B.; M	Norwegian V	2021-07-01
EPI_ISL_77787	A/common_eider/Norway/FU453_21VIR7634-3	A / H5N8	Europe / Norwa	Wild bird	Bianca Zecchin	Istituto Zooprof	Gjerset, B.; M	Norwegian V	2021-06-23
EPI_ISL_77787	A/mute_swan/Norway/FU452_21VIR7634-2/20	A / H5N8	Europe / Norwa	Wild bird	Bianca Zecchin	Istituto Zooprof	Gjerset, B.; M	Norwegian V	2021-06-21
EPI_ISL_77787	A/greylag_goose/Norway/V294_21VIR7634-1/2	A / H5N8	Europe / Norwa	Wild bird	Bianca Zecchin	Istituto Zooprof	Gjerset, B.; M	Norwegian V	2021-06-14
EPI_ISL_77787	A/barnacle_goose/Finland/6955_21VIR7689-9/	A / H5N1	Europe / Finlan	Wild bird	Bianca Zecchin	Istituto Zooprof	Tammiranta,	Finnish Fooc	2021-06-01
EPI_ISL_77787	A/barnacle_goose/Finland//6378_21VIR7689-7	A / H5N1	Europe / Finlan	Wild bird	Bianca Zecchin	Istituto Zooprof	Tammiranta,	Finnish Fooc	2021-05-01
EPI_ISL_77787	A/barnacle_goose/Finland/6247_21VIR7689-6/	A / H5N1	Europe / Finlan	Wild bird	Bianca Zecchin	Istituto Zooprof	Tammiranta,	Finnish Fooc	2021-05-01
EPI_ISL_77787	A/mute_swan/Finland/1325_21VIR7689-2/2021	A / H5N8	Europe / Finlan	Wild bird	Bianca Zecchin	Istituto Zooprof	Tammiranta,	Finnish Fooc	2021-01-01
EPI_ISL_77787	A/Eurasian_eagle-owl/Finland/10617_21VIR76	A / H5N1	Europe / Finlan	Wild bird	Bianca Zecchin	Istituto Zooprof	Tammiranta,	Finnish Fooc	2021-08-01
EPI_ISL_77787	A/whooper_swan/Finland/9906_21VIR7689-14	A / H5N8	Europe / Finlan	Wild bird	Bianca Zecchin	Istituto Zooprof	Tammiranta,	Finnish Fooc	2021-08-01
EPI_ISL_77787	A/European_herring_gull/Finland/9722_21VIR7	A / H5N1	Europe / Finlan	Wild bird	Bianca Zecchin	Istituto Zooprof	Tammiranta,	Finnish Fooc	2021-08-01
EPI_ISL_77787	A/golden_eagle/Finland/9378_21VIR7689-12/2	A / H5N1	Europe / Finlan	Wild bird	Bianca Zecchin	Istituto Zooprof	Tammiranta,	Finnish Fooc	2021-07-01
EPI_ISL_77787	A/white-tailed_eagle/Finland/9257_21VIR7689	A / H5N8	Europe / Finlan	Wild bird	Bianca Zecchin	Istituto Zooprof	Tammiranta,	Finnish Fooc	2021-07-01
EPI_ISL_77787	A/white-tailed_eagle/Finland/6984_21VIR7689	A / H5N1	Europe / Finlan	Wild bird	Bianca Zecchin	Istituto Zooprof	Tammiranta,	Finnish Fooc	2021-06-01
EPI_ISL_77787	A/mute_swan/Estonia/TA2108545-1_21VIR751	A / H5N8	Europe / Estoni	Wild bird	Bianca Zecchin	Istituto Zooprof	Nurmoja, I.; V	Estonian Vet	2021-03-26
EPI_ISL_77787	A/mute_swan/Estonia/TA2106615_21VIR7512	A / H5N8	Europe / Estoni	Wild bird	Bianca Zecchin	Istituto Zooprof	Nurmoja, I.; V	Estonian Vet	2021-03-04
EPI_ISL_77787	A/mute_swan/Estonia/TA2106419-1_21VIR751	A / H5N8	Europe / Estoni	Wild bird	Bianca Zecchin	Istituto Zooprof	Nurmoja, I.; V	Estonian Vet	2021-03-04
EPI_ISL_77787	A/mute_swan/Estonia/TA2106241_21VIR7512	A / H5N8	Europe / Estoni	Wild bird	Bianca Zecchin	Istituto Zooprof	Nurmoja, I.; V	Estonian Vet	2021-03-02
EPI_ISL_77787	A/mute_swan/Estonia/TA2106111-2_21VIR751	A / H5N8	Europe / Estoni	Wild bird	Bianca Zecchin	Istituto Zooprof	Nurmoja, I.; V	Estonian Vet	2021-03-02
EPI_ISL_77787	A/gull/Estonia/TA2113284-4_21VIR7512-8/202	A / H5N1	Europe / Estoni	Wild bird	Bianca Zecchin	Istituto Zooprof	Nurmoja, I.; V	Estonian Vet	2021-06-02
EPI_ISL_77787	A/white-tailed_eagle/Estonia/TA2111864-2_21	A / H5N1	Europe / Estoni	Wild bird	Bianca Zecchin	Istituto Zooprof	Nurmoja, I.; V	Estonian Vet	2021-05-16
EPI_ISL_77787	A/Withe-tiled_eagle/Estonia/TA2124126-1_21V	A / H5N1	Europe / Estoni	Wild bird	Bianca Zecchin	Istituto Zooprof	Nurmoja, I.; V	Estonian Vet	2021-10-12
EPI_ISL_77787	A/Eagle_owl/Estonia/TA2122239_21VIR10433	A / H5N8	Europe / Estoni	Wild bird	Bianca Zecchin	Istituto Zooprof	Nurmoja, I.; V	Estonian Vet	2021-09-19
EPI_ISL_58047	A/great_skua/Scotland/B07779/2021	A / H5N1	Europe / Unitec	Wild bird	Alex Byrne (Ani	Animal and Pla		Animal and F	2021-07-29
EPI_ISL_31423	A/mute_swan/Austria/21051907_21VIR3291-7/	A / H5N8	Europe / Austria	Wild bird	Bianca Zecchin	Istituto Zooprof	Wodak, E.; R	Istituto Zoopr	2021-04-27

EPI_ISL_31423	A/mute_swan/Austria/21051907_21VIR3291-6.	A / H5N8	Europe / Austria	Wild bird	Bianca Zecchin	Istituto Zooprof	Wodak, E.; R	Istituto Zoopr	2021-04-27
EPI_ISL_31020	A/mute_swan/Romania/11981-2_21VIR3163-6	A / H5N8	Europe / Roma	Wild bird	Bianca Zecchin	Istituto Zooprof	Onita, I.; Neic	Istituto Zoopr	2021-04-08
EPI_ISL_31020	A/mute_swan/Romania/11981-1_21VIR3163-5	A / H5N5	Europe / Roma	Wild bird	Bianca Zecchin	Istituto Zooprof	Onita, I.; Neic	Istituto Zoopr	2021-04-08
EPI_ISL_31020	A/mute_swan/Poland/MB396_21RS1385-19/2(	A / H5N8	Europe / Polan	Wild bird	Bianca Zecchin	Istituto Zooprof	Smietanka, K	Istituto Zoopr	2021-04-22
EPI_ISL_31020	A/white_stork/Poland/MB412_21RS1385-11/2(	A / H5N8	Europe / Polan	Wild bird	Bianca Zecchin	Istituto Zooprof	Smietanka, K	Istituto Zoopr	2021-05-02
EPI_ISL_31020	A/grey_heron/Bulgaria/223_21VIR4270-2/2021	A / H5N5	Europe / Bulgar	Wild bird	Bianca Zecchin	Istituto Zooprof	Goujgoulova,	Istituto Zoopr	2021-03-30
EPI_ISL_31020	A/European_herring_gull/Bulgaria/222_21VIR4	A / H5N5	Europe / Bulgar	Wild bird	Bianca Zecchin	Istituto Zooprof	Goujgoulova,	Istituto Zoopr	2021-03-30
EPI_ISL_12593	A/red knot/France/21P003249/2021	A / H5N3	Europe / Franc	Wild bird	Francois-Xavier	ANSES Agence		Anses (Plouf	2021-01-18
EPI_ISL_95641	A/Eurasian_wigeon/Italy/20VIR7301-362/2020	A / H5N8	Europe / Italy /	Wild bird	Bianca Zecchin	Istituto Zooprof	Zecchin, B.; F	Istituto Zoopr	2020-11-21
EPI_ISL_40581	A/hawk/Poland/003/2020	A / H5N8	Europe / Polan	Wild bird	Edyta Świętoń	National Veterin		National Vet	2020-01-06
EPI_ISL_14767	A/mallard/Alaska/AK20-526/2020	A / H5N2	North America /	Mallard			Reeves,A.B.;		2020-10-22
EPI_ISL_95726	A/mallard/Shanghai/JDS20876/2020	A / H5N8	Asia / China / S	Mallard			Ming,L.		2020-11-19
EPI_ISL_67953	A/mallard/New York/AH0179244/2021	A / H5N4	North America /	Mallard			USDA Nation		2021-08-11
EPI_ISL_58636	A/mallard/Georgia/DT-22495/2020	A / H5N8	Asia / Georgia	Mallard	Nicola Lewis (R	Royal Veterinar	Fouchier, R.A	Erasmus Me	2020-10-09
EPI_ISL_58634	A/mallard/Georgia/DT-22456/2020	A / H5N8	Asia / Georgia	Mallard	Nicola Lewis (R	Royal Veterinar	Fouchier, R.A	Erasmus Me	2020-10-06
EPI_ISL_58633	A/mallard/Georgia/DT-23072/2020	A / H5N8	Asia / Georgia	Mallard	Nicola Lewis (R	Royal Veterinar	Fouchier, R.A	Erasmus Me	2020-10-27
EPI_ISL_58633	A/mallard/Georgia/DT-22360/2020	A / H5N8	Asia / Georgia	Mallard	Nicola Lewis (R	Royal Veterinar	Fouchier, R.A	Erasmus Me	2020-10-02
EPI_ISL_58630	A/mallard/Georgia/DT-22620/2020	A / H5N8	Asia / Georgia	Mallard	Nicola Lewis (R	Royal Veterinar	Fouchier, R.A	Erasmus Me	2020-10-12
EPI_ISL_28153	A/mallard/Ningxia/175/2020	A / H5N8	Asia / China / N	Mallard	Hongliang Chai	Northeast Fores		College of W	2020-10-15
EPI_ISL_95726	A/northern pintail/Shanghai/JDS20843/2020	A / H5N8	Asia / China / S	Northern pi			Ming,L.		2020-11-19
EPI_ISL_12593	A/curlew/France/21P003648/2021	A / H5N3	Europe / Franc	Eurasian cu	Francois-Xavier	ANSES Agence		Anses (Plouf	2021-01-22
EPI_ISL_12250	A/eurasian curlew/Netherlands/21024069-002/	A / H5N4	Europe / Nethe	Eurasian cu	Rene Heutink ('	Wageningen Bi	Beerens, Nar	Wageningen	2021-02-15
EPI_ISL_11580	A/Greylag goose/Sweden/SVA220308SZ0382/	A / H5N1	Europe / Swede	Greylag goo	Siamak Zohari	National Veterin		National Vet	2022-01-21
EPI_ISL_70496	A/bean goose/Sweden/SVA211111SZ0372/FB	A / H5N1	Europe / Swede	Greylag goo	Siamak Zohari	National Veterin		National Vet	2021-11-05
EPI_ISL_65990	A/greylag goose /Sweden/SVA211103SZ0398/	A / H5N1	Europe / Swede	Greylag goo	Siamak Zohari	National Veterin		National Vet	2021-11-01
EPI_ISL_32983	A/Greylag goose/Sweden/SVA210324SZ0403/	A / H5N8	Europe / Swede	Greylag goo	Siamak Zohari	National Veterin		National Vet	2021-03-03
EPI_ISL_10953	A/Greylag goose/Sweden/SVA210209SZ0422/	A / H5N8	Europe / Swede	Greylag goo	Siamak Zohari	National Veterin		National Vet	2021-02-03
EPI_ISL_95641	A/greylag_goose/Italy/20VIR7660-6/2020	A / H5N8	Europe / Italy /	Greylag goo	Bianca Zecchin	Istituto Zooprof	Zecchin, B.; F	Istituto Zoopr	2020-11-29
EPI_ISL_71050	A/Greylag_goose/England/033100/2020	A / H5N8	Europe / United	Greylag goo	Alex Byrne (Ani	Animal and Pla		Animal and F	2020-10-30
EPI_ISL_71050	A/Greylag_goose/England/032698/2020	A / H5N8	Europe / United	Greylag goo	Alex Byrne (Ani	Animal and Pla		Animal and F	2020-11-03
EPI_ISL_77787	A/guinea_fowl/Estonia/TA2104719_21VIR7512	A / H5N8	Europe / Estoni	Guinea fow	Bianca Zecchin	Istituto Zooprof	Nurmoja, I.; V	Estonian Vet	2021-02-18
EPI_ISL_75978	A/Podiceps_cristatus/Belgium/12659_0015/202	A / H5N8	Europe / Belgiu	Great crest	Steven Van Bor	Sciensano, Dep	Van Borm, St	Sciensano -	2020-11-24
EPI_ISL_10056	A/common teal/Chany Lake/213/2020	A / H5N2	Europe / Russia	Teal	Takehiko Saito	National Institut	Mine, J.; Uchi	Research Ins	2020-08-29
EPI_ISL_83324	A/Muscovy duck/China/FJFZ21/H5N6/2020	A / H5N6	Asia / China / F	Teal	Rui Zhang (Fuji	Fujian Agricultu		Institute of ar	2020-08-12
EPI_ISL_40610	A/Red knot/Delaware Bay/404/2020	A / H5N3	North America /	Calidris car			Direct Submis		2020-05-29
EPI_ISL_95728	A/Muscovy duck/Vietnam/HN6111/2020	A / H5N6	Asia / Vietnam	Cairina mos			Kawaoka,Y.;		2020-02-10
EPI_ISL_95728	A/Muscovy duck/Vietnam/HN6114/2020	A / H5N6	Asia / Vietnam	Cairina mos			Kawaoka,Y.;		2020-02-10
EPI_ISL_95728	A/Muscovy duck/Vietnam/HN6119/2020	A / H5N6	Asia / Vietnam	Cairina mos			Kawaoka,Y.;		2020-02-10
EPI_ISL_95727	A/Muscovy duck/Vietnam/HN6115/2020	A / H5N6	Asia / Vietnam	Cairina mos			Kawaoka,Y.;		2020-02-10
EPI_ISL_95727	A/Muscovy duck/Vietnam/HN6120/2020	A / H5N6	Asia / Vietnam	Cairina mos			Kawaoka,Y.;		2020-02-10
EPI_ISL_95727	A/Muscovy duck/Vietnam/HN6113/2020	A / H5N6	Asia / Vietnam	Cairina mos			Kawaoka,Y.;		2020-02-10
EPI_ISL_95727	A/Muscovy duck/Vietnam/HN6608/2020	A / H5N6	Asia / Vietnam	Cairina mos			Kawaoka,Y.;		2020-09-29

EPI_ISL_95727	A/Muscovy duck/Vietnam/HN6607/2020	A / H5N6	Asia / Vietnam	Cairina mos			Kawaoka,Y.;			2020-09-29
EPI_ISL_95727	A/Muscovy duck/Vietnam/HN6606/2020	A / H5N6	Asia / Vietnam	Cairina mos			Kawaoka,Y.;			2020-09-29
EPI_ISL_95727	A/Muscovy duck/Vietnam/HN6609/2020	A / H5N6	Asia / Vietnam	Cairina mos			Kawaoka,Y.;			2020-09-29
EPI_ISL_95727	A/Muscovy duck/Vietnam/HN6610/2020	A / H5N6	Asia / Vietnam	Cairina mos			Kawaoka,Y.;			2020-09-29
EPI_ISL_77524	A/muscovy duck/Netherlands/20019914-001/2(	A / H5N8	Europe / Nethe	Cairina mos	Rene Heutink ('	Wageningen Bi	Beerens, Nar		Wageningen	2020-12-11
EPI_ISL_76233	A/Garrulus_glandarius/Belgium/2928_003/202	A / H5N8	Europe / Belgiu	Passerine	Steven Van Bo	Sciensano, Dep	Van Borm, St		Sciensano -	2021-03-01
EPI_ISL_13300	A/brant goose/Netherlands/22009881-002/202	A / H5N1	Europe / Nethe	Branta berr	Rene Heutink ('	Wageningen Bi	Beerens, Nar		Wageningen	2022-05-26
EPI_ISL_66009	A/great black-backed gull/Sweden/SVA211109	A / H5N1	Europe / Swede	Chroicocep	Siamak Zohari	National Veterii			National Vete	2021-11-09
EPI_ISL_92215	A/barnacle goose/Netherlands/22000419-002/2	A / H5N1	Europe / Nethe	Branta leuc	Rene Heutink ('	Wageningen Bi	Beerens, Nar		Wageningen	2022-01-06
EPI_ISL_60256	A/Barnacle goose/Netherlands/21037293-001/2	A / H5N1	Europe / Nethe	Branta leuc	Rene Heutink ('	Wageningen Bi	Beerens, Nar		Wageningen	2021-10-25
EPI_ISL_50994	A/barnacle goose/Germany-NI/AI01605/2021	A / H5N1	Europe / Germa	Branta leuc	Jacqueline King	Friedrich-Loeffl			Lebensmittel	2021-02-17
EPI_ISL_50600	A/barnacle goose/Germany-SH/AI02199/2020	A / H5N8	Europe / Germa	Branta leuc	Jacqueline King	Friedrich-Loeffl			Landeslabor	2020-11-03
EPI_ISL_50584	A/barnacle goose/Germany-SH/AI02190/2020	A / H5N8	Europe / Germa	Branta leuc	Jacqueline King	Friedrich-Loeffl			Landeslabor	2020-11-02
EPI_ISL_31022	A/barnacle goose/Sweden/SVA210210SZ0372	A / H5N8	Europe / Swede	Branta leuc	Siamak Zohari	National Veterii			National Vete	2021-02-01
EPI_ISL_31020	A/barnacle goose/Sweden/SVA210423SZ0252	A / H5N8	Europe / Swede	Branta leuc	Siamak Zohari	National Veterii			National Vete	2021-01-06
EPI_ISL_31020	A/barnacle goose/Sweden/SVA210423SZ0250	A / H5N8	Europe / Swede	Branta leuc	Siamak Zohari	National Veterii			National Vete	2021-01-19
EPI_ISL_21768	A/barnacle goose/Netherlands/21023501-002/2	A / H5N8	Europe / Nethe	Branta leuc	Rene Heutink ('	Wageningen Bi	Beerens, Nar		Wageningen	2021-02-05
EPI_ISL_21763	A/barnacle goose/Netherlands/21023498-002/2	A / H5N8	Europe / Nethe	Branta leuc	Rene Heutink ('	Wageningen Bi	Beerens, Nar		Wageningen	2021-02-14
EPI_ISL_12249	A/barnacle goose/Netherlands/21022611-001/2	A / H5N1	Europe / Nethe	Branta leuc	Rene Heutink ('	Wageningen Bi	Beerens, Nar		Wageningen	2021-01-31
EPI_ISL_10954	A/barnacle goose/Sweden/SVA210126SZ0462	A / H5N8	Europe / Swede	Branta leuc	Siamak Zohari	National Veterii			National Vete	2021-01-22
EPI_ISL_10954	A/barnacle goose/Sweden/SVA210202SZ0453	A / H5N8	Europe / Swede	Branta leuc	Siamak Zohari	National Veterii			National Vete	2021-02-01
EPI_ISL_71822	A/barnacle goose/Sweden/SVA201125SZ0472	A / H5N8	Europe / Swede	Branta leuc	Siamak Zohari	National Veterii			National Vete	2020-11-15
EPI_ISL_66845	A/barnacle goose/Sweden/SVA201117SZ0468	A / H5N8	Europe / Swede	Branta leuc	Siamak Zohari	National Veterii			National Vete	2020-11-12
EPI_ISL_64482	A/barnacle goose/Denmark/14138-1/2020-11-(	A / H5N8	Europe / Denm	Branta leuc	Charlotte Kristia	Statens Serum	Charlotte,Hjul	Liang	Statens Seru	2020-11-04
EPI_ISL_13514	A/Gallus gallus/Belgium/8092_0002/2022	A / H5N1	Europe / Belgiu	Gallus gallu	Steven Van Bo	Sciensano, Dep	Van Borm, St		Sciensano -	2022-06-20
EPI_ISL_12980	A/Chicken/Sweden/SVA210201SZ0080/FB000	A / H5N5	Europe / Swede	Gallus gallu	Siamak Zohari	National Veterii			National Vete	2022-01-21
EPI_ISL_11449	A/Gallus_gallus/Belgium/3194_0001/2022	A / H5N1	Europe / Belgiu	Gallus gallu	Steven Van Bo	Sciensano, Dep	Van Borm, St		Sciensano -	2022-03-10
EPI_ISL_76241	A/Gallus_gallus/Belgium/11644/2021	A / H5N8	Europe / Belgiu	Gallus gallu	Steven Van Bo	Sciensano, Dep	Van Borm, St		Sciensano -	2021-09-09
EPI_ISL_76237	A/Gallus_gallus/Belgium/9247/2021	A / H5N8	Europe / Belgiu	Gallus gallu	Steven Van Bo	Sciensano, Dep	Van Borm, St		Sciensano -	2021-07-12
EPI_ISL_76235	A/Gallus_gallus/Belgium/7578_001/2021	A / H5N8	Europe / Belgiu	Gallus gallu	Steven Van Bo	Sciensano, Dep	Van Borm, St		Sciensano -	2021-06-04
EPI_ISL_83220	A/Chicken/Sweden/SVA210111SZ0065/KN005	A / H5N8	Europe / Swede	Gallus gallu	Siamak Zohari	National Veterii			National Vete	2021-01-10
EPI_ISL_71051	A/chicken/England/037052/2020	A / H5N8	Europe / Unitec	Gallus gallu	Alex Byrne (Ani	Animal and Pla			Animal and P	2020-11-21
EPI_ISL_71050	A/chicken/England/033708/2020	A / H5N8	Europe / Unitec	Gallus gallu	Alex Byrne (Ani	Animal and Pla			Animal and P	2020-11-10
EPI_ISL_66026	A/Gallus_gallus/Belgium/12168_002/2020	A / H5N5	Europe / Belgiu	Gallus gallu	Steven Van Bo	Sciensano, Dep	Van Borm,Ste		Sciensano -	2020-11-18
EPI_ISL_64416	A/chicken/Chelyabinsk/404/2020	A / H5N8	Europe / Russia	Gallus gallu	Natalia Goncha	State Research	Natalia,Goncl		State Resea	2020-08-06
EPI_ISL_64416	A/chicken/Chelyabinsk/403/2020	A / H5N8	Europe / Russia	Gallus gallu	Natalia Goncha	State Research	Natalia,Goncl		State Resea	2020-08-06
EPI_ISL_64415	A/chicken/Omsk/0073/2020	A / H5N8	Europe / Russia	Gallus gallu	Natalia Goncha	State Research	Natalia,Goncl		State Resea	2020-08-17
EPI_ISL_64415	A/chicken/Omsk/0119/2020	A / H5N8	Europe / Russia	Gallus gallu	Natalia Goncha	State Research	Natalia,Goncl		State Resea	2020-08-17
EPI_ISL_64415	A/chicken/Omsk/0118/2020	A / H5N8	Europe / Russia	Gallus gallu	Natalia Goncha	State Research	Natalia,Goncl		State Resea	2020-08-17
EPI_ISL_64415	A/chicken/Omsk/0112/2020	A / H5N8	Europe / Russia	Gallus gallu	Natalia Goncha	State Research	Natalia,Goncl		State Resea	2020-08-17
EPI_ISL_64414	A/chicken/Chelyabinsk/404/2020	A / H5N8	Europe / Russia	Gallus gallu	Natalia Goncha	State Research	Natalia,Goncl		State Resea	2020-08-06
EPI_ISL_64414	A/chicken/Chelyabinsk/403/2020	A / H5N8	Europe / Russia	Gallus gallu	Natalia Goncha	State Research	Natalia,Goncl		State Resea	2020-08-06

EPI_ISL_6441	A/chicken/Chelyabinsk/402/2020	A / H5N8	Europe / Russia	Gallus gallu	Natalia Goncha	State Research	Natalia,Goncl	State Resea	2020-08-06
EPI_ISL_6441	A/chicken/Chelyabinsk/401/2020	A / H5N8	Europe / Russia	Gallus gallu	Natalia Goncha	State Research	Natalia,Goncl	State Resea	2020-08-06
EPI_ISL_6441	A/chicken/Omsk/0073/2020	A / H5N8	Europe / Russia	Gallus gallu	Natalia Goncha	State Research	Natalia,Goncl	State Resea	2020-08-17
EPI_ISL_6441	A/chicken/Omsk/0119/2020	A / H5N8	Europe / Russia	Gallus gallu	Natalia Goncha	State Research	Natalia,Goncl	State Resea	2020-08-17
EPI_ISL_6441	A/chicken/Omsk/0118/2020	A / H5N8	Europe / Russia	Gallus gallu	Natalia Goncha	State Research	Natalia,Goncl	State Resea	2020-08-17
EPI_ISL_6441	A/chicken/Omsk/0112/2020	A / H5N8	Europe / Russia	Gallus gallu	Natalia Goncha	State Research	Natalia,Goncl	State Resea	2020-08-17
EPI_ISL_6031	A/chicken/Netherlands/20016597-026030/202	A / H5N8	Europe / Nethe	Gallus gallu	Rene Heutink (	Wageningen Bi	Beerens, Nar	Wageningen	2020-10-28
EPI_ISL_4151	A/chicken/Germany-SN/AI00276/2020	A / H5N8	Europe / Germa	Gallus gallu	Jacqueline Kin	Friedrich-Loeffl		Landesunter	2020-03-12
EPI_ISL_4053	A/chicken/Czech Republic/1175-1/2020	A / H5N8	Europe / Czech	Gallus gallu	Alexander Nag	State Veterinar	Nagy,A	State Veterir	2020-01-17
EPI_ISL_1257	A/pigeon/Jiangxi/S40784/2021(H5N1)	A / H5N1	Asia / China / J	Pigeon	Pengfei Cui (H	Harbin Veterina	Pengfei Cui,	Harbin Veter	2021-12-08
EPI_ISL_1026	A/pigeon/Germany-NW/AI00951/2022	A / H5N1	Europe / Germa	Pigeon	Jacqueline Kin	Friedrich-Loeffl		Chemisches	2022-01-24
EPI_ISL_7623	A/Columba_palumbus/Belgium/2928_004/202	A / H5N8	Europe / Belgiu	Pigeon	Steven Van Bo	Sciensano, De	Van Borm, St	Sciensano -	2021-03-01
EPI_ISL_5863	A/green-winged-teal/Georgia/DT-23070/2020	A / H5N8	Asia / Georgia	Green-wing	Nicola Lewis (R	Royal Veterinar	Fouchier, R.A	Erasmus Me	2020-10-27
EPI_ISL_5863	A/green-winged-teal/Georgia/DT-22792/2020	A / H5N8	Asia / Georgia	Green-wing	Nicola Lewis (R	Royal Veterinar	Fouchier, R.A	Erasmus Me	2020-10-16
EPI_ISL_5863	A/green-winged-teal/Georgia/DT-22332/2020	A / H5N8	Asia / Georgia	Green-wing	Nicola Lewis (R	Royal Veterinar	Fouchier, R.A	Erasmus Me	2020-10-02
EPI_ISL_5862	A/green-winged-teal/Georgia/DT-22810/2020	A / H5N8	Asia / Georgia	Green-wing	Nicola Lewis (R	Royal Veterinar	Fouchier,R.A.	Erasmus Me	2020-10-16
EPI_ISL_4061	A/ruddy turnstone/Delaware Bay/386/2020	A / H5N9	North America	Arenaria int			Direct Submis		2020-05-29
EPI_ISL_1484	A/Whooper swan/Sanmenxia/B2465/2021	A / H5N8	Asia / China / H	Whooper s	Zeyu Yang (Ch	Chinese Acade		Xi'an Tianlon	2021-01-21
EPI_ISL_1484	A/Whooper swan/Sanmenxia/B1770/2020	A / H5N8	Asia / China / H	Whooper s	Zeyu Yang (Ch	Chinese Acade		Xi'an Tianlon	2021-01-15
EPI_ISL_1483	A/Whooper swan/Sanmenxia/B1109/2021	A / H5N8	Asia / China / H	Whooper s	Zeyu Yang (Ch	Chinese Acade		Xi'an Tianlon	2021-01-15
EPI_ISL_1482	A/Whooper swan/Sanmenxia/Y52/2020	A / H5N8	Asia / China / H	Whooper s	Zeyu Yang (Ch	Chinese Acade		Xi'an Tianlon	2020-11-10
EPI_ISL_1482	A/Whooper swan/Sanmenxia/Y16/2020	A / H5N8	Asia / China / H	Whooper s	Zeyu Yang (Ch	Chinese Acade		Xi'an Tianlon	2020-11-10
EPI_ISL_1482	A/Whooper swan/Sanmenxia/Y15/2020	A / H5N8	Asia / China / H	Whooper s	Zeyu Yang (Ch	Chinese Acade		Xi'an Tianlon	2020-11-10
EPI_ISL_1482	A/Whooper swan/Sanmenxia/G15/2020	A / H5N8	Asia / China / H	Whooper s	Zeyu Yang (Ch	Chinese Acade		Xi'an Tianlon	2020-11-10
EPI_ISL_1482	A/Whooper swan/Sanmenxia/Y14/2020	A / H5N8	Asia / China / H	Whooper s	Zeyu Yang (Ch	Chinese Acade		Xi'an Tianlon	2020-11-09
EPI_ISL_1482	A/Whooper swan/Sanmenxia/G13/2020	A / H5N8	Asia / China / H	Whooper s	Zeyu Yang (Ch	Chinese Acade		Xi'an Tianlon	2020-11-09
EPI_ISL_1482	A/Whooper swan/Sanmenxia/Y12/2020	A / H5N8	Asia / China / H	Whooper s	Zeyu Yang (Ch	Chinese Acade		Xi'an Tianlon	2020-11-08
EPI_ISL_1482	A/Whooper swan/Sanmenxia/Y11/2020	A / H5N8	Asia / China / H	Whooper s	Zeyu Yang (Ch	Chinese Acade		Xi'an Tianlon	2020-11-08
EPI_ISL_1482	A/Whooper swan/Sanmenxia/Y10/2020	A / H5N8	Asia / China / H	Whooper s	Zeyu Yang (Ch	Chinese Acade		Xi'an Tianlon	2020-11-08
EPI_ISL_1482	A/Whooper swan/Sanmenxia/Y8/2020	A / H5N8	Asia / China / H	Whooper s	Zeyu Yang (Ch	Chinese Acade		Xi'an Tianlon	2020-11-05
EPI_ISL_1482	A/Whooper swan/Sanmenxia/Y6/2020	A / H5N8	Asia / China / H	Whooper s	Zeyu Yang (Ch	Chinese Acade		Xi'an Tianlon	2020-11-05
EPI_ISL_1482	A/Whooper swan/Sanmenxia/G2/2020	A / H5N8	Asia / China / H	Whooper s	Zeyu Yang (Ch	Chinese Acade		Xi'an Tianlon	2020-11-04
EPI_ISL_1482	A/Whooper swan/Sanmenxia/Y1/2020	A / H5N8	Asia / China / H	Whooper s	Zeyu Yang (Ch	Chinese Acade		Xi'an Tianlon	2020-10-30
EPI_ISL_3315	A/whooper swan/Sweden/SVA210318SZ0304/	A / H5N8	Europe / Swed	Whooper s	Siamak Zohari	National Veteri		National Vet	2021-03-10
EPI_ISL_1939	A/whooper swan/Henan/SMQ10/2020	A / H5N8	Asia / China / H	Whooper s	Hongliang Chai	Northeast Fore		College of W	2020-11-10
EPI_ISL_1938	A/whooper swan/Henan/SMQ9/2020	A / H5N8	Asia / China / H	Whooper s	Hongliang Chai	Northeast Fore		College of W	2020-11-10
EPI_ISL_1938	A/whooper swan/Henan/SM111/2020	A / H5N8	Asia / China / H	Whooper s	Hongliang Chai	Northeast Fore		College of W	2020-11-10
EPI_ISL_1938	A/whooper swan/Henan/SM86/2020	A / H5N8	Asia / China / H	Whooper s	Hongliang Chai	Northeast Fore		College of W	2020-11-10
EPI_ISL_1937	A/whooper swan/Henan/SM76/2020	A / H5N8	Asia / China / H	Whooper s	Hongliang Chai	Northeast Fore		College of W	2020-11-10
EPI_ISL_1937	A/whooper swan/Henan/SM61/2020	A / H5N8	Asia / China / H	Whooper s	Hongliang Chai	Northeast Fore		College of W	2020-11-10
EPI_ISL_1937	A/whooper swan/Henan/SM31/2020	A / H5N8	Asia / China / H	Whooper s	Hongliang Chai	Northeast Fore		College of W	2020-11-10
EPI_ISL_1937	A/whooper swan/Henan/SM16/2020	A / H5N8	Asia / China / H	Whooper s	Hongliang Chai	Northeast Fore		College of W	2020-11-10

EPI_ISL_19378	A/whooper swan/Henan/SM1/2020	A / H5N8	Asia / China / H	Whooper sw	Hongliang Chai	Northeast Fores		College of W	2020-11-10
EPI_ISL_19378	A/whooper swan/Henan/SMQ7/2020	A / H5N8	Asia / China / H	Whooper sw	Hongliang Chai	Northeast Fores		College of W	2020-11-09
EPI_ISL_18225	A/whooper swan/Shanxi/SX346/2020	A / H5N8	Asia / China / S	Whooper sw	Hongliang Chai	Northeast Fores		College of W	2020-11-10
EPI_ISL_18225	A/whooper swan/Shanxi/SX291/2020	A / H5N8	Asia / China / S	Whooper sw	Hongliang Chai	Northeast Fores		College of W	2020-11-10
EPI_ISL_18225	A/whooper swan/Shanxi/SX276/2020	A / H5N8	Asia / China / S	Whooper sw	Hongliang Chai	Northeast Fores		College of W	2020-11-10
EPI_ISL_18225	A/whooper swan/Shanxi/SX251/2020	A / H5N8	Asia / China / S	Whooper sw	Hongliang Chai	Northeast Fores		College of W	2020-11-10
EPI_ISL_18225	A/whooper swan/Shanxi/SX231/2020	A / H5N8	Asia / China / S	Whooper sw	Hongliang Chai	Northeast Fores		College of W	2020-11-10
EPI_ISL_18225	A/whooper swan/Shanxi/SX216/2020	A / H5N8	Asia / China / S	Whooper sw	Hongliang Chai	Northeast Fores		College of W	2020-11-10
EPI_ISL_18225	A/whooper swan/Shanxi/SX206/2020	A / H5N8	Asia / China / S	Whooper sw	Hongliang Chai	Northeast Fores		College of W	2020-11-10
EPI_ISL_18225	A/whooper swan/Shanxi/SX166/2020	A / H5N8	Asia / China / S	Whooper sw	Hongliang Chai	Northeast Fores		College of W	2020-11-10
EPI_ISL_77526	A/black-headed gull/Netherlands/20020162-00	A / H5N8	Europe / Nethe	Black-head	Rene Heutink ('	Wageningen Bi	Beerens, Nar	Wageningen	2020-12-17
EPI_ISL_14389	A/Anser_anser_domesticus/Belgium/1668_001	A / H5N1	Europe / Belgiu	Anser anse	Steven Van Bo	Sciensano, Dep	Van Borm, St	Sciensano - .	2022-01-26
EPI_ISL_64415	A/goose/Omsk/011101/2020	A / H5N8	Europe / Russia	Anser anse	Natalia Goncha	State Research	Natalia,Goncl	State Resea	2020-08-17
EPI_ISL_64415	A/goose/Omsk/01161/2020	A / H5N8	Europe / Russia	Anser anse	Natalia Goncha	State Research	Natalia,Goncl	State Resea	2020-08-17
EPI_ISL_64415	A/goose/Omsk/0114/2020	A / H5N8	Europe / Russia	Anser anse	Natalia Goncha	State Research	Natalia,Goncl	State Resea	2020-08-17
EPI_ISL_64414	A/goose/Omsk/0074/2020	A / H5N8	Europe / Russia	Anser anse	Natalia Goncha	State Research	Natalia,Goncl	State Resea	2020-08-17
EPI_ISL_64413	A/goose/Omsk/0071/2020	A / H5N8	Europe / Russia	Anser anse	Natalia Goncha	State Research	Natalia,Goncl	State Resea	2020-08-17
EPI_ISL_64413	A/goose/Omsk/011101/2020	A / H5N8	Europe / Russia	Anser anse	Natalia Goncha	State Research	Natalia,Goncl	State Resea	2020-08-17
EPI_ISL_64413	A/goose/Omsk/01171/2020	A / H5N8	Europe / Russia	Anser anse	Natalia Goncha	State Research	Natalia,Goncl	State Resea	2020-08-17
EPI_ISL_64413	A/goose/Omsk/01161/2020	A / H5N8	Europe / Russia	Anser anse	Natalia Goncha	State Research	Natalia,Goncl	State Resea	2020-08-17
EPI_ISL_64412	A/goose/Omsk/0115/2020	A / H5N8	Europe / Russia	Anser anse	Natalia Goncha	State Research	Natalia,Goncl	State Resea	2020-08-17
EPI_ISL_64412	A/goose/Omsk/0114/2020	A / H5N8	Europe / Russia	Anser anse	Natalia Goncha	State Research	Natalia,Goncl	State Resea	2020-08-17
EPI_ISL_64412	A/goose/Omsk/0113/2020	A / H5N8	Europe / Russia	Anser anse	Natalia Goncha	State Research	Natalia,Goncl	State Resea	2020-08-17
EPI_ISL_64412	A/goose/Omsk/0111/2020	A / H5N8	Europe / Russia	Anser anse	Natalia Goncha	State Research	Natalia,Goncl	State Resea	2020-08-17
EPI_ISL_64412	A/goose/Omsk/0002/2020	A / H5N8	Europe / Russia	Anser anse	Natalia Goncha	State Research	Natalia,Goncl	State Resea	2020-08-13
EPI_ISL_69595	A/quail/Korea/H526/2021	A / H5N1	Asia / Korea, R	Coturnix sp	Yu-Na Lee (Ani	Animal and Pla		Animal and F	2021-11-08
EPI_ISL_66131	A/Anser_albifrons/Belgium/11956_005/2020	A / H5N8	Europe / Belgiu	White-front	Steven Van Bo	Sciensano, Dep	Van Borm,Ste	Sciensano - .	2020-11-07
EPI_ISL_64415	A/duck/Omsk/0076/2020	A / H5N8	Europe / Russia	Anas platyr	Natalia Goncha	State Research	Natalia,Goncl	State Resea	2020-08-17
EPI_ISL_64414	A/duck/Omsk/0004/2020	A / H5N8	Europe / Russia	Anas platyr	Natalia Goncha	State Research	Natalia,Goncl	State Resea	2020-08-13
EPI_ISL_64414	A/duck/Omsk/0077/2020	A / H5N8	Europe / Russia	Anas platyr	Natalia Goncha	State Research	Natalia,Goncl	State Resea	2020-08-17
EPI_ISL_64414	A/duck/Omsk/0076/2020	A / H5N8	Europe / Russia	Anas platyr	Natalia Goncha	State Research	Natalia,Goncl	State Resea	2020-08-17
EPI_ISL_64414	A/duck/Omsk/0075/2020	A / H5N8	Europe / Russia	Anas platyr	Natalia Goncha	State Research	Natalia,Goncl	State Resea	2020-08-17
EPI_ISL_64412	A/duck/Omsk/0004/2020	A / H5N8	Europe / Russia	Anas platyr	Natalia Goncha	State Research	Natalia,Goncl	State Resea	2020-08-13
EPI_ISL_83348	A/Mallard_duck/Hungary/18410/2020	A / H5N8	Europe / Hunga	Mulard duc	Katalin Szentpá	National Food (	Katalin,Szent	National Foo	2020-04-14
EPI_ISL_28153	A/common teal/Ningxia/105/2020	A / H5N8	Asia / China / N	Common te	Hongliang Chai	Northeast Fores		College of W	2020-10-11
EPI_ISL_95640	A/common_teal/Italy/20VIR7439-190/2020	A / H5N5	Europe / Italy /	Common te	Bianca Zecchin	Istituto Zooprof	Zecchin, B.; F	Istituto Zoopi	2020-11-28
EPI_ISL_95640	A/common_teal/Italy/20VIR7608-73/2020	A / H5N8	Europe / Italy /	Common te	Bianca Zecchin	Istituto Zooprof	Zecchin, B.; F	Istituto Zoopi	2020-12-04
EPI_ISL_98767	A/American blue-winged teal/South Carolina/Al	A / H5N1	North America /	Anseriform	Mary Lea Killiar	National Veterii	Chinh,Thanh;	USDA-NWR	2021-12-30
EPI_ISL_99093	A/turkey/Indiana/22-003707-003/2022	A / H5N1	North America /	Meleagris g	Mary Lea Killiar	National Veterii	Chinh,Thanh;	Indiana State	2022-02-07
EPI_ISL_69355	A/turkey/Poland/H1944-N/2021	A / H5N1	Europe / Poland	Meleagris g	Edyta Świętoń (	National Veterii	E. Swieton, K	National Vet(	2021-11-08
EPI_ISL_43967	A/Meleagris gallopavo/Belgium/11293_001/202	A / H5N8	Europe / Belgiu	Meleagris g	Steven Van Bo	Sciensano, Dep		Sciensano - .	2021-08-30
EPI_ISL_10954	A/Turkey/Sweden/SVA201118SZ0002/KN3056	A / H5N8	Europe / Swede	Meleagris g	Siamak Zohari	National Veterii		National Vet(	2020-11-17

EPI_ISL_83220	A/Turkey/Sweden/SVA210114SZ0001/KN0095	A / H5N8	Europe / Swede	Meleagris g	Siamak Zohari	National Veterii		National Vete	2021-01-13
EPI_ISL_77526	A/turkey/Netherlands/21020942-001005/2021	A / H5N8	Europe / Nethe	Meleagris g	Rene Heutink ('	Wageningen Bi	Beerens, Nar	Wageningen	2021-01-04
EPI_ISL_64412	A/turkey/Omsk/0003/2020	A / H5N8	Europe / Russia	Meleagris g	Natalia Goncha	State Research	Natalia,Goncl	State Resear	2020-08-13
EPI_ISL_64412	A/turkey/Omsk/0001/2020	A / H5N8	Europe / Russia	Meleagris g	Natalia Goncha	State Research	Natalia,Goncl	State Resear	2020-08-13
EPI_ISL_75910	A/Anas_platyrhynchos domestica/Belgium/551	A / H5N8	Europe / Belgiu	Anas platyr	Steven Van Bo	Sciensano, Dep	Van Borm, St	Sciensano -	2021-04-20
EPI_ISL_69595	A/mandarin duck/Korea/WA585/2021	A / H5N1	Asia / Korea, R	Wild waterf	Yu-Na Lee (Ani	Animal and Pla		Animal and F	2021-10-26
EPI_ISL_68294	A/hooded crane/Kagoshima/KU-5T/2021 (H5N	A / H5N8	Asia / Japan / K	Wild waterf	Makoto Ozawa	Kagoshima Uni		Kagoshima U	2021-11-19
EPI_ISL_10639	A/common kestrel/Denmark/16023-01/2021-01	A / H5N3	Europe / Denm	Falco tinnu	Charlotte Kristia	Statens Serum	Charlotte Hjul	Statens Seru	2021-01-01
EPI_ISL_10482	A/common kestrel/Netherlands/20020264-002/	A / H5N8	Europe / Nethe	Falco tinnu	Rene Heutink ('	Wageningen Bi	Beerens, Nar	Wageningen	2020-12-17
EPI_ISL_14761	A/goose/Italy/22VIR1520/2022	A / H5N1	Europe / Italy	Goose	Giacomo Barbi	Istituto Zooprof	Barbierato, G	Istituto Zoop	2022-02-14
EPI_ISL_14760	A/goose/Italy/Italy/21VIR10458/2021	A / H5N1	Europe / Italy	Goose	Giacomo Barbi	Istituto Zooprof	Barbierato, G	Istituto Zoop	2021-11-30
EPI_ISL_14760	A/goose/Italy/IZSLT_21VIR10273/2021	A / H5N1	Europe / Italy	Goose	Giacomo Barbi	Istituto Zooprof	Barbierato, G	Istituto Zoop	2021-11-22
EPI_ISL_73805	A/Goose/Shandong/21153-3/2021(H5N8)	A / H5N8	Asia / China / S	Goose	Jiahao Zhang (	South China Ag	Zhang, Jiaha	South China	2021-02-01
EPI_ISL_73802	A/Goose/Jiangsu/21153-2/2021(H5N8)	A / H5N8	Asia / China / J	Goose	Jiahao Zhang (	South China Ag	Zhang, Jiaha	South China	2021-02
EPI_ISL_73794	A/Goose/Henan/21056-2/2021(H5N8)	A / H5N8	Asia / China / H	Goose	Jiahao Zhang (	South China Ag	Zhang, Jiaha	South China	2021-01
EPI_ISL_73794	A/Goose/Henan/21028/2021(H5N8)	A / H5N8	Asia / China / H	Goose	Jiahao Zhang (	South China Ag	Zhang, Jiaha	South China	2021-01
EPI_ISL_67728	A/Goose/Guangdong/211106-2/2021(H5N6)	A / H5N6	Asia / China / G	Goose	Jiahao Zhang (	South China Ag	Zhang, Jiaha	South China	2021-08
EPI_ISL_67580	A/Goose/Sichuan/21418-2/2021(H5N6)	A / H5N6	Asia / China / S	Goose	Jiahao Zhang (	South China Ag	Zhang, Jiaha	South China	2021-04
EPI_ISL_67576	A/Goose/Sichuan/21406-5/2021(H5N6)	A / H5N6	Asia / China / S	Goose	Jiahao Zhang (	South China Ag	Zhang, Jiaha	South China	2021-04
EPI_ISL_66757	A/goose/Netherlands/21038413-002/2021	A / H5N1	Europe / Nethe	Goose	Rene Heutink ('	Wageningen Bi	Beerens, Nar	Wageningen	2021-11-08
EPI_ISL_61018	A/goose/Netherlands/21037720-001/2021	A / H5N1	Europe / Nethe	Goose	Rene Heutink ('	Wageningen Bi	Beerens, Nar	Wageningen	2021-10-28
EPI_ISL_51445	A/domestic goose/Germany-NI/AI03142/2021	A / H5N8	Europe / Germa	Goose	Jacqueline King	Friedrich-Loeffl		Lebensmittel	2021-03-20
EPI_ISL_51433	A/domestic goose/Germany-MV/AI02558/2021	A / H5N8	Europe / Germa	Goose	Jacqueline King	Friedrich-Loeffl		Landesamt f	2021-03-10
EPI_ISL_50953	A/wild goose/Germany-NI/AI03473/2020	A / H5N8	Europe / Germa	Goose	Jacqueline King	Friedrich-Loeffl		Lebensmittel	2020-12-17
EPI_ISL_50953	A/wild goose/Germany-NI/AI03220/2020	A / H5N8	Europe / Germa	Goose	Jacqueline King	Friedrich-Loeffl		Lebensmittel	2020-12-10
EPI_ISL_50618	A/domestic goose/Germany-SH/AI02884/2020	A / H5N8	Europe / Germa	Goose	Jacqueline King	Friedrich-Loeffl		Landeslabor	2020-11-18
EPI_ISL_29326	A/goose/Kazakhstan/7-20-B-Talg-12/2020	A / H5N8	Asia / Kazakhst	Goose	Alexandr Shevt	National Center	Asylulan,Amii	National Vete	2020-09-22
EPI_ISL_29326	A/goose/Kazakhstan/4-190-20-B-H5N8-1/2020	A / H5N8	Asia / Kazakhst	Goose	Alexandr Shevt	National Center	Asylulan,Amii	National Vete	2020-09-01
EPI_ISL_22348	A/goose/Romania/10330_21VIR2593-35/2021	A / H5N8	Europe / Roma	Goose	Bianca Zecchin	Istituto Zooprof	Onita, I.; Neic	Istituto Zoop	2021-01-19
EPI_ISL_22348	A/goose/Romania/10205-t5_21VIR2593-8/202	A / H5N8	Europe / Roma	Goose	Bianca Zecchin	Istituto Zooprof	Onita, I.; Neic	Istituto Zoop	2021-01-13
EPI_ISL_22348	A/goose/Romania/10205-t3_21VIR2593-7/202	A / H5N8	Europe / Roma	Goose	Bianca Zecchin	Istituto Zooprof	Onita, I.; Neic	Istituto Zoop	2021-01-13
EPI_ISL_22272	A/goose/Netherlands/21028502-002/21028502	A / H5N1	Europe / Nethe	Goose	Rene Heutink ('	Wageningen Bi	Beerens, Nar	Wageningen	2021-05-09
EPI_ISL_12200	A/tundra_bean_goose/Poland/MB132/2020	A / H5N8	Europe / Polan	Goose	Edyta Świętoń	National Veterii	Swieton E., S	National Vete	2020-12-09
EPI_ISL_80965	A/Goose/Hungary/18406/2020	A / H5N8	Europe / Hunga	Goose	Katalin Szentpá	National Food (	Katalin,Szent	National Foo	2020-04-14
EPI_ISL_12474	A/greylag goose /Netherlands/22006859-001/2	A / H5N1	Europe / Nethe	Anser anse	Rene Heutink ('	Wageningen Bi	Beerens, Nar	Wageningen	2022-04-11
EPI_ISL_82156	A/Bar-headed Goose/Tibet/XZ1131/2021	A / H5N1	Asia / China / T	Anser indic	Hongliang Chai	Northeast Fores		College of W	2021-05-20
EPI_ISL_82156	A/Bar-headed Goose/Tibet/XZ901/2021	A / H5N1	Asia / China / T	Anser indic	Hongliang Chai	Northeast Fores		College of W	2021-05-20
EPI_ISL_82156	A/Bar-headed Goose/Tibet/XZQ18-1/2021	A / H5N8	Asia / China / T	Anser indic	Hongliang Chai	Northeast Fores		College of W	2021-05-17
EPI_ISL_82156	A/Bar-headed Goose/Tibet/XZQ17-1/2021	A / H5N8	Asia / China / T	Anser indic	Hongliang Chai	Northeast Fores		College of W	2021-05-17
EPI_ISL_82156	A/Bar-headed Goose/Tibet/XZQ13-1/2021	A / H5N8	Asia / China / T	Anser indic	Hongliang Chai	Northeast Fores		College of W	2021-05-17
EPI_ISL_82156	A/Bar-headed Goose/Tibet/XZ181/2021	A / H5N8	Asia / China / T	Anser indic	Hongliang Chai	Northeast Fores		College of W	2021-05-15
EPI_ISL_82156	A/Bar-headed Goose/Tibet/XZ81/2021	A / H5N8	Asia / China / T	Anser indic	Hongliang Chai	Northeast Fores		College of W	2021-05-15

EPI_ISL_82156	A/Bar-headed Goose/Tibet/XZ71/2021	A / H5N8	Asia / China / T	Anser indic	Hongliang Chai	Northeast Fores		College of W	2021-05-15
EPI_ISL_82156	A/Bar-headed Goose/Tibet/XZ6/2021	A / H5N8	Asia / China / T	Anser indic	Hongliang Chai	Northeast Fores		College of W	2021-05-15
EPI_ISL_82156	A/Bar-headed Goose/Tibet/XZQ10-1/2021	A / H5N8	Asia / China / T	Anser indic	Hongliang Chai	Northeast Fores		College of W	2021-05-15
EPI_ISL_82156	A/Bar-headed Goose/Tibet/XZQ9-1/2021	A / H5N8	Asia / China / T	Anser indic	Hongliang Chai	Northeast Fores		College of W	2021-05-15
EPI_ISL_82156	A/Bar-headed Goose/Tibet/XZQ8-1/2021	A / H5N8	Asia / China / T	Anser indic	Hongliang Chai	Northeast Fores		College of W	2021-05-15
EPI_ISL_82156	A/Bar-headed Goose/Tibet/XZQ7/2021	A / H5N8	Asia / China / T	Anser indic	Hongliang Chai	Northeast Fores		College of W	2021-05-15
EPI_ISL_82156	A/Bar-headed Goose/Tibet/XZQ5-1/2021	A / H5N8	Asia / China / T	Anser indic	Hongliang Chai	Northeast Fores		College of W	2021-05-15
EPI_ISL_14761	A/guinea_fowl/Italy/22VIR205-4/2022	A / H5N1	Europe / Italy	Guineafowl	Giacomo Barbi	Istituto Zooprof	Barbierato, G	Istituto Zoopr	2022-01-05
EPI_ISL_14760	A/guinea_fowl/Italy/21VIR11383-1/2021	A / H5N1	Europe / Italy	Guineafowl	Giacomo Barbi	Istituto Zooprof	Barbierato, G	Istituto Zoopr	2021-12-16
EPI_ISL_14760	A/guinea_fowl/Italy/21VIR11298/2021	A / H5N1	Europe / Italy	Guineafowl	Giacomo Barbi	Istituto Zooprof	Barbierato, G	Istituto Zoopr	2021-12-14
EPI_ISL_14760	A/guinea_fowl/Italy/21VIR10351/2021	A / H5N1	Europe / Italy	Guineafowl	Giacomo Barbi	Istituto Zooprof	Barbierato, G	Istituto Zoopr	2021-11-26
EPI_ISL_14760	A/Guinea_fowl/Italy/21VIR10231/2021	A / H5N1	Europe / Italy	Guineafowl	Giacomo Barbi	Istituto Zooprof	Barbierato, G	Istituto Zoopr	2021-11-24
EPI_ISL_14760	A/Guinea_fowl/Italy/21VIR10216/2021	A / H5N1	Europe / Italy	Guineafowl	Giacomo Barbi	Istituto Zooprof	Barbierato, G	Istituto Zoopr	2021-11-24
EPI_ISL_77330	A/black-headed gull/Germany-NI/AI05962/2021	A / H5N1	Europe / Germa	Gull	Jacqueline King	Friedrich-Loeffl		Lebensmittel	2021-10-12
EPI_ISL_25555	A/Chlidonias hybrida/Hubei/55/2020	A / H5N8	Asia / China / J	Chlidonias	Ma Liping (Wuh	Wuhan Institute	Jiason Xiong,	State Key La	2020-11-16
EPI_ISL_82156	A/Brown-headed Gull/Tibet/XZ19/2021	A / H5N8	Asia / China / T	Larus brunn	Hongliang Chai	Northeast Fores		College of W	2021-05-17
EPI_ISL_82156	A/Brown-headed Gull/Tibet/XZQ16-2/2021	A / H5N8	Asia / China / T	Larus brunn	Hongliang Chai	Northeast Fores		College of W	2021-05-17
EPI_ISL_82156	A/Brown-headed Gull/Tibet/XZQ15-2/2021	A / H5N8	Asia / China / T	Larus brunn	Hongliang Chai	Northeast Fores		College of W	2021-05-17
EPI_ISL_96908	A/great black-backed gull/Netherlands/220000	A / H5N1	Europe / Nethe	Larus marir	Rene Heutink (	Wageningen Bi	Beerens, Nan	Wageningen	2022-01-03
EPI_ISL_14761	A/pheasant/Italy/22VIR1953-1/2022	A / H5N1	Europe / Italy	Pheasant	Giacomo Barbi	Istituto Zooprof	Barbierato, G	Istituto Zoopr	2022-02-22
EPI_ISL_14760	A/pheasant/Italy/21VIR11165/2021	A / H5N1	Europe / Italy	Pheasant	Giacomo Barbi	Istituto Zooprof	Barbierato, G	Istituto Zoopr	2021-12-13
EPI_ISL_14760	A/pheasant/Italy/21VIR11162/2021	A / H5N1	Europe / Italy	Pheasant	Giacomo Barbi	Istituto Zooprof	Barbierato, G	Istituto Zoopr	2021-12-13
EPI_ISL_14760	A/pheasant/Italy/21VIR10359/2021	A / H5N1	Europe / Italy	Pheasant	Giacomo Barbi	Istituto Zooprof	Barbierato, G	Istituto Zoopr	2021-11-25
EPI_ISL_21773	A/peacock/Netherlands/21026542-001/2021	A / H5N8	Europe / Nethe	Pavo crista	Rene Heutink (	Wageningen Bi	Beerens, Nan	Wageningen	2021-04-07
EPI_ISL_46519	A/common pheasant /Sweden/SVA210923SZ0	A / H5N1	Europe / Swede	Phasanius	Siamak Zohari	National Veteri		National Vet	2021-09-22
EPI_ISL_15022	A/swan/Lithuania/1220PG1_22VIR7255-1/2022	A / H5N1	Europe / Lithua	Swan	Giacomo Barbi	Istituto Zooprof	Pileviciene, S	Lithuanian N	2022-02-09
EPI_ISL_14770	A/swan/Shandong/W3762/2020	A / H5N8	Asia / China	Swan			Wang,Y.; Wa		2020-11-23
EPI_ISL_14761	A/swan/Italy/22VIR1560/2022	A / H5N1	Europe / Italy	Swan	Giacomo Barbi	Istituto Zooprof	Barbierato, G	Istituto Zoopr	2022-01-27
EPI_ISL_29326	A/swan/Kazakhstan/1-267-20-B-Talg-52/2020	A / H5N8	Asia / Kazakhst	Swan	Alexandr Shevt	National Center	Asylulan,Amir	National Vet	2020-09-23
EPI_ISL_29326	A/swan/Kazakhstan/9-20-B-Talg-39/2020	A / H5N8	Asia / Kazakhst	Swan	Alexandr Shevt	National Center	Asylulan,Amir	National Vet	2020-09-21
EPI_ISL_22348	A/whooper_swan/Romania/10362_21VIR2593-	A / H5N5	Europe / Roma	Swan	Bianca Zecchin	Istituto Zooprof	Onita, I.; Neic	Istituto Zoopr	2021-01-22
EPI_ISL_22348	A/whooper_swan/Romania/10213_21VIR2593-	A / H5N5	Europe / Roma	Swan	Bianca Zecchin	Istituto Zooprof	Onita, I.; Neic	Istituto Zoopr	2021-01-14
EPI_ISL_22348	A/whooper_swan/Romania/10171_21VIR2593-	A / H5N5	Europe / Roma	Swan	Bianca Zecchin	Istituto Zooprof	Onita, I.; Neic	Istituto Zoopr	2021-01-12
EPI_ISL_22348	A/whooper_swan/Romania/10122_21VIR2593-	A / H5N5	Europe / Roma	Swan	Bianca Zecchin	Istituto Zooprof	Onita, I.; Neic	Istituto Zoopr	2021-01-08
EPI_ISL_17199	A/swan/Lithuania/2306PG1_21VIR2606-7/2021	A / H5N8	Europe / Lithua	Swan	Bianca Zecchin	Istituto Zooprof	Pridotkas, G.;	Istituto Zoopr	2021-03-19
EPI_ISL_17199	A/swan/Lithuania/1842PG1_21VIR2606-5/2021	A / H5N8	Europe / Lithua	Swan	Bianca Zecchin	Istituto Zooprof	Pridotkas, G.;	Istituto Zoopr	2021-03-19
EPI_ISL_17199	A/swan/Lithuania/1298PG1_21VIR2606-3/2021	A / H5N8	Europe / Lithua	Swan	Bianca Zecchin	Istituto Zooprof	Pridotkas, G.;	Istituto Zoopr	2021-02-26
EPI_ISL_17199	A/swan/Lithuania/1258PG1_21VIR2606-2/2021	A / H5N8	Europe / Lithua	Swan	Bianca Zecchin	Istituto Zooprof	Pridotkas, G.;	Istituto Zoopr	2021-02-26
EPI_ISL_26859	A/tundra swan/Hubei/BQ8/2020	A / H5N8	Asia / China / H	Cygnus col	Hongliang Chai	Northeast Fores		College of W	2020-11-16
EPI_ISL_26859	A/tundra swan/Hubei/BQ7/2020	A / H5N8	Asia / China / H	Cygnus col	Hongliang Chai	Northeast Fores		College of W	2020-11-16
EPI_ISL_26858	A/tundra swan/Hubei/BQ6/2020	A / H5N8	Asia / China / H	Cygnus col	Hongliang Chai	Northeast Fores		College of W	2020-11-16
EPI_ISL_26858	A/tundra swan/Hubei/BQ4/2020	A / H5N8	Asia / China / H	Cygnus col	Hongliang Chai	Northeast Fores		College of W	2020-11-11

EPI_ISL_26858	A/tundra swan/Hubei/BQ3/2020	A / H5N8	Asia / China / H	Cygnus col	Hongliang Chai	Northeast Fores		College of W	2020-11-09
EPI_ISL_26858	A/tundra swan/Hubei/BQ2/2020	A / H5N8	Asia / China / H	Cygnus col	Hongliang Chai	Northeast Fores		College of W	2020-11-04
EPI_ISL_25555	A/Cygnus columbianus/Hubei/50/2020	A / H5N8	Asia / China / J	Cygnus col	Ma Liping (Wuh	Wuhan Institute	Jiason Xiong,	State Key La	2020-11-12
EPI_ISL_25555	A/Cygnus columbianus/Hubei/51/2020	A / H5N8	Asia / China / J	Cygnus col	Ma Liping (Wuh	Wuhan Institute	Jiason Xiong,	State Key La	2020-11-12
EPI_ISL_25555	A/Cygnus columbianus/Hubei/52/2020	A / H5N8	Asia / China / J	Cygnus col	Ma Liping (Wuh	Wuhan Institute	Jiason Xiong,	State Key La	2020-11-16
EPI_ISL_25555	A/Cygnus columbianus/Hubei/53/2020	A / H5N8	Asia / China / J	Cygnus col	Ma Liping (Wuh	Wuhan Institute	Jiason Xiong,	State Key La	2020-11-16
EPI_ISL_25555	A/Cygnus columbianus/Hubei/56/2020	A / H5N8	Asia / China / J	Cygnus col	Ma Liping (Wuh	Wuhan Institute	Jiason Xiong,	State Key La	2020-11-04
EPI_ISL_25555	A/Cygnus columbianus/Hubei/116/2020	A / H5N8	Asia / China / J	Cygnus col	Ma Liping (Wuh	Wuhan Institute		State Key La	2020-11-16
EPI_ISL_12139	A/mute swan/Czech Republic/785/2022	A / H5N1	Europe / Czech	Cygnus olo	Alexander Nagy	State Veterinar	Alexander,Na	State Veterir	2022-01-10
EPI_ISL_69371	A/mute_swan/Poland/MB490-L1/2021	A / H5N1	Europe / Poland	Cygnus olo	Edyta Świętoń	National Veterii	E. Swieton, K	National Vet	2021-11-08
EPI_ISL_31359	A/swan/Hungary/9638/2021 (H5N8)	A / H5N8	Europe / Hunga	Cygnus olo	Katalin Szentpá	National Food (	Katalin,Szent	National Foo	2021-03-08
EPI_ISL_21134	A/mute_swan/Poland/MB268/2021	A / H5N8	Europe / Poland	Cygnus olo	Kamila Dziadek	National Veterii	Dziadek, K.; S	National Vet	2021-03-03
EPI_ISL_21131	A/mute_swan/Poland/MB189/2021	A / H5N8	Europe / Poland	Cygnus olo	Kamila Dziadek	National Veterii	Dziadek, K.; S	National Vet	2021-02-22
EPI_ISL_13992	A/mute swan/Czech Republic/4799/2021	A / H5N8	Europe / Czech	Cygnus olo	Alexander Nagy	State Veterinar	Nagy,A;Cerni	State Veterir	2021-03-06
EPI_ISL_13992	A/mute swan/Czech Republic/4607-2/2021	A / H5N8	Europe / Czech	Cygnus olo	Alexander Nagy	State Veterinar	Nagy,A;Cerni	State Veterir	2021-03-04
EPI_ISL_13992	A/mute swan/Czech Republic/4607-1/2021	A / H5N8	Europe / Czech	Cygnus olo	Alexander Nagy	State Veterinar	Nagy,A;Cerni	State Veterir	2021-03-04
EPI_ISL_13992	A/mute swan/Czech Republic/4606/2021	A / H5N8	Europe / Czech	Cygnus olo	Alexander Nagy	State Veterinar	Nagy,A;Cerni	State Veterir	2021-03-04
EPI_ISL_13992	A/mute swan/Czech Republic/4270/2021	A / H5N8	Europe / Czech	Cygnus olo	Alexander Nagy	State Veterinar	Nagy,A;Cerni	State Veterir	2021-03-02
EPI_ISL_13992	A/mute swan/Czech Republic/4100/2021	A / H5N8	Europe / Czech	Cygnus olo	Alexander Nagy	State Veterinar	Nagy,A;Cerni	State Veterir	2021-02-23
EPI_ISL_13992	A/mute swan/Czech Republic/3777/2021	A / H5N8	Europe / Czech	Cygnus olo	Alexander Nagy	State Veterinar	Nagy,A;Cerni	State Veterir	2021-02-22
EPI_ISL_13992	A/mute swan/Czech Republic/3549/2021	A / H5N8	Europe / Czech	Cygnus olo	Alexander Nagy	State Veterinar	Nagy,A;Cerni	State Veterir	2021-02-19
EPI_ISL_10804	A/mute swan/Czech Republic/3160-1/2021	A / H5N8	Europe / Czech	Cygnus olo	Alexander Nagy	State Veterinar	Nagy,A; Cern	State Veterir	2021-02-14
EPI_ISL_71105	A/mute swan/Netherlands/20019255-002/2020	A / H5N8	Europe / Nethe	Cygnus olo	Rene Heutink ('	Wageningen Bi	Beerens, Nar	Wageningen	2020-12-02
EPI_ISL_71105	A/mute swan/Netherlands/20019252-002/2020	A / H5N8	Europe / Nethe	Cygnus olo	Rene Heutink ('	Wageningen Bi	Beerens, Nar	Wageningen	2020-12-02
EPI_ISL_14761	A/turkey/Italy/22VIR171-2/2022	A / H5N1	Europe / Italy	Turkey	Giacomo Barbi	Istituto Zooprof	Barbierato, G	Istituto Zoop	2022-01-05
EPI_ISL_14761	A/turkey/Italy/IZSLT_22VIR366-3/2022	A / H5N1	Europe / Italy	Turkey	Giacomo Barbi	Istituto Zooprof	Barbierato, G	Istituto Zoop	2022-01-04
EPI_ISL_14761	A/turkey/Italy/21VIR11935/2021	A / H5N1	Europe / Italy	Turkey	Giacomo Barbi	Istituto Zooprof	Barbierato, G	Istituto Zoop	2021-12-29
EPI_ISL_14761	A/turkey/Italy/21VIR11772-7/2021	A / H5N1	Europe / Italy	Turkey	Giacomo Barbi	Istituto Zooprof	Barbierato, G	Istituto Zoop	2021-12-28
EPI_ISL_14761	A/turkey/Italy/21VIR11661-7/2021	A / H5N1	Europe / Italy	Turkey	Giacomo Barbi	Istituto Zooprof	Barbierato, G	Istituto Zoop	2021-12-26
EPI_ISL_14761	A/turkey/Italy/21VIR11690/2021	A / H5N1	Europe / Italy	Turkey	Giacomo Barbi	Istituto Zooprof	Barbierato, G	Istituto Zoop	2021-12-24
EPI_ISL_14761	A/turkey/Italy/21VIR11692/2021	A / H5N1	Europe / Italy	Turkey	Giacomo Barbi	Istituto Zooprof	Barbierato, G	Istituto Zoop	2021-12-23
EPI_ISL_14761	A/turkey/Italy/21VIR11691/2021	A / H5N1	Europe / Italy	Turkey	Giacomo Barbi	Istituto Zooprof	Barbierato, G	Istituto Zoop	2021-12-23
EPI_ISL_14761	A/turkey/Italy/21VIR11587-2/2021	A / H5N1	Europe / Italy	Turkey	Giacomo Barbi	Istituto Zooprof	Barbierato, G	Istituto Zoop	2021-12-23
EPI_ISL_14761	A/turkey/Italy/21VIR11587-1/2021	A / H5N1	Europe / Italy	Turkey	Giacomo Barbi	Istituto Zooprof	Barbierato, G	Istituto Zoop	2021-12-23
EPI_ISL_14761	A/turkey/Italy/21VIR11586-1/2021	A / H5N1	Europe / Italy	Turkey	Giacomo Barbi	Istituto Zooprof	Barbierato, G	Istituto Zoop	2021-12-23
EPI_ISL_14761	A/turkey/Italy/21VIR11596-3/2021	A / H5N1	Europe / Italy	Turkey	Giacomo Barbi	Istituto Zooprof	Barbierato, G	Istituto Zoop	2021-12-22
EPI_ISL_14760	A/turkey/Italy/21VIR11504/2021	A / H5N1	Europe / Italy	Turkey	Giacomo Barbi	Istituto Zooprof	Barbierato, G	Istituto Zoop	2021-12-21
EPI_ISL_14760	A/turkey/Italy/21VIR11500-3/2021	A / H5N1	Europe / Italy	Turkey	Giacomo Barbi	Istituto Zooprof	Barbierato, G	Istituto Zoop	2021-12-21
EPI_ISL_14760	A/turkey/Italy/21VIR11495-5/2021	A / H5N1	Europe / Italy	Turkey	Giacomo Barbi	Istituto Zooprof	Barbierato, G	Istituto Zoop	2021-12-21
EPI_ISL_14760	A/turkey/Italy/21VIR11492-4/2021	A / H5N1	Europe / Italy	Turkey	Giacomo Barbi	Istituto Zooprof	Barbierato, G	Istituto Zoop	2021-12-21
EPI_ISL_14760	A/turkey/Italy/21VIR11503-1/2021	A / H5N1	Europe / Italy	Turkey	Giacomo Barbi	Istituto Zooprof	Barbierato, G	Istituto Zoop	2021-12-21
EPI_ISL_14760	A/turkey/Italy/21VIR11499-5/2021	A / H5N1	Europe / Italy	Turkey	Giacomo Barbi	Istituto Zooprof	Barbierato, G	Istituto Zoop	2021-12-21

EPI_ISL_1476(	A/turkey/Italy/21VIR11506/2022	A / H5N1	Europe / Italy	Turkey	Giacomo Barbi	Istituto Zooprof	Barbierato, G		Istituto Zoop	2021-12-20
EPI_ISL_1476(	A/turkey/Italy/21VIR11511/2021	A / H5N1	Europe / Italy	Turkey	Giacomo Barbi	Istituto Zooprof	Barbierato, G		Istituto Zoop	2021-12-20
EPI_ISL_1476(	A/turkey/Italy/21VIR11440-2/2021	A / H5N1	Europe / Italy	Turkey	Giacomo Barbi	Istituto Zooprof	Barbierato, G		Istituto Zoop	2021-12-20
EPI_ISL_1476(	A/turkey/Italy/21VIR11510/2021	A / H5N1	Europe / Italy	Turkey	Giacomo Barbi	Istituto Zooprof	Barbierato, G		Istituto Zoop	2021-12-20
EPI_ISL_1476(	A/turkey/Italy/21VIR11509/2021	A / H5N1	Europe / Italy	Turkey	Giacomo Barbi	Istituto Zooprof	Barbierato, G		Istituto Zoop	2021-12-20
EPI_ISL_1476(	A/turkey/Italy/21VIR11396-1/2021	A / H5N1	Europe / Italy	Turkey	Giacomo Barbi	Istituto Zooprof	Barbierato, G		Istituto Zoop	2021-12-16
EPI_ISL_1476(	A/turkey/Italy/21VIR11386-1/2021	A / H5N1	Europe / Italy	Turkey	Giacomo Barbi	Istituto Zooprof	Barbierato, G		Istituto Zoop	2021-12-16
EPI_ISL_1476(	A/turkey/Italy/21VIR11393-3/2021	A / H5N1	Europe / Italy	Turkey	Giacomo Barbi	Istituto Zooprof	Barbierato, G		Istituto Zoop	2021-12-16
EPI_ISL_1476(	A/turkey/Italy/21VIR11392-2/2021	A / H5N1	Europe / Italy	Turkey	Giacomo Barbi	Istituto Zooprof	Barbierato, G		Istituto Zoop	2021-12-16
EPI_ISL_1476(	A/turkey/Italy/21VIR11391-3/2021	A / H5N1	Europe / Italy	Turkey	Giacomo Barbi	Istituto Zooprof	Barbierato, G		Istituto Zoop	2021-12-16
EPI_ISL_1476(	A/turkey/Italy/21VIR11387-1/2021	A / H5N1	Europe / Italy	Turkey	Giacomo Barbi	Istituto Zooprof	Barbierato, G		Istituto Zoop	2021-12-16
EPI_ISL_1476(	A/turkey/Italy/21VIR11384-1/2021	A / H5N1	Europe / Italy	Turkey	Giacomo Barbi	Istituto Zooprof	Barbierato, G		Istituto Zoop	2021-12-16
EPI_ISL_1476(	A/turkey/Italy/21VIR11254-1/2021	A / H5N1	Europe / Italy	Turkey	Giacomo Barbi	Istituto Zooprof	Barbierato, G		Istituto Zoop	2021-12-15
EPI_ISL_1476(	A/turkey/Italy/21VIR11252-1/2021	A / H5N1	Europe / Italy	Turkey	Giacomo Barbi	Istituto Zooprof	Barbierato, G		Istituto Zoop	2021-12-15
EPI_ISL_1476(	A/turkey/Italy/21VIR11251-1/2021	A / H5N1	Europe / Italy	Turkey	Giacomo Barbi	Istituto Zooprof	Barbierato, G		Istituto Zoop	2021-12-15
EPI_ISL_1476(	A/turkey/Italy/21VIR11302/2021	A / H5N1	Europe / Italy	Turkey	Giacomo Barbi	Istituto Zooprof	Barbierato, G		Istituto Zoop	2021-12-14
EPI_ISL_1476(	A/turkey/Italy/21VIR11237/2021	A / H5N1	Europe / Italy	Turkey	Giacomo Barbi	Istituto Zooprof	Barbierato, G		Istituto Zoop	2021-12-14
EPI_ISL_1476(	A/turkey/Italy/21VIR11163/2021	A / H5N1	Europe / Italy	Turkey	Giacomo Barbi	Istituto Zooprof	Barbierato, G		Istituto Zoop	2021-12-13
EPI_ISL_1476(	A/turkey/Italy/21VIR11058/2021	A / H5N1	Europe / Italy	Turkey	Giacomo Barbi	Istituto Zooprof	Barbierato, G		Istituto Zoop	2021-12-13
EPI_ISL_1476(	A/turkey/Italy/21VIR11164/2021	A / H5N1	Europe / Italy	Turkey	Giacomo Barbi	Istituto Zooprof	Barbierato, G		Istituto Zoop	2021-12-13
EPI_ISL_1476(	A/turkey/Italy/21VIR11166/2021	A / H5N1	Europe / Italy	Turkey	Giacomo Barbi	Istituto Zooprof	Barbierato, G		Istituto Zoop	2021-12-13
EPI_ISL_1476(	A/turkey/Italy/21VIR11007-1/2021	A / H5N1	Europe / Italy	Turkey	Giacomo Barbi	Istituto Zooprof	Barbierato, G		Istituto Zoop	2021-12-12
EPI_ISL_1476(	A/turkey/Italy/21VIR11006-1/2021	A / H5N1	Europe / Italy	Turkey	Giacomo Barbi	Istituto Zooprof	Barbierato, G		Istituto Zoop	2021-12-12
EPI_ISL_1476(	A/turkey/Italy/21VIR11077-1/2021	A / H5N1	Europe / Italy	Turkey	Giacomo Barbi	Istituto Zooprof	Barbierato, G		Istituto Zoop	2021-12-10
EPI_ISL_1476(	A/turkey/Italy/21VIR10988-1/2021	A / H5N1	Europe / Italy	Turkey	Giacomo Barbi	Istituto Zooprof	Barbierato, G		Istituto Zoop	2021-12-09
EPI_ISL_1476(	A/turkey/Italy/21VIR10970-3/2021	A / H5N1	Europe / Italy	Turkey	Giacomo Barbi	Istituto Zooprof	Barbierato, G		Istituto Zoop	2021-12-09
EPI_ISL_1476(	A/turkey/Italy/21VIR10847-1/2021	A / H5N1	Europe / Italy	Turkey	Giacomo Barbi	Istituto Zooprof	Barbierato, G		Istituto Zoop	2021-12-08
EPI_ISL_1476(	A/turkey/Italy/21VIR10757-1/2021	A / H5N1	Europe / Italy	Turkey	Giacomo Barbi	Istituto Zooprof	Barbierato, G		Istituto Zoop	2021-12-07
EPI_ISL_1476(	A/turkey/Italy/21VIR10930/2021	A / H5N1	Europe / Italy	Turkey	Giacomo Barbi	Istituto Zooprof	Barbierato, G		Istituto Zoop	2021-12-07
EPI_ISL_1476(	A/turkey/Italy/21VIR10929/2021	A / H5N1	Europe / Italy	Turkey	Giacomo Barbi	Istituto Zooprof	Barbierato, G		Istituto Zoop	2021-12-07
EPI_ISL_1476(	A/turkey/Italy/21VIR10810-1/2021	A / H5N1	Europe / Italy	Turkey	Giacomo Barbi	Istituto Zooprof	Barbierato, G		Istituto Zoop	2021-12-07
EPI_ISL_1476(	A/turkey/Italy/21VIR10737-1/2021	A / H5N1	Europe / Italy	Turkey	Giacomo Barbi	Istituto Zooprof	Barbierato, G		Istituto Zoop	2021-12-06
EPI_ISL_1476(	A/turkey/Italy/21VIR10697-1/2021	A / H5N1	Europe / Italy	Turkey	Giacomo Barbi	Istituto Zooprof	Barbierato, G		Istituto Zoop	2021-12-06
EPI_ISL_1476(	A/turkey/Italy/21VIR10855/2021	A / H5N1	Europe / Italy	Turkey	Giacomo Barbi	Istituto Zooprof	Barbierato, G		Istituto Zoop	2021-12-06
EPI_ISL_1476(	A/turkey/Italy/21VIR10854/2021	A / H5N1	Europe / Italy	Turkey	Giacomo Barbi	Istituto Zooprof	Barbierato, G		Istituto Zoop	2021-12-06
EPI_ISL_1476(	A/turkey/Italy/21VIR10857/2021	A / H5N1	Europe / Italy	Turkey	Giacomo Barbi	Istituto Zooprof	Barbierato, G		Istituto Zoop	2021-12-06
EPI_ISL_1476(	A/turkey/Italy/21VIR10852/2021	A / H5N1	Europe / Italy	Turkey	Giacomo Barbi	Istituto Zooprof	Barbierato, G		Istituto Zoop	2021-12-06
EPI_ISL_1476(	A/turkey/Italy/21VIR10856/2021	A / H5N1	Europe / Italy	Turkey	Giacomo Barbi	Istituto Zooprof	Barbierato, G		Istituto Zoop	2021-12-06
EPI_ISL_1476(	A/turkey/Italy/21VIR10699-1/2021	A / H5N1	Europe / Italy	Turkey	Giacomo Barbi	Istituto Zooprof	Barbierato, G		Istituto Zoop	2021-12-06
EPI_ISL_1476(	A/turkey/Italy/21VIR10645-15/2021	A / H5N1	Europe / Italy	Turkey	Giacomo Barbi	Istituto Zooprof	Barbierato, G		Istituto Zoop	2021-12-05
EPI_ISL_1476(	A/turkey/Italy/21VIR10636-17/2021	A / H5N1	Europe / Italy	Turkey	Giacomo Barbi	Istituto Zooprof	Barbierato, G		Istituto Zoop	2021-12-04
EPI_ISL_1476(	A/turkey/Italy/21VIR10621-1/2021	A / H5N1	Europe / Italy	Turkey	Giacomo Barbi	Istituto Zooprof	Barbierato, G		Istituto Zoop	2021-12-02

EPI_ISL_1476(	A/turkey/Italy/21VIR10617-2/2021	A / H5N1	Europe / Italy	Turkey	Giacomo Barbi	Istituto Zooprof	Barbierato, G	Istituto Zoop	2021-12-02
EPI_ISL_1476(	A/turkey/Italy/21VIR10622-1/2021	A / H5N1	Europe / Italy	Turkey	Giacomo Barbi	Istituto Zooprof	Barbierato, G	Istituto Zoop	2021-12-02
EPI_ISL_1476(	A/turkey/Italy/21VIR10459-1/2021	A / H5N1	Europe / Italy	Turkey	Giacomo Barbi	Istituto Zooprof	Barbierato, G	Istituto Zoop	2021-12-01
EPI_ISL_1476(	A/turkey/Italy/21VIR10524-1/2021	A / H5N1	Europe / Italy	Turkey	Giacomo Barbi	Istituto Zooprof	Barbierato, G	Istituto Zoop	2021-12-01
EPI_ISL_1476(	A/turkey/Italy/21VIR10471/2021	A / H5N1	Europe / Italy	Turkey	Giacomo Barbi	Istituto Zooprof	Barbierato, G	Istituto Zoop	2021-11-30
EPI_ISL_1476(	A/turkey/Italy/21VIR10398/2021	A / H5N1	Europe / Italy	Turkey	Giacomo Barbi	Istituto Zooprof	Barbierato, G	Istituto Zoop	2021-11-29
EPI_ISL_1476(	A/turkey/Italy/21VIR10397/2021	A / H5N1	Europe / Italy	Turkey	Giacomo Barbi	Istituto Zooprof	Barbierato, G	Istituto Zoop	2021-11-29
EPI_ISL_1476(	A/turkey/Italy/21VIR10395/2021	A / H5N1	Europe / Italy	Turkey	Giacomo Barbi	Istituto Zooprof	Barbierato, G	Istituto Zoop	2021-11-29
EPI_ISL_1476(	A/turkey/Italy/21VIR10396/2021	A / H5N1	Europe / Italy	Turkey	Giacomo Barbi	Istituto Zooprof	Barbierato, G	Istituto Zoop	2021-11-29
EPI_ISL_1476(	A/turkey/Italy/21VIR10338/2021	A / H5N1	Europe / Italy	Turkey	Giacomo Barbi	Istituto Zooprof	Barbierato, G	Istituto Zoop	2021-11-29
EPI_ISL_1476(	A/turkey/Italy/21VIR10103/2021	A / H5N1	Europe / Italy	Turkey	Giacomo Barbi	Istituto Zooprof	Barbierato, G	Istituto Zoop	2021-11-24
EPI_ISL_1476(	A/turkey/Italy/21VIR9901-3/2021	A / H5N1	Europe / Italy	Turkey	Giacomo Barbi	Istituto Zooprof	Barbierato, G	Istituto Zoop	2021-11-23
EPI_ISL_1476(	A/turkey/Italy/21VIR10092/2021	A / H5N1	Europe / Italy	Turkey	Giacomo Barbi	Istituto Zooprof	Barbierato, G	Istituto Zoop	2021-11-23
EPI_ISL_1476(	A/turkey/Italy/21VIR10095/2021	A / H5N1	Europe / Italy	Turkey	Giacomo Barbi	Istituto Zooprof	Barbierato, G	Istituto Zoop	2021-11-23
EPI_ISL_1476(	A/turkey/Italy/21VIR10097/2021	A / H5N1	Europe / Italy	Turkey	Giacomo Barbi	Istituto Zooprof	Barbierato, G	Istituto Zoop	2021-11-23
EPI_ISL_1476(	A/turkey/Italy/21VIR9949-9/2021	A / H5N1	Europe / Italy	Turkey	Giacomo Barbi	Istituto Zooprof	Barbierato, G	Istituto Zoop	2021-11-23
EPI_ISL_1476(	A/turkey/Italy/21VIR9894-3/2021	A / H5N1	Europe / Italy	Turkey	Giacomo Barbi	Istituto Zooprof	Barbierato, G	Istituto Zoop	2021-11-23
EPI_ISL_1476(	A/turkey/Italy/21VIR10001/2021	A / H5N1	Europe / Italy	Turkey	Giacomo Barbi	Istituto Zooprof	Barbierato, G	Istituto Zoop	2021-11-22
EPI_ISL_1476(	A/turkey/Italy/21VIR9997/2021	A / H5N1	Europe / Italy	Turkey	Giacomo Barbi	Istituto Zooprof	Barbierato, G	Istituto Zoop	2021-11-22
EPI_ISL_1476(	A/turkey/Italy/21VIR9999/2021	A / H5N1	Europe / Italy	Turkey	Giacomo Barbi	Istituto Zooprof	Barbierato, G	Istituto Zoop	2021-11-22
EPI_ISL_1476(	A/turkey/Italy/21VIR9996/2021	A / H5N1	Europe / Italy	Turkey	Giacomo Barbi	Istituto Zooprof	Barbierato, G	Istituto Zoop	2021-11-22
EPI_ISL_1476(	A/turkey/Italy/21VIR9891-21/2021	A / H5N1	Europe / Italy	Turkey	Giacomo Barbi	Istituto Zooprof	Barbierato, G	Istituto Zoop	2021-11-22
EPI_ISL_1476(	A/turkey/Italy/21VIR9889-1/2021	A / H5N1	Europe / Italy	Turkey	Giacomo Barbi	Istituto Zooprof	Barbierato, G	Istituto Zoop	2021-11-22
EPI_ISL_1476(	A/turkey/Italy/21VIR9839-1/2021	A / H5N1	Europe / Italy	Turkey	Giacomo Barbi	Istituto Zooprof	Barbierato, G	Istituto Zoop	2021-11-21
EPI_ISL_1476(	A/turkey/Italy/21VIR9905-2/2021	A / H5N1	Europe / Italy	Turkey	Giacomo Barbi	Istituto Zooprof	Barbierato, G	Istituto Zoop	2021-11-19
EPI_ISL_1476(	A/turkey/Italy/21VIR9906-2/2021	A / H5N1	Europe / Italy	Turkey	Giacomo Barbi	Istituto Zooprof	Barbierato, G	Istituto Zoop	2021-11-19
EPI_ISL_1476(	A/turkey/Italy/21VIR9903-2/2021	A / H5N1	Europe / Italy	Turkey	Giacomo Barbi	Istituto Zooprof	Barbierato, G	Istituto Zoop	2021-11-18
EPI_ISL_1476(	A/turkey/Italy/21VIR9869-4/2021	A / H5N1	Europe / Italy	Turkey	Giacomo Barbi	Istituto Zooprof	Barbierato, G	Istituto Zoop	2021-11-18
EPI_ISL_1476(	A/turkey/Italy/21VIR9870-3/2021	A / H5N1	Europe / Italy	Turkey	Giacomo Barbi	Istituto Zooprof	Barbierato, G	Istituto Zoop	2021-11-18
EPI_ISL_1476(	A/turkey/Italy/21VIR9766-3/2021	A / H5N1	Europe / Italy	Turkey	Giacomo Barbi	Istituto Zooprof	Barbierato, G	Istituto Zoop	2021-11-17
EPI_ISL_1476(	A/turkey/Italy/21VIR9769-4/2021	A / H5N1	Europe / Italy	Turkey	Giacomo Barbi	Istituto Zooprof	Barbierato, G	Istituto Zoop	2021-11-17
EPI_ISL_1476(	A/turkey/Italy/21VIR9697-3_/2022	A / H5N1	Europe / Italy	Turkey	Giacomo Barbi	Istituto Zooprof	Barbierato, G	Istituto Zoop	2021-11-16
EPI_ISL_1476(	A/turkey/Italy/21VIR9696-2/2021	A / H5N1	Europe / Italy	Turkey	Giacomo Barbi	Istituto Zooprof	Barbierato, G	Istituto Zoop	2021-11-16
EPI_ISL_1476(	A/turkey/Italy/21VIR9693-3/2021	A / H5N1	Europe / Italy	Turkey	Giacomo Barbi	Istituto Zooprof	Barbierato, G	Istituto Zoop	2021-11-16
EPI_ISL_1476(	A/turkey/Italy/21VIR9695-4/2021	A / H5N1	Europe / Italy	Turkey	Giacomo Barbi	Istituto Zooprof	Barbierato, G	Istituto Zoop	2021-11-16
EPI_ISL_1476(	A/turkey/Italy/21VIR9692-16/2021	A / H5N1	Europe / Italy	Turkey	Giacomo Barbi	Istituto Zooprof	Barbierato, G	Istituto Zoop	2021-11-16
EPI_ISL_1476(	A/turkey/Italy/21VIR9694-2/2021	A / H5N1	Europe / Italy	Turkey	Giacomo Barbi	Istituto Zooprof	Barbierato, G	Istituto Zoop	2021-11-16
EPI_ISL_1476(	A/turkey/Italy/21VIR9647-2/2021	A / H5N1	Europe / Italy	Turkey	Giacomo Barbi	Istituto Zooprof	Barbierato, G	Istituto Zoop	2021-11-15
EPI_ISL_1476(	A/turkey/Italy/21VIR9646-2/2021	A / H5N1	Europe / Italy	Turkey	Giacomo Barbi	Istituto Zooprof	Barbierato, G	Istituto Zoop	2021-11-15
EPI_ISL_1476(	A/turkey/Italy/21VIR9650-2/2021	A / H5N1	Europe / Italy	Turkey	Giacomo Barbi	Istituto Zooprof	Barbierato, G	Istituto Zoop	2021-11-15
EPI_ISL_1476(	A/turkey/Italy/21VIR9651-2/2021	A / H5N1	Europe / Italy	Turkey	Giacomo Barbi	Istituto Zooprof	Barbierato, G	Istituto Zoop	2021-11-15
EPI_ISL_1476(	A/turkey/Italy/21VIR9648-2/2021	A / H5N1	Europe / Italy	Turkey	Giacomo Barbi	Istituto Zooprof	Barbierato, G	Istituto Zoop	2021-11-15

EPI_ISL_1476(	A/turkey/Italy/21VIR9579-10/2021	A / H5N1	Europe / Italy	Turkey	Giacomo Barbi	Istituto Zooprof	Barbierato, G	Istituto Zoop	2021-11-15
EPI_ISL_1476(	A/turkey/Italy/21VIR9645-2/2021	A / H5N1	Europe / Italy	Turkey	Giacomo Barbi	Istituto Zooprof	Barbierato, G	Istituto Zoop	2021-11-15
EPI_ISL_1476(	A/turkey/Italy/21VIR9644-2/2021	A / H5N1	Europe / Italy	Turkey	Giacomo Barbi	Istituto Zooprof	Barbierato, G	Istituto Zoop	2021-11-15
EPI_ISL_1476(	A/turkey/Italy/21VIR9643-2/2021	A / H5N1	Europe / Italy	Turkey	Giacomo Barbi	Istituto Zooprof	Barbierato, G	Istituto Zoop	2021-11-15
EPI_ISL_1476(	A/turkey/Italy/21VIR9520-1/2021	A / H5N1	Europe / Italy	Turkey	Giacomo Barbi	Istituto Zooprof	Barbierato, G	Istituto Zoop	2021-11-14
EPI_ISL_1476(	A/turkey/Italy/21VIR9519-1/2021	A / H5N1	Europe / Italy	Turkey	Giacomo Barbi	Istituto Zooprof	Barbierato, G	Istituto Zoop	2021-11-14
EPI_ISL_1476(	A/turkey/Italy/21VIR9518-1/2021	A / H5N1	Europe / Italy	Turkey	Giacomo Barbi	Istituto Zooprof	Barbierato, G	Istituto Zoop	2021-11-14
EPI_ISL_1476(	A/turkey/Italy/21VIR9608-2/2021	A / H5N1	Europe / Italy	Turkey	Giacomo Barbi	Istituto Zooprof	Barbierato, G	Istituto Zoop	2021-11-12
EPI_ISL_1476(	A/turkey/Italy/21VIR9605-4/2021	A / H5N1	Europe / Italy	Turkey	Giacomo Barbi	Istituto Zooprof	Barbierato, G	Istituto Zoop	2021-11-12
EPI_ISL_1476(	A/turkey/Italy/21VIR9609-2/2021	A / H5N1	Europe / Italy	Turkey	Giacomo Barbi	Istituto Zooprof	Barbierato, G	Istituto Zoop	2021-11-12
EPI_ISL_1476(	A/turkey/Italy/21VIR9604-3/2021	A / H5N1	Europe / Italy	Turkey	Giacomo Barbi	Istituto Zooprof	Barbierato, G	Istituto Zoop	2021-11-12
EPI_ISL_1476(	A/turkey/Italy/21VIR9606-2/2021	A / H5N1	Europe / Italy	Turkey	Giacomo Barbi	Istituto Zooprof	Barbierato, G	Istituto Zoop	2021-11-12
EPI_ISL_1476(	A/turkey/Italy/21VIR9607-2/2021	A / H5N1	Europe / Italy	Turkey	Giacomo Barbi	Istituto Zooprof	Barbierato, G	Istituto Zoop	2021-11-12
EPI_ISL_1476(	A/turkey/Italy/21VIR9475-1/2021	A / H5N1	Europe / Italy	Turkey	Giacomo Barbi	Istituto Zooprof	Barbierato, G	Istituto Zoop	2021-11-10
EPI_ISL_1476(	A/turkey/Italy/21VIR9476-1/2021	A / H5N1	Europe / Italy	Turkey	Giacomo Barbi	Istituto Zooprof	Barbierato, G	Istituto Zoop	2021-11-10
EPI_ISL_1476(	A/turkey/Italy/21VIR9426-3/2021	A / H5N1	Europe / Italy	Turkey	Giacomo Barbi	Istituto Zooprof	Barbierato, G	Istituto Zoop	2021-11-09
EPI_ISL_1476(	A/turkey/Italy/21VIR9372-1/2021	A / H5N1	Europe / Italy	Turkey	Giacomo Barbi	Istituto Zooprof	Barbierato, G	Istituto Zoop	2021-11-08
EPI_ISL_1476(	A/turkey/Italy/21VIR9215-1/2021	A / H5N1	Europe / Italy	Turkey	Giacomo Barbi	Istituto Zooprof	Barbierato, G	Istituto Zoop	2021-11-06
EPI_ISL_1476(	A/turkey/Italy/21VIR9213-1/2021	A / H5N1	Europe / Italy	Turkey	Giacomo Barbi	Istituto Zooprof	Barbierato, G	Istituto Zoop	2021-11-06
EPI_ISL_1476(	A/turkey/Italy/21VIR9217-1/2021	A / H5N1	Europe / Italy	Turkey	Giacomo Barbi	Istituto Zooprof	Barbierato, G	Istituto Zoop	2021-11-06
EPI_ISL_1476(	A/turkey/Italy/21VIR9211-1/2021	A / H5N1	Europe / Italy	Turkey	Giacomo Barbi	Istituto Zooprof	Barbierato, G	Istituto Zoop	2021-11-05
EPI_ISL_1476(	A/turkey/Italy/21VIR9209-1/2021	A / H5N1	Europe / Italy	Turkey	Giacomo Barbi	Istituto Zooprof	Barbierato, G	Istituto Zoop	2021-11-05
EPI_ISL_1476(	A/turkey/Italy/21VIR9144-2/2021	A / H5N1	Europe / Italy	Turkey	Giacomo Barbi	Istituto Zooprof	Barbierato, G	Istituto Zoop	2021-11-03
EPI_ISL_1476(	A/turkey/Italy/21VIR9073-3/2021	A / H5N1	Europe / Italy	Turkey	Giacomo Barbi	Istituto Zooprof	Barbierato, G	Istituto Zoop	2021-11-02
EPI_ISL_1476(	A/turkey/Italy/21VIR8926-2/2021	A / H5N1	Europe / Italy	Turkey	Giacomo Barbi	Istituto Zooprof	Barbierato, G	Istituto Zoop	2021-10-30
EPI_ISL_1476(	A/turkey/Italy/21VIR8926-1/2021	A / H5N1	Europe / Italy	Turkey	Giacomo Barbi	Istituto Zooprof	Barbierato, G	Istituto Zoop	2021-10-30
EPI_ISL_1476(	A/turkey/Italy/21VIR8925-6/2021	A / H5N1	Europe / Italy	Turkey	Giacomo Barbi	Istituto Zooprof	Barbierato, G	Istituto Zoop	2021-10-29
EPI_ISL_1476(	A/turkey/Italy/21VIR8816-1/2021	A / H5N1	Europe / Italy	Turkey	Giacomo Barbi	Istituto Zooprof	Barbierato, G	Istituto Zoop	2021-10-25
EPI_ISL_1476(	A/turkey/Italy/21VIR8728-1/2021	A / H5N1	Europe / Italy	Turkey	Giacomo Barbi	Istituto Zooprof	Barbierato, G	Istituto Zoop	2021-10-22
EPI_ISL_1399(	A/turkey/Spain/1097-7_22VIR6312-36/2022	A / H5N1	Europe / Spain	Turkey	Giacomo Barbi	Istituto Zooprof	Ruano, M.J.;	Laboratorio (	2022-03-16
EPI_ISL_1399(	A/turkey/Spain/859-8_22VIR6312-30/2022	A / H5N1	Europe / Spain	Turkey	Giacomo Barbi	Istituto Zooprof	Ruano, M.J.;	Laboratorio (	2022-03-02
EPI_ISL_1399(	A/turkey/Spain/830-5_22VIR6312-29/2022	A / H5N1	Europe / Spain	Turkey	Giacomo Barbi	Istituto Zooprof	Ruano, M.J.;	Laboratorio (	2022-03-01
EPI_ISL_1399(	A/turkey/Spain/805-8_22VIR6312-28/2022	A / H5N1	Europe / Spain	Turkey	Giacomo Barbi	Istituto Zooprof	Ruano, M.J.;	Laboratorio (	2022-02-26
EPI_ISL_1399(	A/turkey/Spain/803-5_22VIR6312-27/2022	A / H5N1	Europe / Spain	Turkey	Giacomo Barbi	Istituto Zooprof	Ruano, M.J.;	Laboratorio (	2022-02-26
EPI_ISL_1399(	A/turkey/Spain/802-7_22VIR6312-26/2022	A / H5N1	Europe / Spain	Turkey	Giacomo Barbi	Istituto Zooprof	Ruano, M.J.;	Laboratorio (	2022-02-26
EPI_ISL_1399(	A/turkey/Spain/801-6_22VIR6312-25/2022	A / H5N1	Europe / Spain	Turkey	Giacomo Barbi	Istituto Zooprof	Ruano, M.J.;	Laboratorio (	2022-02-23
EPI_ISL_1399(	A/turkey/Spain/712-7_22VIR6312-24/2022	A / H5N1	Europe / Spain	Turkey	Giacomo Barbi	Istituto Zooprof	Ruano, M.J.;	Laboratorio (	2022-02-21
EPI_ISL_1399(	A/turkey/Spain/711-5_22VIR6312-23/2022	A / H5N1	Europe / Spain	Turkey	Giacomo Barbi	Istituto Zooprof	Ruano, M.J.;	Laboratorio (	2022-02-21
EPI_ISL_12917	A/turkey/Israel/564/2021	A / H5N1	Asia / Israel / N	Turkey	Irina Shkoda (K	Kimron Veterina	Shkoda,I; Lu	Kimron Vete	2021-10-29
EPI_ISL_51441	A/turkey/Germany-NI/AI03160/2021	A / H5N8	Europe / Germa	Turkey	Jacqueline King	Friedrich-Loeffl		Lebensmittel	2021-03-21
EPI_ISL_51436	A/turkey/Germany-NI/AI03209/2021	A / H5N8	Europe / Germa	Turkey	Jacqueline King	Friedrich-Loeffl		Lebensmittel	2021-03-23
EPI_ISL_51435	A/turkey/Germany-MV/AI03535/2021	A / H5N8	Europe / Germa	Turkey	Jacqueline King	Friedrich-Loeffl		Landesamt f	2021-03-26

EPI_ISL_51434	A/turkey/Germany-BB/AI02434/2021	A / H5N8	Europe / Germa	Turkey	Jacqueline King	Friedrich-Loeffl		Landeslabor	2021-03-10
EPI_ISL_51431	A/turkey/Germany-NI/AI02424/2021	A / H5N8	Europe / Germa	Turkey	Jacqueline King	Friedrich-Loeffl		Lebensmittel	2021-03-10
EPI_ISL_51429	A/turkey/Germany-MV/AI02458/2021	A / H5N8	Europe / Germa	Turkey	Jacqueline King	Friedrich-Loeffl		Landesamt fü	2021-03-10
EPI_ISL_51361	A/turkey/Germany-NI/AI02933/2021	A / H5N8	Europe / Germa	Turkey	Jacqueline King	Friedrich-Loeffl		Lebensmittel	2021-03-15
EPI_ISL_51346	A/turkey/Germany-NI/AI02943/2021	A / H5N8	Europe / Germa	Turkey	Jacqueline King	Friedrich-Loeffl		Lebensmittel	2021-03-15
EPI_ISL_50953	A/turkey/Germany-NI/AI00439/2021	A / H5N8	Europe / Germa	Turkey	Jacqueline King	Friedrich-Loeffl		Lebensmittel	2021-01-11
EPI_ISL_50953	A/turkey/Germany-NI/AI00429/2021	A / H5N8	Europe / Germa	Turkey	Jacqueline King	Friedrich-Loeffl		Lebensmittel	2021-01-08
EPI_ISL_50953	A/turkey/Germany-NI/AI00406/2021	A / H5N8	Europe / Germa	Turkey	Jacqueline King	Friedrich-Loeffl		Lebensmittel	2021-01-06
EPI_ISL_50953	A/turkey/Germany-NI/AI00402/2021	A / H5N8	Europe / Germa	Turkey	Jacqueline King	Friedrich-Loeffl		Lebensmittel	2021-01-06
EPI_ISL_41741	A/turkey/Germany-NI/AI00334/2020	A / H5N8	Europe / Germa	Turkey	Jacqueline King	Friedrich-Loeffl		Lebensmittel	2020-03-20
EPI_ISL_15023	A/stork/Lithuania/4371PG1_22VIR7255-3/2022	A / H5N1	Europe / Lithua	Other aviar	Giacomo Barbie	Istituto Zooprof	Pileviciene, S	Lithuanian N	2022-04-19
EPI_ISL_14761	A/peacock/Italy/22VIR1953-2/2022	A / H5N1	Europe / Italy	Other aviar	Giacomo Barbie	Istituto Zooprof	Barbierato, G	Istituto Zoopr	2022-02-22
EPI_ISL_14761	A/laying_hen/Italy/22VIR1521-2/2022	A / H5N1	Europe / Italy	Other aviar	Giacomo Barbie	Istituto Zooprof	Barbierato, G	Istituto Zoopr	2022-02-14
EPI_ISL_14761	A/herring_gull/Italy/22VIR1710-3/2022	A / H5N1	Europe / Italy	Other aviar	Giacomo Barbie	Istituto Zooprof	Barbierato, G	Istituto Zoopr	2022-02-01
EPI_ISL_14761	A/buzzard/Italy/22VIR767-2/2022	A / H5N1	Europe / Italy	Other aviar	Giacomo Barbie	Istituto Zooprof	Barbierato, G	Istituto Zoopr	2022-01-24
EPI_ISL_14761	A/buzzard/Italy/22VIR767-1/2022	A / H5N1	Europe / Italy	Other aviar	Giacomo Barbie	Istituto Zooprof	Barbierato, G	Istituto Zoopr	2022-01-24
EPI_ISL_14761	A/hawk/Italy/22VIR428-7/2022	A / H5N1	Europe / Italy	Other aviar	Giacomo Barbie	Istituto Zooprof	Barbierato, G	Istituto Zoopr	2022-01-14
EPI_ISL_14761	A/hawk/Italy/22VIR428-5/2022	A / H5N1	Europe / Italy	Other aviar	Giacomo Barbie	Istituto Zooprof	Barbierato, G	Istituto Zoopr	2022-01-14
EPI_ISL_14761	A/laying_hen/Italy/22VIR204-1/2022	A / H5N1	Europe / Italy	Other aviar	Giacomo Barbie	Istituto Zooprof	Barbierato, G	Istituto Zoopr	2022-01-10
EPI_ISL_14761	A/laying_hen/Italy/22VIR203-3/2022	A / H5N1	Europe / Italy	Other aviar	Giacomo Barbie	Istituto Zooprof	Barbierato, G	Istituto Zoopr	2022-01-05
EPI_ISL_14761	A/laying_hen/Italy/22VIR48-1/2022	A / H5N1	Europe / Italy	Other aviar	Giacomo Barbie	Istituto Zooprof	Barbierato, G	Istituto Zoopr	2022-01-03
EPI_ISL_14761	A/laying_hen/Italy/21VIR11801-7/2021	A / H5N1	Europe / Italy	Other aviar	Giacomo Barbie	Istituto Zooprof	Barbierato, G	Istituto Zoopr	2021-12-27
EPI_ISL_14761	A/laying_hen/Italy/21VIR11592-1/2021	A / H5N1	Europe / Italy	Other aviar	Giacomo Barbie	Istituto Zooprof	Barbierato, G	Istituto Zoopr	2021-12-23
EPI_ISL_14761	A/laying_hen/Italy/21VIR11597-1/2021	A / H5N1	Europe / Italy	Other aviar	Giacomo Barbie	Istituto Zooprof	Barbierato, G	Istituto Zoopr	2021-12-22
EPI_ISL_14761	A/laying_hen/Italy/21VIR11595-5/2021	A / H5N1	Europe / Italy	Other aviar	Giacomo Barbie	Istituto Zooprof	Barbierato, G	Istituto Zoopr	2021-12-22
EPI_ISL_14760	A/laying_hen/Italy/21VIR11498-1/2021	A / H5N1	Europe / Italy	Other aviar	Giacomo Barbie	Istituto Zooprof	Barbierato, G	Istituto Zoopr	2021-12-21
EPI_ISL_14760	A/laying_hen/Italy/21VIR11505-2/2021	A / H5N1	Europe / Italy	Other aviar	Giacomo Barbie	Istituto Zooprof	Barbierato, G	Istituto Zoopr	2021-12-21
EPI_ISL_14760	A/laying_hen/Italy/21VIR11486/2021	A / H5N1	Europe / Italy	Other aviar	Giacomo Barbie	Istituto Zooprof	Barbierato, G	Istituto Zoopr	2021-12-17
EPI_ISL_14760	A/laying_hen/Italy/21VIR11395-1/2021	A / H5N1	Europe / Italy	Other aviar	Giacomo Barbie	Istituto Zooprof	Barbierato, G	Istituto Zoopr	2021-12-16
EPI_ISL_14760	A/laying_hen/Italy/21VIR11394-1/2021	A / H5N1	Europe / Italy	Other aviar	Giacomo Barbie	Istituto Zooprof	Barbierato, G	Istituto Zoopr	2021-12-16
EPI_ISL_14760	A/laying_hen/Italy/21VIR11390-2/2021	A / H5N1	Europe / Italy	Other aviar	Giacomo Barbie	Istituto Zooprof	Barbierato, G	Istituto Zoopr	2021-12-16
EPI_ISL_14760	A/laying_hen/Italy/21VIR11299/2021	A / H5N1	Europe / Italy	Other aviar	Giacomo Barbie	Istituto Zooprof	Barbierato, G	Istituto Zoopr	2021-12-15
EPI_ISL_14760	A/laying_hen/Italy/21VIR11253-1/2021	A / H5N1	Europe / Italy	Other aviar	Giacomo Barbie	Istituto Zooprof	Barbierato, G	Istituto Zoopr	2021-12-15
EPI_ISL_14760	A/laying_hen/Italy/21VIR11300/2021	A / H5N1	Europe / Italy	Other aviar	Giacomo Barbie	Istituto Zooprof	Barbierato, G	Istituto Zoopr	2021-12-13
EPI_ISL_14760	A/laying_hen/Italy/21VIR11169/2021	A / H5N1	Europe / Italy	Other aviar	Giacomo Barbie	Istituto Zooprof	Barbierato, G	Istituto Zoopr	2021-12-13
EPI_ISL_14760	A/laying_hen/Italy/21VIR11168/2021	A / H5N1	Europe / Italy	Other aviar	Giacomo Barbie	Istituto Zooprof	Barbierato, G	Istituto Zoopr	2021-12-13
EPI_ISL_14760	A/laying_hen/Italy/21VIR11167/2021	A / H5N1	Europe / Italy	Other aviar	Giacomo Barbie	Istituto Zooprof	Barbierato, G	Istituto Zoopr	2021-12-13
EPI_ISL_14760	A/quail/Italy/21VIR10356/2021	A / H5N1	Europe / Italy	Other aviar	Giacomo Barbie	Istituto Zooprof	Barbierato, G	Istituto Zoopr	2021-11-26
EPI_ISL_14760	A/quail/Italy/21VIR9515-1/2021	A / H5N1	Europe / Italy	Other aviar	Giacomo Barbie	Istituto Zooprof	Barbierato, G	Istituto Zoopr	2021-11-13
EPI_ISL_14760	A/quail/Italy/21VIR9474-1/2021	A / H5N1	Europe / Italy	Other aviar	Giacomo Barbie	Istituto Zooprof	Barbierato, G	Istituto Zoopr	2021-11-09
EPI_ISL_13990	A/Anser_anser/Spain/1124-65_22VIR6312-9/2	A / H5N1	Europe / Spain	Other aviar	Giacomo Barbie	Istituto Zooprof	Ruano, M.J.;	Laboratorio C	2022-03-18
EPI_ISL_13990	A/Anser_anser/Spain/1035-5_22VIR6312-8/20	A / H5N1	Europe / Spain	Other aviar	Giacomo Barbie	Istituto Zooprof	Ruano, M.J.;	Laboratorio C	2022-03-14

EPI_ISL_1399	A/Ciconia_ciconia/Spain/971-6_22VIR6312-7/2	A / H5N1	Europe / Spain	Other avian	Giacomo Barbi	Istituto Zooprof	Ruano, M.J.;		Laboratorio	2022-02-28
EPI_ISL_1399	A/Anser_anser/Spain/863-2_22VIR6312-6/202	A / H5N1	Europe / Spain	Other avian	Giacomo Barbi	Istituto Zooprof	Ruano, M.J.;		Laboratorio	2022-03-02
EPI_ISL_1399	A/Ardea_cinerea/Spain/863-1_22VIR6312-5/2	A / H5N1	Europe / Spain	Other avian	Giacomo Barbi	Istituto Zooprof	Ruano, M.J.;		Laboratorio	2022-03-02
EPI_ISL_1399	A/Cygnus_olor/Spain/1950-10_22VIR6312-46/	A / H5N1	Europe / Spain	Other avian	Giacomo Barbi	Istituto Zooprof	Ruano, M.J.;		Laboratorio	2022-05-27
EPI_ISL_1399	A/Falco_peregrinus/Spain/1932-1_22VIR6312-	A / H5N1	Europe / Spain	Other avian	Giacomo Barbi	Istituto Zooprof	Ruano, M.J.;		Laboratorio	2022-05-18
EPI_ISL_1399	A/Ciconia_ciconia/Spain/853-14_22VIR6312-4	A / H5N1	Europe / Spain	Other avian	Giacomo Barbi	Istituto Zooprof	Ruano, M.J.;		Laboratorio	2022-02-21
EPI_ISL_1399	A/Anser_anser/Spain/638-6_22VIR6312-39/20	A / H5N1	Europe / Spain	Other avian	Giacomo Barbi	Istituto Zooprof	Ruano, M.J.;		Laboratorio	2022-02-13
EPI_ISL_1399	A/Anser_anser/Spain/750-4_22VIR6312-2/202	A / H5N1	Europe / Spain	Other avian	Giacomo Barbi	Istituto Zooprof	Ruano, M.J.;		Laboratorio	2022-02-02
EPI_ISL_1399	A/Anser_anser/Spain/2010-3_22VIR6312-21/2	A / H5N1	Europe / Spain	Other avian	Giacomo Barbi	Istituto Zooprof	Ruano, M.J.;		Laboratorio	2022-06-02
EPI_ISL_1399	A/Falco_peregrinus/Spain/2010-11_22VIR631	A / H5N1	Europe / Spain	Other avian	Giacomo Barbi	Istituto Zooprof	Ruano, M.J.;		Laboratorio	2022-05-30
EPI_ISL_1399	A/Gypaetus_barbatus/Spain/1956-25_22VIR63	A / H5N1	Europe / Spain	Other avian	Giacomo Barbi	Istituto Zooprof	Ruano, M.J.;		Laboratorio	2022-05-27
EPI_ISL_1399	A/Gypaetus_barbatus/Spain/1878-9_22VIR631	A / H5N1	Europe / Spain	Other avian	Giacomo Barbi	Istituto Zooprof	Ruano, M.J.;		Laboratorio	2022-05-11
EPI_ISL_1399	A/Rhea/Spain/1950-6_22VIR6312-17/2022	A / H5N1	Europe / Spain	Other avian	Giacomo Barbi	Istituto Zooprof	Ruano, M.J.;		Laboratorio	2022-05-27
EPI_ISL_1399	A/Cygnus_olor/Spain/1675-1_22VIR6312-14/2	A / H5N1	Europe / Spain	Other avian	Giacomo Barbi	Istituto Zooprof	Ruano, M.J.;		Laboratorio	2022-04-26
EPI_ISL_1399	A/wild_bird/Spain/1302-1_22VIR6312-11/2022	A / H5N1	Europe / Spain	Other avian	Giacomo Barbi	Istituto Zooprof	Ruano, M.J.;		Laboratorio	2022-03-30
EPI_ISL_1399	A/Anser_anser/Spain/1277-10_22VIR6312-10/	A / H5N1	Europe / Spain	Other avian	Giacomo Barbi	Istituto Zooprof	Ruano, M.J.;		Laboratorio	2022-03-26
EPI_ISL_5062	A/Eurasian oystercatcher/Germany-SH/AI0226	A / H5N8	Europe / Germa	Other avian	Jacqueline King	Friedrich-Loeffl			Landeslabor	2020-11-06
EPI_ISL_3143	A/common buzzard/Sweden/SVA210505SZ03	A / H5N8	Europe / Swed	Other avian	Siamak Zohari	National Veteri			National Vet	2021-04-04
EPI_ISL_2301	A/common buzzard/Sweden/SVA210323SZ04	A / H5N8	Europe / Swed	Other avian	Siamak Zohari	National Veteri			National Vet	2021-03-14
EPI_ISL_1391	A/northern goshawk/Sweden/SVA210318SZ03	A / H5N5	Europe / Swed	Other avian	Siamak Zohari	National Veteri			National Vet	2021-02-19
EPI_ISL_1080	A/common coot/Czech Republic/3160-2/2021	A / H5N8	Europe / Czech	Other avian	Alexander Nag	State Veterinar	Nagy,A; Cern		State Veterir	2021-02-14
EPI_ISL_1439	A/Fox/Netherlands/EMC4/2022	A / H5N1	Europe / Nethe	mammals	Sanne Thewes	Erasmus Medic			Erasmus Me	2022-04-02
EPI_ISL_2194	A/red fox/Netherlands/21028774-004/2021	A / H5N1	Europe / Nethe	mammals	Rene Heutink ('	Wageningen Bi	Beerens, Nar		Wageningen	2021-05-17
EPI_ISL_1481	A/porpoise /Sweden/SVA220712SZ0367/FB00	A / H5N1	Europe / Swed	Other mam	Siamak Zohari	National Veteri		Elina	National Vet	2022-06-29
EPI_ISL_1206	A/Fox/Netherlands/EMC1/2022	A / H5N1	Europe / Nethe	Other mam	Oanh Vuong-Z	Erasmus Medic	R.A.M., Foucl		Erasmus Me	2022-01-26
EPI_ISL_2194	A/red fox/Netherlands/21028774-002/2021	A / H5N1	Europe / Nethe	Other mam	Rene Heutink ('	Wageningen Bi	Beerens, Nar		Wageningen	2021-05-17
EPI_ISL_1508	A/Mallard/Netherlands/6/2022	A / H5N1	Europe / Nethe	Avian	Sanne Thewes	Erasmus Medic			Erasmus Me	2022-08-24
EPI_ISL_1508	A/Mallard/Netherlands/3/2022	A / H5N1	Europe / Nethe	Avian	Sanne Thewes	Erasmus Medic			Erasmus Me	2022-08-18
EPI_ISL_1508	A/Common Teal/Netherlands/1/2022	A / H5N1	Europe / Nethe	Avian	Sanne Thewes	Erasmus Medic			Erasmus Me	2022-08-29
EPI_ISL_1508	A/European Herring Gull/Netherlands/15/2022	A / H5N1	Europe / Nethe	Avian	Sanne Thewes	Erasmus Medic			Erasmus Me	2022-08-29
EPI_ISL_1508	A/Lesser Black-backed Gull/Netherlands/3/202	A / H5N1	Europe / Nethe	Avian	Sanne Thewes	Erasmus Medic			Erasmus Me	2022-08-29
EPI_ISL_1508	A/Greylag Goose/Netherlands/13/2022	A / H5N1	Europe / Nethe	Avian	Sanne Thewes	Erasmus Medic			Erasmus Me	2022-08-27
EPI_ISL_1508	A/Greylag Goose/Netherlands/11/2022	A / H5N1	Europe / Nethe	Avian	Sanne Thewes	Erasmus Medic			Erasmus Me	2022-08-26
EPI_ISL_1508	A/Greylag Goose/Netherlands/10/2022	A / H5N1	Europe / Nethe	Avian	Sanne Thewes	Erasmus Medic			Erasmus Me	2022-08-26
EPI_ISL_1508	A/Northern Gannet/Netherlands/1/2022	A / H5N1	Europe / Nethe	Avian	Sanne Thewes	Erasmus Medic			Erasmus Me	2022-08-23
EPI_ISL_1508	A/European Herring Gull/Netherlands/14/2022	A / H5N1	Europe / Nethe	Avian	Sanne Thewes	Erasmus Medic			Erasmus Me	2022-08-23
EPI_ISL_1508	A/European Herring Gull/Netherlands/13/2022	A / H5N1	Europe / Nethe	Avian	Sanne Thewes	Erasmus Medic			Erasmus Me	2022-08-19
EPI_ISL_1508	A/Common Buzzard/Netherlands/1/2022	A / H5N1	Europe / Nethe	Avian	Sanne Thewes	Erasmus Medic			Erasmus Me	2022-08-17
EPI_ISL_1507	A/crow/Hokkaido/HU-1/2022	A / H5N1	Asia / Japan / H	Avian	Norikazu Isoda	Graduate Scho	Yoshihiro, SA		Graduate Sc	2022-05-18
EPI_ISL_1507	A/emu/Hokkaido/A-2/2022	A / H5N1	Asia / Japan / H	Avian	Norikazu Isoda	Graduate Scho	Yoshihiro, SA		Graduate Sc	2022-04-16
EPI_ISL_14761	A/avian/Italy/21VIR11773-4/2021	A / H5N1	Europe / Italy	Avian	Giacomo Barbi	Istituto Zooprof	Barbierato, G		Istituto Zoop	2021-12-28
EPI_ISL_1476	A/avian/Italy/21VIR11301/2021	A / H5N1	Europe / Italy	Avian	Giacomo Barbi	Istituto Zooprof	Barbierato, G		Istituto Zoop	2021-12-15

EPI_ISL_1476(	A/avian/Italy/21VIR11236/2021	A / H5N1	Europe / Italy	Avian	Giacomo Barbi	Istituto Zooprof	Barbierato, G		Istituto Zoopr	2021-12-14
EPI_ISL_1476(	A/avian/Italy/21VIR10913-3/2021	A / H5N1	Europe / Italy	Avian	Giacomo Barbi	Istituto Zooprof	Barbierato, G		Istituto Zoopr	2021-12-09
EPI_ISL_1476(	A/avian/Italy/21VIR9425-2/2021	A / H5N1	Europe / Italy	Avian	Giacomo Barbi	Istituto Zooprof	Barbierato, G		Istituto Zoopr	2021-11-08
EPI_ISL_14171	A/Eurasian Spoonbill/Netherlands/1B/2022	A / H5N1	Europe / Nethe	Avian	Sanne Thewes	Erasmus Medic			Erasmus Me	2022-07-11
EPI_ISL_14171	A/Eurasian Spoonbill/Netherlands/1A/2022	A / H5N1	Europe / Nethe	Avian	Sanne Thewes	Erasmus Medic			Erasmus Me	2022-07-11
EPI_ISL_14163	A/Greylag Goose/Netherlands/9/2022	A / H5N1	Europe / Nethe	Avian	Sanne Thewes	Erasmus Medic			Erasmus Me	2022-07-12
EPI_ISL_14163	A/European Herring Gull/Netherlands/9/2022	A / H5N1	Europe / Nethe	Avian	Sanne Thewes	Erasmus Medic			Erasmus Me	2022-07-04
EPI_ISL_14163	A/Caspian Gull/Netherlands/5/2022	A / H5N1	Europe / Nethe	Avian	Sanne Thewes	Erasmus Medic			Erasmus Me	2022-07-08
EPI_ISL_14163	A/European Herring Gull/Netherlands/8/2022	A / H5N1	Europe / Nethe	Avian	Sanne Thewes	Erasmus Medic			Erasmus Me	2022-07-07
EPI_ISL_14163	A/Common Tern/Netherlands/10/2022	A / H5N1	Europe / Nethe	Avian	Sanne Thewes	Erasmus Medic			Erasmus Me	2022-07-07
EPI_ISL_14065	A/white-tailed eagle/Hokkaido/20220210001/2(	A / H5N1	Asia / Japan / H	Avian	Norikazu Isoda	Graduate Scho	Yoshihiro, Sa		Graduate Sc	2022-02-10
EPI_ISL_14064	A/crow/Hokkaido/0102M086/2022	A / H5N1	Asia / Japan / H	Avian	Norikazu Isoda	Graduate Scho	Yoshihiro, Sa		Graduate Sc	2022-02-09
EPI_ISL_14064	A/crow/Hokkaido/0102L010/2022	A / H5N1	Asia / Japan / H	Avian	Norikazu Isoda	Graduate Scho	Yoshihiro, Sa		Graduate Sc	2022-02-14
EPI_ISL_14064	A/crow/Hokkaido/0103B073/2022 (H5N1)	A / H5N1	Asia / Japan / H	Avian	Norikazu Isoda	Graduate Scho	Yoshihiro, Sa		Graduate Sc	2022-04-01
EPI_ISL_14064	A/crow/Hokkaido/0102F043/2022	A / H5N1	Asia / Japan / H	Avian	Norikazu Isoda	Graduate Scho	Yoshihiro, Sa		Graduate Sc	2022-02-07
EPI_ISL_14064	A/crow/Hokkaido/0102F048/2022	A / H5N1	Asia / Japan / H	Avian	Norikazu Isoda	Graduate Scho	Yoshihiro, Sa		Graduate Sc	2022-02-08
EPI_ISL_14064	A/crow/Hokkaido/0102F046/2022	A / H5N1	Asia / Japan / H	Avian	Norikazu Isoda	Graduate Scho	Yoshihiro, Sa		Graduate Sc	2022-02-07
EPI_ISL_14064	A/crow/Hokkaido/0101Q054/2022	A / H5N1	Asia / Japan / H	Avian	Norikazu Isoda	Graduate Scho	Yoshihiro, Sa		Graduate Sc	2022-02-03
EPI_ISL_14064	A/crow/Hokkaido/0101Q044/2022 (H5N1)	A / H5N1	Asia / Japan / H	Avian	Norikazu Isoda	Graduate Scho	Yoshihiro, Sa		Graduate Sc	2022-01-20
EPI_ISL_13778	A/Common Tern/Netherlands/7/2022	A / H5N1	Europe / Nethe	Avian	Sanne Thewes	Erasmus Medic			Erasmus Me	2022-06-20
EPI_ISL_13778	A/Common Tern/Netherlands/6/2022	A / H5N1	Europe / Nethe	Avian	Sanne Thewes	Erasmus Medic			Erasmus Me	2022-06-20
EPI_ISL_13778	A/Common Tern/Netherlands/5/2022	A / H5N1	Europe / Nethe	Avian	Sanne Thewes	Erasmus Medic			Erasmus Me	2022-06-20
EPI_ISL_13778	A/Common Tern/Netherlands/4/2022	A / H5N1	Europe / Nethe	Avian	Sanne Thewes	Erasmus Medic			Erasmus Me	2022-06-20
EPI_ISL_13778	A/Sandwich Tern/Netherlands/15/2022	A / H5N1	Europe / Nethe	Avian	Sanne Thewes	Erasmus Medic			Erasmus Me	2022-06-24
EPI_ISL_13778	A/Common Tern/Netherlands/9/2022	A / H5N1	Europe / Nethe	Avian	Sanne Thewes	Erasmus Medic			Erasmus Me	2022-06-24
EPI_ISL_13778	A/Sandwich Tern/Netherlands/14/2022	A / H5N1	Europe / Nethe	Avian	Sanne Thewes	Erasmus Medic			Erasmus Me	2022-06-24
EPI_ISL_13778	A/Common Tern/Netherlands/8/2022	A / H5N1	Europe / Nethe	Avian	Sanne Thewes	Erasmus Medic			Erasmus Me	2022-06-16
EPI_ISL_13778	A/Sandwich Tern/Netherlands/13/2022	A / H5N1	Europe / Nethe	Avian	Sanne Thewes	Erasmus Medic			Erasmus Me	2022-06-07
EPI_ISL_13778	A/Sandwich Tern/Netherlands/12/2022	A / H5N1	Europe / Nethe	Avian	Sanne Thewes	Erasmus Medic			Erasmus Me	2022-06-07
EPI_ISL_13778	A/European Herring Gull/Netherlands/7/2022	A / H5N1	Europe / Nethe	Avian	Sanne Thewes	Erasmus Medic			Erasmus Me	2022-06-07
EPI_ISL_13778	A/European Herring Gull/Netherlands/6/2022	A / H5N1	Europe / Nethe	Avian	Sanne Thewes	Erasmus Medic			Erasmus Me	2022-06-07
EPI_ISL_13778	A/European Herring Gull/Netherlands/5/2022	A / H5N1	Europe / Nethe	Avian	Sanne Thewes	Erasmus Medic			Erasmus Me	2022-06-07
EPI_ISL_13778	A/Sandwich Tern/Netherlands/11/2022	A / H5N1	Europe / Nethe	Avian	Sanne Thewes	Erasmus Medic			Erasmus Me	2022-06-19
EPI_ISL_13778	A/European Herring Gull/Netherlands/4/2022	A / H5N1	Europe / Nethe	Avian	Sanne Thewes	Erasmus Medic			Erasmus Me	2022-06-19
EPI_ISL_13778	A/Lesser Black-backed Gull/Netherlands/2/202	A / H5N1	Europe / Nethe	Avian	Sanne Thewes	Erasmus Medic			Erasmus Me	2022-06-17
EPI_ISL_13434	A/turkey/Minnesota/22-010464-002-original/20	A / H5N1	North America /	Avian	Mary Lea Killiar	National Veterii	Chinh,Thanh;		National Vet	2022-04-05
EPI_ISL_13434	A/turkey/Minnesota/22-010464-001-original/20	A / H5N1	North America /	Avian	Mary Lea Killiar	National Veterii	Chinh,Thanh;		National Vet	2022-04-05
EPI_ISL_13434	A/turkey/Minnesota/22-010463-002-original/20	A / H5N1	North America /	Avian	Mary Lea Killiar	National Veterii	Chinh,Thanh;		National Vet	2022-04-05
EPI_ISL_13434	A/Cascade duck/Montana/22-010454-003-origi	A / H5N1	North America /	Avian	Mary Lea Killiar	National Veterii	Chinh,Thanh;		National Vet	2022-04-06
EPI_ISL_13434	A/Cascade duck/Montana/22-010454-001-origi	A / H5N1	North America /	Avian	Mary Lea Killiar	National Veterii	Chinh,Thanh;		National Vet	2022-04-06
EPI_ISL_13434	A/turkey/North Carolina/22-010448-003-origina	A / H5N1	North America /	Avian	Mary Lea Killiar	National Veterii	Chinh,Thanh;		National Vet	2022-04-06
EPI_ISL_13434	A/turkey/North Carolina/22-010448-001-origina	A / H5N1	North America /	Avian	Mary Lea Killiar	National Veterii	Chinh,Thanh;		National Vet	2022-04-06

EPI_ISL_13434	A/chicken/Montana/22-010445-003-original/202	A / H5N1	North America /	Avian	Mary Lea Killian	National Veterin	Chinh,Thanh;		National Vete	2022-04-05
EPI_ISL_13434	A/chicken/Montana/22-010445-001-original/202	A / H5N1	North America /	Avian	Mary Lea Killian	National Veterin	Chinh,Thanh;		National Vete	2022-04-05
EPI_ISL_13434	A/chicken/Wyoming/22-010378-001-original/20	A / H5N1	North America /	Avian	Mary Lea Killian	National Veterin	Chinh,Thanh;		National Vete	2022-03-31
EPI_ISL_13433	A/turkey/South Dakota/22-010346-002-original	A / H5N1	North America /	Avian	Mary Lea Killian	National Veterin	Chinh,Thanh;		National Vete	2022-04-04
EPI_ISL_13433	A/turkey/South Dakota/22-010346-001-original	A / H5N1	North America /	Avian	Mary Lea Killian	National Veterin	Chinh,Thanh;		National Vete	2022-04-04
EPI_ISL_13433	A/turkey/Wisconsin/22-010325-003-original/20	A / H5N1	North America /	Avian	Mary Lea Killian	National Veterin	Chinh,Thanh;		National Vete	2022-04-05
EPI_ISL_13433	A/chicken/Wisconsin/22-010325-001-original/2	A / H5N1	North America /	Avian	Mary Lea Killian	National Veterin	Chinh,Thanh;		National Vete	2022-04-05
EPI_ISL_13433	A/domestic duck/New York/22-010321-003-orig	A / H5N1	North America /	Avian	Mary Lea Killian	National Veterin	Chinh,Thanh;		National Vete	2022-04-04
EPI_ISL_13433	A/chicken/New York/22-010321-002-original/20	A / H5N1	North America /	Avian	Mary Lea Killian	National Veterin	Chinh,Thanh;		National Vete	2022-04-04
EPI_ISL_13433	A/chicken/New York/22-010321-001-original/20	A / H5N1	North America /	Avian	Mary Lea Killian	National Veterin	Chinh,Thanh;		National Vete	2022-04-04
EPI_ISL_13433	A/turkey/Minnesota/22-010312-004-original/20	A / H5N1	North America /	Avian	Mary Lea Killian	National Veterin	Chinh,Thanh;		National Vete	2022-04-04
EPI_ISL_13433	A/turkey/Minnesota/22-010312-001-original/20	A / H5N1	North America /	Avian	Mary Lea Killian	National Veterin	Chinh,Thanh;		National Vete	2022-04-04
EPI_ISL_13433	A/chicken/North Carolina/22-010299-002-origir	A / H5N1	North America /	Avian	Mary Lea Killian	National Veterin	Chinh,Thanh;		National Vete	2022-04-04
EPI_ISL_13432	A/chicken/North Carolina/22-010298-002-origir	A / H5N1	North America /	Avian	Mary Lea Killian	National Veterin	Chinh,Thanh;		National Vete	2022-04-04
EPI_ISL_13432	A/chicken/North Carolina/22-010298-001-origir	A / H5N1	North America /	Avian	Mary Lea Killian	National Veterin	Chinh,Thanh;		National Vete	2022-04-04
EPI_ISL_13432	A/turkey/Missouri/22-010296-001-original/2022	A / H5N1	North America /	Avian	Mary Lea Killian	National Veterin	Chinh,Thanh;		National Vete	2022-04-04
EPI_ISL_13286	A/turkey/Minnesota/22-009996-002-original/20	A / H5N1	North America /	Avian	Mary Lea Killian	National Veterin	Chinh,Thanh;		National Vete	2022-04-01
EPI_ISL_13048	A/avian/Burkina_Faso/21VIR11911-3/2021	A / H5N1	Africa / Burkina	Avian	Bianca Zecchin	Istituto Zooprof	LALIDIA-OUC		Laboratoire N	2021-12-07
EPI_ISL_10993	A/Sanderling/Netherlands/1/2022	A / H5N1	Europe / Nethe	Avian	Mark Pronk (Er	Erasmus Medic			Erasmus Me	2022-01-29
EPI_ISL_10993	A/Mallard/Netherlands/8/2021	A / H5N1	Europe / Nethe	Avian	Mark Pronk (Er	Erasmus Medic			Erasmus Me	2021-11-29
EPI_ISL_10993	A/Mallard/Netherlands/7/2021	A / H5N1	Europe / Nethe	Avian	Mark Pronk (Er	Erasmus Medic			Erasmus Me	2021-11-29
EPI_ISL_10993	A/Mallard/Netherlands/6/2021	A / H5N1	Europe / Nethe	Avian	Mark Pronk (Er	Erasmus Medic			Erasmus Me	2021-11-29
EPI_ISL_10993	A/Mallard/Netherlands/5/2021	A / H5N1	Europe / Nethe	Avian	Mark Pronk (Er	Erasmus Medic			Erasmus Me	2021-11-29
EPI_ISL_10993	A/Mallard/Netherlands/4/2021	A / H5N1	Europe / Nethe	Avian	Mark Pronk (Er	Erasmus Medic			Erasmus Me	2021-11-29
EPI_ISL_10993	A/Mallard/Netherlands/2/2022	A / H5N1	Europe / Nethe	Avian	Mark Pronk (Er	Erasmus Medic			Erasmus Me	2022-02-14
EPI_ISL_10992	A/Mallard/Netherlands/1/2022	A / H5N1	Europe / Nethe	Avian	Mark Pronk (Er	Erasmus Medic			Erasmus Me	2022-02-12
EPI_ISL_10943	A/northern goshawk/Sweden/SVA220210SZ03	A / H5N1	Europe / Swede	Avian	Siamak Zohari	National Veterin			National Vete	2022-01-25
EPI_ISL_77482	A/Eurasian wigeon/Germany-SH/AI05952/2021	A / H5N1	Europe / Germa	Avian	Jacqueline King	Friedrich-Loeffle			Landeslabor	2021-10-14
EPI_ISL_77480	A/Eurasian curlew/Germany-SH/AI05960/2021	A / H5N1	Europe / Germa	Avian	Jacqueline King	Friedrich-Loeffle			Landeslabor	2021-10-14
EPI_ISL_70530	A/common buzzard/Sweden/SVA211111SZ037	A / H5N1	Europe / Swede	Avian	Siamak Zohari	National Veterin			National Vete	2021-11-09
EPI_ISL_29326	A/crow/Kazakhstan/15-20-B-Talg-4/2020	A / H5N8	Asia / Kazakhst	Avian	Alexandr Shevt	National Center	Asylulan,Amir		National Vete	2020-09-15
EPI_ISL_41741	A/buzzard/Germany-SN/AI00285/2020	A / H5N8	Europe / Germa	Avian	Jacqueline King	Friedrich-Loeffle			Landesunter	2020-03-20
EPI_ISL_15350	A/chicken/Ghana/AVL-763_21VIR7050-39/202	A / H5N1	Africa / Ghana	Chicken	Giacomo Barbie	Istituto Zooprof	Odoom, T.; A		Veterinary Se	2021-06-08
EPI_ISL_15072	A/chicken/Hokkaido/I-1/2022	A / H5N1	Asia / Japan / H	Chicken	Norikazu Isoda	Graduate Scho	Yoshihiro, SA		Graduate Sc	2022-04-16
EPI_ISL_14769	A/chicken/Lesotho/352.3/2021	A / H5N1	Africa / Lesotho	Chicken			Makalo,M.R.;			2021-06-05
EPI_ISL_14769	A/chicken/Lesotho/341.10/2021	A / H5N1	Africa / Lesotho	Chicken			Makalo,M.R.;			2021-05-29
EPI_ISL_14761	A/chicken/Italy/IZSLT22VIR2562-1/2022	A / H5N1	Europe / Italy	Chicken	Giacomo Barbie	Istituto Zooprof	Barbierato, G		Istituto Zoopr	2022-03-15
EPI_ISL_14761	A/broiler/Italy/22VIR1892-3/2022	A / H5N1	Europe / Italy	Chicken	Giacomo Barbie	Istituto Zooprof	Barbierato, G		Istituto Zoopr	2022-02-23
EPI_ISL_14761	A/chicken/Italy/22VIR1953-4/2022	A / H5N1	Europe / Italy	Chicken	Giacomo Barbie	Istituto Zooprof	Barbierato, G		Istituto Zoopr	2022-02-22
EPI_ISL_14761	A/chicken/Italy/22VIR1953-3/2022	A / H5N1	Europe / Italy	Chicken	Giacomo Barbie	Istituto Zooprof	Barbierato, G		Istituto Zoopr	2022-02-22
EPI_ISL_14761	A/chicken/Italy/22VIR1694-5/2022	A / H5N1	Europe / Italy	Chicken	Giacomo Barbie	Istituto Zooprof	Barbierato, G		Istituto Zoopr	2022-02-18
EPI_ISL_14761	A/broiler/Italy/22VIR278-3/2022	A / H5N1	Europe / Italy	Chicken	Giacomo Barbie	Istituto Zooprof	Barbierato, G		Istituto Zoopr	2022-01-11

EPI_ISL_14761	A/chicken/Italy/22VIR234-1/2022	A / H5N1	Europe / Italy	Chicken	Giacomo Barbi	Istituto Zooprof	Barbierato, G	Istituto Zoop	2022-01-08
EPI_ISL_14761	A/broiler/Italy/22VIR54-1/2022	A / H5N1	Europe / Italy	Chicken	Giacomo Barbi	Istituto Zooprof	Barbierato, G	Istituto Zoop	2022-01-03
EPI_ISL_14761	A/heron/Italy/22VIR1562/2022	A / H5N1	Europe / Italy	Chicken	Giacomo Barbi	Istituto Zooprof	Barbierato, G	Istituto Zoop	2022-01-02
EPI_ISL_14761	A/broiler/Italy/21VIR11886-1/2021	A / H5N1	Europe / Italy	Chicken	Giacomo Barbi	Istituto Zooprof	Barbierato, G	Istituto Zoop	2021-12-29
EPI_ISL_14761	A/broiler/Italy/21VIR11847-1/2021	A / H5N1	Europe / Italy	Chicken	Giacomo Barbi	Istituto Zooprof	Barbierato, G	Istituto Zoop	2021-12-29
EPI_ISL_14761	A/chicken/Italy/21VIR11774-2/2021	A / H5N1	Europe / Italy	Chicken	Giacomo Barbi	Istituto Zooprof	Barbierato, G	Istituto Zoop	2021-12-28
EPI_ISL_14761	A/broiler/Italy/21VIR11802-1/2021	A / H5N1	Europe / Italy	Chicken	Giacomo Barbi	Istituto Zooprof	Barbierato, G	Istituto Zoop	2021-12-27
EPI_ISL_14761	A/broiler/Italy/21VIR11800/2021	A / H5N1	Europe / Italy	Chicken	Giacomo Barbi	Istituto Zooprof	Barbierato, G	Istituto Zoop	2021-12-27
EPI_ISL_14761	A/broiler/Italy/21VIR11583-1/2021	A / H5N1	Europe / Italy	Chicken	Giacomo Barbi	Istituto Zooprof	Barbierato, G	Istituto Zoop	2021-12-22
EPI_ISL_14761	A/chicken/Italy/21VIR11572/2021	A / H5N1	Europe / Italy	Chicken	Giacomo Barbi	Istituto Zooprof	Barbierato, G	Istituto Zoop	2021-12-21
EPI_ISL_14761	A/chicken/Italy/21VIR11571/2021	A / H5N1	Europe / Italy	Chicken	Giacomo Barbi	Istituto Zooprof	Barbierato, G	Istituto Zoop	2021-12-21
EPI_ISL_1476(	A/chicken/Italy/21VIR11508/2021	A / H5N1	Europe / Italy	Chicken	Giacomo Barbi	Istituto Zooprof	Barbierato, G	Istituto Zoop	2021-12-20
EPI_ISL_1476(	A/chicken/Italy/21VIR11485/2021	A / H5N1	Europe / Italy	Chicken	Giacomo Barbi	Istituto Zooprof	Barbierato, G	Istituto Zoop	2021-12-17
EPI_ISL_1476(	A/chicken/Italy/21VIR11385-1/2021	A / H5N1	Europe / Italy	Chicken	Giacomo Barbi	Istituto Zooprof	Barbierato, G	Istituto Zoop	2021-12-16
EPI_ISL_1476(	A/chicken/Italy/21VIR11238/2021	A / H5N1	Europe / Italy	Chicken	Giacomo Barbi	Istituto Zooprof	Barbierato, G	Istituto Zoop	2021-12-14
EPI_ISL_1476(	A/chicken/Italy/21VIR11235/2021	A / H5N1	Europe / Italy	Chicken	Giacomo Barbi	Istituto Zooprof	Barbierato, G	Istituto Zoop	2021-12-14
EPI_ISL_1476(	A/chicken/Italy/21VIR11056-6/2021	A / H5N1	Europe / Italy	Chicken	Giacomo Barbi	Istituto Zooprof	Barbierato, G	Istituto Zoop	2021-12-13
EPI_ISL_1476(	A/chicken/Italy/21VIR11056-1/2021	A / H5N1	Europe / Italy	Chicken	Giacomo Barbi	Istituto Zooprof	Barbierato, G	Istituto Zoop	2021-12-13
EPI_ISL_1476(	A/chicken/Italy/21VIR11054-1/2021	A / H5N1	Europe / Italy	Chicken	Giacomo Barbi	Istituto Zooprof	Barbierato, G	Istituto Zoop	2021-12-13
EPI_ISL_1476(	A/chicken/Italy/21VIR11234/2021	A / H5N1	Europe / Italy	Chicken	Giacomo Barbi	Istituto Zooprof	Barbierato, G	Istituto Zoop	2021-12-13
EPI_ISL_1476(	A/chicken/Italy/21VIR11055-1/2021	A / H5N1	Europe / Italy	Chicken	Giacomo Barbi	Istituto Zooprof	Barbierato, G	Istituto Zoop	2021-12-13
EPI_ISL_1476(	A/chicken/Italy/21VIR10997-1/2021	A / H5N1	Europe / Italy	Chicken	Giacomo Barbi	Istituto Zooprof	Barbierato, G	Istituto Zoop	2021-12-11
EPI_ISL_1476(	A/chicken/Italy/21VIR10999-1/2021	A / H5N1	Europe / Italy	Chicken	Giacomo Barbi	Istituto Zooprof	Barbierato, G	Istituto Zoop	2021-12-11
EPI_ISL_1476(	A/chicken/Italy/21VIR10995-1/2021	A / H5N1	Europe / Italy	Chicken	Giacomo Barbi	Istituto Zooprof	Barbierato, G	Istituto Zoop	2021-12-11
EPI_ISL_1476(	A/chicken/Italy/21VIR11075-2/2021	A / H5N1	Europe / Italy	Chicken	Giacomo Barbi	Istituto Zooprof	Barbierato, G	Istituto Zoop	2021-12-10
EPI_ISL_1476(	A/chicken/Italy/21VIR11073-1/2021	A / H5N1	Europe / Italy	Chicken	Giacomo Barbi	Istituto Zooprof	Barbierato, G	Istituto Zoop	2021-12-10
EPI_ISL_1476(	A/chicken/Italy/21VIR11078-10/2021	A / H5N1	Europe / Italy	Chicken	Giacomo Barbi	Istituto Zooprof	Barbierato, G	Istituto Zoop	2021-12-10
EPI_ISL_1476(	A/chicken/Italy/21VIR11079-11/2021	A / H5N1	Europe / Italy	Chicken	Giacomo Barbi	Istituto Zooprof	Barbierato, G	Istituto Zoop	2021-12-10
EPI_ISL_1476(	A/chicken/Italy/21VIR11076-1/2021	A / H5N1	Europe / Italy	Chicken	Giacomo Barbi	Istituto Zooprof	Barbierato, G	Istituto Zoop	2021-12-10
EPI_ISL_1476(	A/chicken/Italy/21VIR10987-1/2021	A / H5N1	Europe / Italy	Chicken	Giacomo Barbi	Istituto Zooprof	Barbierato, G	Istituto Zoop	2021-12-09
EPI_ISL_1476(	A/chicken/Italy/21VIR11071-2/2021	A / H5N1	Europe / Italy	Chicken	Giacomo Barbi	Istituto Zooprof	Barbierato, G	Istituto Zoop	2021-12-09
EPI_ISL_1476(	A/chicken/Italy/21VIR11074-1/2021	A / H5N1	Europe / Italy	Chicken	Giacomo Barbi	Istituto Zooprof	Barbierato, G	Istituto Zoop	2021-12-09
EPI_ISL_1476(	A/chicken/Italy/21VIR11072-1/2021	A / H5N1	Europe / Italy	Chicken	Giacomo Barbi	Istituto Zooprof	Barbierato, G	Istituto Zoop	2021-12-09
EPI_ISL_1476(	A/chicken/Italy/21VIR10989/2021	A / H5N1	Europe / Italy	Chicken	Giacomo Barbi	Istituto Zooprof	Barbierato, G	Istituto Zoop	2021-12-09
EPI_ISL_1476(	A/chicken/Italy/21VIR10969-3/2021	A / H5N1	Europe / Italy	Chicken	Giacomo Barbi	Istituto Zooprof	Barbierato, G	Istituto Zoop	2021-12-09
EPI_ISL_1476(	A/chicken/Italy/21VIR10972-3/2021	A / H5N1	Europe / Italy	Chicken	Giacomo Barbi	Istituto Zooprof	Barbierato, G	Istituto Zoop	2021-12-09
EPI_ISL_1476(	A/chicken/Italy/21VIR10973-4/2021	A / H5N1	Europe / Italy	Chicken	Giacomo Barbi	Istituto Zooprof	Barbierato, G	Istituto Zoop	2021-12-09
EPI_ISL_1476(	A/chicken/Italy/21VIR10971-3/2021	A / H5N1	Europe / Italy	Chicken	Giacomo Barbi	Istituto Zooprof	Barbierato, G	Istituto Zoop	2021-12-09
EPI_ISL_1476(	A/chicken/Italy/21VIR10843-1/2021	A / H5N1	Europe / Italy	Chicken	Giacomo Barbi	Istituto Zooprof	Barbierato, G	Istituto Zoop	2021-12-08
EPI_ISL_1476(	A/chicken/Italy/21VIR10928/2021	A / H5N1	Europe / Italy	Chicken	Giacomo Barbi	Istituto Zooprof	Barbierato, G	Istituto Zoop	2021-12-07
EPI_ISL_1476(	A/chicken/Italy/21VIR10812-11/2021	A / H5N1	Europe / Italy	Chicken	Giacomo Barbi	Istituto Zooprof	Barbierato, G	Istituto Zoop	2021-12-07
EPI_ISL_1476(	A/chicken/Italy/21VIR10811-28/2021	A / H5N1	Europe / Italy	Chicken	Giacomo Barbi	Istituto Zooprof	Barbierato, G	Istituto Zoop	2021-12-07

EPI_ISL_1476(	A/chicken/Italy/21VIR10808-1/2021	A / H5N1	Europe / Italy	Chicken	Giacomo Barbi	Istituto Zooprof	Barbierato, G	Istituto Zoop	2021-12-07
EPI_ISL_1476(	A/chicken/Italy/21VIR10814-1/2021	A / H5N1	Europe / Italy	Chicken	Giacomo Barbi	Istituto Zooprof	Barbierato, G	Istituto Zoop	2021-12-07
EPI_ISL_1476(	A/chicken/Italy/21VIR10696-7/2021	A / H5N1	Europe / Italy	Chicken	Giacomo Barbi	Istituto Zooprof	Barbierato, G	Istituto Zoop	2021-12-06
EPI_ISL_1476(	A/chicken/Italy/21VIR10853/2021	A / H5N1	Europe / Italy	Chicken	Giacomo Barbi	Istituto Zooprof	Barbierato, G	Istituto Zoop	2021-12-06
EPI_ISL_1476(	A/chicken/Italy/21VIR10637-2/2021	A / H5N1	Europe / Italy	Chicken	Giacomo Barbi	Istituto Zooprof	Barbierato, G	Istituto Zoop	2021-12-04
EPI_ISL_1476(	A/chicken/Italy/21VIR10635-1/2021	A / H5N1	Europe / Italy	Chicken	Giacomo Barbi	Istituto Zooprof	Barbierato, G	Istituto Zoop	2021-12-04
EPI_ISL_1476(	A/chicken/Italy/21VIR10634-1/2021	A / H5N1	Europe / Italy	Chicken	Giacomo Barbi	Istituto Zooprof	Barbierato, G	Istituto Zoop	2021-12-04
EPI_ISL_1476(	A/chicken/Italy/21VIR10723-1/2021	A / H5N1	Europe / Italy	Chicken	Giacomo Barbi	Istituto Zooprof	Barbierato, G	Istituto Zoop	2021-12-03
EPI_ISL_1476(	A/chicken/Italy/21VIR10724-13/2021	A / H5N1	Europe / Italy	Chicken	Giacomo Barbi	Istituto Zooprof	Barbierato, G	Istituto Zoop	2021-12-03
EPI_ISL_1476(	A/chicken/Italy/21VIR10620-1/2021	A / H5N1	Europe / Italy	Chicken	Giacomo Barbi	Istituto Zooprof	Barbierato, G	Istituto Zoop	2021-12-02
EPI_ISL_1476(	A/chicken/Italy/21VIR10619-1/2021	A / H5N1	Europe / Italy	Chicken	Giacomo Barbi	Istituto Zooprof	Barbierato, G	Istituto Zoop	2021-12-02
EPI_ISL_1476(	A/chicken/Italy/21VIR10618-19/2021	A / H5N1	Europe / Italy	Chicken	Giacomo Barbi	Istituto Zooprof	Barbierato, G	Istituto Zoop	2021-12-02
EPI_ISL_1476(	A/chicken/Italy/21VIR10616-1/2021	A / H5N1	Europe / Italy	Chicken	Giacomo Barbi	Istituto Zooprof	Barbierato, G	Istituto Zoop	2021-12-02
EPI_ISL_1476(	A/chicken/Italy/21VIR10522-13/2021	A / H5N1	Europe / Italy	Chicken	Giacomo Barbi	Istituto Zooprof	Barbierato, G	Istituto Zoop	2021-12-01
EPI_ISL_1476(	A/chicken/Italy/21VIR10463-1/2021	A / H5N1	Europe / Italy	Chicken	Giacomo Barbi	Istituto Zooprof	Barbierato, G	Istituto Zoop	2021-12-01
EPI_ISL_1476(	A/chicken/Italy/21VIR10386/2021	A / H5N1	Europe / Italy	Chicken	Giacomo Barbi	Istituto Zooprof	Barbierato, G	Istituto Zoop	2021-11-30
EPI_ISL_1476(	A/chicken/Italy/21VIR10469/2021	A / H5N1	Europe / Italy	Chicken	Giacomo Barbi	Istituto Zooprof	Barbierato, G	Istituto Zoop	2021-11-30
EPI_ISL_1476(	A/chicken/Italy/21VIR10385/2021	A / H5N1	Europe / Italy	Chicken	Giacomo Barbi	Istituto Zooprof	Barbierato, G	Istituto Zoop	2021-11-30
EPI_ISL_1476(	A/chicken/Italy/21VIR10387/2021	A / H5N1	Europe / Italy	Chicken	Giacomo Barbi	Istituto Zooprof	Barbierato, G	Istituto Zoop	2021-11-30
EPI_ISL_1476(	A/chicken/Italy/21VIR10470/2021	A / H5N1	Europe / Italy	Chicken	Giacomo Barbi	Istituto Zooprof	Barbierato, G	Istituto Zoop	2021-11-30
EPI_ISL_1476(	A/chicken/Italy/21VIR10394/2021	A / H5N1	Europe / Italy	Chicken	Giacomo Barbi	Istituto Zooprof	Barbierato, G	Istituto Zoop	2021-11-29
EPI_ISL_1476(	A/chicken/Italy/21VIR10382/2021	A / H5N1	Europe / Italy	Chicken	Giacomo Barbi	Istituto Zooprof	Barbierato, G	Istituto Zoop	2021-11-29
EPI_ISL_1476(	A/chicken/Italy/21VIR10265/2021	A / H5N1	Europe / Italy	Chicken	Giacomo Barbi	Istituto Zooprof	Barbierato, G	Istituto Zoop	2021-11-28
EPI_ISL_1476(	A/chicken/Italy/21VIR10264/2021	A / H5N1	Europe / Italy	Chicken	Giacomo Barbi	Istituto Zooprof	Barbierato, G	Istituto Zoop	2021-11-28
EPI_ISL_1476(	A/chicken/Italy/21VIR10271/2021	A / H5N1	Europe / Italy	Chicken	Giacomo Barbi	Istituto Zooprof	Barbierato, G	Istituto Zoop	2021-11-28
EPI_ISL_1476(	A/chicken/Italy/21VIR10263/2021	A / H5N1	Europe / Italy	Chicken	Giacomo Barbi	Istituto Zooprof	Barbierato, G	Istituto Zoop	2021-11-27
EPI_ISL_1476(	A/chicken/Italy/21VIR10262/2021	A / H5N1	Europe / Italy	Chicken	Giacomo Barbi	Istituto Zooprof	Barbierato, G	Istituto Zoop	2021-11-27
EPI_ISL_1476(	A/chicken/Italy/21VIR10357/2021	A / H5N1	Europe / Italy	Chicken	Giacomo Barbi	Istituto Zooprof	Barbierato, G	Istituto Zoop	2021-11-26
EPI_ISL_1476(	A/chicken/Italy/21VIR10358/2021	A / H5N1	Europe / Italy	Chicken	Giacomo Barbi	Istituto Zooprof	Barbierato, G	Istituto Zoop	2021-11-26
EPI_ISL_1476(	A/chicken/Italy/21VIR10146/2021	A / H5N1	Europe / Italy	Chicken	Giacomo Barbi	Istituto Zooprof	Barbierato, G	Istituto Zoop	2021-11-25
EPI_ISL_1476(	A/chicken/Italy/21VIR10252/2021	A / H5N1	Europe / Italy	Chicken	Giacomo Barbi	Istituto Zooprof	Barbierato, G	Istituto Zoop	2021-11-25
EPI_ISL_1476(	A/chicken/Italy/21VIR10094/2021	A / H5N1	Europe / Italy	Chicken	Giacomo Barbi	Istituto Zooprof	Barbierato, G	Istituto Zoop	2021-11-23
EPI_ISL_1476(	A/chicken/Italy/21VIR10093/2021	A / H5N1	Europe / Italy	Chicken	Giacomo Barbi	Istituto Zooprof	Barbierato, G	Istituto Zoop	2021-11-23
EPI_ISL_1476(	A/chicken/Italy/21VIR10096/2021	A / H5N1	Europe / Italy	Chicken	Giacomo Barbi	Istituto Zooprof	Barbierato, G	Istituto Zoop	2021-11-23
EPI_ISL_1476(	A/chicken/Italy/21VIR9940-5/2021	A / H5N1	Europe / Italy	Chicken	Giacomo Barbi	Istituto Zooprof	Barbierato, G	Istituto Zoop	2021-11-23
EPI_ISL_1476(	A/chicken/Italy/21VIR9944-2/2021	A / H5N1	Europe / Italy	Chicken	Giacomo Barbi	Istituto Zooprof	Barbierato, G	Istituto Zoop	2021-11-23
EPI_ISL_1476(	A/chicken/Italy/21VIR9998/2021	A / H5N1	Europe / Italy	Chicken	Giacomo Barbi	Istituto Zooprof	Barbierato, G	Istituto Zoop	2021-11-22
EPI_ISL_1476(	A/chicken/Italy/21VIR10000/2021	A / H5N1	Europe / Italy	Chicken	Giacomo Barbi	Istituto Zooprof	Barbierato, G	Istituto Zoop	2021-11-22
EPI_ISL_1476(	A/chicken/Italy/21VIR9858-3/2021	A / H5N1	Europe / Italy	Chicken	Giacomo Barbi	Istituto Zooprof	Barbierato, G	Istituto Zoop	2021-11-22
EPI_ISL_1476(	A/chicken/Italy/21VIR9904-18/2021	A / H5N1	Europe / Italy	Chicken	Giacomo Barbi	Istituto Zooprof	Barbierato, G	Istituto Zoop	2021-11-19
EPI_ISL_1476(	A/chicken/Italy/21VIR9904-17/2021	A / H5N1	Europe / Italy	Chicken	Giacomo Barbi	Istituto Zooprof	Barbierato, G	Istituto Zoop	2021-11-19
EPI_ISL_1476(	A/chicken/Italy/21VIR9904-16/2021	A / H5N1	Europe / Italy	Chicken	Giacomo Barbi	Istituto Zooprof	Barbierato, G	Istituto Zoop	2021-11-19

EPI_ISL_14760	A/chicken/Italy/21VIR9830-27/2021	A / H5N1	Europe / Italy	Chicken	Giacomo Barbi	Istituto Zooprof	Barbierato, G	Istituto Zoopr	2021-11-19
EPI_ISL_14760	A/chicken/Italy/21VIR9902-2/2021	A / H5N1	Europe / Italy	Chicken	Giacomo Barbi	Istituto Zooprof	Barbierato, G	Istituto Zoopr	2021-11-18
EPI_ISL_14760	A/chicken/Italy/21VIR9868-8/2021	A / H5N1	Europe / Italy	Chicken	Giacomo Barbi	Istituto Zooprof	Barbierato, G	Istituto Zoopr	2021-11-17
EPI_ISL_14760	A/chicken/Italy/21VIR9580-23/2021	A / H5N1	Europe / Italy	Chicken	Giacomo Barbi	Istituto Zooprof	Barbierato, G	Istituto Zoopr	2021-11-15
EPI_ISL_14760	A/chicken/Italy/21VIR9508-3/2021	A / H5N1	Europe / Italy	Chicken	Giacomo Barbi	Istituto Zooprof	Barbierato, G	Istituto Zoopr	2021-11-11
EPI_ISL_14760	A/chicken/Italy/21VIR9507-7/2021	A / H5N1	Europe / Italy	Chicken	Giacomo Barbi	Istituto Zooprof	Barbierato, G	Istituto Zoopr	2021-11-11
EPI_ISL_14760	A/chicken/Italy/21VIR9509-1/2021	A / H5N1	Europe / Italy	Chicken	Giacomo Barbi	Istituto Zooprof	Barbierato, G	Istituto Zoopr	2021-11-11
EPI_ISL_14760	A/chicken/Italy/21VIR9371-1/2021	A / H5N1	Europe / Italy	Chicken	Giacomo Barbi	Istituto Zooprof	Barbierato, G	Istituto Zoopr	2021-11-08
EPI_ISL_14760	A/chicken/Italy/21VIR9219-6/2021	A / H5N1	Europe / Italy	Chicken	Giacomo Barbi	Istituto Zooprof	Barbierato, G	Istituto Zoopr	2021-11-07
EPI_ISL_14760	A/chicken/Italy/21VIR9212-1/2021	A / H5N1	Europe / Italy	Chicken	Giacomo Barbi	Istituto Zooprof	Barbierato, G	Istituto Zoopr	2021-11-05
EPI_ISL_14760	A/chicken/Italy/21VIR9133-21/2021	A / H5N1	Europe / Italy	Chicken	Giacomo Barbi	Istituto Zooprof	Barbierato, G	Istituto Zoopr	2021-11-04
EPI_ISL_14760	A/chicken/Italy/21VIR9074-10/2021	A / H5N1	Europe / Italy	Chicken	Giacomo Barbi	Istituto Zooprof	Barbierato, G	Istituto Zoopr	2021-11-02
EPI_ISL_13990	A/chicken/Spain/340-37_22VIR6312-37/2022	A / H5N1	Europe / Spain	Chicken	Giacomo Barbi	Istituto Zooprof	Ruano, M.J.;	Laboratorio	2022-01-31
EPI_ISL_13990	A/chicken/Spain/1096-8_22VIR6312-35/2022	A / H5N1	Europe / Spain	Chicken	Giacomo Barbi	Istituto Zooprof	Ruano, M.J.;	Laboratorio	2022-03-16
EPI_ISL_13990	A/chicken/Spain/942-8_22VIR6312-34/2022	A / H5N1	Europe / Spain	Chicken	Giacomo Barbi	Istituto Zooprof	Ruano, M.J.;	Laboratorio	2022-03-08
EPI_ISL_13990	A/chicken/Spain/924-3_22VIR6312-33/2022	A / H5N1	Europe / Spain	Chicken	Giacomo Barbi	Istituto Zooprof	Ruano, M.J.;	Laboratorio	2022-03-06
EPI_ISL_13990	A/chicken/Spain/899-7_22VIR6312-32/2022	A / H5N1	Europe / Spain	Chicken	Giacomo Barbi	Istituto Zooprof	Ruano, M.J.;	Laboratorio	2022-03-04
EPI_ISL_13990	A/chicken/Spain/897-7_22VIR6312-31/2022	A / H5N1	Europe / Spain	Chicken	Giacomo Barbi	Istituto Zooprof	Ruano, M.J.;	Laboratorio	2022-03-03
EPI_ISL_13957	A/chicken/Mali/T4_180_22VIR6104-7/2022	A / H5N1	Africa / Mali / K	Chicken	Bianca Zecchin	Istituto Zooprof	DIAKITÉ, A.;	Laboratoire	2022-03-16
EPI_ISL_13957	A/chicken/Mali/S3-179_22VIR6104-5/2022	A / H5N1	Africa / Mali / K	Chicken	Bianca Zecchin	Istituto Zooprof	DIAKITÉ, A.;	Laboratoire	2022-03-16
EPI_ISL_13957	A/chicken/Mali/T2-178_22VIR6104-3/2022	A / H5N1	Africa / Mali / K	Chicken	Bianca Zecchin	Istituto Zooprof	DIAKITÉ, A.;	Laboratoire	2022-03-16
EPI_ISL_13957	A/chicken/Mali/T1-177_22VIR6104-1/2022	A / H5N1	Africa / Mali / K	Chicken	Bianca Zecchin	Istituto Zooprof	DIAKITÉ, A.;	Laboratoire	2022-03-16
EPI_ISL_13048	A/chicken/Burkina_Faso/21VIR11911-5/2021	A / H5N1	Africa / Burkina	Chicken	Bianca Zecchin	Istituto Zooprof	LALIDIA-OUC	Laboratoire N	2021-12-16
EPI_ISL_12754	A/laying_hen/Romania/11562_22VIR4106-4/20	A / H5N1	Europe / Roma	Chicken	Bianca Zecchin	Istituto Zooprof	Barbuceanu,	Institute for D	2022-03-26
EPI_ISL_12754	A/laying_hen/Romania/11343_22VIR4106-1/20	A / H5N1	Europe / Roma	Chicken	Bianca Zecchin	Istituto Zooprof	Barbuceanu,	Institute for D	2022-03-18
EPI_ISL_85182	A/Chicken/Hangzhou/E1149/2021	A / H5N6	Asia / China / Z	Chicken	Jun Li (Hangzh	Hangzhou Cen	Li,J;Wang,HQ	Hangzhou C	2021-12-23
EPI_ISL_76265	A/chicken/Czech Republic/23404-4K/2021	A / H5N1	Europe / Czech	Chicken	Alexander Nagy	State Veterinar	Alexander,Na	State Veterin	2021-11-25
EPI_ISL_51444	A/chicken/Germany-NW/AI03154/2021	A / H5N8	Europe / Germa	Chicken	Jacqueline King	Friedrich-Loeffle		Chemisches	2021-03-20
EPI_ISL_51443	A/chicken/Germany-NW/AI03148/2021	A / H5N8	Europe / Germa	Chicken	Jacqueline King	Friedrich-Loeffle		Chemisches	2021-03-20
EPI_ISL_51439	A/domestic duck/Germany-ST/AI03183/2021	A / H5N8	Europe / Germa	Chicken	Jacqueline King	Friedrich-Loeffle		Landesamt f	2021-03-21
EPI_ISL_51438	A/chicken/Germany-BW/AI03205/2021	A / H5N8	Europe / Germa	Chicken	Jacqueline King	Friedrich-Loeffle		CVUA Karlsr	2021-03-22
EPI_ISL_51436	A/chicken/Germany-TH/AI03193/2021	A / H5N8	Europe / Germa	Chicken	Jacqueline King	Friedrich-Loeffle		Thüringer La	2021-03-22
EPI_ISL_51435	A/chicken/Germany-SN/AI03502/2021	A / H5N8	Europe / Germa	Chicken	Jacqueline King	Friedrich-Loeffle		Landesunter	2021-03-24
EPI_ISL_51435	A/chicken/Germany-SN/AI03496/2021	A / H5N8	Europe / Germa	Chicken	Jacqueline King	Friedrich-Loeffle		Landesunter	2021-03-24
EPI_ISL_51435	A/chicken/Germany-BY/AI03544/2021	A / H5N8	Europe / Germa	Chicken	Jacqueline King	Friedrich-Loeffle		Bayrisches L	2021-03-24
EPI_ISL_51435	A/chicken/Germany-TH/AI03513/2021	A / H5N8	Europe / Germa	Chicken	Jacqueline King	Friedrich-Loeffle		Thüringer La	2021-03-24
EPI_ISL_51435	A/chicken/Germany-NI/AI02412/2021	A / H5N8	Europe / Germa	Chicken	Jacqueline King	Friedrich-Loeffle		Lebensmittel	2021-03-09
EPI_ISL_51428	A/chicken/Germany-NI/AI02543/2021	A / H5N8	Europe / Germa	Chicken	Jacqueline King	Friedrich-Loeffle		Lebensmittel	2021-03-10
EPI_ISL_51350	A/chicken/Germany-NI/AI02942/2021	A / H5N8	Europe / Germa	Chicken	Jacqueline King	Friedrich-Loeffle		Lebensmittel	2021-03-15
EPI_ISL_51025	A/chicken/Germany-MV/AI01407/2021	A / H5N8	Europe / Germa	Chicken	Jacqueline King	Friedrich-Loeffle		Landesamt f	2021-02-12
EPI_ISL_51021	A/chicken/Germany-BY/AI02066/2021	A / H5N8	Europe / Germa	Chicken	Jacqueline King	Friedrich-Loeffle		Bayrisches L	2021-03-02
EPI_ISL_51021	A/chicken/Germany-BY/AI02064/2021	A / H5N8	Europe / Germa	Chicken	Jacqueline King	Friedrich-Loeffle		Bayrisches L	2021-03-01

EPI_ISL_51021	A/chicken/Germany-NW/AI02054/2021	A / H5N8	Europe / Germa	Chicken	Jacqueline King	Friedrich-Loeffl		Chemisches	2021-03-01
EPI_ISL_50616	A/chicken/Germany-MV/AI02431/2020	A / H5N5	Europe / Germa	Chicken	Jacqueline King	Friedrich-Loeffl		Landesamt f	2020-11-09
EPI_ISL_40614	A/chicken/Nigeria/VRD21-98_21VIR2288-6/20	A / H5N1	Africa / Nigeria	Chicken			Shittu,I.; Meso		2021-02-12
EPI_ISL_40614	A/chicken/Nigeria/VRD21-88_21VIR2288-8/20	A / H5N8	Africa / Nigeria	Chicken			Shittu,I.; Meso		2021-02-25
EPI_ISL_40614	A/chicken/Nigeria/VRD21-109_21VIR2370-425	A / H5N1	Africa / Nigeria	Chicken			Shittu,I.; Meso		2021-03-01
EPI_ISL_40614	A/chicken/Nigeria/VRD21-102_21VIR2370-424	A / H5N1	Africa / Nigeria	Chicken			Shittu,I.; Meso		2021-03-01
EPI_ISL_40614	A/chicken/Nigeria/VRD21-100_21VIR2370-423	A / H5N8	Africa / Nigeria	Chicken			Shittu,I.; Meso		2021-03-01
EPI_ISL_40614	A/chicken/Nigeria/VRD21-035B_21VIR2288-1/	A / H5N8	Africa / Nigeria	Chicken			Shittu,I.; Meso		2021-02-05
EPI_ISL_40614	A/chicken/Nigeria/VRD21-43_21VIR2288-4/20	A / H5N8	Africa / Nigeria	Chicken			Shittu,I.; Meso		2021-02-10
EPI_ISL_31285	A/chicken/Albania/3816_21VIR5387-2/2021	A / H5N8	Europe / Albani	Chicken	Bianca Zecchin	Istituto Zooprof	Boci, J.; Shko	Istituto Zoop	2021-06-04
EPI_ISL_31285	A/chicken/Albania/2574_21VIR5387-1/2021	A / H5N8	Europe / Albani	Chicken	Bianca Zecchin	Istituto Zooprof	Boci, J.; Shko	Istituto Zoop	2021-05-31
EPI_ISL_31285	A/chicken/Kosovo/104_21VIR5162-7/2021	A / H5N8	Europe / Kosov	Chicken	Bianca Zecchin	Istituto Zooprof	Uka, K.; Cana	Istituto Zoop	2021-06-01
EPI_ISL_31285	A/chicken/Kosovo/90_21VIR5162-4/2021	A / H5N8	Europe / Kosov	Chicken	Bianca Zecchin	Istituto Zooprof	Uka, K.; Cana	Istituto Zoop	2021-05-25
EPI_ISL_31285	A/chicken/Kosovo/84_21VIR5162-2/2021	A / H5N8	Europe / Kosov	Chicken	Bianca Zecchin	Istituto Zooprof	Uka, K.; Cana	Istituto Zoop	2021-05-25
EPI_ISL_31285	A/chicken/Kosovo/82_21VIR5162-1/2021	A / H5N8	Europe / Kosov	Chicken	Bianca Zecchin	Istituto Zooprof	Uka, K.; Cana	Istituto Zoop	2021-05-19
EPI_ISL_31285	A/chicken/Kosovo/113_21VIR5162-19/2021	A / H5N8	Europe / Kosov	Chicken	Bianca Zecchin	Istituto Zooprof	Uka, K.; Cana	Istituto Zoop	2021-06-03
EPI_ISL_31285	A/chicken/Kosovo/98_21VIR5162-16/2021	A / H5N8	Europe / Kosov	Chicken	Bianca Zecchin	Istituto Zooprof	Uka, K.; Cana	Istituto Zoop	2021-05-31
EPI_ISL_31285	A/chicken/Kosovo/97_21VIR5162-15/2021	A / H5N8	Europe / Kosov	Chicken	Bianca Zecchin	Istituto Zooprof	Uka, K.; Cana	Istituto Zoop	2021-05-31
EPI_ISL_22760	A/chicken/Senegal/21VIR1084-5/2021	A / H5N1	Africa / Senega	Chicken	Bianca Zecchin	Istituto Zooprof	Lo, F.T.; Diall	Istituto Zoop	2020-12-23
EPI_ISL_22760	A/chicken/Senegal/21VIR1084-4/2021	A / H5N1	Africa / Senega	Chicken	Bianca Zecchin	Istituto Zooprof	Lo, F.T.; Diall	Istituto Zoop	2020-12-23
EPI_ISL_22760	A/chicken/Senegal/21VIR1084-3/2021	A / H5N1	Africa / Senega	Chicken	Bianca Zecchin	Istituto Zooprof	Lo, F.T.; Diall	Istituto Zoop	2020-12-23
EPI_ISL_17199	A/chicken/Bulgaria/50-1_21VIR1454-9/2021	A / H5N8	Europe / Bulga	Chicken	Bianca Zecchin	Istituto Zooprof	Goujgoulova,	Istituto Zoop	2021-02-08
EPI_ISL_17199	A/chicken/Bulgaria/39_21VIR1454-3/2021	A / H5N8	Europe / Bulga	Chicken	Bianca Zecchin	Istituto Zooprof	Goujgoulova,	Istituto Zoop	2021-02-01
EPI_ISL_81097	A/Chicken/Hungary/18466/2020	A / H5N8	Europe / Hunga	Chicken	Katalin Szentpa	National Food (	Katalin,Szent	National Foo	2020-04-14
EPI_ISL_61018	A/chicken/Netherlands/21038165-006010/2021	A / H5N1	Europe / Nethe	Gallus gallu	Rene Heutink (	Wageningen Bi	Beerens, Nan	Wageningen	2021-11-07
EPI_ISL_21945	A/chicken/Czech Republic/6151-2/2021	A / H5N8	Europe / Czech	Gallus gallu	Alexander Nagy	State Veterinar	Nagy,A;Cerni	State Veterin	2021-03-29
EPI_ISL_13992	A/chicken/Czech Republic/4526/2021	A / H5N8	Europe / Czech	Gallus gallu	Alexander Nagy	State Veterinar	Nagy,A;Cerni	State Veterin	2021-03-06
EPI_ISL_13992	A/chicken/Czech Republic/4756/2021	A / H5N8	Europe / Czech	Gallus gallu	Alexander Nagy	State Veterinar	Nagy,A;Cerni	State Veterin	2021-03-10
EPI_ISL_13992	A/chicken/Czech Republic/4527-1/2021	A / H5N8	Europe / Czech	Gallus gallu	Alexander Nagy	State Veterinar	Nagy,A;Cerni	State Veterin	2021-03-06
EPI_ISL_13992	A/chicken/Czech Republic/4092-2/2021	A / H5N8	Europe / Czech	Gallus gallu	Alexander Nagy	State Veterinar	Nagy,A;Cerni	State Veterin	2021-02-27
EPI_ISL_13992	A/chicken/Czech Republic/4092-1/2021	A / H5N8	Europe / Czech	Gallus gallu	Alexander Nagy	State Veterinar	Nagy,A;Cerni	State Veterin	2021-02-27
EPI_ISL_13992	A/chicken/Czech Republic/3893/2021	A / H5N8	Europe / Czech	Gallus gallu	Alexander Nagy	State Veterinar	Nagy,A;Cerni	State Veterin	2021-02-25
EPI_ISL_13992	A/chicken/Czech Republic/3531-1/2021	A / H5N8	Europe / Czech	Gallus gallu	Alexander Nagy	State Veterinar	Nagy,A;Cerni	State Veterin	2021-02-18
EPI_ISL_10468	A/chicken/Netherlands/21023815-001005/2021	A / H5N8	Europe / Nethe	Gallus gallu	Rene Heutink (	Wageningen Bi	Beerens, Nan	Wageningen	2021-02-19
EPI_ISL_71105	A/chicken/Netherlands/20019879-001005/2020	A / H5N1	Europe / Nethe	Gallus gallu	Rene Heutink (	Wageningen Bi	Beerens, Nan	Wageningen	2020-12-14
EPI_ISL_65392	A/chicken/Netherlands/20018523-001005/2020	A / H5N8	Europe / Nethe	Gallus gallu	Rene Heutink (	Wageningen Bi	Beerens, Nan	Wageningen	2020-11-21
EPI_ISL_65392	A/chicken/Netherlands/20018496-006010/2020	A / H5N8	Europe / Nethe	Gallus gallu	Rene Heutink (	Wageningen Bi	Beerens, Nan	Wageningen	2020-11-20
EPI_ISL_14933	A/duck/France/22P020165/2022	A / H5N1	Europe / Franc	Duck	Francois-Xavie	ANSES Agence		Anses (Plouf	2022-08-24
EPI_ISL_14761	A/duck/Italy/22VIR1295-1/2022	A / H5N1	Europe / Italy	Duck	Giacomo Barbi	Istituto Zooprof	Barbierato, G	Istituto Zoop	2022-02-08
EPI_ISL_14761	A/duck/Italy/22VIR1294-1/2022	A / H5N1	Europe / Italy	Duck	Giacomo Barbi	Istituto Zooprof	Barbierato, G	Istituto Zoop	2022-02-08
EPI_ISL_14760	A/duck/Italy/21VIR11501/2021	A / H5N1	Europe / Italy	Duck	Giacomo Barbi	Istituto Zooprof	Barbierato, G	Istituto Zoop	2021-12-21
EPI_ISL_14760	A/duck/Italy/21VIR10809-1/2021	A / H5N1	Europe / Italy	Duck	Giacomo Barbi	Istituto Zooprof	Barbierato, G	Istituto Zoop	2021-12-07

EPI_ISL_14760	A/duck/Italy/21VIR10523-1/2021	A / H5N1	Europe / Italy	Duck	Giacomo Barbi	Istituto Zooprof	Barbierato, G	Istituto Zoop	2021-12-01
EPI_ISL_14760	A/duck/Italy/21VIR9836-15/2021	A / H5N1	Europe / Italy	Duck	Giacomo Barbi	Istituto Zooprof	Barbierato, G	Istituto Zoop	2021-11-20
EPI_ISL_14760	A/duck/Italy/21VIR9373-2/2021	A / H5N1	Europe / Italy	Duck	Giacomo Barbi	Istituto Zooprof	Barbierato, G	Istituto Zoop	2021-11-09
EPI_ISL_13131	A/duck/Bangladesh/17D1845/2022	A / H5N1	Asia / Banglade	Duck	Mohammad Er	International Ce	Hossain, M.E	International	2022-02-13
EPI_ISL_13131	A/duck/Bangladesh/17D1844/2022	A / H5N1	Asia / Banglade	Duck	Mohammad Er	International Ce	Hossain, M.E	International	2022-02-13
EPI_ISL_13131	A/duck/Bangladesh/17D1843/2022	A / H5N1	Asia / Banglade	Duck	Mohammad Er	International Ce	Hossain, M.E	International	2022-02-13
EPI_ISL_90095	A/duck/Thanh Hoa/4643VTC/2020	A / H5N6	Asia / Vietnam	Duck	Natalia Goncha	State Research		State Resea	2020-09-14
EPI_ISL_90095	A/duck/Thanh Hoa/5331VTC/2020	A / H5N6	Asia / Vietnam	Duck	Natalia Goncha	State Research		State Resea	2020-10-15
EPI_ISL_77481	A/Eurasian wigeon/Germany-SH/AI05950/2021	A / H5N1	Europe / Germa	Duck	Jacqueline King	Friedrich-Loeffl		Landeslabor	2021-10-14
EPI_ISL_73808	A/Duck/Guangdong/21316/2021(H5N8)	A / H5N8	Asia / China / G	Duck	Jiahao Zhang (	South China Ag	Zhang, Jiahao	South China	2021-03
EPI_ISL_73806	A/Duck/Shandong/21232-5/2021(H5N8)	A / H5N8	Asia / China / S	Duck	Jiahao Zhang (	South China Ag	Zhang, Jiahao	South China	2021-03
EPI_ISL_73795	A/Duck/Guangdong/21057/2021(H5N8)	A / H5N8	Asia / China / G	Duck	Jiahao Zhang (	South China Ag	Zhang, Jiahao	South China	2021-01
EPI_ISL_73794	A/Duck/Sichuan/21044-2/2021(H5N8)	A / H5N8	Asia / China / S	Duck	Jiahao Zhang (	South China Ag	Zhang, Jiahao	South China	2021-01
EPI_ISL_67576	A/Duck/Sichuan/21406-8/2021(H5N6)	A / H5N6	Asia / China / S	Duck	Jiahao Zhang (	South China Ag	Zhang, Jiahao	South China	2021-04
EPI_ISL_67576	A/Duck/Guangdong/21314/2021(H5N6)	A / H5N6	Asia / China / G	Duck	Jiahao Zhang (	South China Ag	Zhang, Jiahao	South China	2021-03
EPI_ISL_58654	A/domestic duck/Georgia/DT-22368/2020	A / H5N8	Asia / Georgia	Duck	Nicola Lewis (R	Royal Veterinar	Fouchier, R.A	Erasmus Me	2020-10-02
EPI_ISL_58639	A/garganey/Georgia/DT-22572/2020	A / H5N8	Asia / Georgia	Duck	Nicola Lewis (R	Royal Veterinar	Fouchier, R.A	Erasmus Me	2020-10-12
EPI_ISL_53233	A/duck/Czech Republic/18520-2/2021	A / H5N1	Europe / Czech	Duck	Alexander Nagy	State Veterinar	Alexander,Na	State Veterir	2021-09-27
EPI_ISL_51443	A/domestic duck/Germany-NI/AI03088/2021	A / H5N8	Europe / Germa	Duck	Jacqueline King	Friedrich-Loeffl		Lebensmittel	2021-03-18
EPI_ISL_51020	A/domestic duck/Germany-NW/AI02049/2021	A / H5N8	Europe / Germa	Duck	Jacqueline King	Friedrich-Loeffl		Chemisches	2021-03-01
EPI_ISL_31285	A/duck/Kosovo/107_21VIR5162-17/2021	A / H5N8	Europe / Kosov	Duck	Bianca Zecchin	Istituto Zooprof	Uka, K.; Cana	Istituto Zoop	2021-06-02
EPI_ISL_29326	A/duck/Kazakhstan/12-20-B-Talg-11/2020	A / H5N8	Asia / Kazakhst	Duck	Alexandr Shevt	National Center	Asylulan,Amir	National Vet	2020-09-15
EPI_ISL_22348	A/duck/Romania/10205-t4_21VIR2593-3/2021	A / H5N8	Europe / Roma	Duck	Bianca Zecchin	Istituto Zooprof	Onita, I.; Neic	Istituto Zoop	2021-01-13
EPI_ISL_22348	A/duck/Romania/10202_21VIR2593-31/2021	A / H5N8	Europe / Roma	Duck	Bianca Zecchin	Istituto Zooprof	Onita, I.; Neic	Istituto Zoop	2021-01-13
EPI_ISL_17199	A/pekin_duck/Bulgaria/48-3_21VIR1454-7/202	A / H5N8	Europe / Bulgar	Duck	Bianca Zecchin	Istituto Zooprof	Goujgoulova,	Istituto Zoop	2021-02-04
EPI_ISL_81096	A/Duck/Hungary/18444/2020	A / H5N8	Europe / Hunga	Duck	Katalin Szentpá	National Food (	Katalin,Szent	National Foo	2020-04-14
EPI_ISL_12140	A/duck/Czech Republic/913/2022	A / H5N1	Europe / Czech	Anas platyr	Alexander Nagy	State Veterinar	Alexander,Na	State Veterir	2022-01-12
EPI_ISL_76265	A/duck/Czech Republic/23589-1T/2021	A / H5N1	Europe / Czech	Anas platyr	Alexander Nagy	State Veterinar	Alexander,Na	State Veterir	2021-11-29
EPI_ISL_19414	A/duck/Czech Republic/7681-5/2021	A / H5N8	Europe / Czech	Anas platyr	Alexander Nagy	State Veterinar	Nagy,A;Cerni	State Veterir	2021-04-18
EPI_ISL_19414	A/duck/Czech Republic/7681-7/2021	A / H5N8	Europe / Czech	Anas platyr	Alexander Nagy	State Veterinar	Nagy,A;Cerni	State Veterir	2021-04-18
EPI_ISL_19414	A/duck/Czech Republic/7681-8/2021	A / H5N8	Europe / Czech	Anas platyr	Alexander Nagy	State Veterinar	Nagy,A;Cerni	State Veterir	2021-04-18
EPI_ISL_19413	A/duck/Czech Republic/7682-2/2021	A / H5N8	Europe / Czech	Anas platyr	Alexander Nagy	State Veterinar	Nagy,A;Cerni	State Veterir	2021-04-18
EPI_ISL_19413	A/duck/Czech Republic/7682-5/2021	A / H5N8	Europe / Czech	Anas platyr	Alexander Nagy	State Veterinar	Nagy,A;Cerni	State Veterir	2021-04-18
EPI_ISL_19413	A/duck/Czech Republic/7682-9/2021	A / H5N8	Europe / Czech	Anas platyr	Alexander Nagy	State Veterinar	Nagy,A;Cerni	State Veterir	2021-04-18
EPI_ISL_14884	A/duck/Bangladesh/50002/2021	A / H5N1	Asia / Banglade	Anas sp.			Barman,S.; T		2021-09-18
EPI_ISL_14884	A/duck/Bangladesh/50000/2021	A / H5N1	Asia / Banglade	Anas sp.			Barman,S.; T		2021-09-18
EPI_ISL_14884	A/duck/Bangladesh/50186/2021	A / H5N1	Asia / Banglade	Anas sp.			Barman,S.; T		2021-10-16
EPI_ISL_91172	A/northern goshawk/Netherlands/22000305-00	A / H5N1	Europe / Nethe	Accipiter ge	Rene Heutink ('	Wageningen Bi	Beerens, Nar	Wageningen	2022-01-04
EPI_ISL_14391	A/Buteo_buteo/Belgium/2606_0006/2022	A / H5N1	Europe / Belgiu	Buteo buteo	Steven Van Bo	Sciensano, Dep	Van Borm, St	Sciensano -	2022-02-20
EPI_ISL_10482	A/common buzzard/Netherlands/21021396-002	A / H5N1	Europe / Nethe	Buteo buteo	Rene Heutink ('	Wageningen Bi	Beerens, Nar	Wageningen	2021-01-09
EPI_ISL_10482	A/common buzzard/Netherlands/21021023-002	A / H5N3	Europe / Nethe	Buteo buteo	Rene Heutink ('	Wageningen Bi	Beerens, Nar	Wageningen	2021-01-04
EPI_ISL_61439	A/buzzard/Germany-MV/AI02166/2020	A / H5N5	Europe / Germa	Buteo buteo	Jacqueline King	Friedrich-Loeffl		Landesamt f	2020-10-29

EPI_ISL_22272	A/western marsh harrier/Netherlands/21028600	A / H5N1	Europe / Nethe	Circus aeru	Rene Heutink (\	Wageningen Bi	Beerens, Nar	Wageningen	2021-05-07
EPI_ISL_51024	A/white-tailed eagle/Germany-SH/AI02170/202	A / H5N8	Europe / Germa	Halietus alk	Jacqueline King	Friedrich-Loeffle		Landeslabor	2020-10-29
EPI_ISL_31359	A/eagle/Hungary/8569/2021 (H5N5)	A / H5N5	Europe / Hunga	Halietus alk	Katalin Szentpá	National Food (	Katalin,Szent	National Foo	2021-03-08
EPI_ISL_21940	A/white-tailed eagle/Netherlands/21027616-00	A / H5N1	Europe / Nethe	Halietus alk	Rene Heutink (\	Wageningen Bi	Beerens, Nar	Wageningen	2021-04-23
EPI_ISL_31285	A/Canada goose/Sweden/SVA210407SZ0510/	A / H5N8	Europe / Swede	Branta cana	Siamak Zohari	National Veterii		National Vete	2021-04-06
EPI_ISL_31281	A/Canada goose/Sweden/SVA210331SZ/FB00	A / H5N8	Europe / Swede	Branta cana	Siamak Zohari	National Veterii		National Vete	2021-03-25
EPI_ISL_14778	A/Wild bird/China/Cixi02/2020	A / H5N8	Asia / China	Wild bird			Miao,X.		2020-11-25
EPI_ISL_14702	A/nothern_gannet/France/22P019331/2022	A / H5N1	Europe / France	Wild bird	Francois-Xavier	ANSES Agence		Anses (Plouf	2022-07-13
EPI_ISL_12754	A/sea_eagle/Norway/2022-07-198_22VIR3866	A / H5N5	Europe / Norwa	Wild bird	Bianca Zecchin	Istituto Zooprof	Gjerset, B.; M	Norwegian V	2022-03-24
EPI_ISL_12754	A/sea_eagle/Norway/2022-07-196_22VIR3866	A / H5N5	Europe / Norwa	Wild bird	Bianca Zecchin	Istituto Zooprof	Gjerset, B.; M	Norwegian V	2022-03-25
EPI_ISL_12754	A/pelican/Romania/11334_22VIR4106-3/2022	A / H5N1	Europe / Roma	Wild bird	Bianca Zecchin	Istituto Zooprof	Barbuceanu,	Institute for I	2022-03-17
EPI_ISL_12754	A/pelican/Romania/11335_22VIR4106-2/2022	A / H5N1	Europe / Roma	Wild bird	Bianca Zecchin	Istituto Zooprof	Barbuceanu,	Institute for I	2022-03-17
EPI_ISL_31285	A/partridge/Kosovo/96_21VIR5162-14/2021	A / H5N8	Europe / Kosov	Wild bird	Bianca Zecchin	Istituto Zooprof	Uka, K.; Cana	Istituto Zoopr	2021-05-31
EPI_ISL_31285	A/dalmatian pelican/Greece/47t_21VIR3735-3/	A / H5N8	Europe / Greec	Wild bird	Bianca Zecchin	Istituto Zooprof	Georgiades, (	Istituto Zoopr	2021-03-22
EPI_ISL_22760	A/great-white_pelican/Senegal/21-67_21VIR10	A / H5N1	Africa / Senega	Wild bird	Bianca Zecchin	Istituto Zooprof	Lo, F.T.; Diall	Istituto Zoopr	2021-01-23
EPI_ISL_22348	A/peregrine_falcon/Northern_Ireland/21VIR166	A / H5N3	Europe / Unitec	Wild bird	Bianca Zecchin	Istituto Zooprof	McMenamy, M	Istituto Zoopr	2021-01-25
EPI_ISL_22348	A/hawk/Romania/10363_21VIR2593-11/2021	A / H5N8	Europe / Roma	Wild bird	Bianca Zecchin	Istituto Zooprof	Onita, I.; Neic	Istituto Zoopr	2021-01-22
EPI_ISL_22348	A/peregrine_falcon/Spain/3365-1_21VIR1230-1	A / H5N8	Europe / Spain	Wild bird	Bianca Zecchin	Istituto Zooprof	Ruano Ramo	Istituto Zoopr	2020-11-20
EPI_ISL_22348	A/ciconia_ciconia/Spain/102-1_21VIR1230-2/2	A / H5N8	Europe / Spain	Wild bird	Bianca Zecchin	Istituto Zooprof	Ruano Ramo	Istituto Zoopr	2021-01-07
EPI_ISL_22348	A/anser_anser/Spain/297-1_21VIR1230-5/202	A / H5N8	Europe / Spain	Wild bird	Bianca Zecchin	Istituto Zooprof	Ruano Ramo	Istituto Zoopr	2021-01-27
EPI_ISL_22348	A/anser_anser/Spain/102-8_21VIR1230-4/202	A / H5N8	Europe / Spain	Wild bird	Bianca Zecchin	Istituto Zooprof	Ruano Ramo	Istituto Zoopr	2021-01-07
EPI_ISL_22348	A/seagull/Italy/21VIR2479/2021	A / H5N8	Europe / Italy	Wild bird	Bianca Zecchin	Istituto Zooprof	Zecchin, B.; F	Istituto Zoopr	2021-01-28
EPI_ISL_22348	A/yellow-legged_gull/Switzerland/15-0039_21V	A / H5N4	Europe / Switze	Wild bird	Bianca Zecchin	Istituto Zooprof	Albini, S.; H?s	Istituto Zoopr	2021-02-01
EPI_ISL_22348	A/knot_wader/Ireland/473_21VIR2956-2/2021	A / H5N3	Europe / Ireland	Wild bird	Bianca Zecchin	Istituto Zooprof	Garza Cuarte	Istituto Zoopr	2021-01-01
EPI_ISL_22348	A/knot_wader/Ireland/472_21VIR2956-1/2021	A / H5N3	Europe / Ireland	Wild bird	Bianca Zecchin	Istituto Zooprof	Garza Cuarte	Istituto Zoopr	2021-01-01
EPI_ISL_19396	A/eurasian eagle-owl/Henan/SMQ11/2020	A / H5N8	Asia / China / H	Wild bird	Hongliang Chai	Northeast Fores		College of W	2020-11-11
EPI_ISL_14841	A/mallard/22P019377/France/2022	A / H5N1	Europe / France	Mallard	Francois-Xavier	ANSES Agence		Anses (Plouf	2022-07-06
EPI_ISL_58636	A/mallard/Georgia/DT-20222/2020	A / H5N8	Asia / Georgia	Mallard	Nicola Lewis (R	Royal Veterinar	Fouchier, R.A	Erasmus Me	2020-12-29
EPI_ISL_58636	A/mallard/Georgia/DT-22862/2020	A / H5N8	Asia / Georgia	Mallard	Nicola Lewis (R	Royal Veterinar	Fouchier, R.A	Erasmus Me	2020-10-20
EPI_ISL_65495	A/mallard/Italy/20VIR7139-73/2020	A / H5N8	Europe / Italy /	Mallard	Bianca Zecchin	Istituto Zooprof	Zecchin, B.; F	Istituto Zoopr	2020-11-14
EPI_ISL_50613	A/greylag goose/Germany-SH/AI02207/2020	A / H5N8	Europe / Germa	Greylag goo	Jacqueline King	Friedrich-Loeffle		Landeslabor	2020-11-03
EPI_ISL_71105	A/greylag goose/Netherlands/20019685-002/20	A / H5N1	Europe / Nethe	Greylag goo	Rene Heutink (\	Wageningen Bi	Beerens, Nar	Wageningen	2020-12-08
EPI_ISL_65391	A/greylag goose/Netherlands/20016494-001/20	A / H5N8	Europe / Nethe	Greylag goo	Rene Heutink (\	Wageningen Bi	Beerens, Nar	Wageningen	2020-10-27
EPI_ISL_65391	A/greylag goose/Netherlands/20016414-001/20	A / H5N8	Europe / Nethe	Greylag goo	Rene Heutink (\	Wageningen Bi	Beerens, Nar	Wageningen	2020-10-26
EPI_ISL_29326	A/pigeon/Kazakhstan/15-20-B-Talg-5/2020	A / H5N8	Asia / Kazakhst	Dove	Alexandr Shevt	National Center	Asylulan,Amir	National Vete	2020-09-15
EPI_ISL_96162	A/red knot/Netherlands/22000409-002/2022	A / H5N1	Europe / Nethe	Calidris car	Rene Heutink (\	Wageningen Bi	Beerens, Nar	Wageningen	2022-01-06
EPI_ISL_90094	A/muscovy duck/Nghe An/7006VTC/2020	A / H5N6	Asia / Vietnam	Cairina mos	Natalia Goncha	State Research		State Resea	2020-12-28
EPI_ISL_90094	A/muscovy duck/Nghe An/6873VTC/2020	A / H5N6	Asia / Vietnam	Cairina mos	Natalia Goncha	State Research		State Resea	2020-12-21
EPI_ISL_65485	A/muscovy duck/Thanh Hoa/892VTC/2020	A / H5N6	Asia / Vietnam	Cairina mos	Natalia Goncha	State Research		State Resea	2020-02-21
EPI_ISL_65485	A/muscovy duck/Thanh Hoa/879VTC/2020	A / H5N6	Asia / Vietnam	Cairina mos	Natalia Goncha	State Research		State Resea	2020-02-20
EPI_ISL_13910	A/common golden eye/Sweden/SVA210316SZ	A / H5N5	Europe / Swede	Bucephala	Siamak Zohari	National Veterii		National Vete	2021-03-15
EPI_ISL_14777	A/Sanderling/Delaware/518/2021	A / H5N2	North America /	Calidris alb			Direct Submis		2021-06-01

EPI_ISL_10635	A/Mandarin duck/Kagoshima/KU-d57/2020	A / H5N8	Asia / Japan / K	Aix galericu	Ahmed Magdy	Kagoshima Uni			Kagoshima U	2020-12-22
EPI_ISL_61018	A/black-headed gull/Netherlands/21037589-00	A / H5N1	Europe / Nethe	Chroicocep	Rene Heutink ('	Wageningen Bi	Beerens, Nar		Wageningen	2021-10-27
EPI_ISL_14392	A/Branta_leucopsis/Belgium/2606_0009/2022	A / H5N1	Europe / Belgiu	Branta leuc	Steven Van Bo	Sciensano, Dep	Van Borm, St		Sciensano -	2022-02-17
EPI_ISL_12474	A/barnacle goose/Netherlands/22007405-004/2	A / H5N1	Europe / Nethe	Branta leuc	Rene Heutink ('	Wageningen Bi	Beerens, Nar		Wageningen	2022-04-17
EPI_ISL_30059	A/barnacle goose/Sweden/SVA210511SZ0567	A / H5N1	Europe / Swede	Branta leuc	Siamak Zohari	National Veterii			National Vet	2021-05-11
EPI_ISL_22272	A/barnacle goose/Netherlands/21028534-002/2	A / H5N1	Europe / Nethe	Branta leuc	Rene Heutink ('	Wageningen Bi	Beerens, Nar		Wageningen	2021-05-13
EPI_ISL_22272	A/barnacle goose/Netherlands/21027357-002/2	A / H5N1	Europe / Nethe	Branta leuc	Rene Heutink ('	Wageningen Bi	Beerens, Nar		Wageningen	2021-04-20
EPI_ISL_21940	A/barnacle goose/Netherlands/21028196-002/2	A / H5N1	Europe / Nethe	Branta leuc	Rene Heutink ('	Wageningen Bi	Beerens, Nar		Wageningen	2021-04-22
EPI_ISL_21939	A/barnacle goose/Netherlands/21027016-002/2	A / H5N1	Europe / Nethe	Branta leuc	Rene Heutink ('	Wageningen Bi	Beerens, Nar		Wageningen	2021-04-15
EPI_ISL_10482	A/barnacle goose/Netherlands/21021591-001/2	A / H5N1	Europe / Nethe	Branta leuc	Rene Heutink ('	Wageningen Bi	Beerens, Nar		Wageningen	2021-01-14
EPI_ISL_82629	A/barnacle goose/Sweden/SVA201221SZ0154	A / H5N8	Europe / Swede	Branta leuc	Siamak Zohari	National Veterii			National Vet	2020-12-15
EPI_ISL_82628	A/barnacle goose/Sweden/SVA201215SZ0368	A / H5N8	Europe / Swede	Branta leuc	Siamak Zohari	National Veterii			National Vet	2020-12-09
EPI_ISL_13114	A/Broiler chicken/BC/FAV-0228-OS/2022	A / H5N1	North America /	Gallus gallu	Yohannes Berh	Canadian Food	Hisanaga, Ta		Animal Healt	2022-04-12
EPI_ISL_90095	A/chicken/Ha Tinh/514VTC/2021	A / H5N6	Asia / Vietnam	Gallus gallu	Natalia Goncha	State Research			State Resea	2021-02-04
EPI_ISL_90095	A/chicken/Thanh Hoa/1351VTC/2021	A / H5N6	Asia / Vietnam	Gallus gallu	Natalia Goncha	State Research			State Resea	2021-03-15
EPI_ISL_90094	A/chicken/Nghe An/7007VTC/2020	A / H5N6	Asia / Vietnam	Gallus gallu	Natalia Goncha	State Research			State Resea	2020-12-28
EPI_ISL_90094	A/chicken/Thanh Hoa/V3S3VTC/2020	A / H5N6	Asia / Vietnam	Gallus gallu	Natalia Goncha	State Research			State Resea	2020-07-21
EPI_ISL_90094	A/chicken/Quang Tri/V4S4VTC/2020	A / H5N6	Asia / Vietnam	Gallus gallu	Natalia Goncha	State Research			State Resea	2020-10-30
EPI_ISL_78806	A/Gallus_gallus/Belgium/15977/2021	A / H5N1	Europe / Belgiu	Gallus gallu	Steven Van Bo	Sciensano, Dep	Van Borm, St		Sciensano -	2021-12-06
EPI_ISL_78806	A/Gallus_gallus/Belgium/16070_003/2021	A / H5N1	Europe / Belgiu	Gallus gallu	Steven Van Bo	Sciensano, Dep	Van Borm, St		Sciensano -	2021-12-07
EPI_ISL_76265	A/chicken/Czech Republic/23589-4/2021	A / H5N1	Europe / Czech	Gallus gallu	Alexander Nagy	State Veterinar	Alexander,Na		State Veterir	2021-11-29
EPI_ISL_76265	A/chicken/Czech Republic/23589-3/2021	A / H5N1	Europe / Czech	Gallus gallu	Alexander Nagy	State Veterinar	Alexander,Na		State Veterir	2021-11-29
EPI_ISL_76265	A/chicken/Czech Republic/23589-1T/2021	A / H5N1	Europe / Czech	Gallus gallu	Alexander Nagy	State Veterinar	Alexander,Na		State Veterir	2021-11-29
EPI_ISL_76265	A/chicken/Czech Republic/23404-4T/2021	A / H5N1	Europe / Czech	Gallus gallu	Alexander Nagy	State Veterinar	Alexander,Na		State Veterir	2021-11-25
EPI_ISL_76265	A/chicken/Czech Republic/23404-2T/2021	A / H5N1	Europe / Czech	Gallus gallu	Alexander Nagy	State Veterinar	Alexander,Na		State Veterir	2021-11-25
EPI_ISL_76264	A/chicken/Czech Republic/23404-2K/2021	A / H5N1	Europe / Czech	Gallus gallu	Alexander Nagy	State Veterinar	Alexander,Na		State Veterir	2021-11-25
EPI_ISL_74528	A/Chicken/Sweden/SVA211130SZ0427/FB290	A / H5N1	Europe / Swede	Gallus gallu	Siamak Zohari	National Veterii			National Vet	2021-11-30
EPI_ISL_72244	A/chicken/Czech Republic/22224-4T/2021	A / H5N1	Europe / Czech	Gallus gallu	Alexander Nagy	State Veterinar	Alexander,Na		State Veterir	2021-11-12
EPI_ISL_72244	A/chicken/Czech Republic/22224-3T/2021	A / H5N1	Europe / Czech	Gallus gallu	Alexander Nagy	State Veterinar	Alexander,Na		State Veterir	2021-11-12
EPI_ISL_72244	A/chicken/Czech Republic/22224-3K/2021	A / H5N1	Europe / Czech	Gallus gallu	Alexander Nagy	State Veterinar	Alexander,Na		State Veterir	2021-11-12
EPI_ISL_72244	A/chicken/Czech Republic/22224-2T/2021	A / H5N1	Europe / Czech	Gallus gallu	Alexander Nagy	State Veterinar	Alexander,Na		State Veterir	2021-11-12
EPI_ISL_54638	A/chicken/Tyumen/33-45V/2021	A / H5N1	Europe / Russia	Gallus gallu	Natalia Goncha	State Research	Natalia,Goncl		FBUZ Cente	2021-10-07
EPI_ISL_54638	A/chicken/Saratov/29-07V/2021	A / H5N1	Europe / Russia	Gallus gallu	Natalia Goncha	State Research	Natalia,Goncl		Center of Hy	2021-09-30
EPI_ISL_54638	A/chicken/Saratov/29-06V/2021	A / H5N1	Europe / Russia	Gallus gallu	Natalia Goncha	State Research	Natalia,Goncl		Center of Hy	2021-09-30
EPI_ISL_54637	A/chicken/Tyumen/27-42V/2021	A / H5N1	Europe / Russia	Gallus gallu	Natalia Goncha	State Research	Natalia,Goncl		FBUZ Cente	2021-10-06
EPI_ISL_54637	A/chicken/Tyumen/27-40V/2021	A / H5N1	Europe / Russia	Gallus gallu	Natalia Goncha	State Research	Natalia,Goncl		FBUZ Cente	2021-10-06
EPI_ISL_54637	A/chicken/Tyumen/27-39V/2021	A / H5N1	Europe / Russia	Gallus gallu	Natalia Goncha	State Research	Natalia,Goncl		FBUZ Cente	2021-10-06
EPI_ISL_54637	A/chicken/Tyumen/27-31V/2021	A / H5N1	Europe / Russia	Gallus gallu	Natalia Goncha	State Research	Natalia,Goncl		FBUZ Cente	2021-10-06
EPI_ISL_29326	A/chicken/Kazakhstan/1-20-B-Talg-67/2020	A / H5N8	Asia / Kazakhst	Gallus gallu	Alexandr Shevt	National Center	Asylulan,Ami		National Vet	2020-10-09
EPI_ISL_29326	A/chicken/Kazakhstan/12-20-B-Talg-45/2020	A / H5N8	Asia / Kazakhst	Gallus gallu	Alexandr Shevt	National Center	Asylulan,Ami		National Vet	2020-09-19
EPI_ISL_29326	A/chicken/Kazakhstan/220-B-2-H5N8-4/2020	A / H5N8	Asia / Kazakhst	Gallus gallu	Alexandr Shevt	National Center	Asylulan,Ami		National Vet	2020-09-01
EPI_ISL_12356	A/Chicken/Sweden/SVA210301SZ0001/KN049	A / H5N8	Europe / Swede	Gallus gallu	Siamak Zohari	National Veterii			National Vet	2021-02-28

EPI_ISL_1219(	A/Chicken/Sweden/SVA210226SZ0168/KN048	A / H5N5	Europe / Swede	Gallus gallu	Siamak Zohari	National Veterii		National Vete	2021-02-24
EPI_ISL_10392	A/chicken/Astrakhan/321-10/2020	A / H5N8	Europe / Russia	Gallus gallu	Natalia Goncha	State Research	Natalia,Goncl	State Resea	2020-12-12
EPI_ISL_10392	A/chicken/Astrakhan/321-10/2020	A / H5N8	Europe / Russia	Gallus gallu	Natalia Goncha	State Research	Natalia,Goncl	State Resea	2020-12-12
EPI_ISL_10392	A/chicken/Astrakhan/321-09/2020	A / H5N8	Europe / Russia	Gallus gallu	Natalia Goncha	State Research	Natalia,Goncl	State Resea	2020-12-12
EPI_ISL_10392	A/chicken/Astrakhan/321-06/2020	A / H5N8	Europe / Russia	Gallus gallu	Natalia Goncha	State Research	Natalia,Goncl	State Resea	2020-12-12
EPI_ISL_10392	A/chicken/Astrakhan/321-05/2020	A / H5N8	Europe / Russia	Gallus gallu	Natalia Goncha	State Research	Natalia,Goncl	State Resea	2020-12-12
EPI_ISL_10392	A/chicken/Astrakhan/321-05/2020	A / H5N8	Europe / Russia	Gallus gallu	Natalia Goncha	State Research		State Resea	2020-12-12
EPI_ISL_10392	A/chicken/Astrakhan/321-01/2020	A / H5N8	Europe / Russia	Gallus gallu	Natalia Goncha	State Research		State Resea	2020-12-12
EPI_ISL_10392	A/chicken/Astrakhan/321-01/2020	A / H5N8	Europe / Russia	Gallus gallu	Natalia Goncha	State Research		State Resea	2020-12-12
EPI_ISL_82645	A/Chicken/Sweden/SVA210102SZ0002/KN00(	A / H5N8	Europe / Swede	Gallus gallu	Siamak Zohari	National Veterii		National Vete	2021-01-02
EPI_ISL_82628	A/Chicken/Sweden/SVA201221SZ0027/KN342	A / H5N8	Europe / Swede	Gallus gallu	Siamak Zohari	National Veterii		National Vete	2020-12-21
EPI_ISL_65485	A/chicken/Thanh Hoa/1152VTC/2020	A / H5N6	Asia / Vietnam	Gallus gallu	Natalia Goncha	State Research		State Resea	2020-03-06
EPI_ISL_65485	A/chicken/Thanh Hoa/980VTC/2020	A / H5N6	Asia / Vietnam	Gallus gallu	Natalia Goncha	State Research		State Resea	2020-02-26
EPI_ISL_65485	A/chicken/Thanh Hoa/968VTC/2020	A / H5N6	Asia / Vietnam	Gallus gallu	Natalia Goncha	State Research		State Resea	2020-02-26
EPI_ISL_65484	A/chicken/Thanh Hoa/844VTC/2020	A / H5N6	Asia / Vietnam	Gallus gallu	Natalia Goncha	State Research		State Resea	2020-02-18
EPI_ISL_65484	A/chicken/Nghe An/842VTC/2020	A / H5N6	Asia / Vietnam	Gallus gallu	Natalia Goncha	State Research		State Resea	2020-02-18
EPI_ISL_65484	A/chicken/Thanh Hoa/VTC741/2020	A / H5N6	Asia / Vietnam	Gallus gallu	Natalia Goncha	State Research		State Resea	2020-02-13
EPI_ISL_65483	A/chicken/Kurgan/1010/2020	A / H5N8	Europe / Russia	Gallus gallu	Natalia Goncha	State Research		State Resea	2020-08-27
EPI_ISL_65483	A/chicken/Kurgan/1005/2020	A / H5N8	Europe / Russia	Gallus gallu	Natalia Goncha	State Research		State Resea	2020-08-27
EPI_ISL_65483	A/chicken/Kurgan/1004/2020	A / H5N8	Europe / Russia	Gallus gallu	Natalia Goncha	State Research	Natalia,Goncl	State Resea	2020-08-27
EPI_ISL_65483	A/chicken/Kurgan/1003/2020	A / H5N8	Europe / Russia	Gallus gallu	Natalia Goncha	State Research	Natalia,Goncl	State Resea	2020-08-27
EPI_ISL_65483	A/chicken/Kurgan/1001/2020	A / H5N8	Europe / Russia	Gallus gallu	Natalia Goncha	State Research	Natalia,Goncl	State Resea	2020-08-27
EPI_ISL_65483	A/chicken/Chelyabinsk/201/2020	A / H5N8	Europe / Russia	Gallus gallu	Natalia Goncha	State Research	Natalia,Goncl	State Resea	2020-09-08
EPI_ISL_65483	A/chicken/Omsk/30007/2020	A / H5N8	Europe / Russia	Gallus gallu	Natalia Goncha	State Research	Natalia,Goncl	State Resea	2020-09-03
EPI_ISL_65482	A/chicken/Saratov/29801/2020	A / H5N8	Europe / Russia	Gallus gallu	Natalia Goncha	State Research	Natalia,Goncl	State Resea	2020-09-15
EPI_ISL_58637	A/green-winged-teal/Georgia/DT-22894/2020	A / H5N3	Asia / Georgia	Green-wing	Nicola Lewis (F	Royal Veterinai	Fouchier, R.A	Erasmus Me	2020-10-20
EPI_ISL_58635	A/green-winged-teal/Georgia/DT-22392/2020	A / H5N8	Asia / Georgia	Green-wing	Nicola Lewis (F	Royal Veterinai	Fouchier, R.A	Erasmus Me	2020-10-06
EPI_ISL_58635	A/green-winged-teal/Georgia/DT-22978/2020	A / H5N8	Asia / Georgia	Green-wing	Nicola Lewis (F	Royal Veterinai	Fouchier, R.A	Erasmus Me	2020-10-23
EPI_ISL_67814	A/Whooper Swan/Khuvsgul/#4/2020	A / H5N6	Asia / Mongolia	Whooper sv			Duong,B.T.; T		2020-05
EPI_ISL_67814	A/Whooper Swan/Khuvsgul/#1/2020	A / H5N6	Asia / Mongolia	Whooper sv			Duong,B.T.; T		2020-05
EPI_ISL_67812	A/Whooper swan/Mongolia/03/2020	A / H5N6	Asia / Mongolia	Whooper sv			Ankhanbaata		2020-04
EPI_ISL_67811	A/Whooper swan/Mongolia/01/2020	A / H5N6	Asia / Mongolia	Whooper sv			Ankhanbaata		2020-04
EPI_ISL_40617	A/whooper swan/Fukushima/0701B002/2021	A / H5N8	Asia / Japan / F	Whooper sv			Sakoda,Y.; Is		2021-02-08
EPI_ISL_40617	A/whooper swan/Miyagi/0402B001/2021	A / H5N8	Asia / Japan / M	Whooper sv			Sakoda,Y.; Is		2021-02-17
EPI_ISL_31439	A/whooper swan/Sweden/SVA2100329SZ021(	A / H5N5	Europe / Swede	Whooper sv	Siamak Zohari	National Veterii		National Vete	2021-07-06
EPI_ISL_31278	A/whooper swan/Sweden/SVA210331SZ0441/	A / H5N8	Europe / Swede	Whooper sv	Siamak Zohari	National Veterii		National Vete	2021-03-29
EPI_ISL_18341	A/whooper swan/Henan/SMQ6/2020	A / H5N8	Asia / China / H	Whooper sv	Hongliang Chai	Northeast Fores		College of W	2020-11-06
EPI_ISL_17604	A/whooper swan/Shanxi/SX116/2020	A / H5N2	Asia / China / S	Whooper sv	Hongliang Chai	Northeast Fores		College of W	2020-11-10
EPI_ISL_17604	A/whooper swan/Shanxi/SX106/2020	A / H5N8	Asia / China / S	Whooper sv	Hongliang Chai	Northeast Fores		College of W	2020-11-10
EPI_ISL_17604	A/whooper swan/Shanxi/SX56/2020	A / H5N8	Asia / China / S	Whooper sv	Hongliang Chai	Northeast Fores		College of W	2020-11-10
EPI_ISL_17604	A/whooper swan/Shanxi/SX31/2020	A / H5N8	Asia / China / S	Whooper sv	Hongliang Chai	Northeast Fores		College of W	2020-11-10
EPI_ISL_17604	A/whooper swan/Shanxi/SX16/2020	A / H5N8	Asia / China / S	Whooper sv	Hongliang Chai	Northeast Fores		College of W	2020-11-10

EPI_ISL_17604	A/whooper swan/Shaanxi/SXY2-1/2020	A / H5N8	Asia / China / S	Whooper sw	Hongliang Chai	Northeast Fores		College of W	2020-11-10
EPI_ISL_17604	A/whooper swan/Shaanxi/SXY66/2020	A / H5N8	Asia / China / S	Whooper sw	Hongliang Chai	Northeast Fores		College of W	2020-11-10
EPI_ISL_17604	A/whooper swan/Shaanxi/SXY26/2020	A / H5N8	Asia / China / S	Whooper sw	Hongliang Chai	Northeast Fores		College of W	2020-11-09
EPI_ISL_65125	A/goose/Czech Republic/20689-28T/2021	A / H5N1	Europe / Czech	Anser anse	Alexander Nagy	State Veterinary	Nagy,Alexand	State Veterir	2021-10-22
EPI_ISL_65119	A/goose/Czech Republic/20689-27T/2021	A / H5N1	Europe / Czech	Anser anse	Alexander Nagy	State Veterinary	Nagy,Alexand	State Veterir	2021-10-22
EPI_ISL_54638	A/goose/Tyumen/33-53V/2021	A / H5N1	Europe / Russia	Anser anse	Natalia Goncha	State Research	Natalia,Goncl	FBUZ Cente	2021-10-07
EPI_ISL_54638	A/goose/Tyumen/33-52V/2021	A / H5N1	Europe / Russia	Anser anse	Natalia Goncha	State Research	Natalia,Goncl	FBUZ Cente	2021-10-07
EPI_ISL_53233	A/goose/Czech Republic/18520-1/2021	A / H5N1	Europe / Czech	Anser anse	Alexander Nagy	State Veterinary	Alexander,Na	State Veterir	2021-09-27
EPI_ISL_12369	A/goose/Sweden/SVA210301SZ0005/KN0498	A / H5N8	Europe / Swede	Anser anse	Siamak Zohari	National Veterii		National Vete	2021-02-27
EPI_ISL_65483	A/goose/Kurgan/01/2020	A / H5N8	Europe / Russia	Anser anse	Natalia Goncha	State Research		State Resea	2020-08-27
EPI_ISL_65483	A/goose/Omsk/30010/2020	A / H5N8	Europe / Russia	Anser anse	Natalia Goncha	State Research	Natalia,Goncl	State Resea	2020-09-03
EPI_ISL_65483	A/goose/Omsk/30009/2020	A / H5N8	Europe / Russia	Anser anse	Natalia Goncha	State Research	Natalia,Goncl	State Resea	2020-09-03
EPI_ISL_65482	A/goose/Omsk/30006/2020	A / H5N8	Europe / Russia	Anser anse	Natalia Goncha	State Research	Natalia,Goncl	State Resea	2020-09-03
EPI_ISL_65482	A/goose/Omsk/30004/2020	A / H5N8	Europe / Russia	Anser anse	Natalia Goncha	State Research	Natalia,Goncl	State Resea	2020-09-03
EPI_ISL_65482	A/goose/Omsk/30003/2020	A / H5N8	Europe / Russia	Anser anse	Natalia Goncha	State Research	Natalia,Goncl	State Resea	2020-09-03
EPI_ISL_65482	A/goose/Omsk/30001/2020	A / H5N8	Europe / Russia	Anser anse	Natalia Goncha	State Research	Natalia,Goncl	State Resea	2020-09-03
EPI_ISL_54638	A/duck/Saratov/29-11V/2021	A / H5N1	Europe / Russia	Anas platyr	Natalia Goncha	State Research	Natalia,Goncl	Center of Hy	2021-09-30
EPI_ISL_54638	A/duck/Saratov/29-08V/2021	A / H5N1	Europe / Russia	Anas platyr	Natalia Goncha	State Research	Natalia,Goncl	Center of Hy	2021-09-30
EPI_ISL_54637	A/duck/Saratov/29-04V/2021	A / H5N1	Europe / Russia	Anas platyr	Natalia Goncha	State Research	Natalia,Goncl	Center of Hy	2021-09-30
EPI_ISL_54637	A/duck/Saratov/29-03V/2021	A / H5N1	Europe / Russia	Anas platyr	Natalia Goncha	State Research	Natalia,Goncl	Center of Hy	2021-09-30
EPI_ISL_54637	A/duck/Saratov/29-02V/2021	A / H5N1	Europe / Russia	Anas platyr	Natalia Goncha	State Research	Natalia,Goncl	Center of Hy	2021-09-30
EPI_ISL_65486	A/duck/Thanh Hoa/2202VTC/2020	A / H5N6	Asia / Vietnam	Anas platyr	Natalia Goncha	State Research		State Resea	2020-05-02
EPI_ISL_65486	A/duck/Nghe An/1693VTC/2020	A / H5N6	Asia / Vietnam	Anas platyr	Natalia Goncha	State Research		State Resea	2020-04-04
EPI_ISL_65486	A/duck/Thanh Hoa/1182VTC/2020	A / H5N6	Asia / Vietnam	Anas platyr	Natalia Goncha	State Research		State Resea	2020-03-06
EPI_ISL_65486	A/duck/Thanh Hoa/1180VTC/2020	A / H5N6	Asia / Vietnam	Anas platyr	Natalia Goncha	State Research		State Resea	2020-03-06
EPI_ISL_65485	A/duck/Thanh Hoa/1151VTC/2020	A / H5N6	Asia / Vietnam	Anas platyr	Natalia Goncha	State Research		State Resea	2020-03-05
EPI_ISL_65485	A/duck/Thanh Hoa/1107VTC/2020	A / H5N6	Asia / Vietnam	Anas platyr	Natalia Goncha	State Research		State Resea	2020-03-03
EPI_ISL_65485	A/duck/Thanh Hoa/945VTC/2020	A / H5N6	Asia / Vietnam	Anas platyr	Natalia Goncha	State Research		State Resea	2020-02-24
EPI_ISL_65485	A/duck/Thanh Hoa/923VTC/2020	A / H5N6	Asia / Vietnam	Anas platyr	Natalia Goncha	State Research		State Resea	2020-02-22
EPI_ISL_65485	A/duck/Thanh Hoa/893VTC/2020	A / H5N6	Asia / Vietnam	Anas platyr	Natalia Goncha	State Research		State Resea	2020-02-21
EPI_ISL_65484	A/duck/Thanh Hoa/808VTC/2020	A / H5N6	Asia / Vietnam	Anas platyr	Natalia Goncha	State Research		State Resea	2020-02-16
EPI_ISL_65484	A/duck/Thanh Hoa/752VTC/2020	A / H5N6	Asia / Vietnam	Anas platyr	Natalia Goncha	State Research		State Resea	2020-02-13
EPI_ISL_65484	A/duck/Thanh Hoa/740VTC/2020	A / H5N6	Asia / Vietnam	Anas platyr	Natalia Goncha	State Research		State Resea	2020-02-13
EPI_ISL_65484	A/duck/Thanh Hoa/722VTC/2020	A / H5N6	Asia / Vietnam	Anas platyr	Natalia Goncha	State Research		State Resea	2020-02-14
EPI_ISL_65484	A/duck/Nghe An/695VTC/2020	A / H5N6	Asia / Vietnam	Anas platyr	Natalia Goncha	State Research		State Resea	2020-02-11
EPI_ISL_65484	A/duck/Nghe An/694VTC/2020	A / H5N6	Asia / Vietnam	Anas platyr	Natalia Goncha	State Research		State Resea	2020-02-11
EPI_ISL_65484	A/duck/Thanh Hoa/676VTC/2020	A / H5N6	Asia / Vietnam	Anas platyr	Natalia Goncha	State Research		State Resea	2020-02-10
EPI_ISL_65482	A/duck/Saratov/29804/2020	A / H5N8	Europe / Russia	Anas platyr	Natalia Goncha	State Research	Natalia,Goncl	State Resea	2020-09-15
EPI_ISL_17604	A/common teal/Shaanxi/SXY1-1/2020	A / H5N8	Asia / China / S	Common te	Hongliang Chai	Northeast Fores		College of W	2020-11-10
EPI_ISL_14835	A/Bean Goose(Anser fabalis)/South Korea/KNU	A / H5N8	Asia / Korea, R	Bean goose			Tran,K.N.T.; F		2021-04-06
EPI_ISL_14835	A/Bean Goose(Anser fabalis)/South Korea/KNU	A / H5N8	Asia / Korea, R	Bean goose			Tran,K.N.T.; F		2021-02-17
EPI_ISL_75857	A/Aalopochen_Aegyptiaca/Belgium/2928_002/	A / H5N8	Europe / Belgiu	Anseriforme	Steven Van Bor	Sciensano, Dep	Van Borm, St	Sciensano -	2021-02-28

EPI_ISL_88141	A/turkey/England/056764/2021	A / H5N1	Europe / Unitec	Meleagris g	Alex Byrne (Ani	Animal and Pla		Animal and F	2021-11-10
EPI_ISL_88141	A/turkey/England/055251/2021	A / H5N1	Europe / Unitec	Meleagris g	Alex Byrne (Ani	Animal and Pla		Animal and F	2021-11-06
EPI_ISL_83380	A/Turkey/Sweden/SVA211212SZ0001/FB3010	A / H5N1	Europe / Swede	Meleagris g	Siamak Zohari	National Veteri	'Siamak, Zoha	National Vet	2021-12-12
EPI_ISL_69302	A/turkey/Poland/H1913-T1/2021	A / H5N1	Europe / Polan	Meleagris g	Edyta Świętoń	National Veteri	E. Swieton, K	National Vet	2021-11-02
EPI_ISL_69299	A/turkey/Poland/H1911-N/2021	A / H5N1	Europe / Polan	Meleagris g	Edyta Świętoń	National Veteri	E. Swieton, K	National Vet	2021-11-01
EPI_ISL_69299	A/turkey/Poland/H1910-T3/2021	A / H5N1	Europe / Polan	Meleagris g	Edyta Świętoń	National Veteri	E. Swieton, K	National Vet	2021-11-01
EPI_ISL_31358	A/Turkey/Hungary/16603/2021 (H5N1)	A / H5N1	Europe / Hunga	Meleagris g	Katalin Szentpá	National Food (	Katalin,Szent	National Foo	2021-04-13
EPI_ISL_22272	A/turkey/Netherlands/21028936-001005/2021	A / H5N8	Europe / Nethe	Meleagris g	Rene Heutink ('	Wageningen Bi	Beerens, Nar	Wageningen	2021-05-21
EPI_ISL_98697	A/American wigeon/South Carolina/AH019514	A / H5N1	North America /	Wild waterf	Mary Lea Killiar	National Veteri	Chinh,Thanh;	USDA-NWR	2021-12-30
EPI_ISL_14393	A/Fox/Netherlands/EMC6/2022	A / H5N1	Europe / Nethe	mammals	Sanne Thewess	Erasmus Medic		Erasmus Me	2022-02-15
