# Supplementary material for: Introduction of Multiple Novel High Pathogenicity Avian Influenza (H5N1) Virus of Clade 2.3.4.4b into South Korea in 2022
Source: Transbound Emerg Dis. 2023 Apr 13;2023:8339427. doi: 10.1155/2023/8339427 (PMC12017251; doi:10.1155/2023/8339427)
Supplement: Supplementary Materials — Supplementary Table S1: List of viruses from the GISAID EPIFLU on which this research is based (Table 2). All available AIV sequences (approximately 3,200) were obtained from the GISAID EpiFlu database (https://www.gisaid.org) on 17 October 2022. Initially, RAxML was used to construct the phylogenetic trees using all eight genes of the H5NX strains collected after 2020, as well as sequences that were genetically close to MD/WA496, CT/WA537, and BD/H493 (Supplementary Table S1): Among these, representative sequences (50–60) from one cluster of the phylogenetic tree were selected after considering genetic homology, collection date, and geographical location. Phylogenetic trees were generated using the final datasets, and included reference strains detected in the past in Korea. Supplementary Table S2. List of reference H5NX isolates used for tMRCA. To identify the tMRCA of G10-like H5NX HPAI, representative H5NX AI viruses and the internal genes of other subtypes were selected based on genetic homology, geographical location, and collection date (Supplement Table S2): Complete coding sequences of each gene segment were used for comparative phylogenetic analyses. Multiple sequence alignment (PB2: 2280 bp, PB1: 2274 bp, PA: 2151 bp, HA: 1704 bp, NP: 1497 bp, NA: 1413 bp, M: 982 bp, and NS: 838 bp) was performed using MAFFT (https://phylo.org). Supplementary Table S3: Initial three cases and AI surveillance system of HPAI in 2022/2023. Maximum-likelihood phylogenetic trees for the PB2 gene (Figure S1a), PB1 gene (Figure S1b), PA gene (Figure S1c), NP gene (Figure S1d), MP gene (Figure S1e), and NS gene (Figure S1f). The phylogenetic trees are based on H5N1 viruses isolated recently, as well as other H5Nx viruses. Bootstrap values (1,000 replicates) >70% are displayed at the branch nodes. A/mandarin duck/Korea/WA496/2022 (H5N1) and A/breeder duck/Korea/H493/2022 (H5N1) are indicated by a blue solid circle. A/common teal/Korea/WA537/2022 (H5N1) is indicated by a red solid [file 8339427.f1.zip › Supplementary Table2-edit.pdf]

Number	Accession numbe	Collection_Date	STRAIN NAME	HA Subtype	HPAI/LPAI	Segment number used in tMRCA
1	EPI_ISL_157609	2014-01-16	A/breeder_duck/Korea/Gochang1/2014	H5N8	HP	1/2/3/4/5/7/8
2	EPI_ISL_157610	2014-01-17	A/broiler_duck/Korea/Buan2/2014	H5N8	HP	1/2/3/4/5/7/8
3	EPI_ISL_239261	2016-11-16	A/chicken/Korea/HN1/2016(H5N6)	H5N6	HP	1/2/3/4/5/7/8
4	EPI_ISL_288439	2017-02-06	A/chicken/Korea/Gimje2/2017	H5N8	HP	1/2/3/4/5/7/8
5	EPI_ISL_288437	2017-11-17	A/duck/Korea/HD1/2017	H5N6	HP	1/2/3/4/5/7/8
6	EPI_ISL_292345	2017-12-10	A/duck/Korea/H35/2017	H5N6	HP	1/2/3/4/5/7/8
7	EPI_ISL_503513	2019-01-17	/A/duck/Bangladesh/37631/2019	H10	LP	1
8	EPI_ISL_503499	2019-02-18	/A/duck/Bangladesh/38285/2019	H11	LP	1
9	EPI_ISL_4071028	2019-03-18	A/Mallard/Anas_platyrhynchos/South/Korea/KNU2	H1	LP	1
10	EPI_ISL_445093	2019-04-26	A/shorebird/Liaoning/dandong786/2019	H7	LP	1
11	EPI_ISL_400267	2019-09-07	A_Common_Teal_Chany_Lake_29_2019	H14	LP	1
12	EPI_ISL_503357	2019-09-20	A/duck/Mongolia/826/2019	H4	LP	1
13	EPI_ISL_503359	2019-09-20	/A/duck/Mongolia/961/2019	H3	LP	1
14	EPI_ISL_9572802	2019-12-30	/A/duck/Vietnam/HN6039/2019	H6	LP	1
15	EPI_ISL_404993	2020-01-16	A/white-fronted goose/Germany-BB/AI00018/202	H5N8	HP	1/2/3/4/5/7/8
16	EPI_ISL_9572775	2020-01-20	/A/duck/Vietnam/QN6085/2020	H6	LP	1
17	EPI_ISL_6795222	2020-02-03	/A/Bean/goose/Anser/fabalis/South/Korea/KNU20	H11	LP	1
18	EPI_ISL_525449	2020-02-20	A/domestic_duck/Poland/219/2020	H5N8	HP	1/2/3/4/5/7/8
19	EPI_ISL_623074	2020-05-12	A/chicken/Iraq/1/2020	H5N8	HP	1/2/3/4/5/7/8
20	EPI_ISL_661178	2020-09-10	A/swan/Tumen/1479-2/2020	H5N8	HP	1/2/3/4/5/7/8
21	EPI_ISL_2932612	2020-09-22	A/goose/Kazakhstan/7-20-B-Talg-12/2020	H5N8	HP	1/2/3/4/5/7/8
22	EPI_ISL_6784992	2020-10-12	/A/Wild/Duck/South/Korea/KNU2020-110/2020	H9	LP	1
23	EPI_ISL_14775931	2020-10-20	A/duck/Bangladesh/45997/2020	H1	LP	1
24	EPI_ISL_631824	2020-10-21	A/Mandarin duck/Korea/H242/2020	H5N8	HP	1/2/3/4/5/7/8
25	EPI_ISL_697771	2020-10-24	A/northern pintail/Hokkaido/M13/2020	H5N8	HP	1/2/3/4/5/7/8
26	EPI_ISL_614400	2020-10-28	A/barnacle goose/Germany-SH/AI02167/2020	H5N8	HP	1/2/3/4/5/7/8
27	EPI_ISL_1009710	2020-11-17	A/wild duck/Korea/H331/2020	H5N8	HP	1/2/3/4/5/7/8
28	EPI_ISL_683592	2020-11-21	A/Eurasian_wigeon/Italy/20VIR7301-206/2020	H5N1	HP	1/2/3/4/5/6/7/8
29	EPI_ISL_1665262	2021-01-15	A/mute_swan/Slovakia/Pah6_21VIR1086-2/2021	H5N5	HP	1/2/3/4/5/7/8

30	EPI_ISL_4061484	2021-03-01	A/chicken/Nigeria/VRD21-102_21VIR2370-424/202	H5N1	HP	1/2/3/4/5/6/7/8
31	EPI_ISL_3135897	2021-04-13	A/Turkey/Hungary/16603/2021 (H5N1)	H5N1	HP	1/2/3/4/5/6/7/8
32	EPI_ISL_2681045	2021-04-20	A/white_stork/Poland/MB391/2021	H5N1	HP	1/2/3/4/5/6/7/8
33	EPI_ISL_5463803	2021-09-30	A/duck/Saratov/29-11V/2021	H5N1	HP	1/2/3/4/5/6/7/8
34	EPI_ISL_5463806	2021-10-07	A/goose/Tyumen/33-53V/2021	H5N1	HP	1/2/3/4/5/6/7/8
35	EPI_ISL_8769018	2021-10-12	A/chicken/Tyumen/47-79V/2021	H5N1	HP	4
36	EPI_ISL_5524384	2021-10-18	A/turkey/Italy/21VIR8585-1/2021	H5N1	HP	1/2/3/4/5/6/7/8
37	EPI_ISL_5804708	2021-10-24	A/mute_swan/England/053054/2021	H5N1	HP	1/2/3/4/5/6/7/8
38	EPI_ISL_6959592	2021-10-26	A/mandarin duck/Korea/WA585/2021	H5N1	HP	1/2/3/4/5/6/7/8
39	EPI_ISL_8769034	2021-10-30	A/chicken/Tyumen/81-97V/2021	H5N1	HP	4
40	EPI_ISL_6829533	2021-11-12	A/chicken/Kagoshima/21A6T/2021	H5N1	HP	1/2/3/4/5/6/7/8
41	EPI_ISL_12572664	2021-11-25	A/wild duck/Hebei/SD012/2021	H5N1	HP	1/2/3/4/5/6/7/8
42	EPI_ISL_7635907	2021-11-26	A/white-tailed eagle/Sweden/SVA211201SZ0380/I	H5N1	HP	1/2/3/4/5/6/7/8
43	EPI_ISL_11007540	2021-12-01	A/duck/Bulgaria/756-4_22VIR778-6/2021	H5N1	HP	1/2/3/4/5/6/7/8
44	EPI_ISL_12968817	2021-12-17	A/emu/NL/FAV-0035-12/2021	H5N1	HP	1/2/3/4/5/6/7/8
45	EPI_ISL_14886367	2021-12-19	A/duck/Bangladesh/51602/2021	H5N1	HP	1/2/3/4/5/6/7/8
46	EPI_ISL_11633614	2021-12-20	A/Cygnus columbianus/Hubei/121/2021	H5N1	HP	1/2/3/4/5/6/7/8
47	EPI_ISL_11633707	2021-12-20	A/Cygnus columbianus/Hubei/127/2021	H5N1	HP	2/3/4/5/6/7/8
48	EPI_ISL_11633709	2021-12-20	A/Cygnus columbianus/Hubei/128/2021	H5N1	HP	1/2/3/4/5/6/7/8
49	EPI_ISL_13243698	2021-12-22	A/duck/Bangladesh/19D1819/2021	H5N1	HP	1/2/3/4/5/6/7/8
50	EPI_ISL_9869760	2021-12-30	A/American wigeon/South Carolina/AH0195145/2	H5N1	HP	1/2/3/4/5/6/7/8
51	EPI_ISL_11330431	2022-01-02	A/white-tailed eagle/Hokkaido/22-RU-WTE-2/202	H5N1	HP	1/2/3/4/5/6/7/8
52	EPI_ISL_12572662	2022-01-05	A/goose/Hunan/SE284/2022	H5N1	HP	1/2/3/4/5/6/7/8
53	EPI_ISL_11561589	2022-02-02	A/chicken/England/011981/2022	H5N1	HP	1/2/3/4/5/6/7/8
54	EPI_ISL_11922811	2022-02-05	A/goose/Poland/H124_22VIR2515-5/2022	H5N1	HP	1/2/3/4/5/6/7/8
55	EPI_ISL_15647837	2022-02-06	A/duck/Korea/H125/2022	H5N1	HP	1/2/3/4/5/6/7/8
56	EPI_ISL_9909371	2022-02-07	A/turkey/Indiana/22-003707-003/2022	H5N1	HP	1/2/3/4/5/6/7/8
57	EPI_ISL_14615027	2022-03-14	A/crow/Hokkaido/0103L018/2022	H5N1	HP	1/2/3/4/5/6/7/8
58	EPI_ISL_13009694	2022-03-26	A/chicken/Wyoming/22-009599-002-original/2022	H5N1	HP	1/2/3/4/5/6/7/8
59	EPI_ISL_12174843	2022-03-29	A/crow/Hokkaido/0103B065/2022	H5N1	HP	1/2/3/4/5/6/7/8
60	EPI_ISL_12174842	2022-03-31	A/Ezo red fox/Hokkaido/1/2022	H5N1	HP	1/2/3/4/5/6/7/8
61	EPI_ISL_13117168	2022-05-05	A/Chicken/BC/FAV-0348-OS/2022	H5N1	HP	1/2/3/4/5/6/7/8

62	EPI_ISL_15072540	2022-05-18	A/crow/Hokkaido/HU-1/2022	H5N1	HP	1/2/3/4/5/6/7/8
63	EPI_ISL_14098920	2022-06-29	A/harbor seal/Maine/22-020983-001-original/202	H5N1	HP	1/2/3/4/5/6/7/8
64	EPI_ISL_15647836	2022-10-10	A/Mandarin/duck/Korea/WA496/2022	H5N1	HP	1/2/3/4/5/6/7/8
65	EPI_ISL_15647834	2022-10-17	A/duck/Korea/H493/2022	H5N1	HP	1/2/3/4/5/6/7/8
